# An Isochroman Analog of CD3254 and Allyl-, Isochroman-Analogs of NEt-TMN Prove to Be More Potent Retinoid-X-Receptor (RXR) Selective Agonists Than Bexarotene

**DOI:** 10.3390/ijms232416213

**Published:** 2022-12-19

**Authors:** Peter W. Jurutka, Orsola di Martino, Sabeeha Reshi, Sanchita Mallick, Michael A. Sausedo, Grant A. Moen, Isaac J. Lee, Dominic J. Ivan, Tyler D. Krall, Samuel J. Peoples, Anthony Perez, Lucas Tromba, Anh Le, Iraj Khadka, Ryan Petros, Brianna M. Savage, Eleine Salama, Jakline Salama, Joseph W. Ziller, Youngbin Noh, Ming-Yue Lee, Wei Liu, John S. Welch, Pamela A. Marshall, Carl E. Wagner

**Affiliations:** 1 School of Mathematical and Natural Sciences, Arizona State University, Glendale, AZ 85306, USA; 2 Department of Basic Medical Sciences, University of Arizona College of Medicine, Phoenix, AZ 85004, USA; 3 Department of Internal Medicine, Washington University, St. Louis, MO 63110, USA; 4 School of Molecular Sciences, Arizona State University, Tempe, AZ 85281, USA; 5 Department of Chemistry, University of California, Irvine, CA 92697, USA; 6 Poseida Therapeutics, Inc. 9390 Towne Centre Drive, Suite 200, San Diego, CA 92121, USA; 7 Cancer Center and Department of Pharmacology and Toxicology, Medical College of Wisconsin, Milwaukee, WI 53226, USA; 8 A2 Biotherapeutics, 30301 Agoura Rd, Agoura Hills, CA 91301, USA

**Keywords:** retinoid-x-receptor, retinoid, rexinoid, leukemia, small molecule therapeutic, structure-activity-relationship

## Abstract

Bexarotene is an FDA-approved drug for the treatment of cutaneous T-cell lymphoma (CTCL); however, its use provokes or disrupts other retinoid-X-receptor (RXR)-dependent nuclear receptor pathways and thereby incites side effects including hypothyroidism and raised triglycerides. Two novel bexarotene analogs, as well as three unique CD3254 analogs and thirteen novel NEt-TMN analogs, were synthesized and characterized for their ability to induce RXR agonism in comparison to bexarotene (**1**). Several analogs in all three groups possessed an isochroman ring substitution for the bexarotene aliphatic group. Analogs were modeled for RXR binding affinity, and EC_50_ as well as IC_50_ values were established for all analogs in a KMT2A-MLLT3 leukemia cell line. All analogs were assessed for liver-X-receptor (LXR) activity in an LXRE system to gauge the potential for the compounds to provoke raised triglycerides by increasing LXR activity, as well as to drive LXRE-mediated transcription of brain ApoE expression as a marker for potential therapeutic use in neurodegenerative disorders. Preliminary results suggest these compounds display a broad spectrum of off-target activities. However, many of the novel compounds were observed to be more potent than **1**. While some RXR agonists cross-signal the retinoic acid receptor (RAR), many of the rexinoids in this work displayed reduced RAR activity. The isochroman group did not appear to substantially reduce RXR activity on its own. The results of this study reveal that modifying potent, selective rexinoids like bexarotene, CD3254, and NEt-TMN can provide rexinoids with increased RXR selectivity, decreased potential for cross-signaling, and improved anti-proliferative characteristics in leukemia models compared to **1**.

## 1. Introduction

Nuclear receptors (NRs) are ligand-dependent transcription factors that bind DNA sequence-specific motifs in enhancers and promoters to transactivate their target genes [1]. The retinoid X receptors (RXRs) are ligand-activated NRs that have pleiotropic effects including the control of hematopoietic stem cell self-renewal and differentiation. There are three different isoforms of each receptor (α, β, and γ) that are differently expressed in mouse and human tissues [2,3]. The RXRs, often working in concert with other NRs regulate gene transcription through receptor-specific molecular signals. The RXRs are remarkably versatile compared to other NRs, since they partner with many of the NRs to form heterodimers that modulate cell differentiation, migration, proliferation, and metabolic pathways. Several critical NR pathways that are RXR-dependent include those regulated by the retinoic acid receptor (RAR), the vitamin D receptor (VDR), the peroxisome proliferator-activated receptor (PPAR), the thyroid hormone receptor (TR), the farnesoid X receptor (FXR), and the liver-X-receptor (LXR), to cite just a few. All NRs function as transcriptional modulators, most often promoting transcription as a result of the presence of corresponding receptor ligand in addition to any obligate receptor partner. The receptor ligands are often endogenous molecules that bind to a ligand-binding domain (LBD) in the receptor. This subsequently forces the receptor into a new conformation more conducive towards dimerizing with another receptor, recruiting associated co-factors, and finally binding to a high affinity hormone responsive element (HRE) specific to the genes the receptor controls in the DNA. While several HREs have been located proximal to or inside the promoter region of the regulated genes, HREs are increasingly being observed a considerable distance down- or upstream from the regulated genes. The HREs display a shared sequence specificity that includes two repeat hexads enclosing a specific quantity of spacers that separate those inverted, everted, or direct repeats [4]. RAR, TR, and VDR’s HREs comprise half-sites enclosing, respectively, five, four, and three nucleotide spacers [5,6].

Before RXR was well known, TR, RAR, and VDR were believed to assemble into homodimers [7] in order to bind their respective HREs, though heterodimerizing with RXR was later discovered to be the prerequisite for them to bind and activate their HREs [8]. The naturally occurring 9-cis-retinoic acid (9-cis-RA)—a geometric isomer of the all-trans-retinoic acid (ATRA)—was identified as an RXR-specific agonist (a rexinoid) by Zhang and coworkers, who documented that its binding to RXR’s LBD triggers RXR homodimerization and the subsequent association of the homodimer to the RXR responsive elements (RXREs) [9]. In other NR heterodimers where RXR is involved, the LBD of RXR may not necessarily need to possess a rexinoid. For instance, the VDR-RXR heterodimer functions absent a ligand or rexinoid binding to RXR [10]. Conversely, there are examples of RXR heterodimers where a rexinoid bound to RXR enhances that heterodimer’s activity, such as for the LXR-RXR heterodimer [11]. This remarkable versatility—where RXR can partner with many other NRs with and without rexinoids—has led to RXR’s classification as the indispensable, master receptor [12].

A great number of studies reported in the literature concerning RXR partnering with other NRs and comprising many rexinoids have been distilled to yield two primary classifications for RXR heterodimers—permissive and nonpermissive. For purely nonpermissive heterodimers of RXR, only the other NR’s agonist can activate the heterodimer, whereas permissive RXR heterodimers can be activated by either a rexinoid or the partnering NR’s agonists [13]. The RAR-RXR, TR-RXR, and VDR-RXR heterodimers are generally nonpermissive. In the majority, but not every instance, the partnering RXR receptor is “silent” in the TR and VDR heterodimers. The RAR-RXR heterodimer, meanwhile, displays increased activity in the presence of certain rexinoids as well as agonists specific for RAR. The presence of certain rexinoids have been shown to activate RAR-RXR despite the absence of agonists specific for RAR [14]. Thus, the classical idea of purely nonpermissive RXR heterodimers has evolved towards a spectrum of conditions for permissibility, such that some RXR heterodimers, such as RAR-RXR, could be more accurately described as conditionally nonpermissive. The LXR-RXRs, FXR-RXRs, and the PPAR-RXRs, however, are fully permissive.

Potent rexinoids can disrupt the proper functioning of both types of RXR heterodimers, giving rise to pleiotropy by stimulating activity in the permissive RXR heterodimers or by removing RXR from participation in the nonpermissive heterodimers. The tendency to exert pleotropic effects has blocked clinical development of many rexinoids for various therapeutic applications. Rexinoids like 9-cis-RA, for example, arrest activity for VDR-RXR [15,16,17] and TR-RXR [18]. Similarly, the 1,25-dihydroxyvitamin D_3_ (1,25D) and T3 promote formation of VDR-RXR and TR-RXR, respectively, and thus deplete RXR availability for other RXR-dependent pathways. This effect has been termed cross-receptor squelching and is manifested in the loss of TR function via (1,25D)-VDR-RXR-modulated inhibition [18,19], or similarly, in the loss of VDR activity paralleling T_3_-TR-RXR-activation [20,21]. However, more than just RXR depletion may account for this crosstalk inhibition. Accordingly, the potency and selectivity are the primary characteristics that must be considered in the development of novel rexinoids to minimize side effects and maximize therapeutic potential. Hence, the approach of slightly modifying the structural features of a parent rexinoid’s structure could impact both characteristics and generate rexinoids with less severe pleiotropy and greater specificity, resulting in specific NR modulators (SNuRMs) [22].

A number of rexinoid SNuRMs can be found in advanced stages of pre-clinical or clinical investigation as therapeutics, especially as preventative or treatment regimens for various cancers where selective RXR versus RAR activation exerts therapeutic effects and avoids RAR toxicities [23] in treating many human cancers. After multiple studies [24,25] that used 9-cis-RA as a starting point to model and design new compounds for RXR-selectivity, 4-[1-(3,5,5,8,8,-pentamethyltetralin-2-yl)ethynyl]benzoic acid (**1**) [26] was identified as a lead compound in terms of its stability and RXR-selectivity, though there were many additional candidates with equally promising profiles. After Ligand Pharmaceuticals Inc. earned FDA approval for **1** as a treatment for cutaneous T-cell lymphoma, the tradename of “bexarotene”—the more common name by which **1** is known—was assigned. Many studies making very minor modifications of the structure of bexarotene have identified structurally similar analogs of **1** that display similar profiles of activity in RXR-binding and activity—disilabexarotene (**2**) [27] is a representative example.

Even though bexarotene (**1**) is an approved treatment for CTCL, there are an increasing number of studies in other human cancers and cancer models such as lung [28], breast [29], and colon cancer [30]. In fact, bexarotene is prescribed in certain cases of non-small cell lung cancer off-label, since a proof-of-concept (POC) clinical trial showed benefits of **1** as a treatment [31,32]. Increasingly, studies are linking suppression of cell-proliferation and apoptosis synergy for combination-chemotherapeutic approaches that target RXR-controlled pathways. There are numerous studies in the literature for bexarotene (**1**) and analogous synthetic rexinoids exhibiting therapeutic effects for non-insulin-dependent diabetes mellitus murine models likely tied to metabolic impacts from RXR:PPAR activity [33]. While the selective activation of RXR by bexarotene avoids toxicities associated with RAR activity, humans dosed with **1** often suffer hyperlipidemia and hypothyroidism [34], and sometimes cutaneous toxicity, as the most significant side effects. These side effects arise because, much like 9-cis-RA, bexarotene (**1**) disrupts nonpermissive heterodimers—such as TR-RXR to incite hypothyroidism [35]—and concurrently stimulates permissive heterodimers—such as LXR-RXR to provoke hyperlipidemia [36,37] or cutaneous toxicity [38] via activating RAR at raised dose concentrations. It is difficult to dissociate the potential of a potent synthetic rexinoid to provoke increased activity in RXR’s permissive heterodimers from its selectivity for RXR alone, though many research groups are exploring this area. Developing potent rexinoids that mitigate permissive RXR heterodimer activity is a timely objective since **1** has exhibited potential to stem neurodegenerative progression in models of Alzheimer’s disease [39] (AD) as well as Parkinson’s disease (PD) [40]. A limited POC clinical trial for moderate AD patients treated with **1** or placebo revealed that non-apoE4 genotypes treated with **1** showed a reduction of soluble amyloid beta from cerebrospinal fluid that was statistically significant [41].

**Figure 1:**
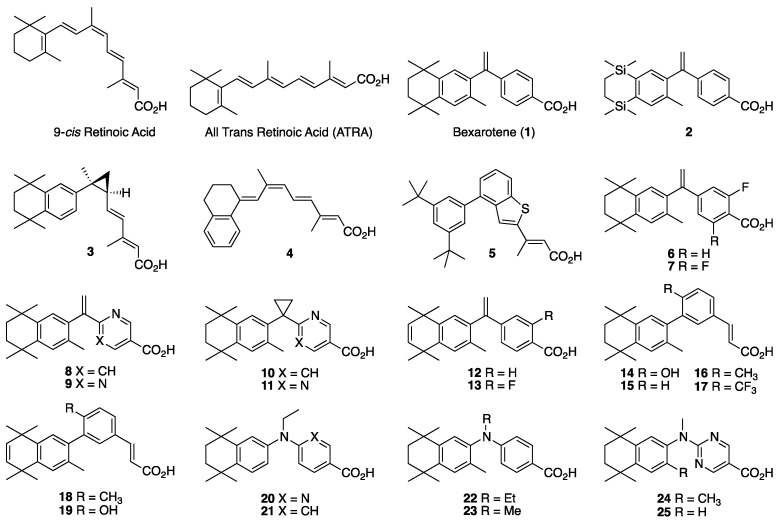
Structures of 9-*cis* Retinoic Acid, ATRA, Bexarotene (**1**), and Rexinoids **2–25**.

Many groups in this field have successfully used modeling and the structures of potent rexinoids in the literature as the basis to develop new rexinoids that display unique profiles. One well-known rexinoid that is in phase II clinical trials for prostate cancer [42] and pre-clinical trials for PD, AD, and multiple sclerosis is IRX-4204 (**3**) [43], a chiral rexinoid shown to be more selective and potent for RXR than its enantiomer. The 9cUAB30 (**4**) [44] is another rexinoid in clinical trials for breast cancer [45,46,47], and studies of its methylated analogs [48,49] have helped elucidate the reasons that **4** does not provoke hyperlipidemia via LXR-RXR agonism. Boehm’s group reported unbranched trienes terminating in carboxylic acids [50], along with analogs incorporating one [51] or fused aryl ring-systems [52]—in the case of the latter, compound **5 [52]** is an example. When our group first entered the field, we reported a fluorinated bexarotene analog (**6**) [53] followed by other halogenated, and even a difluorinated bexarotene analog (**7**) [54]. The pyridine bexarotene analog (**8**) [55] and the pyrimidine bexarotene analog (**9**) [56], as well as LGD100268 (**10**) [55] and the LGD100268 pyrimidine analog (**11**) [56] all showed enhanced RXR activity compared to **1** and superior therapeutic effects in mouse models of cancer [28,57]. Installing an unsaturation in bexarotene’s aliphatic ring results in compound **12 [58,59]**, and the unsaturated-fluorinated bexarotene (**13**) [56] also activates LXR [60]. CD3254 (**14**) [61] and CD2915 (**15**) [62] are two synthetic rexinoids with activity comparable to **1**. Our group utilized **14** and **15** to design analogous rexinoids **16–19 [56]**. Kakuta and colleagues reported compound **20** (NEt-TMN) [63,64,65,66] as well as its analogous compounds **21** [67,68,69] and **22 [67,68]**—all of which showed high potency and selectivity for RXR alongside many other NEt-TMN derivatives that our group reported [70]. Even replacing the ethyl group with a methyl group on the linking nitrogen atom of NEt-TMN leads to potent rexinoids such as **23** [71], **24** [71], and **25** [71] (Figure 1).

**Figure 2:**
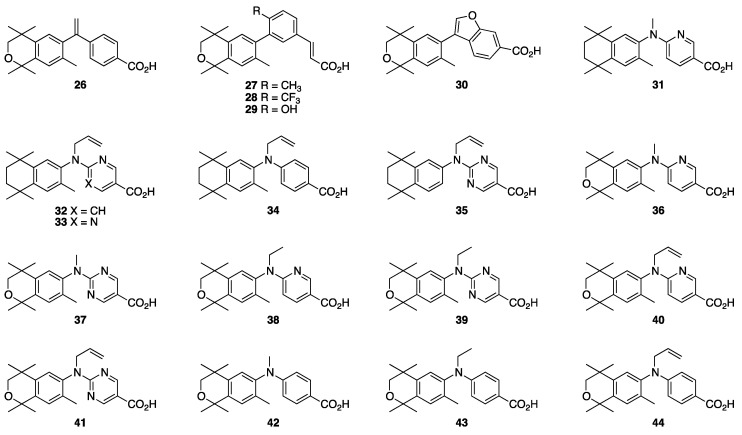
Structures of Reported Rexinoids **3–27**.

The current study uses many of the above compounds cited above as starting points to investigate how introducing new modifications result in changes to the compounds’ activities. For example, we were interested in substituting an isochroman group for the aliphatic ring system in bexarotene and some of the CD3254 analogs that have been reported, as well as a multiple fused aryl-ring system, and hence we targeted the synthesis of rexinoids **26–30**. We were also interested in exploring a pyridine aromatic ring substitution from reported analogs **23–25**, so we targeted the synthesis of **31**. Due to the potency of NEt-TMN (**20**) and its analogs, we were curious about the impacts that substituting an allyl group, varying aromatic rings, and adding a methyl group would have on RXR activity for the new rexinoids, so we targeted the synthesis of **32–35**. Finally, we were interested in substituting the isochroman group for the aliphatic ring system of NEt-TMN and then varying the *N*-alkyl chains—including methy, ethyl and allyl—along with different aromatic acid ring systems, and so we targeted **36–44** for synthesis (Figure 2). Interestingly, compound **34 [67]** was previously made and disclosed by Kagechika and co-workers, so we were eager to synthesize several possible analogs of it (**32**, **33**, **35**, **40**, **41**, and **44**) and compare their activities.

While the isochroman group places a fairly polar, hydrogen-bonding oxygen atom in the nonpolar aliphatic ring system for the known parent rexinoids, we hypothesized that it would not disrupt the largely non-polar binding interaction in the RXR LBD. Further, we hypothesized that the isochroman group would make the rexinoids possessing it more resistant to metabolic oxidation. If the isochroman rexinoids were as potent and selective as their non-isochroman counterparts, we also hypothesized that they would possess similar in vitro activities as their parent compounds. Importantly, we wanted to assess these specific hypotheses about the incorporation of an isochroman group across multiple parent compound structures that include bexarotene (**1**), CD3254 (**14**), and various NEt-TMN (**20**) analogs coupled with other potential modifications to assess similarities or differences in the activities of the resulting analogs and their predecessors. Hence, we proceeded with the synthesis and testing of these compounds.

## 2. Results: Molecular Modeling

**Figure 3:**
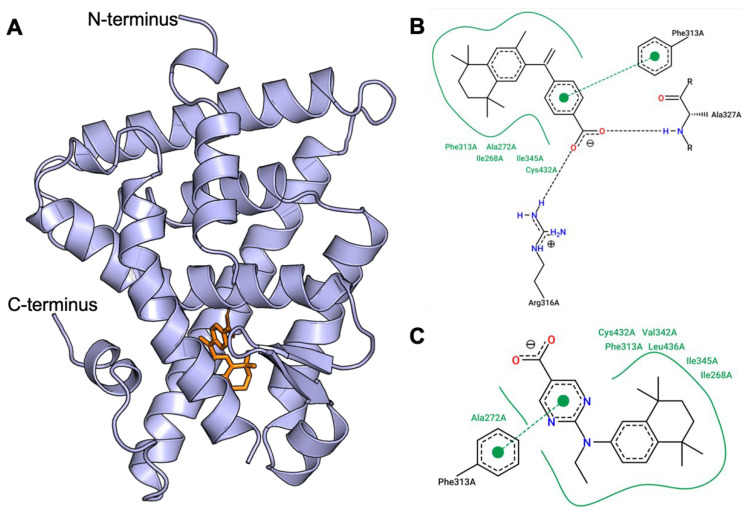
AutoDock Vina simulation of bexarotene bound to human RXR protein. (**A**) Cartoon representation of the human RXR alpha ligand binding domain (PDB:1FBY, blue) and the docked compound bexarotene (orange). N- and C-termini are labeled. (**B**,**C**) Two-dimensional depiction of the interactions between protein residue sidechains with bexarotene (**B**) and compound **26** (**C**) using PoseView (BioSolvIT [73,74]). In both (**B**) and (**C**), hydrogen bonds are presented as dashed lines between interaction partners, and hydrophobic interactions are depicted as smooth contour lines.

The binding affinity, predicted using AutoDock Vina [72], of human-RXR for each ligand is output as an energy unit (in kcal/mol, Table 1). These predicted ligand-bound RXR complexes were then visually inspected using PyMol (version 2.3, Shrodinger, LLC) (Figure 3). To further analyze and illustrate the interactions between RXR protein residue sidechains with the ligands, PoseView (BioSolvIT [73,74]) was used to generate the detailed two-dimensional depictions (Figure 3B,C). In these two-dimensional depictions, hydrogen bonds are presented as dashed lines between interaction partners, and hydrophobic interactions are depicted as smooth contour lines.

The AutoDock Vina docking results showed that the standard compound bexarotene (**1**), with a score of −12.7 kcal/mol, was the most potent among all compounds. Compound **26** has a comparable score of −12.4 kcal/mol. Additionally, similar interactions with RXR residue sidechains were observed between **1** and **26** such as direct interactions between Phe313 and the aromatic cores of either ligand, as well as hydrophobic residues Ile268, Ile345, and Ala272 bearing common interactions (Figure 3B,C). Several other compounds have similar but slightly lower scores of −11.9 kcal/mol (**30**), −11.7 kcal/mol (**28**), −11.6 kcal/mol (**34**), and −11.5 kcal/mol (**23**, **29**, **31**, **32**, **42**, **43**, and **44**) (Table 1). Therefore, we can conclude that RXR compounds with a docking score within 10% that of bexarotene could potentially possess comparable, or better, EC_50_ and IC_50_ profiles for further study.

## 3. Results: Chemistry

**Scheme 1:**
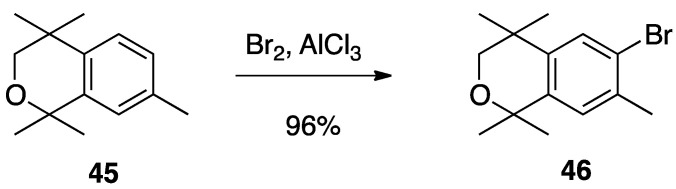
Synthesis of bromo-isochroman **46**.

The synthesis of compound **26** begins with the bromination of commercially available 1,1,4,4,7-pentamethylisochroman (**45**) with bromine in dichloromethane and aluminum chloride catalyst to give 6-bromo-1,1,4,4,7-pentamethylisochroman (**46**) in 96% yield (Scheme 1) [75].

**Scheme 2:**
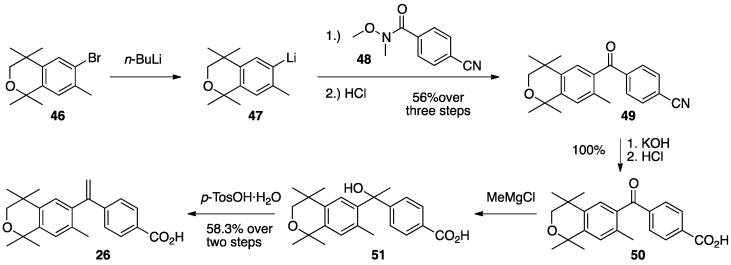
Conversion of **46** to lithium-isochroman **47**, its reaction with amide-nitrile **48** to give ketone-nitrile **49** that was subsequently hydrolyzed to ketone-acid **50** which was converted to alcohol-acid **51** that was finally dehydrated to isochroman acid **26**.

Compound **46** was lithiated by treatment with n-butyllithium, and the resulting aryl lithium reagent (**47**) was transferred to a solution of the reported amide **48 [59]** in THF to provide ketone (**49**) in 56% purified yield whose nitrile group is then hydrolyzed to a carboxylic acid (**50**) in quantitative yield. Compound **50**, when treated with more than two equivalents of methylmagnesium chloride is converted to alcohol **51** that is then dehydrated to compound **26** in 58.3% over two steps (Scheme 2).

**Figure 4:**
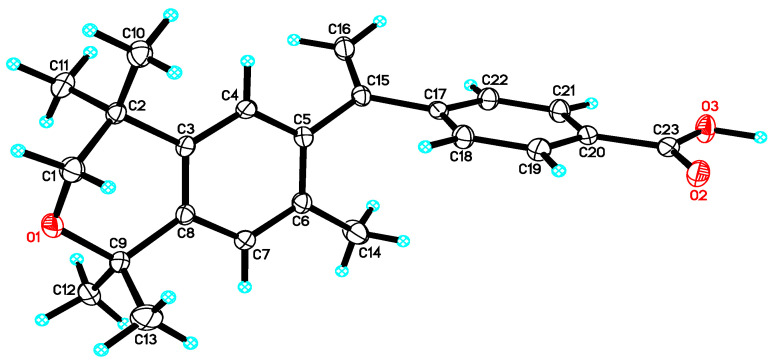
X-ray crystal structure of a 0.114 *×* 0.141 × 0.380 mm^3^ crystal of **26**. Thermal ellipsoids are shown at 50% probability level.

Acid **26** formed single, transparent crystals, so an X-ray diffraction study was conducted to confirm the structure of **26** (Figure 4).

**Scheme 3:**
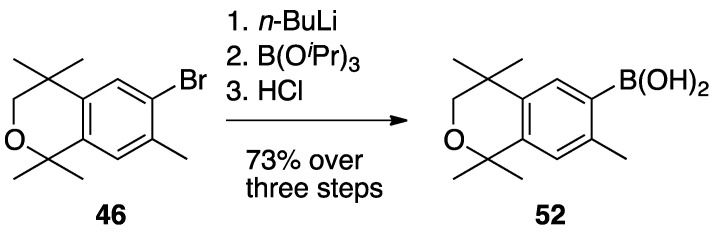
Synthesis of boronic acid **52**.

To synthesize compound **27**, compound **46** was treated with n-butyllithium and then triisopropylborate to give **52** in 73% yield (Scheme 3).

**Scheme 4:**
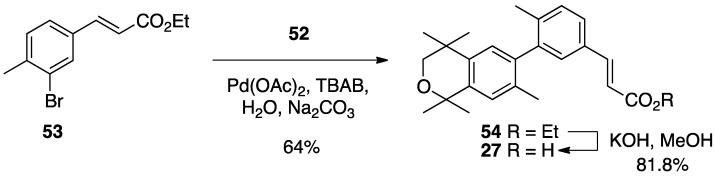
Synthesis of biphenyl **54** and its saponification to give acid **27**.

The known ethyl ester **53** [56] was combined with boronic acid **53** along with Pd(II) diacetate, TBAB, and sodium carbonate in water and heated to boiling for 5 min to give **54** in 64% yield, and ethyl ester **54** was saponified with KOH in methanol to give **27** in 81.8% yield after acidification with 1N HCl, filtration, and purification by column chromatography (Scheme 4).

**Scheme 5:**
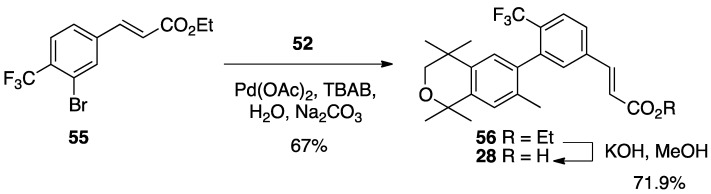
Synthesis of biphenyl **56** and its saponification to give acid **28**.

A similar procedure was used to make compounds **28**, **29**, and **30**. For compound **28**, the known ethyl ester **55** [56] was combined with boronic acid **52** along with Pd(II) diacetate, TBAB, and sodium carbonate in water and heated to boiling for 5 min to give **56** in 67% yield, and ethyl ester **56** was saponified with KOH in methanol to give **28** in 71.9% yield after acidification with 1N HCl, filtration, and purification by column chromatography (Scheme 5).

**Scheme 6:**
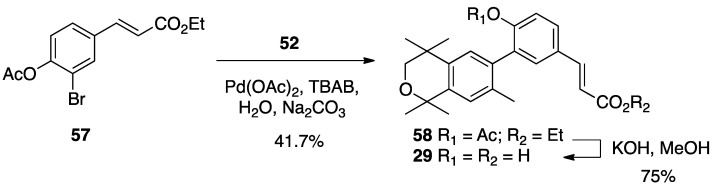
Synthesis of biphenyl **58** and its saponification to give acid **29**.

For compound **5**, the known ethyl ester **57 [56]** was combined with boronic acid **52** along with Pd(II) diacetate, TBAB, and sodium carbonate in water and heated to boiling for 5 min to give **58** in 41.7% yield, and ethyl ester **58** was saponified with KOH in methanol to give **29** in 75% yield after acidification with 1N HCl, filtration, and purification by column chromatography (Scheme 6).

**Scheme 7:**
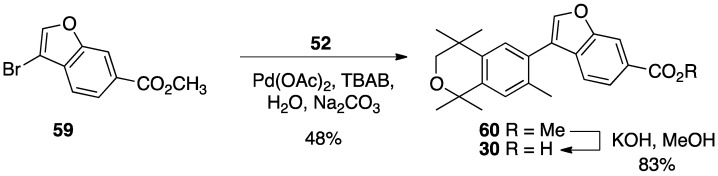
Synthesis of methyl ester **60** and its saponification to give acid **30**.

For compound **30**, the commercially available methyl ester **59** was combined with boronic acid **52** along with Pd(II) diacetate, TBAB, and sodium carbonate in water and heated to boiling for 5 min to give **60** in 48% yield, and methyl ester **60** was saponified with KOH in methanol to give **30** in 83% yield after acidification with 1N HCl, filtration, and purification by column chromatography (Scheme 7).

**Scheme 8:**
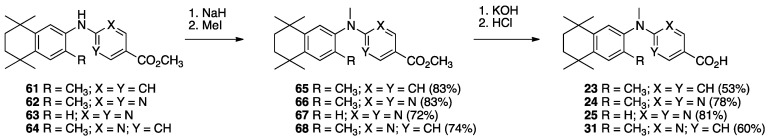
Synthesis of methyl esters **65**–**68** and their saponification to give acids **23**–**25** and **31**.

The synthesis of known compounds **23**, **24**, **25**, and novel compound **31** begins with the conversion of known compounds **61–64 [70]** to compounds **65–68** by treatment with sodium hydride followed by methyl iodide in DMF with stirring at room temperature in good yields. The methyl esters **65–68** were saponified by treatment with KOH in methanol followed by acidification and purification to give compounds **23–25** and **31**, respectively, in moderate to good yields (Scheme 8).

**Scheme 9:**
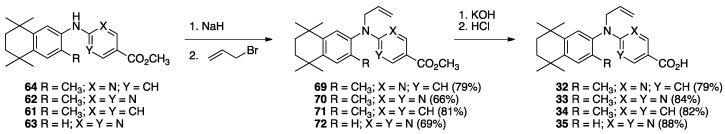
Synthesis of methyl esters **69**–**72** and their saponification to give acids **32**–**35**.

The synthesis of compounds **32–35** begins with the conversion of known compounds [70] **64**, **62**, **61**, and **63** to compounds **69–72** by treatment with sodium hydride followed by allyl bromide in DMF with stirring at room temperature in good yields. The methyl esters **69–72** were saponified by treatment with KOH in methanol followed by acidification and purification to give compounds **32–35** in moderate to good yields (Scheme 9).

**Scheme 10:**
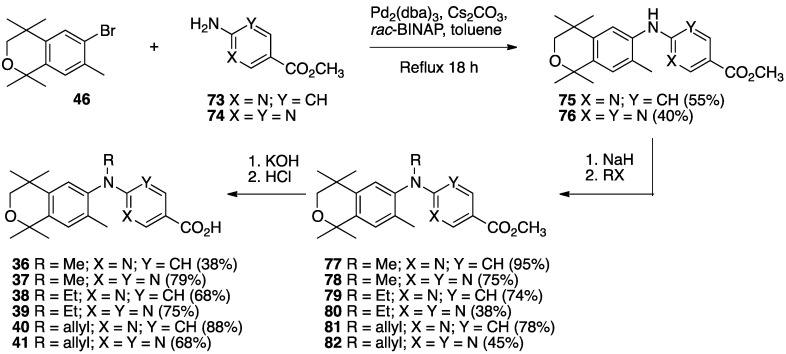
Synthesis of diaryl amines **75**–**76**, their alkylation to give methyl esters **77**–**82** and their saponification to give acids **36**–**41**.

The synthesis of compounds **36–41** begins with the palladium-catalyzed reaction of the known aryl bromide **46** with methyl 6-aminonicotinate (**73**) or methyl 2-aminopyrimidine-5-carboxylate (**74**) to give diaryl amines **75** or **76** in moderate yields of 55% and 40%, respectively. The diaryl amines **75** and **76** were treated with sodium hydride followed by methyl iodide, ethyl iodide, or allyl bromide to give methyl esters **77–82** in moderate to good yields which were then saponified by treatment with KOH in methanol followed by acidification and purification to give compounds **36–41** in moderate to good yields (Scheme 10).

**Figure 5:**
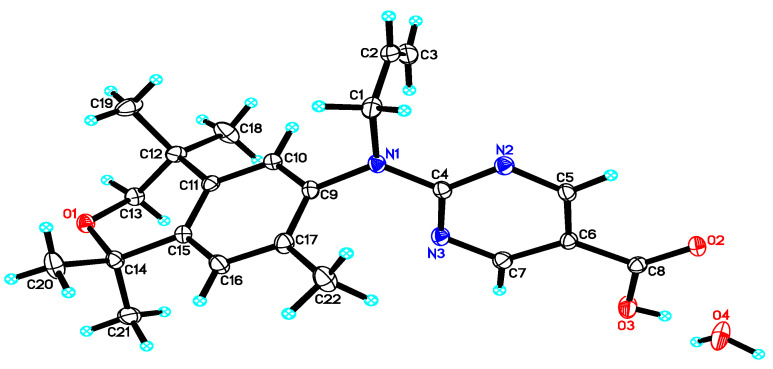
X-ray crystal structure of a 0.184 × 0.217 × 0.371 mm^3^ crystal of compound **41** as a monohydrate. Thermal ellipsoids are shown at 50% probability level.

Acid **41** formed single, transparent crystals from d6-DMSO, so an X-ray diffraction study was undertaken to confirm the structure for **41** (Figure 5).

**Scheme 11:**
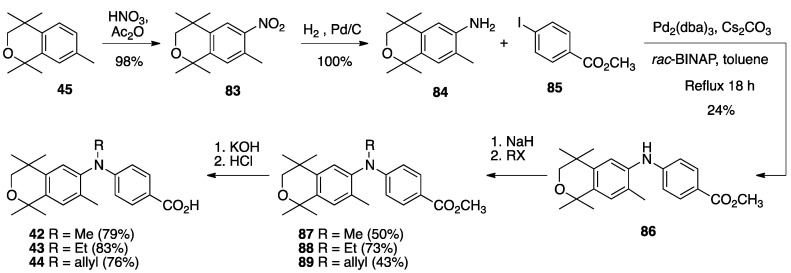
Synthesis of nitro-isochroman **83**, its reduction to aniline **84** and subsequent coupling with **85** to provide **86** and its subsequent alkylation to give methyl esters **87**–**89** and their saponification to yield acids **42**–**44**.

Finally, the synthesis of novel rexinoids **42–44** begins with the nitration of isochroman (**45**) to give **83** in 98% yield, followed by the hydrogenation of **83** to give aryl amine **84** in quantitative yield. Aryl amine **84** is then combined with 4-iodomethylbenzoate (**85**) in a palladium-catalyzed reaction to give **86** in 24% yield. Diaryl amine **86** is then treated with sodium hydride followed by methyl iodide, ethyl iodide, or allyl bromide to give methyl esters **87–89** in moderate to good yields and the methyl esters are saponified with potassium hydroxide in methanol followed by acid work-up to give rexinoids **42–44** in good yields after purification (Scheme 11).

## 4. Results and Discussion: Biological Assays

Bexarotene (**1**) and the isochroman and other analogs of bexarotene, CD3254, and NEt-TMN **26–44** were tested in a KMT2A-MLLT3 cell line to determine RXR*α* activation EC50 values by both a Luc– and GFP-assay, and then IC50 values for a 96 h cell viability assay were determined both in the presence and absence of 100 nM ATRA and the resulting data is tabulated in Table 1. Compounds **1** and **26**–**44** were examined for cytotoxicity and mutagenicity in Saccharomyces cerevisiae [76]. None of the compounds were mutagenic or cytotoxic.

**Figure 6:**
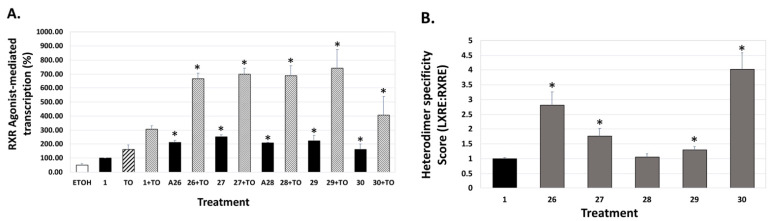
**RXR agonist potentiation of LXRE-regulated transactivation with or without the T0901317 LXR agonist**. (**A**) U87 glial cells were transfected with an expression vector for human LXRα, an LXRE-luciferase reporter gene with three tandem copies of the LXRE from the human ApoE gene, and a Renilla control plasmid. Cells were transfected for 24 h utilizing a liposome-mediated transfection protocol and then treated with ethanol vehicle, or 100 nM of the indicated compound alone or in combination with 100 nM TO901317 (TO). LXRE-directed activity was compared to compound 1 (Bexarotene), set to 100%. (**B**) The “Heterodimer Specificity Score” was determined by the LXRE:RXRE ratio with compound 1 set to 1.0. All error bars represent standard deviations; the data are representative of at least three independent experiments with six replicates in each treatment group. * *p* < 0.05 versus control compound **1**.

We also evaluated the analogs for their capacity to bind and activate the liver-X-receptor (LXR) using a liver-X-receptor responsive element (LXRE)-based assay, and assessed the effects in the presence vs. absence of an authentic LXR compound (TO901317), as well as with bexarotene alone. LXR is known to regulate inflammatory responses and lipid metabolism in multiple tissues, including the central nervous system, and there is sufficient evidence that strong cholesterol and lipid metabolism in the brain, along with enhanced ApoE expression, is paramount to reducing the risk of human dementias. The *“*activity assessment*”* of our novel RXR agonists for their ability to enhance gene expression using a DNA responsive element sequence (LXRE) that is found to occur in the natural human promoter of LXR-RXR controlled genes (such as ApoE) was carried out in U87 glial cells with bexarotene (**1**) as a comparative control. Using the above assay system, the activation from this natural LXRE was probed with either 100 nM RXR analogs (or bexarotene) alone or in combination with 100 nM of both the RXR agonist and an LXR ligand T0901317 (TO). The employment of both LXR and RXR agonists collectively was anticipated to demonstrate a more vigorous response in LXRE transactivation due to cumulative and/or synergistic effects of dual ligand activation of the RXR-LXR heterodimer. The results demonstrated that in comparison to the parent bexarotene (**1**) compound alone, separate dosing of the cells with analogs **26**, **27**, **28**, **29**, and **30** displayed more LXR/LXRE activity (Figure 6A, *p* < 0.05).

**Figure 7:**
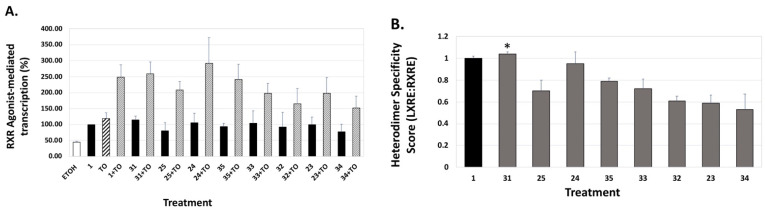
**RXR agonist potentiation of LXRE-regulated transactivation with or without the T0901317 LXR agonist.** (**A**) U87 glial cells were transfected with an expression vector for human LXRα, an LXRE-luciferase reporter gene with three tandem copies of the LXRE from the human ApoE gene, and a Renilla control plasmid. Cells were transfected for 24 h utilizing a liposome-mediated transfection protocol and then treated with ethanol vehicle, or 100 nM of the indicated compound alone or in combination with 100 nM TO901317 (TO). LXRE-directed activity was compared to compound 1 (Bexarotene), set to 100%. (**B**) The “Heterodimer Specificity Score” was determined by the LXRE:RXRE ratio with compound 1 set to 1.0. All error bars represent standard deviations; the data are representative of at least three independent experiments with six replicates in each treatment group. * *p* < 0.05 versus control compound **1**.

**Figure 8:**
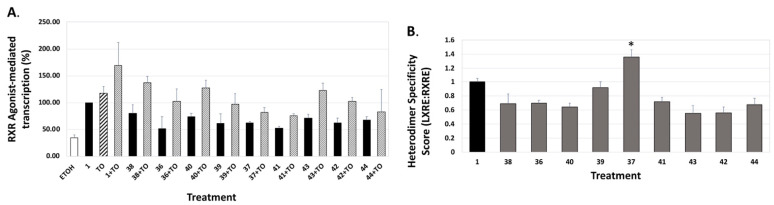
**Evaluation of RXR agonists to potentiate LXRE-mediated transactivation in the absence and presence of LXR ligand T0901317**. (**A**) U87 glial cells were transfected with an expression vector for human LXRα, an LXRE-luciferase reporter gene with three tandem copies of the LXRE from the human ApoE gene, and a Renilla control plasmid. Cells were transfected for 24 h utilizing a liposome-mediated transfection protocol and then treated with ethanol vehicle, or 100 nM of the indicated compound alone or in combination with 100 nM TO901317 (TO). LXRE-directed activity was compared to compound 1 (Bexarotene), set to 100%. (**B**) The “Heterodimer Specificity Score” was determined by the LXRE:RXRE ratio with compound 1 set to 1.0. All error bars represent standard deviations; the data are representative of at least three independent experiments with six replicates in each treatment group. * *p* < 0.05 versus control compound 1.

Specifically, the analogs displayed activities ranging from 52% to 254% of the bexarotene control (set to 100%; Table 1). Furthermore, when the LXR synthetic ligand (TO) was used in combination with bexarotene (**1**) or analogs, similar results were observed (Figure 6A, Figure 7A, and Figure 8A, stippled bars). Although some of the analogs exhibit lower LXR activation when compared to bexarotene (**1**), it is imperative to evaluate this activity in the background of the RXR-RXR homodimer activity of each analog, and to thus “normalize” the LXR/LXRE heterodimer activation results. The LXRE Heterodimer Specificity (LHS) score (Figure 6B, Figure 7B, and Figure 8B) is thus calculated based on both the RXR homodimer activity, as well as the LXR heterodimer activation. The results of this LHS analysis (Table 1) reveal that many of our novel compounds (e.g., **26**, **27**, **29**, **30**, **31**, and **37**, *p* < 0.05) display enhanced LXR/LXRE activity via increased heterodimer specificity compared to the parent bexarotene (**1**).

Finally, even though compound **1** is selective for binding to RXR, it does include some “residual” RAR activity. We assessed the potential of our analogs to induce transcription via the retinoic acid response element (RARE) and retinoic acid receptor. For these studies, we employed human embryonic cells (HEK293) that were transfected with human RARα and dosed with 10 nM of either all-trans retinoic acid (ATRA), the endogenous ligand for RARα, compound **1**, or our panel of analogs. The results of the assay revealed that compound **1** displayed an average of 37% of the activity of the ATRA control (set to 100%, Table 1). Analog **32** exhibited the greatest RARE activation at 62% of ATRA, while analog **37** showed the lowest RARE activity at 4%, which is indistinguishable from the ethanol control (Table 1). Importantly, most of our novel analogs (14 out of 18) possess attenuated “cross-over” onto RAR-RARE signaling compared to bexarotene (**1**).

## 5. Conclusions

While the substitution of the saturated, pentamethyl-tetrahydronaphthyl ring system of bexarotene, CD3254, or Net-TMN-analog parent compound frameworks with a pentamethyl-isochroman ring system tended to decrease both modeled binding scores as well as RXR EC50 values, there were nevertheless some examples of very potent compounds possessing the pentamethyl-isochroman ring system. Several reported and novel compounds displayed lower or equivalent EC50 values than bexarotene (e.g., reported compounds **23–25** and novel compounds **29**, **31–33**, **35**, and **40**), and several reported and novel compounds exhibited lower IC50 values along with 100 nM ATRA (e.g., reported compounds **23** and **24**, and novel compounds **29** and **31–35**) than bexarotene. It should be noted that compound **29** is an isochroman analog of CD3254 and compound **40** is an isochroman analog of NEt-TMN, so while the isochroman substituted analog tends to reduce potency compared to the parent compounds, rexinoids possessing the isochroman group can still be synthesized that exhibit greater potency than bexarotene. Notably, some of the compounds possessing the greatest RARE activity at 10 nM vs. ATRA also exhibited lower IC50 values along with 100 nM ATRA (e.g., **29** and **31–33**). Additionally, the range of LXRE activity vs. bexarotene demonstrated that several compounds exhibit increased LXR activity and specificity vs. bexarotene. In particular, compounds **29** and **31–35** appear to be good candidates for further investigation, and this study suggests that modification of known, potent rexinoids results in novel compounds with unique and promising therapeutic potential meriting further examination. We are actively pursuing additional studies towards these ends.

## 6. Materials and Methods

**Molecular Modeling**. The three-dimensional structures of the compounds reported herein were generated using ChemDraw 3D (PerkinElmer Informatics, Waltham, MA, USA), energy minimized, and exported in the Protein Data Bank (PDB) format. The human RXR alpha ligand binding domain structure model was obtained from the PDB (PDB code: 1FBY [77]). The crystallized ligand, 9-cis retinoic acid, was removed from the protein model prior to docking simulations. Further, 9-cis retinoic acid was also used as a positive control in the docking studies presented here. Both the protein and ligand models were prepared using MGLTools (version 1.5.7) [78] and screened virtually using AutoDock Vina [72]. The search space volume (4032 Å3) was determined using MGLTools (center_x = 12.848, center_y = 29.174, center_z = 50.269, size_x = 16, size_y = 14, size_z = 18). The exhaustiveness was set to 8.

**Hematopoietic cell culture**. Murine bone marrow Kit^+^ cells were isolated using an Automacs Pro (Miltenyi Biotec, San Diego, CA, USA) according to the manufacturer’s protocol. Kit^+^ cells were plated in progenitor expansion medium (RPMI 1640 medium, 15% fetal bovine serum, stem factor [50 ng/mL], interleukin 3 [10 ng/mL], Flt3L [25 ng/mL], thrombopoietin [10 ng/mL], L-glutamine [2 mM], sodium pyruvate [1 mM], HEPES buffer [10 mM], penicillin/streptomycin [100 units/mL], and b-mercaptoethanol [50 mM]) overnight and transduced with MSCV-KMT2A-MLLT3 retrovirus by spinfection with 10 μg/mL polybrene and 10 mM HEPES at 2400 rpm, 30 °C for 90 min in an Eppendorf 5810R centrifuge. Cells were transplanted into sublethally irradiated mice and subsequent leukemia harvested 4 to 6 months later. KMT2A-MLLT3 leukemia cells were cultured in vitro using similar media, but without Flt3L or thrombopoietin.

**UAS/Gal4 assay**. Bone marrow cells from UAS-GFP mice [79] were transduced with retroviruses MSCV-Gal4 (DNA binding domain, DBD)*—*RXR*α* (ligand binding domain, LBD)*—*IRES*—*mCherry. Gal4 is a yeast transcription factor and the UAS sequence is not recognized by mammalian transcription factors. Cells were treated, and after 48 h, GFP was measured by flow cytometry.

**Luciferase detection**. HEK293T cells were transfected using Lipofectamine 2000 (Invitrogen) [80]. Six hours after transfection, the cells were collected and plated into a 48-well plate in 1% BSA media in biological triplicates and treated with compounds. After 40 h incubation, the cells were harvested and assayed for luciferase (Luc Assay System with Reporter Lysis Buffer, Promega) in a Beckman Coulter LD400 plate reader.

**LXRE assay**. Human glioblastoma cells (U87) were purchased from American Type Culture Collection (ATCC, Manassas, VA, USA) and used to perform the LXRE-mediated assays. Authentication and validation of these master stocks included both testing for mycoplasma contamination (via Universal Mycoplasma Detection Kit; ATCC, Cat. #30-1012K), as well as short tandem repeat (STR) analysis to confirm cell line identity. For LXRE experiments*,* cells were seeded at a density of 80,000 cells/well in a 24-well plate and maintained in DMEM (Hyclone) supplemented with 10% fetal bovine serum, 100 µg/mL streptomycin, 100 U/mL penicillin (Invitrogen, Carlsbad, CA, USA) at 37 degrees Celsius, 5% CO2 for 24 h. The cells were transiently transfected in individual wells using Polyethylenimine (PEI) (Polysciences, Inc., Warrington, PA, USA) according to the manufacturer’s protocol. The cells in each well received 250 ng of an LXRE-luciferase reporter gene, 50 ng of CMX-h-LXRα (an expression vector for human LXRα), 50 ng of pSG5-human RXRα (an expression vector for human RXRα), and 20 ng of Renilla control plasmid was used along with 1.25 µL of PEI reagent. After 22–24 h of transfection, the cells were treated with either vehicle control ethanol, reference compound bexarotene (100 nM) alone, or in combination with 100 nM T0901317 (an LXR ligand), or 100 nM of the indicated bexarotene analog either alone or in combination with T0901317, as indicated. All compounds were solubilized in ethanol. After 24 h of treatment, the cells were lysed in 1X passive lysis buffer (Promega, Madison, WI USA) and the amount of reporter gene product (luciferase) was quantified using the Dual-Luciferase Reporter Assay System based on the manufacturer*’*s protocol (Promega) in a Sirius FB12 luminometer (Berthold Detection Systems, Pforzheim, Germany). Luminescence resulting from the inducible firefly luciferase was divided by luminescence from the constitutively expressed Renilla luciferase in order to normalize for transfection efficacy, cell death, and cellular toxicity from ligand exposure. The data are a compilation of between six to eight independent assays with each treatment group dosed in triplicate for each independent assay. The LXRE-directed transcriptional activation of the reporter gene was measured in comparison to the reference compound bexarotene (**1**) set to 100%. Error bars on all graphs indicate the standard deviation of the replicate experiments.

**RARE assay**. Human embryonic kidney cells (HEK293) were plated at 60,000 cells per well in a 24-well plate and maintained as described above. After 22–24 h, the cells were transiently transfected with 250 ng pTK-DR5(X2)-Luc, 25 ng pSG5-human RXRα, and 20 ng of Renilla control plasmid using 1.25 µL polyethylenimine (PEI) per well for 24 h. The sequence of the double DR5 RARE is: 5*’*-AAAGGTCACCGAAAGGTCACCATCCCGGGAGGTCACCGAAAGGTCACC-3*’* (DR5 responsive elements underlined). After 22–24 h of transfection, the cells were treated with ethanol vehicle (0.1%), all-trans-retinoic acid (ATRA, the endogenous ligand for RAR), or the indicated rexinoid analog at a final concentration of 10 nM. After 24 h of treatment, the cells were lysed and the retinoid activity was measured as described above (dual luciferase assay). The activity of compound **1** (or analog) divided by the activity of ATRA (expressed as a percentage) represents the RARE activity. The data (Table 1) are a compilation of between three to four independent assays with each treatment group dosed in triplicate for each independent experiment. The value for the positive control ATRA was set to 100%.

**Data analysis**. Statistical analysis was performed using Microsoft Excel (Microsoft, Redmond, WA, USA) software. *t*-tests were performed, as appropriate. All error bars represent the standard deviation. For Figure 6, Figure 7 and Figure 8, data are expressed as means ± SD. Statistical differences between two groups (generally the bexarotene (**1**) control group versus bexarotene analog group) were determined by a two-sided Student’s *t*-test. A *p-*value of less than or equal to 0.05 was considered significant.

**Mutagenicity and Toxicity Assay**. Mutagenicity and toxicity were assessed in a eukaryotic Saccharomyces cerevisiae model as described previously [76]. Each compound was solubilized in DMSO at increasing concentrations. D7 *S. cerevisiae* indicator cells were incubated with the compounds for 3 h before plating on selective media or YPD. Growth on plates was used to determine mutagenicity and cytotoxicity. Growth of colonies on the full nutrient YPD plate for each treatment was compared to the DMSO only control. Eleven micrograms per microliter was the highest concentration tested.

**X-ray Data Collection, Structure Solution and Refinement**. A colorless crystal of indicated dimensions was mounted on a glass fiber and transferred to a Bruker SMART APEX II diffractometer system. The APEX2 (APEX2 Version 2014.11-0, Bruker AXS, Inc.; Madison, WI 2014 USA) program package was used to determine the unit-cell parameters and for data collection (60 sec/frame scan time). The raw frame data were processed using SAINT (SAINT Version 8.34a, Bruker AXS, Inc.; Madison, WI 2013) and SADABS (Sheldrick, G.M. SADABS, Version 2014/5, Bruker AXS, Inc.; Madison, WI 2014 USA) to yield the reflection data file. Subsequent calculations were carried out using the SHELXTL (Sheldrick, G.M. SHELXTL, Version 2014/7, Bruker AXS, Inc.; Madison, WI 2014 USA) program package. For compound **26**, the diffraction symmetry was 2/m and the systematic absences were consistent with the monoclinic space group *P*2_1_/n that was later determined to be correct. The structure for **26** was solved by direct methods and refined on F^2^ by full-matrix least-squares techniques, and the analytical scattering factors [81] for neutral atoms were used throughout the analysis. Hydrogen atoms in **26**, except those associated with C(14), were located from a difference-Fourier map and refined (x,y,z and U_iso_), and H(14A), H(14B), and H(14C) were included using a riding model. Least-squares analysis for **26** yielded wR2 = 0.1064 and Goof = 1.025 for 328 variables refined against 3880 data (0.80 Å), R1 = 0.0426 for those 3293 data with I > 2.0*σ*(I). For compound **41**, the diffraction symmetry was 2/m and the systematic absences were consistent with the monoclinic space groups Cc and C2/c, and it was later determined that space group Cc was correct. The structure for **41** was solved by direct methods and refined on F^2^ by full-matrix least-squares techniques, and again, the analytical scattering factors for neutral atoms were used throughout the analysis. Hydrogen atoms in **41** were located from a difference-Fourier map and refined (x,y,z and U_iso_), and there was one water solvent molecule present. Least-squares analysis for **41** yielded wR2 = 0.0792 and Goof = 1.022 for 378 variables refined against 5116 data (0.73 Å), R1 = 0.0322 for those 4861 data with I > 2.0*σ*(I), and the absolute structure could not be assigned by refinement of the Flack parameter [82]. The wR2 = [Σ[w(F_o_^2^ − F_c_^2^)^2^]/Σ[w(F_o_^2^)^2^] ]^1/2^ and R1 = Σ||F_o_| − |F_c_||*/*Σ|F_o_|. Goof = S = [Σ[w(F_o_^2^ − F_c_^2^)^2^]/(n − p)]^1/2^ where n is the number of reflections and *p* is the total number of parameters defined.

## Figures and Tables

**Table 1:** Tabe of Auto-Dock Vina Binding Energies, EC_50_ (nM) values, 96h IC_50_ (nM) + 100 nM ATRA values, LXRE Activities (% of Bex), LHS Score (vs. Bex), and RARE Activity (%ATRA at 10 nM).

Compound	Auto-Dock Vina Scores (kcal/mol)	EC_50_ (nM) +/− (SD)	96 h IC_50_ (nM) + 100 nM ATRA +/− SD	LXRE Activity (% of Bex)	LHS Score (vs. Bex)	RARE Activity (%ATRA at 10 nM)
** 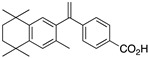 1**	–12.7	17.8 (1.0)	7.8 (1.1)	100	1	37.16
** 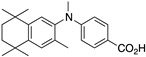 23**	–11.5	8.6 (0.2)	3.7 (1.1)	99.5	0.59	24.33
** 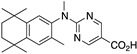 24**	–10.9	6.2 (0.1)	3.9 (1.1)	105.98	0.95	32.04
** 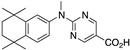 25**	–10.6	17.8 (0.2)	12.9 (1.2)	80.98	0.7	14.32
** 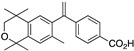 26**	–12.4	51.0 (0.1)	60.0 (1.2)	213.84	2.81	10.1
** 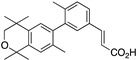 27**	–9.3	65.3 (0.1)	27.6 (1.2)	253.64	1.76	19.45
** 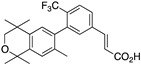 28**	–11.7	59.2 (0.1)	38.4 (1.3)	208.05	1.06	15.85
** 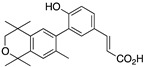 29**	–11.5	3.9 (0.5)	3.7 (1.2)	221.39	1.29	54.64
** 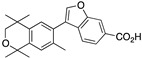 30**	–11.9	>1000	>1000	161.38	4.03	7.05
** 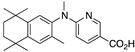 31**	–11.5	5.4 (0.1)	2.1 (1.1)	114.37	1.04	48.61
** 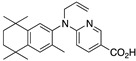 32**	–11.4	1.8 (1.1)	2.0 (1.6)	92.2	0.61	61.79
** 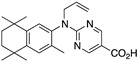 33**	–11.0	5.3 (0.2)	1.3 (1.1)	103.99	0.72	39.94
** 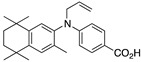 34**	–11.6	11.5 (0.2)	4.1 (1.2)	77.44	0.53	18.74
** 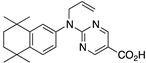 35**	–10.6	0.54 (0.11)	4.68 (1.91)	94.07	0.79	28.76
** 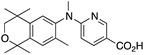 36**	–11.0	75.0 (0.1)	42.3 (1.2)	51.57	0.69	6.25
** 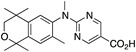 37**	–10.8	404.9 (0.1)	>1000	62.72	1.36	4.02
** 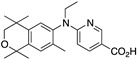 38**	–11.0	23.7 (0.2)	11.6 (1.1)	79.69	0.69	12.59
** 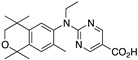 39**	–10.7	151.6 (0.1)	>1000	61.52	0.92	4.96
** 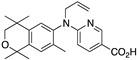 40**	–11.1	10.7 (0.2)	16.6 (1.2)	73.76	0.63	11.13
** 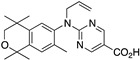 41**	–10.7	143.4 (0.3)	105 (1.6)	75.66	0.71	5.16
** 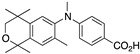 42**	–11.4	87.7 (0.1)	136.7 (1.6)	61.86	0.56	7.99
** 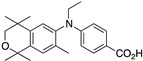 43**	–11.5	23.1 (0.1)	14.8 (1.2)	71.09	0.55	9.6
** 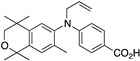 44**	–10.7	116.5 (0.1)	~1000	68.04	0.68	11.13

## Data Availability

CCDC numbers 2191083 and 2191084 contain the supplementary crystallographic data for this paper. These data can be obtained free of charge from The Cambridge Crystallographic Data Centre via www.ccdc.cam.ac.uk/data_request/cif, accessed on 15 December 2022.

## Supplementary Materials

The following are available online at https://www.mdpi.com/article/10.3390/ijms232416213/s1.

## Abbreviations

ATRA	all-*trans*-retinoic acid
AD	Alzheimer’s disease
9-*cis*-RA	9-*cis*-retinoic acid
CTCL	cutaneous T-cell lymphoma
DMF	dimethylformamide
DMSO	dimethylsulfoxide
DNA	deoxyribonucleic acid
FXR	farnesoid-X-receptor
HCl	hydrochloric acid
HRE	hormone responsive element
KOH	potassium hydroxide
LBD	ligand binding domain
LHS	LXR Heterodimer Specificity
LR	lipid risk assessment index
LXR	liver-X-receptor
LXRE	liver-X-receptor element
NaBu	sodium butyrate
NR	nuclear receptor
POC	proof of concept
PPAR	peroxisome proliferator activating receptor
RAR	retinoic-acid-receptor
RARE	retinoic acid receptor element
RXR	retinoid-X-receptor
RXRE	retinoid-X-receptor element
SNuRMs	specific nuclear receptor modulators
SREBP	sterol regulatory element binding protein
TR	thyroid hormone receptor
VDR	vitamin D receptor
