# Supplementary material for: An Isochroman Analog of CD3254 and Allyl-, Isochroman-Analogs of NEt-TMN Prove to Be More Potent Retinoid-X-Receptor (RXR) Selective Agonists Than Bexarotene
Source: Int J Mol Sci. 2022 Dec 19;23(24):16213. doi: 10.3390/ijms232416213 (PMC9782500; doi:10.3390/ijms232416213)
Supplement: Supplementary file 1 [file ijms-23-16213-s001.zip › ijms-2060570-supplementary.pdf]

Supporting Information for **An Isochroman Analog of CD3254 and Allyl-, Isochroman-Analogs of NEt-TMN Prove to be More Potent Retinoid-X-Receptor (RXR) Selective Agonists than Bexarotene**

Synthetic Protocols for compounds made in Schemes 1-10

Representative HPLC Method for compound **36**

HPLC Traces for compounds **23**-**44**

^{1}H-NMR and ^{13}C-NMR for all reported compounds

HPLC. All tested compounds were assessed on a Waters Acquity UPLC with QDA and PDA detectors. Compounds were assayed in ESI-mode on an ACE Excel C18-PFP (1.7 µm, 50 mm x 2.1 mm) column using a 0.1% formic acid/water:acetonitrile gradient over 5 minutes.

NMR and High Resolution Mass Spectrometry. A 400 MHz Bruker Avance III spectrometer was used to acquire ^{1}H NMR and ^{13}C NMR spectra. Chemical shifts (δ) are listed in ppm against residual non-deuterated solvent peaks in a given deuterated solvent (e.g. $CHCl_3$ in $CDCl_3$) as an internal reference. Coupling constants (J) are reported in Hz, and the abbreviations for splitting include: s, single; d, doublet; t, triplet; q, quartet; p, pentet; m, multiplet; br, broad. All ^{13}C NMR spectra were acquired on a Bruker instrument at 100.6 MHz. Chemical shifts (δ) are listed in ppm against deuterated solvent carbon peaks as an internal reference. High resolution mass spectra were recorded using either a JEOL GCmate(2004), a JEOL LCmate(2002) high resolution mass spectrometer or an ABI Mariner (1999) ESI-TOF mass spectrometer. NMR spectra are available in the Supplementary Information document.

General Procedures. Removal of volatile solvents transpired under reduced pressure using a Büchi rotary evaporator and is referred to as removing solvents in vacuo. Thin layer chromatography was conducted on precoated (0.25 mm thickness) silica gel plates with 60F-254 indicator (Merck). Column chromatography was conducted using 230-400 mesh silica gel (E. Merck reagent silica gel 60). The 1,1,4,4,7-pentamethylisochroman (**45**) was purchased from Ark Pharm Inc., compound **59** was purchased from J&W Pharmlab, compound **73** was purchased from Oakwood Chemical, and compound **74** was purchased from Accela. All tested compounds were analyzed for purity by NMR as well as HPLC analysis and were found to be > 95% pure.

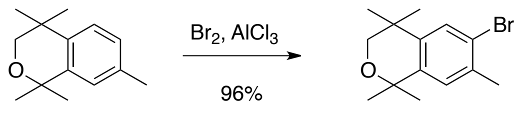

Scheme 1.

6-Bromo-1,1,4,4,7-pentamethylisochroman (46). A modified procedure of Pfahl and co- workers was used.[1] To a 100 mL round bottom flask equipped with a stir bar and charged with 1,1,4,4,7-pentamethylisochroman (**45**) (1.8253 g, 8.934 mmols) dissolved in dichloromethane (6.0 mL) and cooled to 0 °C was added aluminum chloride (0.258 g) followed by bromine (0.50 mL, 9.71 mmols) and the reaction was allowed to warm to room temperature and stirred for 2 hours. Then, 20 mL of a 40% ethyl acetate hexanes solution was added to the reaction and the resulting solution was washed with saturated sodium sulfite (50 mL), the organic layer was separated, and the aqueous layer was extracted with ethyl acetate. The combined organic layers were dried over sodium sulfate, filtered, concentrated in vacuo to give a crude product that was purified by column chromatography (250 mL SiO_2) with 2% ethyl acetate in hexanes to give pure **46** (2.4289 g, 96%) as white solid, m.p. 63.0-65.5 °C: ^{1}H NMR (400 MHz, $CDCl_3$) δ 7.43 (s, 1H), 6.91 (s, 1H), 3.56 (s, 2H), 2.35 (s, 3H), 1.50 (s, 6H), 1.24 (s, 6H); ^{13}C NMR (100.6 MHz, $CDCl_3$) δ 142.2, 140.8, 135.0, 129.0, 127.6, 122.7, 74.8, 70.6, 33.7, 29.6, 26.9, 22.6; GC-MS CI (M+H)+ calcd for $C_{14}H_{19}BrO$ 283.0698, found 283.0701.

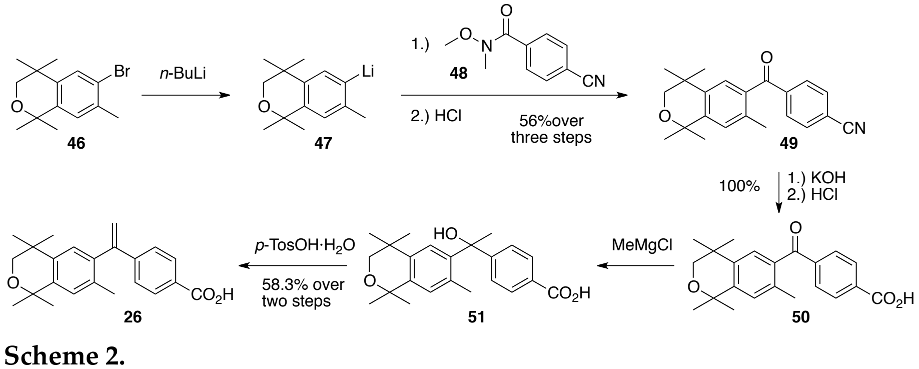

Scheme 2.

4-(1,1,4,4,7-Pentamethylisochroman-6-carbonyl)benzonitrile (49). The method of Faul[2] and co-workers was followed. To a solution of **46** (4.0954 g, 14.46 mmol) in THF (70 mL) at –78 °C under nitrogen was added a 1.6 M solution of n-BuLi in hexanes (10.80 mL, 17.20 mmol) over 10 min and the solution was stirred for 20 min at –78 °C. This reaction solution was transferred via air-tight syringe to a solution of **48**[2] (2.5239 g, 13.27 mmol) in THF (10 mL) at –78 °C, and the combined mixture was stirred for 15 min at –78 °C and then warmed to room temperature before 1.0 N HCl (150 mL) was added to quench the reaction. The solution was poured into ethyl acetate, the layers were separated, and the aqueous layer was extracted with ethyl acetate. The combined organic layers were washed with brine, dried over sodium sulfate, and concentrated in vacuo to give a crude product that was purified by column chromatography (150 mL SiO_2, hexanes:ethyl acetate 95:5) to give **49** (2.7033 g, 56%) as a white crystalline solid, m.p. 136.6-160.0 °C: ^1H NMR (400 MHz, $CDCl_3$) δ 7.89 (d, J = 8.4, 2H), 7.77 (d, J = 8.4, 2H), 7.21 (s, 1H), 6.99 (s, 1H), 3.58 (s, 2H), 2.31 (s, 3H), 1.56 (s, 6H), 1.20 (s, 6H); ^{13}C NMR (100.6 MHz, $CDCl_3$) δ 196.5, 145.1, 141.3, 139.9, 134.9, 134.8, 132.2, 130.3, 128.3, 126.5, 117.9, 116.1, 70.6, 33.5, 29.5, 26.8, 20.0; ES-MS (M+H)+ calcd for $C_{22}H_{23}NO_2Na$ 356.1627, found 356.1639.

4-(1,1,4,4,7-pentamethylisochroman-6-carbonyl)benzoic acid (50). The method of Faul[2] and co-workers was followed. To a heterogeneous solution of **49** (2.2004 g, 6.60 mmol) in 2-methoxyethanol (27 mL) was added a solution of KOH (2.2338. g, 39.8 mmol) in water (10 mL). The reaction was heated in an oil bath at reflux temperature and stirred under nitrogen for 23 h. The reaction was allowed to cool to room temperature before it was quenched with 1 N HCl (70 mL). The solution was poured into ethyl acetate, the layers were separated, and the aqueous layer was extracted with ethyl acetate. The combined organic layers were washed with brine, dried over sodium sulfate, and concentrated in vacuo with additional toluene to azeotrope off 2-methoxyethanol to give **50** (2.32 g, 100%) as a white powder, m.p. 201.4-202.6 °C: ^1H NMR (400 MHz, D_6-DMSO) δ 13.36 (br s, 1H), 8.08 (d, J = 8.4, 2H), 7.78 (d, J = 8.0, 2H), 7.28 (s, 1H), 7.22 (s, 1H), 3.50 (s, 2H), 2.21 (s, 3H), 1.49 (s, 6H), 1.14 (s, 6H); ^{13}C NMR (100.6 MHz, $CDCl_3$) δ 197.0, 166.6, 144.3, 140.6, 139.5, 135.6, 134.6, 133.7, 129.8, 129.6, 128.0, 125.8, 74.6, 69.7, 33.2, 29.3, 26.5, 19.4; ES-MS (M+H)+ calcd for $C_{22}H_{24}O_4$ 353.1753, found 353.1751.

4-(1-(1,1,4,4,7-pentamethylisochroman-6-yl)vinyl)benzoic acid (26). The procedure of Faul and co-workers was followed.[2] To a 100 mL round bottom flask charged with a 3.0 M solution of MeMgCl (4.35 mL, 13.1 mmol) was added THF (8.5 mL), and the solution was cooled to –10 °C in a salt-water ice bath with stirring under nitrogen. To this solution was added a solution of **50** (1.1504 g, 3.26 mmol) in THF (11.3 mL), dropwise, and the reaction was stirred at 0 °C for 4 h. The reaction was quenched with 1.0 N HCl (43 mL), the solution was extracted with ethyl acetate, and the combined organic layers were washed with brine, dried over sodium sulfate, filtered and concentrated in vacuo to give an intermediate alcohol that was used without further purification. The intermediate alcohol was dissolved in toluene (85 mL), and to this solution was added p-toluenesulfonic acid monohydrate (0.07 g, 0.406 mmol), and the solution was refluxed into a Dean-Stark apparatus pre-filled with toluene. After the solution was refluxed for 2 h, it was cooled to room temperature and poured into ethyl acetate and water. The aqueous layer was extracted with ethyl acetate, and the combined organic layers were washed with brine, dried over sodium sulfate, filtered and concentrated in vacuo to give a crude product that was column chromatography (150 mL SiO_2, 30% ethyl acetate:hexanes) to give **26** (0.6672 g, 58.3%) as a white crystalline solid (m.p. 246.7-249.5 °C): ^1H NMR (400 MHz, $CDCl_3$) δ 8.90 (br s, 1H), 8.05 (dd, J = 6.8, 2.0, 2H), 7.37 (dd, J = 6.8, 1.6, 2H), 7.13 (s, 1H), 6.86 (s, 1H), 5.87 (d, J = 1.2, 1H), 5.35 (d, J = 1.2, 1H), 3.62 (s, 2H), 1.96 (s, 3H), 1.57 (s, 6H), 1.27 (s, 6H); ^{13}C NMR (100.6 MHz, $CDCl_3$) δ 171.6, 148.7, 146.0, 141.0, 140.0, 138.7, 133.2, 130.3, 128.2, 126.9, 126.6, 126.5, 117.4, 70.9, 33.5, 29.8, 27.0, 19.9; ES-MS (M-H)- calcd for $C_{23}H_{25}O_3$ 349.1804, found 349.1819.

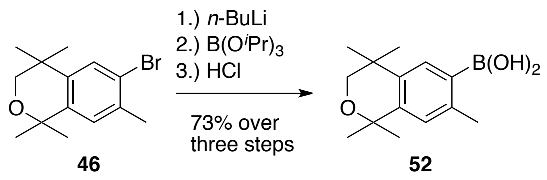

Scheme 3.

(1,1,4,4,7-pentamethylisochroman-6-yl)boronic acid (52). To a 500 mL round bottom flask containing THF (60 mL) was added a 1.6 M solution of n-BuLi in hexanes (16.0 mL, 25.6 mmol), and the resulting solution was cooled in a dry-ice acetone bath to –78 °C with stirring, under nitrogen. To this solution was added a solution of **46** (4.4727 g, 63.32 mmol) in THF (16 mL) over 20 min and the reaction was stirred at –78 °C for 10 min, and a mixture of triisopropylborate (9.8 mL, 42.6 mmol) in THF (10 mL) was added dropwise over 20 min. The reaction was stirred at –78 °C for 1 h and then warmed to room temperature and stirred for 2 h. The reaction was then quenched with 3 N HCl (70 mL), and after stirring for 2 h, it was poured into ethyl acetate, the layers were separated, and the aqueous layer was extracted with ethyl acetate. The combined organic layers were washed with brine, dried over sodium sulfate, and concentrated in vacuo to give a crude product that was purified by column chromatography (150 mL SiO_2, ethyl acetate:hexanes 1:3) to give **52** (5.7572 g, 73%) as a white crystalline solid, m.p. 152.5-168.2 °C: ^1H NMR (400 MHz, $CDCl_3$) δ 8.25 (s, 1H), 6.99 (s, 1H), 3.64 (s, 2H), 2.82 (s, 3H), 1.58 (s, 6H), 1.33 (s, 6H); ^{13}C NMR (100.6 MHz, $CDCl_3$) δ 146.0, 143.2, 139.1, 134.7, 127.3, 127.1, 75.2, 70.8, 33.5, 29.5, 27.0, 26.9, 22.6.

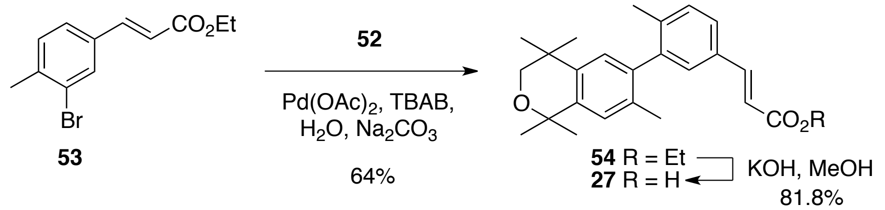

Scheme 4.

(E)-Ethyl 3-(4-methyl-3-(1,1,4,4,7-pentamethylisochroman-6-yl)phenyl)acrylate (54). To a 50 mL Schlenk flask charged with bromide **53**[3] (1.6867 g, 6.2671 mmol), boronic acid **52** (1.5508 g, 6.2501 mmol), TBAB (1.9949 g), Na_2CO_3 (1.9559 g, 18.45 mmol), and water (14.2 mL), was added $Pd(OAc)_2$ (0.0819 g, 0.36 mmol), and the flask was evacuated and back-filled with nitrogen three times. The reaction was stirred at room temperature for 15 min and then placed in an oil bath pre-heated to 150 °C and stirred for 5 min. The reaction was allowed to cool to room temperature, and the black residue was taken up in ethyl acetate and water. The layers were separated, and the aqueous layer was extracted with ethyl acetate. The combined organic layers were washed with brine, dried over sodium sulfate, filtered, and concentrated in vacuo to give a crude product that was purified by column chromatography (150 mL SiO_2, 2% - 4% ethyl acetate:hexanes) to give **54** (1.5781 g, 64.3%) as a white solid, m.p. 133.7-136.9 °C: ^1H NMR (400 MHz, $CDCl_3$) δ 7.68 (d, J = 16.0, 1H), 7.41 (dd, J = 8.0, 2.0, 1H), 7.32 (d, J = 2.0, 1H), 7.28 (d, J = 8.0, 1H), 7.00 (s, 1H), 6.93 (s, 1H), 6.41, (d, J = 16.0, 1H), 4.25 (q, J = 7.2, 2H), 3.61 (s, 2H), 2.08 (s, 3H), 2.01 (s, 3H), 1.57 (s, 6H), 1.32 (t, J = 7.2, 3H), 1.25 (s, 3H), 1.23 (s, 3H); ^{13}C NMR (100.6 MHz, $CDCl_3$) δ 167.1, 144.4, 142.1, 140.5, 139.8, 138.8, 138.6, 132.8, 131.9, 130.4, 129.1, 126.7, 126.5, 125.9, 117.4, 75.0, 70.9, 60.3, 33.6, 29.9, 29.8, 27.1, 27.0, 20.0, 19.5, 14.3; ES-MS (M+Na)+ calcd for $C_{26}H_{32}O_3Na$ 415.2249, found 415.2248.

(E)-3-(4-methyl-3-(1,1,4,4,7-pentamethylisochroman-6-yl)phenyl)acrylic acid (27). To a 100 mL round bottom flask containing **54** (1.3285 g, 3.3844 mmol) suspended in methanol (9.0 mL) was added a solution of KOH (0.5744 g, 10.23 mmol) in water (0.48 mL), and the solution was refluxed in an oil-bath pre-heated to 85 °C for 1 h. The reaction was allowed to cool to room temperature, and acidified with an aqueous 20% HCl solution (80 mL). The resulting precipitate was filtered and washed with copious amounts of water, and the crude white powder was purified by column chromatography (25 mL SiO_2, ethyl acetate:hexanes 1:3) to give **27** (1.0092 g, 81.8%) as a white crystalline solid, m.p. 219.3-224.3 °C: ^1H NMR

(400 MHz, $CDCl_3$) δ 8.78 (br s, 1H), 7.78 (d, J = 16.0, 1H), 7.45 (dd, J = 8.0, 1.6, 1H), 7.34 (d, J = 1.6, 1H), 7.30 (d, J = 8.0, 1H), 7.00 (s, 1H), 6.94 (s, 1H), 6.42, (d, J = 16.0, 1H), 3.62 (s, 2H), 2.10 (s, 3H), 2.02 (s, 3H), 1.58 (s, 6H), 1.26 (s, 3H), 1.23 (s, 3H); ^{13}C NMR (100.6 MHz, $CDCl_3$) δ 172.3, 146.9, 142.2, 140.6, 139.8, 139.5, 138.4, 132.8, 131.5, 130.5, 129.4, 127.1, 126.5, 125.8, 116.4, 75.1, 70.8, 33.6, 29.9, 29.8, 27.1, 26.9, 20.1, 19.5; LC-MS (M-H)- calcd for $C_{25}H_{27}O_3$ 363.1960, found 363.1953.

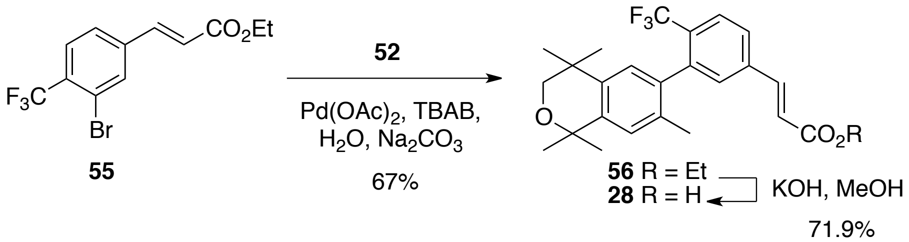

Scheme 5.

(E)-Ethyl 3-(3-(1,1,4,4,7-pentamethylisochroman-6-yl)-4-(trifluoromethyl)phenyl)acrylate (56). To a 50 mL Schlenk flask charged with bromide **55**[3] (2.0182 g, 6.246 mmol), boronic acid **52** (1.5545 g, 6.265 mmol), TBAB (2.046 g), Na_2CO_3 (1.9568 g, 18.46 mmol), and water (14.2 mL), was added $Pd(OAc)_2$ (0.0817 g, 0.36 mmol), and the flask was evacuated and back-filled with nitrogen three times. The reaction was stirred at room temperature for 15 min and then placed in an oil bath pre-heated to 150 °C and stirred for 5 min. The reaction was allowed to cool to room temperature, and the black residue was taken up in ethyl acetate and water. The layers were separated, and the aqueous layer was extracted with ethyl acetate. The combined organic layers were washed with brine, dried over sodium sulfate, filtered, and concentrated in vacuo to give a crude product that was purified by column chromatography (150 mL SiO_2, 2% - 4% ethyl acetate:hexanes) to give **56** (1.8746 g, 67.2%) as a white solid, m.p. 147.9-149.7 °C: ^1H NMR (400 MHz, $CDCl_3$) δ 7.76 (d, J = 8.4, 1H), 7.68 (d, J = 16.0, 1H), 7.58 (d, J = 8.0, 1H), 7.43 (s, 1H), 7.02 (s, 1H), 6.91 (s, 1H), 6.51 (d, J = 16.0, 1H), 4.26 (q, J = 7.2, 2H), 3.60 (s, 2H), 1.99 (s, 3H), 1.56 (s, 6H), 1.32 (t, J = 7.2, 3H), 1.23 (s, 3H), 1.20 (s, 3H); ^{13}C NMR (100.6 MHz, $CDCl_3$) δ 166.3, 142.5, 141.5, 141.4, 141.2, 139.0, 137.2, 135.9, 132.8, 131.0, 130.1, 129.8, 126.7, 126.6, 126.5, 126.3, 126.2, 125.0, 122.2, 121.0, 75.0, 70.8, 60.7, 33.5, 30.0, 29.6, 27.0, 26.6, 19.8, 14.2; ES-MS (M+Na)+ calcd for $C_{26}H_{29}F_3O_3Na$ 469.1967, found 469.1966.

(E)-3-(3-(1,1,4,4,7-pentamethylisochroman-6-yl)-4-(trifluoromethyl)phenyl)acrylic acid (28). To a 100 mL round bottom flask containing **56** (1.5789 g, 3.536 mmol) suspended in methanol (7.0 mL) was added a solution of KOH (0.6462 g, 11.52 mmol) in water (0.48 mL), and the solution was refluxed in an oil-bath pre-heated to 85 °C for 1 h. The reaction was allowed to cool to room temperature, and acidified with an aqueous 1N HCl solution (80 mL). The resulting precipitate was filtered and washed with copious amounts of water, and the crude white powder was purified by column chromatography (150 mL SiO_2, ethyl acetate:hexanes 1:3) to give **28** (1.0647 g, 72%) as a white crystalline solid, m.p. 225.3-232.6 °C: ^1H NMR (400 MHz, $CDCl_3$) δ 7.79 (d, J = 16.0, 1H), 7.78 (d, J = 8.4, 1H), 7.62 (d, J = 8.4, 1H), 7.46 (s, 1H), 7.03 (s, 1H), 6.92 (s, 1H), 6.52, (d, J = 16.4, 1H), 3.62 (s, 2H), 2.00 (s, 3H), 1.58 (s, 6H), 1.24 (s, 3H), 1.21 (s, 3H); ^{13}C NMR (100.6 MHz, $CDCl_3$) δ 171.3, 144.9, 141.6, 141.5, 141.2, 139.0, 136.7, 135.8, 132.8, 131.2, 130.6, 130.3, 126.9, 126.8, 126.7, 126.4, 126.3, 126.2, 124.9, 122.2, 120.0, 75.1, 70.8, 33.5, 29.9, 29.6, 27.0, 26.6, 19.8; ES-MS (M-H)- calcd for $C_{24}H_{24}F_3O_3$ 417.1678, found 417.1697.

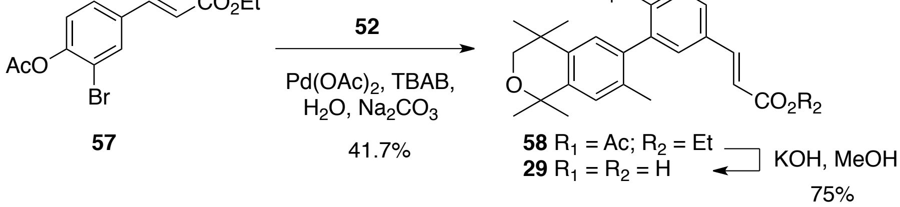

Scheme 6.

(E)-Ethyl 3-(4-acetoxy-3-(1,1,4,4,7-pentamethylisochroman-6-yl)phenyl)acrylate (58). To a 50 mL Schlenk flask charged with bromide **57**[3] (1.9557 g, 6.2454 mmol), boronic acid **52** (1.5712 g, 6.332 mmol), TBAB (1.9968 g), Na_2CO_3 (1.9556 g, 18.44 mmol), and water (14.20 mL), was added $Pd(OAc)_2$ (0.0819 g, 0.3637 mmol), and the flask was evacuated and back-filled with nitrogen three times. The reaction was stirred at room temperature for 15 min and then placed in an oil bath pre-heated to 150 °C and stirred for 5 min. The reaction was allowed to cool to room temperature, and the black residue was taken up in ethyl acetate and water. The layers were separated, and the aqueous layer was extracted with ethyl acetate. The combined organic layers were washed with brine, dried over sodium sulfate, filtered, and concentrated in vacuo to give a crude product that was purified by column chromatography (150 mL SiO_2, 4% - 8% ethyl acetate:hexanes) to give **58** (1.1370 g, 41.7%) as a white solid, m.p. 132.7-136.4 °C: ^1H NMR (400 MHz, $CDCl_3$) δ 7.68 (d, J = 16.0, 1H), 7.54 (dd, J = 8.4, 2.0, 1H), 7.46 (d, J = 2.0, 1H), 7.16 (d, J = 8.4, 1H), 7.04 (s, 1H), 6.93 (s, 1H), 6.40, (d, J = 16.0, 1H), 4.25 (q, J = 7.2, 2H), 3.59 (m, 2H), 2.11 (s, 3H), 1.92 (s, 3H), 1.57 (s, 6H), 1.32 (t, J = 7.2, 3H), 1.23 (s, 6H); ^{13}C NMR (100.6 MHz, $CDCl_3$) δ 169.0, 166.8, 149.8, 143.4, 141.2, 139.5, 135.3, 134.0, 133.3, 132.3, 130.9, 127.9, 126.7, 126.6, 123.1, 118.6, 75.0, 70.8, 60.5, 33.5, 20.4, 19.6, 14.2; ES-MS (M+H)+ calcd for $C_{27}H_{33}O_5$ 437.2328, found 437.2324.

(E)-3-94-hydroxy-3-(1,1,4,4,7-pentamethylisochroman-6-yl)phenyl)acrylic acid (29). To a 100 mL round bottom flask containing **58** (1.0352 g, 2.3714 mmol) suspended in methanol (8.0 mL) was added a solution of KOH (0.9439 g, 16.82 mmol) in water (0.70 mL), and the solution was refluxed in an oil-bath pre-heated to 85 °C for 1.5 h. The reaction was allowed to cool to room temperature, and acidified with an aqueous 1N HCl solution (80 mL). The resulting precipitate was filtered and washed with copious amounts of water, and the crude white powder was purified by column chromatography (25 mL SiO_2, 35% ethyl acetate:hexanes) to give **29** (0.6541 g, 75.2%) as a white crystalline solid, m.p. 175.2-178.0 °C: ^1H NMR (400 MHz, $CDCl_3$) δ 7.75 (d, J = 15.6, 1H), 7.50 (dd, J = 8.4, 2.0, 1H), 7.35 (d, J = 2.0, 1H), 7.14 (s, 1H), 7.02 (s, 1H), 7.01 (d, J = 8.4, 1H), 6.32, (d, J = 15.6, 1H), 3.62 (s, 2H), 2.13 (s, 3H), 1.58 (s, 6H), 1.26 (s, 6H); ^{13}C NMR (100.6 MHz, $CDCl_3$) δ 172.4, 155.1, 146.6, 142.3, 141.0, 134.4, 132.6, 130.8, 129.6, 128.3, 127.6, 127.1, 126.7, 115.9, 114.8, 75.1, 70.7, 33.7, 29.7, 27.0, 19.4; ES-MS (M+Na)+ calcd for $C_{23}H_{26}O_4Na$ 389.1729, found 389.1737.

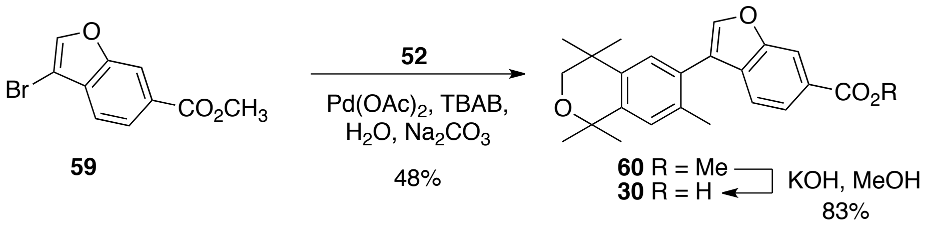

Scheme 7.

Methyl 3-(1,1,4,4,7-pentamethylisochroman-6-yl)benzofuran-6-carboxylate (60). To a 50 mL Schlenk flask charged with bromide **59** (0.9063 g, 3.553 mmol), boronic acid **52** (0.8888 g, 3.582 mmol), TBAB (1.1366 g), Na_2CO_3 (1.1176 g, 10.54 mmol), and water (8.05 mL), was added $Pd(OAc)_2$ (0.0507 g, 0.225 mmol), and the flask was evacuated and back-filled with nitrogen three times. The reaction was stirred at room temperature for 15 min and then placed in an oil bath pre-heated to 150 °C and stirred for 5 min. The reaction was allowed to cool to room temperature, and the black residue was taken up in ethyl acetate and water. The layers were separated, and the aqueous layer was extracted with ethyl acetate. The combined organic layers were washed with brine, dried over sodium sulfate, filtered, and concentrated in vacuo to give a crude product that was purified by column chromatography (150 mL SiO_2, 2% - 4% ethyl acetate:hexane) to give **60** (0.6486 g, 48.2%) as a white solid, m.p. 129.8 – 133.0 °C: ^1H NMR (400 MHz, $CDCl_3$) δ 8.21 (s, 1H), 8.07 (dd, J = 8.8, 1.6, 1H), 7.68 (s, 1H), 7.57 (dd, J = 8.8, 0.4, 1H), 7.30 (s, 1H), 7.03 (s, 1H), 3.91 (s, 3H), 3.64 (s, 2H), 2.26 (s, 3H), 1.60 (s, 6H), 1.29 (s, 6H); ^{13}C NMR (100.6 MHz, $CDCl_3$) δ 167.2, 157.6, 143.2, 141.5, 140.2, 134.1, 128.1, 127.3, 127.2, 126.1, 125.2, 123.2, 121.9, 111.5, 75.0, 70.8, 52.1, 33.6, 29.7, 27.0, 20.4; ES-MS (M+Na)+ calcd for $C_{24}H_{26}O_4Na$ 401.1729, found 401.1740.

3-(1,1,4,4,7-pentamethylisochroman-6-yl)benzofuran-6-carboxylic acid (30). To a 100 mL round bottom flask containing **60** (0.5813 g, 1.5359 mmol) suspended in methanol (4.4 mL) was added a solution of KOH (0.2749 g, 5.08 mmol) in water (0.28 mL), and the solution was refluxed in an oil-bath pre-heated to 85 °C for 1.5 h. The reaction was allowed to cool to room temperature, and acidified with an aqueous 1N HCl solution (80 mL). The resulting precipitate was filtered and washed with copious amounts of water, and the crude white powder was purified by column chromatography (25 mL SiO_2, 25% ethyl acetate:hexanes) to give **30** (0.4670 g, 83.4%) as a white crystalline solid, m.p. 251.8-254.8 °C: ^{1}H NMR (400 MHz, $CDCl_3$) δ 9.49 (br s, 1H), 8.30 (d, J = 1.6, 1H), 8.15 (dd, J = 8.4, 1.6, 1H), 7.71 (s, 1H), 7.61 (d, J = 8.8, 1H), 7.31 (s, 1H), 7.04 (s, 1H), 3.65 (s, 2H), 2.28 (s, 3H), 1.61 (s, 6H), 1.30 (s, 6H); ^{13}C NMR (100.6 MHz, $CDCl_3$) δ 172.1, 158.1, 143.4, 141.5, 140.2, 134.0, 128.1, 128.0, 127.3, 127.1, 126.7, 124.3, 124.1, 121.9, 111.7, 75.1, 70.8, 33.6, 29.7, 27.0, 20.2; ES-MS (M+Na)+ calcd for $C_{23}H_{24}O_4Na$ 387.1572, found 387.1566.

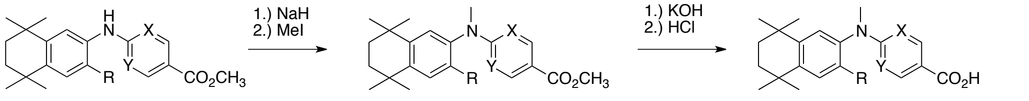

61 R = CH_3; X = Y = CH
62 R = CH_3; X = Y = N
63 R = H; X = Y = N
64 R = CH_3; X = N; Y = CH

65 R = CH_3; X = Y = CH (83%)
66 R = CH_3; X = Y = N (83%)
67 R = H; X = Y = N (72%)
68 R = CH_3; X = N; Y = CH (74%)

23 R = CH_3; X = Y = CH (53%)
24 R = CH_3; X = Y = N (78%)
25 R = H; X = Y = N (81%)
31 R = CH_3; X = N; Y = CH (60%)

Scheme 8.

Methyl 4-(methyl(3,5,5,8,8,-pentamethyl-5,6,7,8-tetrahydronaphthalen-2-yl)amino)benzoate (65). To a flame-dried, 100 mL round-bottomed flask equipped with a magnetic stir bar was added a 60% dispersion of sodium hydride in mineral oil (0.3682 g, 9.206 mmol). The dispersion of sodium hydride was washed with hexanes (3.0 mL, twice) and dried under vacuum and suspended in 4.8 mL of DMF under nitrogen. To this solution of sodium hydride in DMF was added a solution of **61**[4] (1.3285 g, 3.7796 mmol) in DMF (14.0 mL), and the reaction was stirred for 15 min., and then methyl iodide (0.43 mL, 6.91 mmol) was added, and the reaction was stirred for 1 h. The reaction was poured into water and extracted with ethyl acetate. The combined organic layers were washed with brine, dried over sodium sulfate, filtered, and concentrated in vacuo to yield a crude product that was purified by column chromatography (150 mL SiO_2, 6% ethyl acetate:hexanes) to give **65** (1.1502 g, 83.3%) as a white crystalline solid, m.p. 128.5 - 160.0 °C: ^{1}H NMR (400 MHz, $CDCl_3$) δ 7.84 (d, J = 8.8, 2H), 7.18 (s, 1H), 7.03 (s, 1H), 6.46 (d, J = 8.8, 1H), 3.84 (s, 3H), 3.26 (s, 3H), 2.03 (s, 3H), 1.68 (s, 4H), 1.30 (s, 6H), 1.22 (s, 6H); ^{13}C NMR (100.6 MHz, $CDCl_3$) δ 167.4, 152.5, 144.5, 143.8, 142.6, 132.7, 131.1, 129.3, 125.8, 117.2, 111.1, 51.4, 39.1, 35.0, 34.9, 34.1, 33.9, 31.8, 17.3; ES-MS (M+H)+ calcd for $C_{24}H_{31}NO_2$ 366.2433, found 366.2418.

4-(Methyl(3,5,5,8,8-pentamethyl-5,6,7,8-tetrahydronaphthalen-2-yl)amino)benzoic acid (23). To a 100 mL round-bottomed flask equipped with a stir bar and charged with methyl ester **65** (1.0449 g, 2.8588 mmol) suspended in methanol (13.0 mL) was added a solution of potassium hydroxide (0.5551 g, 9.89 mmol) in water (0.64 mL). This reaction was stirred at reflux in an oil bath at 87 °C for 1.5h. The reaction was then cooled to r.t. and acidified with 1N HCl (90 mL). The resulting precipitate was filtered and washed with copious amounts of cold water to give a crude product that was purified by column chromatography (25 mL SiO_2, 15% ethyl acetate:hexanes) to give pure **23** (0.5403 g, 53.8%) as a white crystalline solid, m.p. 242.6 – 247.7 °C: ^{1}H NMR (400 MHz, d6-DMSO) δ 12.14 (br s, 1H), 7.70 (d, J = 9.2, 2H), 7.27 (s, 1H), 7.07 (s, 1H), 6.42 (d, J = 8.4, 1H), 3.20 (s, 3H), 1.97 (s, 3H), 1.63 (s, 4H), 1.25 (s, 6H), 1.19 (s, 6H); ^{13}C NMR (100.6 MHz, d6-DMSO) δ 167.4, 152.0, 144.1, 143.2, 132.4, 131.0, 129.1, 125.5, 117.6, 111.0, 59.7, 34.6, 34.5, 33.8, 33.7, 31.7, 31.6, 17.0; ES-MS (M+Na)+ calcd for $C_{23}H_{29}NO_2Na$ 374.2096, found 374.2097.

Methyl 2-(methyl(3,5,5,8,8-pentamethyl-5,6,7,8-tetrahydronaphthalen-2-yl)amino)pyrimidine-5-carboxylate (66). To a flame-dried, 100 mL round-bottomed flask equipped with a magnetic stir bar was added a 60% dispersion of sodium hydride in mineral oil (0.4425 g, 11.06 mmol). The dispersion of sodium hydride was washed with hexanes (3.7 mL, twice) and dried under vacuum and suspended in 5.8 mL of DMF under nitrogen. To this solution of sodium hydride in DMF was added a solution of **62**[4] (1.6074 g, 4.548 mmol) in DMF (16.9 mL), and the reaction was stirred for 15 min., and then methyl iodide

(0.43 mL, 6.91 mmol) was added, and the reaction was stirred for 1 h. The reaction was poured into water and extracted with ethyl acetate. The combined organic layers were washed with brine, dried over sodium sulfate, filtered, and concentrated in vacuo to yield a crude product that was purified by column chromatography (150 mL SiO_2, 6% ethyl acetate:hexanes) to give **66** (1.4023 g, 83.9%) as a white crystalline solid, m.p. 219.6 - 221.8 °C: ^1H NMR (400 MHz, $CDCl_3$) δ 8.96 (d, J = 2.8, 1H), 8.81 (d, J = 2.8, 1H), 7.19 (s, 1H), 7.04 (s, 1H), 3.86 (s, 3H), 3.50 (s, 3H), 2.05 (s, 3H), 1.68 (s, 4H), 1.31 (s, 3H), 1.27 (s, 3H), 1.25 (s, 3H), 1.24 (s, 3H); ^{13}C NMR (100.6 MHz, $CDCl_3$) δ 165.1, 161.9, 160.2, 159.1, 144.2, 144.1, 140.4, 131.5, 129.1, 124.7, 112.7, 51.8, 39.0, 35.0, 34.9, 34.1, 34.0, 32.0, 31.9, 31.8, 31.6, 17.4; GC-MS CI (M)+ calcd for $C_{22}H_{29}N_3O_2$ 367.2260, found 367.2251.

2-(Methyl(3,5,5,8,8-pentamethyl-5,6,7,8-tetrahydronaphthalen-2-yl)amino)pyrimidine-5-carboxylic acid (24). To a 100 mL round-bottomed flask equipped with a stir bar and charged with methyl ester **66** (0.9053 g, 2.464 mmol) suspended in methanol (9.0 mL) was added a solution of potassium hydroxide (0.4726 g, 8.423 mmol) in water (0.54 mL). This reaction was stirred at reflux in an oil bath at 87 °C for 1.5h. The reaction was then cooled to r.t. and acidified with 1N HCl (90 mL). The resulting precipitate was filtered and washed with copious amounts of cold water to give a crude product that was purified by column chromatography (25 mL SiO_2, 60% ethyl acetate:hexanes to 1% methanol:ethyl acetate) to give pure **24** (0.6819 g, 78.3%) as a white crystalline solid, m.p. 323.0 – 326.9 °C: ^1H NMR (400 MHz, d6-DMSO) δ 12.87 (br s, 1H), 8.87 (d, J = 2.8, 1H), 8.65 (d, J = 2.8, 1H), 7.22 (s, 1H), 7.15 (s, 1H), 3.40 (s, 3H), 1.95 (s, 3H), 1.62 (s, 4H), 1.26 (s, 3H), 1.24 (s, 3H), 1.21 (s, 3H), 1.18 (s, 3H); ^{13}C NMR (100.6 MHz, d6-DMSO) δ 165.6, 162.2, 159.7, 143.5, 143.1, 141.0, 131.8, 128.5, 124.7, 113.2, 38.4, 34.7, 34.6, 33.8, 33.7, 31.6, 31.5, 17.1; ES-MS (M+H)+ calcd for $C_{21}H_{28}N_3O_2$ 354.2181, found 354.2184.

Methyl 2-(methyl(5,5,8,8-tetramethyl-5,6,7,8-tetrahydronapthalen-2-yl)amino)pyrimidine-5-carboxylate (67). To a flame-dried, 100 mL round-bottomed flask equipped with a magnetic stir bar was added a 60% dispersion of sodium hydride in mineral oil (0.4435 g, 11.09 mmol). The dispersion of sodium hydride was washed with hexanes (3.0 mL, twice) and dried under vacuum and suspended in 5.8 mL of DMF under nitrogen. To this solution of sodium hydride in DMF was added a solution of **63**[4] (1.4193 g, 4.1814 mmol) in DMF (16.4 mL), and the reaction was stirred for 15 min., and then methyl iodide (0.42 mL, 6.74 mmol) was added, and the reaction was stirred for 1 h. The reaction was poured into water and extracted with ethyl acetate. The combined organic layers were washed with brine, dried over sodium sulfate, filtered, and concentrated in vacuo to yield a crude product that was purified by column chromatography (150 mL SiO_2, 5% to 10% ethyl acetate:hexanes) to give **67** (1.0728 g, 72.6%) as a white crystalline solid, m.p. 211.8 - 212.9 °C: ^1H NMR (400 MHz, $CDCl_3$) δ 8.88 (s, 2H), 7.34 (d, J = 8.4, 1H), 7.19 (d, J = 2.4, 1H), 7.05 (dd, J = 8.4, 2.4, 1H), 3.87 (s, 3H), 3.58 (s, 3H), 1.69 (s, 4H), 1.29 (s, 6H), 1.27 (s, 6H); ^{13}C NMR (100.6 MHz, $CDCl_3$) δ 165.1, 162.2, 159.5, 145.9, 143.1, 141.5, 127.3, 124.0, 123.2, 113.1, 51.8, 39.5, 34.9, 34.9, 34.4, 34.1, 31.7; ES-MS (M+H)+ calcd for $C_{21}H_{28}N_3O_2$ 354.2181, found 354.2181.

2-(Methyl(5,5,8,8-tetramethyl-5,6,7,8-tetrahydronaphthalen-2-yl)amino)pyrimidine-5-carboxylic acid (25). To a 100 mL round-bottomed flask equipped with a stir bar and charged with methyl ester **67** (0.9075 g, 2.568 mmol) suspended in methanol (8.0 mL) was added a solution of potassium hydroxide (0.4839 g, 8.62 mmol) in water (0.48 mL). This reaction was stirred at reflux in an oil bath at 87 °C for 1.5h. The reaction was then cooled to r.t. and acidified with 1N HCl (80 mL). The resulting precipitate was filtered and washed with copious amounts of cold water to give a crude product that was purified by column chromatography (25 mL SiO_2, 60% ethyl acetate:hexanes to pure ethyl acetate to 1% methanol:ethyl acetate) to give pure **25** (0.7109 g, 81.6%) as a white crystalline solid, m.p. 313.5 – 316.4 °C: ^1H NMR (400 MHz, d6-DMSO) δ 12.86 (br s, 1H), 8.75 (s, 2H), 7.33 (d, J = 8.4, 2H), 7.26 (d, J = 2.0, 1H), 7.06 (dd, J = 8.4, 2.4, 1H), 3.48 (s, 3H), 1.64 (s, 4H), 1.25 (s, 6H), 1.23 (s, 6H); ^{13}C NMR (100.6 MHz, d6-DMSO) δ 165.5, 162.3, 159.4, 145.3, 142.2, 141.9, 126.9, 124.1, 124.0, 113.7, 34.5, 34.1, 33.8, 31.6, 31.5; ES-MS (M+H)+ calcd for $C_{20}H_{26}N_3O_2$ 340.2025, found 340.2025.

Methyl 6-(methyl(3,5,5,8,8-pentamethyl-5,6,7,8-tetrahydronaphthalen-2-yl)amino)nicotinate (68). To a flame-dried, 100 mL round-bottomed flask equipped with a magnetic stir bar was added a 60%

dispersion of sodium hydride in mineral oil (0.4232 g, 10.58 mmol). The dispersion of sodium hydride was washed with hexanes (3.7 mL, twice) and dried under vacuum and suspended in 5.8 mL of DMF under nitrogen. To this solution of sodium hydride in DMF was added a solution of **64**[4] (1.6059 g, 4.556 mmol) in DMF (17.3 mL), and the reaction was stirred for 15 min., and then methyl iodide (0.43 mL, 6.91 mmol) was added, and the reaction was stirred for 1 h. The reaction was poured into water and extracted with ethyl acetate. The combined organic layers were washed with brine, dried over sodium sulfate, filtered, and concentrated in vacuo to yield a crude product that was purified by column chromatography (150 mL SiO_2, 5% - 10% ethyl acetate:hexanes) to give **68** (1.2434 g, 74.5%) as a white crystalline solid, m.p. 161.7-163.6 °C: ^{1}H NMR (400 MHz, $CDCl_3$) δ 8.86 (dd, J = 2.0, 0.4, 1H), 7.82 (dd, J = 9.2, 2.0, 1H), 7.21 (s, 1H), 7.05 (s, 1H), 6.03 (d, J = 9.2, 1H), 3.85 (s, 3H), 3.46 (s, 3H), 2.05 (s, 3H), 1.68 (s, 4H), 1.30 (s, 6H), 1.23 (s, 6H); ^{13}C NMR (100.6 MHz, $CDCl_3$) δ 166.4, 160.1, 150.4, 145.0, 144.7, 140.6, 137.7, 132.3, 129.5, 114.2, 107.2, 51.6, 38.0, 35.0, 34.9, 34.1, 34.0, 31.8, 17.1; ES-MS (M+H)+ calcd for $C_{23}H_{31}N_2O_2$ 367.2386, found 367.2390.

6-(Methyl(3,5,5,8,8-pentamethyl-5,6,7,8-tetrahydronaphthalen-2-yl)amino)nicotinic acid (31). To a 100 mL round-bottomed flask equipped with a stir bar and charged with methyl ester **68** (0.8203 g, 2.238 mmol) suspended in methanol (8.0 mL) was added a solution of potassium hydroxide (0.4284 g, 7.635 mmol) in water (0.42 mL). This reaction was stirred at reflux in an oil bath at 87 °C for 1.5h. The reaction was then cooled to r.t. and acidified with 1N HCl (80 mL). The solution was extracted with ethyl acetate (2 X 70 mL), and the organic layers were dried over sodium sulfate and concentrated in vacuo to give a crude product that was purified by column chromatography (25 mL SiO_2, 30% ethyl acetate:hexanes to 60% ethyl acetate:hexanes) to give pure **31** (0.4790 g, 60.7%) as a white crystalline solid, m.p. 273.5-274.8 °C: ^{1}H NMR (400 MHz, $CDCl_3$) δ 10.37 (br s, 1H), 8.97 (d, J = 2.0, 1H), 7.91 (d, J = 7.6, 1H), 7.22 (s, 1H), 7.06 (s, 1H), 6.10 (d, J = 8.4, 1H), 3.53 (s, 3H), 2.07 (s, 3H), 1.69 (s, 4H), 1.31 (s, 3H), 1.29 (s, 3H), 1.24 (s, 3H), 1.22 (s, 3H); ^{13}C NMR (100.6 MHz, $CDCl_3$) δ 169.6, 161.9, 145.3, 139.9, 139.1, 132.1, 131.9, 129.7, 125.3, 114.1, 112.9, 108.3, 38.9, 34.9, 34.8, 34.2, 34.1, 31.9, 31.8, 17.1; ES-MS (M+H)+ calcd for $C_{22}H_{29}N_2O_2$ 353.2229, found 353.2224.

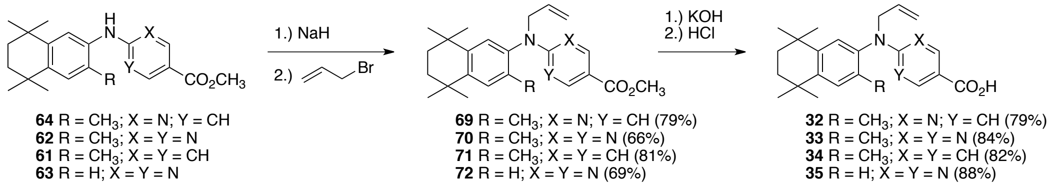

Scheme 9.

Methyl 6-(allyl(3,5,5,8,8-pentamethyl-5,6,7,8-tetrahydronaphthalen-2-yl)amino)nicotinate (69). To a flame-dried, 100 mL round-bottomed flask equipped with a magnetic stir bar was added a 60% dispersion of sodium hydride in mineral oil (0.4441 g, 11.10 mmol). The dispersion of sodium hydride was washed with hexanes (3.0 mL, twice) and dried under vacuum and suspended in 5.8 mL of DMF under nitrogen. To this solution of sodium hydride in DMF was added a solution of **64**[4] (1.6111 g, 4.571 mmol) in DMF (16.9 mL), and the reaction was stirred for 15 min., and then allyl bromide (0.67 mL, 7.74 mmol) was added, and the reaction was stirred for 1 h. The reaction was poured into water and extracted with ethyl acetate. The combined organic layers were washed with brine, dried over sodium sulfate, filtered, and concentrated in vacuo to yield a crude product that was purified by column chromatography (150 mL SiO_2, 5% ethyl acetate:hexanes) to give **69** (1.4175 g, 79%) as a white crystalline solid, m.p. 88.6 - 90.0 °C: ^{1}H NMR (400 MHz, $CDCl_3$) δ 8.86 (d, J = 1.6, 1H), 7.85 (dd, J = 8.8, 1.6, 1H), 7.20 (s, 1H), 7.01 (s, 1H), 6.08-5.98 (m, 2H), 5.21-5.15 (m, 2H), 4.98-4.96 (m, 1H), 4.12 (m, 1H), 3.85 (s, 3H), 2.03 (s, 3H), 1.67 (s, 4H), 1.29 (s, 3H), 1.28 (s, 3H), 1.20 (s, 6H); ^{13}C NMR (100.6 MHz, $CDCl_3$) δ 166.0, 158.9, 149.8, 144.9, 144.6, 138.8, 138.4, 133.0, 132.4, 129.4, 126.7, 117.9, 114.6, 107.6, 53.0, 51.7, 34.9, 34.8, 34.1, 34.0, 31.7, 17.3; ES-MS (M+H)+ calcd for $C_{25}H_{33}N_2O_2$ 393.2542, found 393.2545.

6-(Allyl(3,5,5,8,8-pentamethyl-5,6,7,8-tetrahydronaphthalen-2-yl)amino)nicotinic acid (32). To a 100 mL round-bottomed flask equipped with a stir bar and charged with methyl ester **69** (1.2842 g, 3.272 mmol)

suspended in methanol (8.0 mL) was added a solution of potassium hydroxide (0.6365 g, 11.34 mmol) in water (0.71 mL). This reaction was stirred at reflux in an oil bath at 87 °C for 1.5h. The reaction was then cooled to r.t. and acidified with 1N HCl (80 mL). The resulting precipitate was filtered and washed with copious amounts of cold water to give a crude product that was purified by column chromatography (25 mL SiO_2, 30% ethyl acetate:hexanes) to give pure **32** (0.9875 g, 79.7%) as a white crystalline solid, m.p. 203.4 – 204.4 °C: ^{1}H NMR (400 MHz, d6-DMSO) δ 12.50 (br s, 1H), 8.67 (d, J = 2.0, 1H), 7.82 (dd, J = 8.8, 2.4, 2H), 7.30 (s, 1H), 7.08 (s, 1H), 6.02-5.92 (m, 2H), 5.14-5.09 (m, 2H), 4.80-4.77 (m, 1H), 4.12-4.10 (m, 1H), 1.98 (s, 3H), 1.60 (s, 4H), 1.22 (s, 6H), 1.12 (s, 6H); ^{13}C NMR (100.6 MHz, d6-DMSO) δ 166.6, 159.5, 150.6, 143.9,139.2, 138.1, 134.1, 132.5, 129.2, 126.5, 117.3, 114.9, 106.1, 52.1, 34.6, 34.4, 33.8, 33.7, 31.5, 17.1; ES-MS (M+Na)+ calcd for $C_{24}H_{30}N_2O_2Na$ 401.2205, found 401.2202.

Methyl 2-(allyl(3,5,5,8,8-pentamethyl-5,6,7,8-tetrahydronaphthalen-2-yl)amino)pyrimidine-5-carboxylate (70). To a flame-dried, 100 mL round-bottomed flask equipped with a magnetic stir bar was added a 60% dispersion of sodium hydride in mineral oil (0.4412 g, 11.03 mmol). The dispersion of sodium hydride was washed with hexanes (3.0 mL, twice) and dried under vacuum and suspended in 5.8 mL of DMF under nitrogen. To this solution of sodium hydride in DMF was added a solution of **62**[4] (1.6218 g, 4.588 mmol) in DMF (16.9 mL), and the reaction was stirred for 15 min., and then allyl bromide (0.67 mL, 7.74 mmol) was added, and the reaction was stirred for 1 h. The reaction was poured into water and extracted with ethyl acetate. The combined organic layers were washed with brine, dried over sodium sulfate, filtered, and concentrated in vacuo to yield a crude product that was purified by column chromatography (150 mL SiO_2, 6% ethyl acetate:hexanes) to give **70** (1.1987 g, 66.3%) as a white crystalline solid, m.p. 165.1-166.7 °C: ^{1}H NMR (400 MHz, $CDCl_3$) δ 8.95 (s, 1H), 8.81 (s, 1H), 7.17 (s, 1H), 7.02 (s, 1H), 6.07-5.97 (dddd, J = 15.6, 11.2, 6.8, 5.6, 1H), 5.20 (d, J =16.0, 1H), 5.18 (d, J = 11.2, 1H), 4.86-4.80 (dd, J = 15.2, 5.6, 1H), 4.15-4.10 (dd, J = 14.8, 6.8, 1H), 3.86 (s, 3H), 2.04 (s, 3H), 1.67 (s, 4H), 1.31 (s, 3H), 1.26 (s, 3H), 1.23 (s, 3H), 1.21 (s, 3H); ^{13}C NMR (100.6 MHz, $CDCl_3$) δ 165.2, 162.0, 160.1, 159.6, 144.0, 143.7, 139.2, 133.0, 131.9, 128.9, 125.6, 117.9, 112.9, 53.9, 51.7, 35.0, 34.9, 34.0, 33.9, 32.0, 31.9, 31.7, 31.6, 17.6; ES-MS (M+H)+ calcd for $C_{24}H_{32}N_3O_2$ 394.2495, found 394.2486.

2-(Allyl(3,5,5,8,8-pentamethyl-5,6,7,8-tetrahydronaphthalen-2-yl)amino)pyrimidine-5-carboxylic acid (33). To a 100 mL round-bottomed flask equipped with a stir bar and charged with methyl ester **70** (1.0549 g, 2.681 mmol) suspended in methanol (9.0 mL) was added a solution of potassium hydroxide (0.4895 g, 8.72 mmol) in water (0.59 mL). This reaction was stirred at reflux in an oil bath at 87 °C for 1.5h. The reaction was then cooled to r.t. and acidified with 1N HCl (85 mL). The resulting precipitate was filtered and washed with copious amounts of cold water to give a crude product that was purified by column chromatography (25 mL SiO_2, 40% ethyl acetate:hexanes) to give pure **33** (0.8575 g, 84.3%) as a white crystalline solid, m.p. 216.7 – 217.7 °C: ^{1}H NMR (400 MHz, d6-DMSO) δ 12.89 (br s, 1H), 8.86 (s, 1H), 8.67 (s, 1H), 7.21 (s, 1H), 7.06 (s, 1H), 6.03-5.93 (dddd, J = 16.0, 11.6, 6.0, 5.6, 1H), 5.16 (d, J =16.0, 1H), 5.14 (d, J = 11.6, 1H), 4.75-4.70 (dd, J = 15.2, 5.6, 1H), 4.22-4.17 (dd, J = 15.2, 6.4, 1H), 1.95 (s, 3H), 1.61 (s, 4H), 1.26 (s, 3H), 1.24 (s, 3H), 1.18 (s, 6H) ;^{13}C NMR (100.6 MHz, d6-DMSO) δ 165.5, 161.8, 159.7, 143.1, 143.0, 139.5, 133.6, 132.2, 128.5, 125.5, 117.8, 113.7, 53.2, 34.6, 34.5, 33.7, 33.6, 31.7, 31.6, 31.5, 17.4; ES-MS (M+Na)+ calcd for $C_{23}H_{29}N_3O_2Na$ 402.2158, found 402.2153.

Methyl 4-(allyl(3,5,5,8,8-pentamethyl-5,6,7,8-tetrahydronaphthalen-2-yl)aminobenzoate (71). To a flame-dried, 100 mL round-bottomed flask equipped with a magnetic stir bar was added a 60% dispersion of sodium hydride in mineral oil (0.3564 g, 8.91 mmol). The dispersion of sodium hydride was washed with hexanes (3.0 mL, twice) and dried under vacuum and suspended in 4.8 mL of DMF under nitrogen. To this solution of sodium hydride in DMF was added a solution of **61**[4] (1.3122 g, 3.733 mmol) in DMF (14.0 mL), and the reaction was stirred for 15 min., and then allyl bromide (0.55 mL, 6.40 mmol) was added, and the reaction was stirred for 1 h. The reaction was poured into water and extracted with ethyl acetate. The combined organic layers were washed with brine, dried over sodium sulfate, filtered, and concentrated in vacuo to yield a crude product that was purified by column chromatography (150 mL SiO_2, 2% ethyl acetate:hexanes) to give **71** (1.1947 g, 81.7%) as a white crystalline solid, m.p.

101.9-104.6 °C: ^{1}H NMR (400 MHz, $CDCl_3$) δ 7.81 (d, J = 9.2, 2H), 7.19 (s, 1H), 7.04 (s, 1H), 6.46 (d, J =8.8, 2H), 6.01-5.91 (dddd, J = 17.2, 10.8, 5.6, 5.2, 1H), 5.29 (dd, J =17.2, 1.6, 1H), 5.22 (dd, J = 10.4, 1.6, 1H), 4.21 (br s, 2H), 3.83 (s, 3H), 2.04 (s, 3H), 1.68 (s, 4H), 1.30 (s, 6H), 1.21 (s, 6H); ^{13}C NMR (100.6 MHz, $CDCl_3$) δ 167.3, 151.9, 144.4, 143.9, 141.6, 133.5, 132.8, 131.1, 129.3, 126.7, 117.4, 117.0, 111.6, 54.5, 51.4, 35.0, 34.9, 34.1, 34.0, 31.9, 31.8, 17.6; ES-MS (M+Na)+ calcd for $C_{26}H_{33}NO_2Na$ 414.2409, found 414.2408.

4-(Allyl(3,5,5,8,8-pentamethyl-5,6,7,8-tetrahydronaphthalen-2-yl)amino)benzoic acid (34). To a 100 mL round-bottomed flask equipped with a stir bar and charged with methyl ester **71** (0.6599 g, 1.685 mmol) suspended in methanol (4.0 mL) was added a solution of potassium hydroxide (0.3854 g, 6.87 mmol) in water (0.38 mL). This reaction was stirred at reflux in an oil bath at 87 °C for 1.5h. The reaction was then cooled to r.t. and acidified with 1N HCl (90 mL). The resulting precipitate was filtered and washed with copious amounts of cold water to give a crude product that was purified by column chromatography (25 mL SiO_2, 15% to 30% ethyl acetate:hexanes) to give pure **34** (0.5233 g, 82.2%) as a white crystalline solid, m.p. 235.2 – 238.8 °C: ^{1}H NMR (400 MHz, d6-DMSO) δ 12.14 (br s, 1H), 7.68 (d, J = 8.8, 2H), 7.28 (s, 1H), 7.07 (s, 1H), 6.41 (d, J =8.8, 2H), 5.99-5.89 (dddd, J = 17.2, 10.4, 5.6, 5.2, 1H), 5.27 (dd, J = 17.2, 1.6, 1H), 5.20 (dd, J = 10.4, 1.6, 1H), 4.21 (br s, 2H), 1.98 (s, 3H), 1.62 (s, 4H), 1.25 (s, 6H), 1.12 (s, 6H);^{13}C NMR (100.6 MHz, d6-DMSO) δ 167.4, 151.3, 143.9, 143.3, 141.4, 133.8, 132.6, 131.0, 129.2, 126.3, 117.8, 116.9, 111.3, 54.0, 34.6, 34.4, 33.8, 33.7, 31.6, 31.6, 17.2; ES-MS (-) (M–H)- calcd for $C_{25}H_{30}NO_2$ 376.2277, found 376.2270.

Methyl 2-(allyl(5,5,8,8-tetramethyl-5,6,7,8-tetrahydronaphthalen-2-yl)amino)pyrimidine-5-carboxylate (72). To a flame-dried, 100 mL round-bottomed flask equipped with a magnetic stir bar was added a 60% dispersion of sodium hydride in mineral oil (0.3127 g, 7.82 mmol). The dispersion of sodium hydride was washed with hexanes (3.0 mL, twice) and dried under vacuum and suspended in 4.2 mL of DMF under nitrogen. To this solution of sodium hydride in DMF was added a solution of **63**[4] (1.1785 g, 3.472 mmol) in DMF (12.2 mL), and the reaction was stirred for 15 min., and then allyl bromide (0.50 mL, 5.78 mmol) was added, and the reaction was stirred for 1 h. The reaction was poured into water and extracted with ethyl acetate. The combined organic layers were washed with brine, dried over sodium sulfate, filtered, and concentrated in vacuo to yield a crude product that was purified by column chromatography (150 mL SiO_2, 2% to 6% to 10% ethyl acetate:hexanes) to give **72** (0.9160 g, 69.5%) as a white crystalline solid, m.p. 161.6-165.8 °C: ^{1}H NMR (400 MHz, $CDCl_3$) δ 8.88 (s, 2H), 7.31 (d, J = 8.4, 1H), 7.20 (d, J = 2.4, 1H), 7.05 (dd, J = 8.4, 2.4, 1H), 6.06-5.96 (dddd, J = 16.8, 10.4, 5.2, 5.2, 1H), 5.23-5.18 (dd, J = 17.2, 1.6, 1H), 5.20-5.17 (dd, J = 10.4, 1.6, 1H), 4.62-4.60 (d, J = 5.6, 1H), 3.86 (s, 3H), 1.68 (s, 4H), 1.29 (s, 6H), 1.23 (s, 6H); ^{13}C NMR (100.6 MHz, $CDCl_3$) δ 165.0, 161.9, 159.6, 145.8, 143.3, 140.5, 133.2, 127.3, 124.4, 123.5, 117.0, 113.4, 54.2, 51.8, 34.9, 34.8, 34.3, 34.0, 31.7; ES-MS (M+H)+ calcd for $C_{23}H_{29}N_3O_2$ 380.2338, found 380.2329.

2-(Allyl(5,6,8,8-tetramethyl-5,6,7,8-tetrahydronaphthalen-2-yl)amino)pyrimidine-5-carboxylic acid (35). To a 100 mL round-bottomed flask equipped with a stir bar and charged with methyl ester **72** (0.8218 g, 2.166 mmol) suspended in methanol (7.6 mL) was added a solution of potassium hydroxide (0.4129 g, 7.36 mmol) in water (0.48 mL). This reaction was stirred at reflux in an oil bath at 87 °C for 1.5h. The reaction was then cooled to r.t. and acidified with 1N HCl (85 mL). The resulting precipitate was filtered and washed with copious amounts of cold water to give a crude product that was purified by column chromatography (25 mL SiO_2, 15% to 60% ethyl acetate:hexanes) to give pure **35** (0.7013 g, 88.6%) as a white crystalline solid, m.p. 212.0 – 213.7 °C: ^{1}H NMR (400 MHz, d6-DMSO) δ 12.92 (br s, 1H), 8.75 (s, 2H), 7.32 (d, J = 8.4, 1H), 7.23 (d, J = 2.4, 1H), 7.04 (dd, J = 8.4, 2.0, 1H), 5.99-5.89 (dddd, J = 17.2, 10.4, 5.6, 4.8, 1H), 5.16-5.11 (dd, J = 17.2, 1.6, 1H), 5.13-5.10 (dd, J = 10.4, 1.6, 1H), 4.58 (d, J = 5.2, 2H), 1.62 (s, 4H), 1.23 (s, 6H), 1.18 (s, 6H);^{13}C NMR (100.6 MHz, d6-DMSO) δ 165.5, 162.1, 159.5, 145.2, 142.2, 140.6, 133.8, 126.9, 124.6, 124.5, 116.7, 114.0, 53.2, 34.5, 34.4, 34.0, 33.7, 31.6, 31.5; ES-MS (M+Na)+ calcd for $C_{22}H_{27}N_3O_2Na$ 388.2001, found 388.2011.

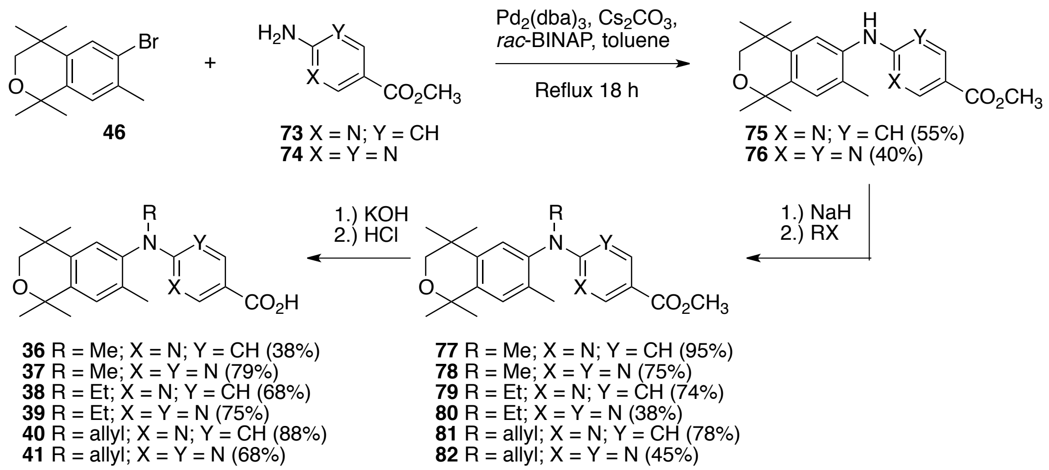

Scheme 10.

Methyl 6-((1,1,4,4,7-pentamethylisochroman-6-yl)amino)nicotinate (75). To a solution of **46** (2.2436 g, 7.922 mmol), **73** (1.1979 g, 7.873 mmol), Cs_2CO_3 (6.2394 g, 19.24 mmol), rac-BINAP (0.3781 g, 0.6072 mmol) in toluene (9.0 mL) in a 100 mL round-bottomed flask was added $Pd_2(dba)_3$ (0.3541 g, 0.3867 mmol). The solution was sparged with nitrogen for 5 min., then a reflux condenser was fitted to the flask, the atmosphere was evacuated and back-filled with nitrogen (three times), and the reaction was heated to reflux with stirring in an oil bath (125-120 °C) for 22h. After cooling the reaction to room temperature, excess cesium carbonate and other solid particulates were filtered and washed with ethyl acetate, and the organic filtrate was concentrated in vacuo to give a crude product that was purified by column chromatography (150 mL SiO_2, 8% ethyl acetate:hexanes to 15% ethyl acetate: hexanes) to give **75** (1.5387 g, 55.1%) as a crystalline solid, m.p. 186.3-188.8 °C: ^1H NMR (400 MHz, $CDCl_3$) δ 8.77 (dd, J = 2.2, 0.6, 1H), 8.03 (dd, J = 8.8, 2.2, 1H), 7.39 (br s, 1H), 7.26 (s, 1H), 6.96 (s, 1H), 6.51 (dd, J = 8.8, 0.6, 1H), 3.87 (s, 3H), 3.59 (s, 2H), 2.21 (s, 3H), 1.53 (s, 6H), 1.24 (s, 6H); ^{13}C NMR (100.6 MHz, $CDCl_3$) δ 165.8, 159.3, 141.6, 139.7, 139.3, 134.6, 130.7, 128.0, 121.7, 116.3, 106.0, 74.9, 70.6, 51.8, 33.7, 29.7, 26.9, 17.7; ES-MS (M+H)+ calcd for $C_{21}H_{27}N_2O_3$ 355.2022, found 355.2012.

Methyl 2-((1,1,4,4,7-pentamethylisochroman-6-yl)amino)pyrimidine-5-carboxylate (76). To a solution of **46** (2.2669 g, 8.004 mmol), **74** (1.2081 g, 7.889 mmol), Cs_2CO_3 (6.2373 g, 19.14 mmol), rac-BINAP (0.3792 g, 0.609 mmol) in toluene (9.0 mL) in a 100 mL round-bottomed flask was added $Pd_2(dba)_3$ (0.3553 g, 0.388 mmol). The solution was sparged with nitrogen for 5 min., then a reflux condenser was fitted to the flask, the atmosphere was evacuated and back-filled with nitrogen (three times), and the reaction was heated to reflux with stirring in an oil bath (125-120 °C) for 22h. After cooling the reaction to room temperature, excess cesium carbonate and other solid particulates were filtered and washed with ethyl acetate, and the organic filtrate was concentrated in vacuo to give a crude product that was purified by column chromatography (150 mL SiO_2, 5% to 15% to 20% to 30% ethyl acetate: hexanes) to give **76** (1.1300 g, 40.3%) as a crystalline solid, m.p. 130.7-135.8 °C: ^1H NMR (400 MHz, $CDCl_3$) δ 8.93 (s. 2H), 7.68 (s, 1H), 7.57 (br s, 1H), 6.93 (s, 1H), 3.90 (s, 3H), 3.59 (s, 2H), 2.26 (s, 3H), 1.53 (s, 6H), 1.27 (s, 6H); ^{13}C NMR (100.6 MHz, $CDCl_3$) δ 164.6, 161.6, 160.1, 141.0, 139.0, 133.9, 128.6, 127.6, 120.6, 114.8, 74.9, 70.7, 52.0, 33.8, 29.7, 26.9, 17.9; ES-MS (M+Na)+ calcd for $C_{20}H_{25}N_3O_3Na$ 378.1794, found 378.1805.

Methyl 6-(methyl(1,1,4,4,7-pentamethylisochroman-6-yl)amino)nicotinate (77). To a flame-dried, 100 mL round-bottomed flask equipped with a magnetic stir bar was added a 60% dispersion of sodium hydride in mineral oil (0.4325 g, 10.81 mmol). The dispersion of sodium hydride was washed with hexanes (3.8 mL, twice) and dried under vacuum and suspended in 5.8 mL of DMF under nitrogen. To this solution of sodium hydride in DMF was added a solution of **75** (1.5942 g, 4.4978 mmol) in DMF (17.3 mL), and the reaction was stirred for 15 min., and then methyl iodide (0.43 mL, 6.9 mmol) was added, and the reaction was stirred for 1 h. The reaction was poured into water and extracted with ethyl acetate. The combined organic layers were washed with brine, dried over sodium sulfate, filtered, and

concentrated in vacuo to yield a crude product that was purified by column chromatography (150 mL SiO_2, 10% ethyl acetate:hexanes) to give **77** (1.5788 g, 95.26%) as a white crystalline solid, m.p. 166.1-168.3 °C: ^{1}H NMR (400 MHz, $CDCl_3$) δ 8.87 (d, J = 2.0, 1H), 7.84 (dd, J = 8.8, 2.0, 1H), 7.06 (s, 1H), 6.99 (s, 1H), 6.04 (d, J = 8.8, 1H), 3.85 (s, 3H), 3.58 (s, 2H), 3.47 (s, 3H), 2.06 (s, 3H), 1.55 (s, 6H), 1.22 (s, 6H); ^{13}C NMR (100.6 MHz, $CDCl_3$) δ 166.2, 142.6, 141.4, 141.3, 137.9, 133.0, 128.4, 124.5, 114.4, 107.1, 75.0, 70.6, 51.6, 38.1, 33.8, 29.7, 27.0, 17.2; ES-MS (M+H)+ calcd for $C_{22}H_{29}N_2O_3$ 369.2178, found 369.2169.

Methyl 2-(methyl(1,1,4,4,7-pentamethylisochroman-6-yl)amino)pyrimidine-5-carboxylate (78). To a flame-dried, 100 mL round-bottomed flask equipped with a magnetic stir bar was added a 60% dispersion of sodium hydride in mineral oil (0.2960 g, 7.401 mmol). The dispersion of sodium hydride was washed with hexanes (3.0 mL, twice) and dried under vacuum and suspended in 3.8 mL of DMF under nitrogen. To this solution of sodium hydride in DMF was added a solution of **76** (1.0752 g, 3.025 mmol) in DMF (11.0 mL), and the reaction was stirred for 15 min., and then methyl iodide (0.28 mL, 4.5 mmol) was added, and the reaction was stirred for 1 h. The reaction was poured into water and extracted with ethyl acetate. The combined organic layers were washed with brine, dried over sodium sulfate, filtered, and concentrated in vacuo to yield a crude product that was purified by column chromatography (150 mL SiO_2, 8.5% to 10% ethyl acetate:hexanes) to give **78** (0.8375 g, 74.9%) as a white crystalline solid, m.p. 216.9-217.8 °C: ^{1}H NMR (400 MHz, $CDCl_3$) δ 8.95 (d, J = 2.0, 1H), 8.79 (d, J = 2.4, 1H), 7.05 (s, 1H), 6.96 (s, 1H), 3.86 (s, 3H), 3.57 (q, J = 11.6, 2H), 3.47 (s, 3H), 2.06 (s, 3H), 1.56 (s, 3H), 1.53 (s, 3H), 1.24 (s, 3H), 1.22 (s, 3H); ^{13}C NMR (100.6 MHz, $CDCl_3$) δ 165.2, 162.5, 159.9, 159.7, 141.8, 141.4, 140.7, 132.3, 128.0, 123.6, 112.9, 75.0, 70.7, 51.7, 38.8, 33.7, 30.3, 29.3, 27.4, 26.5, 17.5; ES-MS (M+H)+ calcd for $C_{21}H_{28}N_3O_3$ 370.2131, found 370.2126.

Methyl 6-(ethyl)1,1,4,4,7-pentamethylisochroman-6-yl)amino)nicotinate (79). To a flame-dried, 100 mL round-bottomed flask equipped with a magnetic stir bar was added a 60% dispersion of sodium hydride in mineral oil (0.4132 g, 10.33 mmol). The dispersion of sodium hydride was washed with hexanes (3.0 mL, twice) and dried under vacuum and suspended in 5.7 mL of DMF under nitrogen. To this solution of sodium hydride in DMF was added a solution of **75** (1.5064 g, 4.2501 mmol) in DMF (16.8 mL), and the reaction was stirred for 15 min., and then ethyl iodide (0.54 mL, 6.7 mmol) was added, and the reaction was stirred for 1 h. The reaction was poured into water and extracted with ethyl acetate. The combined organic layers were washed with brine, dried over sodium sulfate, filtered, and concentrated in vacuo to yield a crude product that was purified by column chromatography (150 mL SiO_2, 6% ethyl acetate:hexanes) to give **79** (1.2146 g, 74.7%) as a white crystalline solid, m.p. 106.3-108.3 °C: ^{1}H NMR (400 MHz, $CDCl_3$) δ 8.85 (d, J = 2.0, 1H), 7.82 (dd, J = 8.8, 2.0, 1H), 7.01 (s, 1H), 7.00 (s, 1H), 5.94 (d, J = 8.8, 1H), 4.25 (m, 1H), 3.84 (s, 3H), 3.69 (m, 1H), 3.59 (s, 2H), 2.05 (s, 3H), 1.56 (s, 3H), 1.54 (s, 3H), 1.24 (t, J = 7.2, 3H), 1.23 (s, 3H), 1.21 (s, 3H); ^{13}C NMR (100.6 MHz, $CDCl_3$) δ 166.2, 159.4, 150.3, 142.4, 141.4, 139.6, 138.1, 133.4, 128.4, 125.6, 114.3, 107.0, 75.0, 70.6, 51.6, 33.7, 29.8, 27.0, 17.3, 12.9; ES-MS (M+H)+ calcd for $C_{23}H_{31}N_2O_3$ 383.2335, found 383.2338.

Methyl 2-(ethyl(1,1,4,4,7-pentamethylisochroman-6-yl)amino)pyrimidine-5-carboxylate (80). To a flame-dried, 100 mL round-bottomed flask equipped with a magnetic stir bar was added a 60% dispersion of sodium hydride in mineral oil (0.2848 g, 7.12 mmol). The dispersion of sodium hydride was washed with hexanes (3.0 mL, twice) and dried under vacuum and suspended in 3.8 mL of DMF under nitrogen. To this solution of sodium hydride in DMF was added a solution of **76** (1.0788 g, 3.035 mmol) in DMF (11.0 mL), and the reaction was stirred for 15 min., and then ethyl iodide (0.36 mL, 4.5 mmol) was added, and the reaction was stirred for 1 h. The reaction was poured into water and extracted with ethyl acetate. The combined organic layers were washed with brine, dried over sodium sulfate, filtered, and concentrated in vacuo to yield a crude product that was purified by column chromatography (150 mL SiO_2, 8.5% to 10% ethyl acetate:hexanes) to give **80** (0.4435 g, 38.1%) as a white crystalline solid, m.p. 172.1 - 173.5 °C: ^{1}H NMR (400 MHz, $CDCl_3$) δ 8.94 (s, 1H), 8.78 (s, 1H), 7.01 (s, 1H), 6.97 (s, 1H), 4.18-4.09 (sext, J = 7.2, 1H), 3.86 (s, 3H), 3.82-3.74 (sext, J = 7.2, 1H), 3.62-3.54 (q, J = 11.6, 2H), 2.05 (s, 3H), 1.57 (s, 3H), 1.53 (s, 3H), 1.26 (t, J =3.6, 3H), 1.25 (s, 3H), 1.22 (s, 3H); ^{13}C NMR (100.6 MHz, $CDCl_3$) δ 165.3, 162.0, 160.0,

159.8, 141.5, 140.6, 139.8, 132.7, 128.0, 124.6, 112.8, 75.0, 70.7, 51.7, 46.0, 33.7, 30.2, 29.4, 27.5, 26.5, 17.6, 12.7; ES-MS (M+H)+ calcd for $C_{22}H_{30}N_3O_3$ 384.2287, found 384.2274.

Methyl 6-(allyl(1,1,4,4,7-pentamethylisochroman-6-yl)amino)nicotinate (81). To a flame-dried, 100 mL round-bottomed flask equipped with a magnetic stir bar was added a 60% dispersion of sodium hydride in mineral oil (0.4580 g, 4.543 mmol). The dispersion of sodium hydride was washed with hexanes (3.0 mL, twice) and dried under vacuum and suspended in 3.8 mL of DMF under nitrogen. To this solution of sodium hydride in DMF was added a solution of **75** (1.6104 g, 11.45 mmol) in DMF (5.8 mL), and the reaction was stirred for 15 min., and then allyl bromide (0.67 mL, 7.7 mmol) was added, and the reaction was stirred for 1 h. The reaction was poured into water and extracted with ethyl acetate. The combined organic layers were washed with brine, dried over sodium sulfate, filtered, and concentrated in vacuo to yield a crude product that was purified by column chromatography (150 mL SiO_2, 6% ethyl acetate:hexanes) to give **81** (1.4000 g, 78.1%) as a dull yellow crystalline solid, m.p. 92.7 - 94.9 °C: ^1H NMR (400 MHz, $CDCl_3$) δ 8.86 (d, J = 2.0, 1H), 7.86 (dd, J = 8.8, 1.6, 1H), 7.02 (s, 1H), 6.98 (s, 1H), 6.08-5.98 (m, 2H), 5.20-5.15 (m, 2H), 4.94 (m, 1H), 4.12 (m, 1H), 3.85 (s, 3H), 3.57 (s, 2H), 2.05 (s, 3H), 1.54 (s, 6H), 1.20 (s, 6H); ^{13}C NMR (100.6 MHz, $CDCl_3$) δ 166.0, 158.7, 149.6, 142.3, 141.6, 139.5, 138.5, 133.1, 132.9, 128.4, 125.5, 118.0, 114.8, 107.5, 74.9, 70.6, 53.1, 51.7, 33.7, 29.7, 26.9, 17.4; ES-MS (M+Na)+ calcd for $C_{24}H_{30}N_2O_3Na$ 417.2154, found 417.2154.

Methyl 2-(allyl(1,1,4,4,7-pentamethylisochroman-6-yl)amino)pyrimidine-5-carboxylate (82). To a flame-dried, 100 mL round-bottomed flask equipped with a magnetic stir bar was added a 60% dispersion of sodium hydride in mineral oil (0.2269 g, 5.673 mmol). The dispersion of sodium hydride was washed with hexanes (3.0 mL, twice) and dried under vacuum and suspended in 2.8 mL of DMF under nitrogen. To this solution of sodium hydride in DMF was added a solution of **76** (0.8014 g, 2.255 mmol) in DMF (8.1 mL), and the reaction was stirred for 15 min., and then allyl bromide (0.33 mL, 3.8 mmol) was added, and the reaction was stirred for 1 h. The reaction was poured into water and extracted with ethyl acetate. The combined organic layers were washed with brine, dried over sodium sulfate, filtered, and concentrated in vacuo to yield a crude product that was purified by column chromatography (150 mL SiO_2, 6% ethyl acetate:hexanes) to give **82** (0.4061 g, 45.5%) as a crystalline solid, m.p. 167.1 - 172.0 °C: ^1H NMR (400 MHz, $CDCl_3$) δ 9.01 (s, 1H), 8.88 (s, 1H), 7.02 (s, 1H), 6.98 (s, 1H), 6.06-5.96 (dddd, J = 6.8, 10.4, 14.8, 17.2, 1H), 5.26-5.21 (dd, J = 18.0, 1.2, 1H), 5.24-5.21 (dd, J =10.4, 1.2, 1H), 4.23-4.18 (dd, J = 6.8, 14.8, 1H), 3.88 (s, 3H), 3.63-3.60 (d, J = 11.2, 1H), 3.55-3.52 (d, J = 11.6, 1H), 2.05 (s, 3H), 1.56 (s, 3H), 1.53 (s, 3H), 1.22 (s, 6H); ^{13}C NMR (100.6 MHz, $CDCl_3$) δ 164.4, 160.1, 160.0, 159.1, 141.7, 141.4, 139.0, 132.3, 132.0, 128.1, 124.4, 118.9, 113.3, 75.0, 70.6, 54.2, 33.7, 30.1, 29.4, 27.4, 26.4, 17.6; ES-MS (M+Na)+ calcd for $C_{23}H_{29}N_3O_3Na$ 418.2107, found 418.2101.

6-(Methyl(1,1,4,4,7-pentamethylisochroman-6-yl)amino)nicotinic acid (36). To a 100 mL round-bottomed flask equipped with a stir bar and charged with methyl ester **77** (1.0403 g, 2.823 mmol) suspended in methanol (8.1 mL) was added a solution of potassium hydroxide (0.5223 g, 9.309 mmol) in water (0.52 mL). This reaction was stirred at reflux in an oil bath at 87 °C for 1.5h. The reaction was then cooled to r.t. and acidified with 1N HCl (90 mL). The solution was extracted with ethyl acetate (2 X 70 mL), and the organic layers were dried over sodium sulfate and concentrated in vacuo to give a crude product that was purified by column chromatography (25 mL SiO_2, 30% ethyl acetate:hexanes to 60% ethyl acetate:hexanes) to give pure **36** (0.3879 g, 38.7%) as a white crystalline solid, m.p. 294.5-296.9 °C: ^1H NMR (400 MHz, d6-DMSO) δ 12.50 (br s, 1H), 8.68 (s, 1H), 7.82 (d, J = 7.6, 1H), 7.19 (d, J = 2.0, 2H), 6.00 (br s, 1H), 3.49 (s, 2H), 3.35 (s, 3H), 1.99 (s, 3H), 1.47 (s, 3H), 1.16 (s, 6H); ^{13}C NMR (100.6 MHz, d6-DMSO) δ 166.7, 159.9, 150.6, 142.2, 141.5, 140.8, 137.9, 132.7, 128.3, 124.4, 114.7, 106.0, 74.5, 69.7, 37.6, 33.4, 29.6, 26.7, 16.9; ES-MS (M+Na)+ calcd for $C_{21}H_{26}N_2O_3Na$ 377.1814, found 377.1830.

2-(Methyl(1,1,4,4,7-pentamethylisochroman-6-yl)amino)pyrimidine-5-carboxylic acid (37). To a 100 mL round-bottomed flask equipped with a stir bar and charged with methyl ester **78** (0.7964 g, 2.156 mmol) suspended in methanol (7.0 mL) was added a solution of potassium hydroxide (0.4088 g, 7.286 mmol) in water (0.47 mL). This reaction was stirred at reflux in an oil bath at 87 °C for 1.5h. The reaction was then

cooled to r.t. and acidified with 1N HCl (80 mL). The solution was extracted with ethyl acetate (2 X 70 mL), and the organic layers were dried over sodium sulfate and concentrated in vacuo to give a crude product that was purified by column chromatography (25 mL SiO_2, 60% ethyl acetate:hexanes to pure ethyl acetate to 2% methanol:ethyl acetate) to give pure **37** (0.6112 g, 79.7%) as a white crystalline solid, m.p. 332.0-334.0 °C: ^{1}H NMR (400 MHz, d6-DMSO) δ 12.88 (br s, 1H), 8.87 (s, 1H), 8.66 (s, 1H), 7.19 (s, 1H), 7.11 (s, 1H), 3.48 (s, 2H), 3.41 (s, 3H), 1.96 (s, 3H), 1.48 (s, 3H), 1.45 (s, 3H), 1.17 (s, 3H), 1.15 (s, 3H); ^{13}C NMR (100.6 MHz, d6-DMSO) δ 165.6, 162.1, 159.7, 159.6, 141.5, 141.4, 140.2, 132.4, 127.6, 123.6, 113.4, 74.5, 69.8, 33.4, 29.7, 29.5, 26.9, 26.5, 17.1; ES-MS (M+H)+ calcd for $C_{20}H_{26}N_3O_3$ 356.1974, found 356.1962.

6-(Ethyl(1,1,4,4,7-pentamethylisochroman-6-yl)amino)nicotinic acid (38). To a 100 mL round-bottomed flask equipped with a stir bar and charged with methyl ester **79** (1.0918 g, 2.854 mmol) suspended in methanol (8.2 mL) was added a solution of potassium hydroxide (0.5468 g, 9.656 mmol) in water (0.52 mL). This reaction was stirred at reflux in an oil bath at 87 °C for 1.5h. The reaction was then cooled to r.t. and acidified with 1N HCl (80 mL). The solution was extracted with ethyl acetate (2 X 70 mL), and the organic layers were dried over sodium sulfate and concentrated in vacuo to give a crude product that was purified by column chromatography (25 mL SiO_2, 30% ethyl acetate:hexanes to 60% ethyl acetate:hexanes) to give pure **38** (0.7156 g, 68.0%) as a white crystalline solid, m.p. 280.4-282.1 °C: ^{1}H NMR (400 MHz, d6-DMSO) δ 12.48 (br s, 1H), 8.67 (d, J = 2.0, 1H), 7.80 (dd, J = 8.8, 2.0, 1H), 7.21 (s, 1H), 7.12 (s, 1H), 5.91 (br s, 1H), 4.11 (m, 1H), 3.66 (m, 1H), 3.50 (s, 2H), 1.99 (s, 3H), 1.48 (s, 3H), 1.47 (s, 3H), 1.17 (s, 3H), 1.13 (t, J = 7.2, 3H); ^{13}C NMR (100.6 MHz, d6-DMSO) δ 166.7, 159.4, 150.7, 142.0, 140.9, 139.7, 138.1, 133.1, 128.3, 125.4, 114.6, 106.0, 74.5, 69.7, 44.2, 33.4, 29.6, 26.7, 17.0, 12.8; ES-MS (M+H)+ calcd for $C_{22}H_{29}N_2O_3$ 369.2178, found 369.2167.

2-(Ethyl(1,1,4,4,7-pentamethylisochroman-6-yl)amino)pyrimidine-5-carboxylic acid (39). To a 100 mL round-bottomed flask equipped with a stir bar and charged with methyl ester **80** (0.4017 g, 1.048 mmol) suspended in methanol (4.0 mL) was added a solution of potassium hydroxide (0.2080 g, 3.707 mmol) in water (0.24 mL). This reaction was stirred at reflux in an oil bath at 87 °C for 1.5h. The reaction was then cooled to r.t. and acidified with 1N HCl (80 mL). The solution was extracted with ethyl acetate (2 X 70 mL), and the organic layers were dried over sodium sulfate and concentrated in vacuo to give a crude product that was purified by column chromatography (25 mL SiO_2, 40% ethyl acetate:hexanes) to give pure **39** (0.2931 g, 75.7%) as a white crystalline solid, m.p. 249.0-250.3 °C: ^{1}H NMR (400 MHz, d6-DMSO) δ 12.86 (br s, 1H), 8.86 (s, 1H), 8.65 (d, J = 2.0, 1H), 7.12 (s, 1H), 7.11 (s, 1H), 4.06-3.98 (sext, J = 6.8, 1H), 3.83-3.74 (sext, J = 6.8, 1H), 3.51 (d, J = 11.6, 1H), 3.48 (d, J = 11.2, 1H), 1.96 (s, 3H), 1.48 (s, 3H), 1.43 (s, 3H), 1.18 (s, 3H), 1.15 (t, J = 6.8, 3H), 1.15 (s, 3H); ^{13}C NMR (100.6 MHz, d6-DMSO) δ 165.6, 161.7, 159.7, 141.1, 140.2, 139.9, 132.8, 127.6, 124.4, 113.4, 74.5, 69.8, 45.4, 33.3, 29.7, 29.6, 26.8, 26.6, 17.3, 12.6; ES-MS (M+H)+ calcd for $C_{21}H_{28}N_3O_3$ 370.2131, found 370.2122.

6-(Allyl(1,1,4,4,7-pentamethylisochroman-6-yl)amino)nicotinic acid (40). To a 100 mL round-bottomed flask equipped with a stir bar and charged with methyl ester **81** (1.1035 g, 2.7972 mmol) suspended in methanol (8.0 mL) was added a solution of potassium hydroxide (0.5389 g, 9.604 mmol) in water (0.61 mL). This reaction was stirred at reflux in an oil bath at 87 °C for 1.5h. The reaction was then cooled to r.t. and acidified with 1N HCl (90 mL). The solution was extracted with ethyl acetate (2 X 70 mL), and the organic layers were dried over sodium sulfate and concentrated in vacuo to give a crude product that was purified by column chromatography (25 mL SiO_2, 30% to 60% ethyl acetate:hexanes) to give pure **40** (0.9411 g, 88.4%) as a white crystalline solid, m.p. 221.3-224.4 °C: ^{1}H NMR (400 MHz, d6-DMSO) δ 12.52 (br s, 1H), 8.67 (d, J = 1.6, 1H), 7.84 (dd, J = 9.2, 1.6, 1H), 7.19 (s, 1H), 7.12 (s, 1H), 6.02-5.92 (dddd, J = 16.4, 10.4, 6.0, 6.0, 1H), 5.96-5.94 (d, J = 10.0, 1H), 5.16-5.11 (dd, J = 16.8, 1.6, 1H), 5.12-5.10 (dd, J = 10.0, 1.6, 1H), 4.74 (m, 1H), 4.18 (m, 1H), 3.49 (s, 2H), 1.99 (s, 3H), 1.44 (s, 6H), 1.13 (s, 6H); ^{13}C NMR (100.6 MHz, d6-DMSO) δ 166.6, 159.4, 150.6, 141.8, 139.8, 138.2, 134.1, 133.1, 128.2, 125.3, 117.4, 115.0, 106.1, 74.5, 69.7, 52.2, 33.3, 29.6, 26.6, 17.1; ES-MS (M+H)+ calcd for $C_{23}H_{29}N_2O_3$ 381.2178, found 381.2176.

2-(Allyl(1,1,4,4,7-pentamethylisochroman-6-yl)amino)pyrimidine-5-carboxylic acid (41). To a 100 mL round-bottomed flask equipped with a stir bar and charged with methyl ester **82** (0.3600 g, 0.9034 mmol)

suspended in methanol (3.5 mL) was added a solution of potassium hydroxide (0.1967 g, 3.506 mmol) in water (0.24 mL). This reaction was stirred at reflux in an oil bath at 87 °C for 1.5h. The reaction was then cooled to r.t. and acidified with 1N HCl (80 mL) to give a precipitate. The precipitate was filtered and washed with copious amounts of cold water to give a crude product that was purified by column chromatography (25 mL SiO_2, 40% ethyl acetate:hexanes) to give pure **41** (0.2366 g, 68.1%) as a white crystalline solid, m.p. 203.1-205.1 °C: ^1H NMR (400 MHz, d6-DMSO) δ 12.90 (br s, 1H), 8.87 (s, 1H), 8.68 (s, 1H), 7.10 (s, 2H), 6.03-5.93 (dddd, J = 16.8, 10.8, 6.4, 6.0, 1H), 5.17-5.12 (dd, J = 16.8, 1.6, 1H), 5.15-5.12 (dd, J = 10.4, 1.2, 1H), 4.73-4.68 (dd, J = 15.2, 6.0, 1H), 4.28-4.23 (dd, J = 15.2, 6.8, 1H), 3.51 (d, J = 11.6, 1H), 3.47 (d, J = 11.6, 1H), 1.99 (s, 3H), 1.49 (s, 3H), 1.46 (s, 3H), 1.12 (s, 6H); ^{13}C NMR (100.6 MHz, d6-DMSO) δ 165.5, 161.8, 159.8, 140.9, 140.2, 140.0, 133.5, 132.8, 127.5, 124.4, 117.9, 113.8, 74.5, 69.7, 53.1, 33.3, 29.7, 29.6, 26.7, 26.6, 17.4; ES-MS (M+H)+ calcd for $C_{22}H_{28}N_3O_3$ 382.2131, found 382.2127.

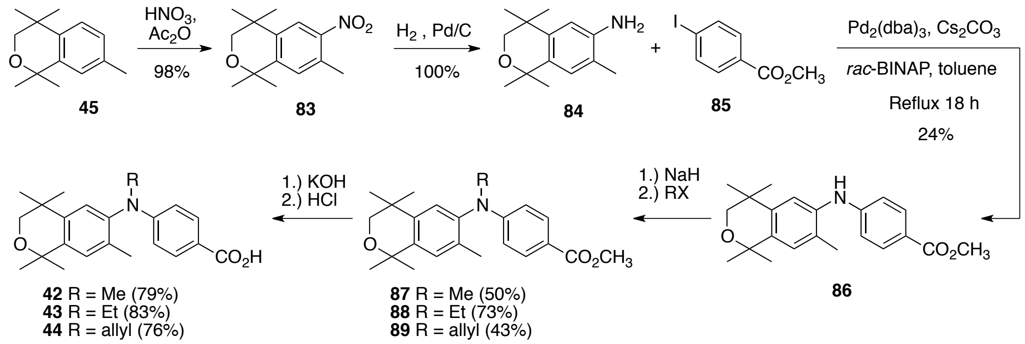

Scheme 11.

1,1,4,4,7-Pentamethyl-6-nitroisochroman (83). To a 100 mL round bottom flask equipped with a stir bar and containing 1,1,4,4,7-pentamethylisochroman (**45**) (6.0185 g, 29.458 mmol) dissolved in acetic anhydride (30.0 mL) cooled to 0 °C in an ice bath was added concentrated nitric acid (2.40 mL, 57.6 mmol), dropwise. After stirring at 0 °C for 20 min, the solution was poured into water (50 mL) and extracted with ethyl acetate, and the combined organic layers were dried over sodium sulfate, concentrated in vacuo to give a crude oil that was purified by column chromatography (150 mL SiO_2, hexanes to 10% ethyl acetate:hexanes) to give pure **83** (7.2334 g, 98.5%) as a dull yellow crystalline solid, m.p. 89.4-91.3 °C: ^1H NMR (400 MHz, $CDCl_3$) δ 7.93 (s, 1H), 6.99 (s, 1H), 3.58 (s, 2H), 2.56 (s, 3H), 1.53 (s, 6H), 1.28 (s, 6H); ^{13}C NMR (100.6 MHz, $CDCl_3$) δ 147.4, 147.3, 142.0, 130.9, 129.7, 121.9, 75.0, 70.4, 33.9, 29.4, 26.7, 20.5; ES-MS (M+Na)+ calcd for $C_{14}H_{19}NO_3Na$ 272.1263, found 272.1252.

1,1,4,4,7-pentamethylisochroman-6-amine (84). To a 300 mL round bottom flask equipped with a stir bar and containing 1,1,4,4,7-pentamethyl-6-nitroisochroman (**83**) (3.04 g, 12.2 mmol) dissolved in ethyl acetate (20.0 mL) was added 10% Pd/C (0.5975 g). The flask was fitted with a three-way glass stopcock to which one side was connected to a balloon containing hydrogen gas, and the other side was connected to high vacuum. The reaction flask was evacuated and back-filled with hydrogen gas three times and then allowed to stir overnight at room temperature. After stirring overnight, the solution was filtered through cotton to give **84** (2.67 g, 100%) as a dull yellow crystalline solid, m.p. 117.4-123.5 °C: ^1H NMR (400 MHz, $CDCl_3$) δ 6.82 (s, 1H), 6.79 (s, 1H), 5.37 (br s, 2H), 3.56 (s, 2H), 2.23 (s, 3H), 1.49 (s, 6H), 1.23 (s, 6H); ^{13}C NMR (100.6 MHz, $CDCl_3$) δ 141.4, 139.1, 134.4, 127.5, 122.5, 113.2, 74.8, 70.8, 33.6, 29.8, 26.9, 17.3; ES-MS (M+H)+ calcd for $C_{14}H_{22}NO$ 220.1701, found 220.1698.

Methyl 4-((1,1,4,4,7-pentamethylisochroman-6-yl)amino)benzoate (86). To a solution of **84** (5.3327 g, 24.314 mmol), 4-iodo-methylbenzoate **85** (6.5862 g, 25.13 mmol), Cs_2CO_3 (9.7904 g, 30.05 mmol), rac-BINAP (1.2082 g, 1.9404 mmol) in toluene (28.0 mL) in a 250 mL round-bottomed flask was added $Pd_2(dba)_3$ (1.1442 g, 1.2495 mmol). The solution was sparged with nitrogen for 5 min., then a reflux condenser was fitted to the flask, the atmosphere was evacuated and back-filled with nitrogen (three times), and the reaction was heated to reflux with stirring in an oil bath (125-120 °C) for 22h. After cooling the reaction to room temperature, excess cesium carbonate and other solid particulates were

filtered and washed with ethyl acetate, and the organic filtrate was concentrated in vacuo to give a crude product that was purified by column chromatography (150 mL SiO_2, 5% ethyl acetate:hexanes to 8% ethyl acetate: hexanes) to give **86** (2.0851 g, 24.2%) as a crystalline solid, m.p. 152.3-172.6 °C: ^{1}H NMR (400 MHz, $CDCl_3$) δ 7.91 (d, J = 8.4, 2H), 7.68 (br s, 1H), 7.25 (s, 1H), 6.93 (s, 1H), 6.83 (d, J = 8.8, 2H), 3.86 (s, 3H), 3.58 (s, 2H), 2.20 (s, 3H), 1.54 (s, 6H), 1.22 (s, 6H); ES-MS (M+Na)+ calcd for $C_{22}H_{27}NO_3Na$ 376.1889, found 376.1888.

Methyl 4-(methyl(1,1,4,4,7-pentamethylisochroman-6-yl)amino)benzoate (87). To a flame-dried, 100 mL round-bottomed flask equipped with a magnetic stir bar was added a 60% dispersion of sodium hydride in mineral oil (0.1994 g, 4.985 mmol). The dispersion of sodium hydride was washed with hexanes (3.0 mL, twice) and dried under vacuum and suspended in 3.0 mL of DMF under nitrogen. To this solution of sodium hydride in DMF was added a solution of **86** (0.6378 g, 1.8045 mmol) in DMF (6.0 mL), and the reaction was stirred for 15 min., and then methyl iodide (0.17 mL, 2.7 mmol) was added, and the reaction was stirred for 1 h. The reaction was poured into water and extracted with ethyl acetate. The combined organic layers were washed with brine, dried over sodium sulfate, filtered, and concentrated in vacuo to yield a crude product that was purified by column chromatography (150 mL SiO_2, 2% to 5% ethyl acetate:hexanes) to give **87** (0.3366 g, 50.76%) as a waxy red crystalline solid: ^{1}H NMR (400 MHz, $CDCl_3$) δ 7.85 (d, J = 9.2, 1H), 7.04 (s, 1H), 6.97 (s, 1H), 6.47 (d, J = 8.8, 1H), 3.84 (s, 3H), 3.59 (s, 2H), 3.27 (s, 3H), 2.05 (s, 3H), 1.56 (s, 6H), 1.22 (s, 6H); ^{13}C NMR (100.6 MHz, $CDCl_3$) δ 167.3, 152.4, 143.3, 142.2, 140.5, 133.4, 131.1, 128.2, 124.7, 117.5, 111.2, 75.0, 70.7, 51.4, 39.1, 33.7, 29.8, 27.0, 17.4; ES-MS (M+H)+ calcd for $C_{23}H_{30}NO_3$ 368.2226, found 368.2217.

Methy 4-(ethyl(1,1,4,4,7-pentamethylisochroman-6-yl)amino)benzoate (88). To a flame-dried, 100 mL round-bottomed flask equipped with a magnetic stir bar was added a 60% dispersion of sodium hydride in mineral oil (0.2025 g, 5.063 mmol). The dispersion of sodium hydride was washed with hexanes (3.0 mL, twice) and dried under vacuum and suspended in 2.6 mL of DMF under nitrogen. To this solution of sodium hydride in DMF was added a solution of **86** (0.7119 g, 2.0141 mmol) in DMF (7.9 mL), and the reaction was stirred for 15 min., and then ethyl iodide (0.25 mL, 3.1 mmol) was added, and the reaction was stirred for 1 h. The reaction was poured into water and extracted with ethyl acetate. The combined organic layers were washed with brine, dried over sodium sulfate, filtered, and concentrated in vacuo to yield a crude product that was purified by column chromatography (150 mL SiO_2, 5% ethyl acetate:hexanes) to give **88** (0.4162 g, 73.4%) as a white crystalline solid, m.p. 95.5-98.4 °C: ^{1}H NMR (400 MHz, $CDCl_3$) δ 7.82 (d, J = 9.2, 1H), 7.01 (s, 1H), 6.98 (s, 1H), 6.43 (d, J = 8.8, 1H), 3.83 (s, 3H), 3.66 (m, 2H), 3.59 (s, 2H), 2.04 (s, 3H), 1.56 (s, 6H), 1.25 (t, J = 7.2, 3H), 1.22 (s, 6H); ^{13}C NMR (100.6 MHz, $CDCl_3$) δ 167.3, 151.5, 142.1, 141.7, 140.6, 133.8, 131.3, 128.5, 117.2, 111.0, 75.0, 70.7, 51.4, 45.9, 33.7, 29.8, 26.9, 17.5, 12.5; ES-MS (M+Na)+ calcd for $C_{24}H_{31}NO_3Na$ 404.2202, found 404.2201.

Methyl 4-(allyl(1,1,4,4,7-pentamethylisochroman-6-yl)amino)benzoate (89). To a flame-dried, 100 mL round-bottomed flask equipped with a magnetic stir bar was added a 60% dispersion of sodium hydride in mineral oil (0.1983 g, 4.958 mmol). The dispersion of sodium hydride was washed with hexanes (3.0 mL, twice) and dried under vacuum and suspended in 2.0 mL of DMF under nitrogen. To this solution of sodium hydride in DMF was added a solution of **86** (0.6040 g, 1.709 mmol) in DMF (6.0 mL), and the reaction was stirred for 15 min., and then allyl bromide (0.26 mL, 3.0 mmol) was added, and the reaction was stirred for 1 h. The reaction was poured into water and extracted with ethyl acetate. The combined organic layers were washed with brine, dried over sodium sulfate, filtered, and concentrated in vacuo to yield a crude product that was purified by column chromatography (150 mL SiO_2, 5% ethyl acetate:hexanes) to give **89** (0.2935 g, 43.6%) as an oil: ^{1}H NMR (400 MHz, $CDCl_3$) δ 7.82 (d, J = 9.2, 1H), 7.05 (s, 1H), 6.97 (s, 1H), 6.46 (d, J = 8.8, 1H), 6.01-5.91 (dddd, J = 17.2, 10.4, 5.6, 5.2, 1H), 5.31-5.26 (dd, J = 17.2, 1.6, 1H), 5.25-5.22 (dd, J = 10.4, 1.6, 1H), 4.22 (m, 2H), 3.83 (s, 3H), 3.58 (s, 2H), 2.05 (s, 3H), 1.56 (s, 6H), 1.21 (s, 6H); ^{13}C NMR (100.6 MHz, $CDCl_3$) δ 167.3, 151.7, 142.4, 142.1, 140.6, 133.5, 133.3, 131.1, 128.2, 125.5, 117.7, 111.6, 75.0, 70.7, 54.5, 51.5, 33.7, 29.8, 26.9, 17.7; ES-MS (M+Na)+ calcd for $C_{25}H_{31}NO_3Na$ 416.2202, found 416.2201.

4-(Methyl(1,1,4,4,7-pentamethylisochroman-6-yl)amino)benzoic acid (42). To a 100 mL round-bottomed flask equipped with a stir bar and charged with methyl ester **87** (0.3023 g, 0.8226 mmol) suspended in methanol (3.0 mL) was added a solution of potassium hydroxide (0.1707 g, 3.042 mmol) in water (0.26 mL). This reaction was stirred at reflux in an oil bath at 87 °C for 1.5h. The reaction was then cooled to r.t. and acidified with 1N HCl (85 mL) to give a precipitate. The precipitate was filtered and washed with copious amounts of cold water to give a crude product that was purified by column chromatography (25 mL SiO_2, 30% to 50% ethyl acetate:hexanes) to give pure **42** (0.2323 g, 79.9%) as a white crystalline solid, m.p. 273.3-275.0 °C: ^{1}H NMR (400 MHz, d6-DMSO) δ 12.16 (br s, 1H), 7.71 (d, J = 8.8, 2H), 7.16 (s, 1H), 7.12 (s, 1H), 6.43 (d, J = 8.4, 1H), 3.49 (s, 2H), 3.21 (s, 3H), 1.98 (s, 3H), 1.47 (s, 6H), 1.16 (s, 6H); ^{13}C NMR (100.6 MHz, d6-DMSO) δ 167.4, 151.9, 143.1, 142.0, 140.3, 132.9, 131.0, 128.2, 124.3, 117.8, 111.0, 74.5, 69.7, 33.4, 29.6, 26.7, 17.0; ES-MS (M+H)+ calcd for $C_{22}H_{27}NO_3Na$ 376.1889, found 376.1893.

4-(Ethyl(1,1,4,4,7-pentamethylisochroman-6-yl)amino)benzoic acid (43). To a 100 mL round-bottomed flask equipped with a stir bar and charged with methyl ester **88** (0.3178 g, 0.8330 mmol) suspended in methanol (2.6 mL) was added a solution of potassium hydroxide (0.1644 g, 2.93 mmol) in water (0.26 mL). This reaction was stirred at reflux in an oil bath at 87 °C for 1.5h. The reaction was then cooled to r.t. and acidified with 1N HCl (85 mL) to give a precipitate. The precipitate was filtered and washed with copious amounts of cold water to give a crude product that was purified by column chromatography (25 mL SiO_2, 30% to 50% ethyl acetate:hexanes) to give pure **43** (0.2562 g, 83.7%) as a white crystalline solid, m.p. 283.9-285.4 °C: ^{1}H NMR (400 MHz, d6-DMSO) δ 12.12 (br s, 1H), 7.69 (d, J = 9.2, 2H), 7.18 (s, 1H), 7.07 (s, 1H), 6.40 (d, J = 8.8, 1H), 3.63 (br s, 2H), 3.50 (s, 2H), 1.98 (s, 3H), 1.47 (s, 6H), 1.16 (s, 6H) 1.14 (t, J = 7.2, 3H); ^{13}C NMR (100.6 MHz, d6-DMSO) δ 167.4, 151.1, 141.9, 141.3, 140.4, 133.4, 131.2, 128.3, 125.5, 117.4, 110.8, 74.5, 69.7, 45.6, 33.3, 29.6, 26.7, 17.1, 12.3; ES-MS (M+H)+ calcd for $C_{23}H_{30}NO_3$ 368.2226, found 368.2209.

4-(Allyl(1,1,4,4,7-pentamethylisochroman-6-yl)amino)benzoic acid (44). To a 100 mL round-bottomed flask equipped with a stir bar and charged with methyl ester **89** (0.2611 g, 0.6635 mmol) suspended in methanol (2.6 mL) was added a solution of potassium hydroxide (0.1927 g, 3.43 mmol) in water (0.26 mL). This reaction was stirred at reflux in an oil bath at 87 °C for 1.5h. The reaction was then cooled to r.t. and acidified with 1N HCl (90 mL) to give a precipitate. The precipitate was filtered and washed with copious amounts of cold water to give a crude product that was purified by column chromatography (25 mL SiO_2, 30% to 50% ethyl acetate:hexanes) to gi: ve pure **44** (0.1936 g, 76.9%) as a white crystalline solid, m.p. 244.5-246.5 °C: ^{1}H NMR (400 MHz, d6-DMSO) δ 12.17 (br s, 1H), 7.69 (d, J = 8.8, 2H), 7.18 (s, 1H), 7.11 (s, 1H), 6.42 (d, J = 8.8, 1H), 5.99-5.90 (dddd, J = 16.8, 10.4, 5.2, 5.2, 1H), 5.31-5.26 (d, J = 17.2, 1.6, 1H), 5.21-5.18 (d, J = 10.4, 1.6, 1H), 4.23 (br s, 2H), 3.49 (s, 2H), 1.99 (s, 3H), 1.47 (s, 6H), 1.15 (s, 6H); ^{13}C NMR (100.6 MHz, d6-DMSO) δ 167.3, 151.2, 142.0, 141.8, 140.4, 133.8, 133.1, 128.3, 125.1, 118.0, 117.0, 111.4, 74.5, 69.7, 54.1, 33.3, 29.6, 26.7, 17.3; ES-MS (M+H)+ calcd for $C_{24}H_{30}NO_3$ 380.2226, found 380.2225.

References

1. Tachdjian, C.G., J.; Boudjelal, M.; Al-Shamma, H.A.; Giachino, A. F.; Jakubowicz-Jaillardon, K.; Chen, Q.; Zapf, J.W.; Pfahl, M., *Preparation of substituted isochroman compounds for the treatment of metabolic disorders, cancer and other diseases*, P.I. Appl, Editor. 2004.
2. Faul, M.M., et al., *Synthesis of Novel Retinoid X Receptor-Selective Retinoids.* J. Org. Chem., 2001. **66**(17): p. 5772-5782.
3. Jurutka, P.W., et al., *Modeling, Synthesis, and Biological Evaluation of Potential Retinoid X Receptor (RXR) Selective Agonists: Novel Analogues of 4-[1-(3,5,5,8,8-Pentamethyl-5,6,7,8-tetrahydro-2-naphthyl)ethynyl]benzoic Acid (Bexarotene) and (E)-3-(3-(1,2,3,4-tetrahydro-1,1,4,4,6-pentamethylnaphthalen-7-yl)-4-hydroxyphenyl)acrylic Acid (CD3254).* J. Med. Chem., 2013. **56**(21): p. 8432-8454.

4. Heck, M.C., et al., *Modeling, Synthesis, and Biological Evaluation of Potential Retinoid X Receptor (RXR)-Selective Agonists: Analogues of 4-[1-(3,5,5,8,8-Pentamethyl-5,6,7,8-tetrahydro-2-naphthyl)ethynyl]benzoic Acid (Bexarotene) and 6-(Ethyl(5,5,8,8-tetrahydronaphthalen-2-yl)amino)nicotinic Acid (NEt-TMN).* J. Med. Chem., 2016. **59**(19): p. 8924-8940.

File: **C:\MASSLYNX\JULY 21.PRO\DATA\210826_CW-VI-171_POS.RAW**

Printed: **Friday, August 27, 2021 09:30:35 Pacific Daylight Time**

Header
Acquired File Name: 210826_CW-VI-171_pos
Acquired Date: 26-Aug-2021
Acquired Time: 16:52:17
Job Code: July21
Task Code:
User Name:
Laboratory Name:
Instrument: ACQ-QDA#KAD3226
Conditions:
Submitter:
SampleID:
Bottle Number: 2:25
Description: mz not observed

Instrument Calibration:
Calibration File: C:\MassLynx\IntelliStart.pro\AcquDB\AutoCal.cal
Parameters
MS1 Static:
Mass: 30 Da to 1250 Da.
Resolution: -0.1/-0.0
Ion Energy: -0.3
Reference File: Internal
Acquisition File:
MS1 Scanning:
Mass: 30 Da to 1250 Da.
Resolution: -0.1/-0.0
Ion Energy: -0.3
Reference File: Internal
Acquisition File:
MS1 Scan Speed Compensation:
Scan: 40 to 10000 amu/sec.
Resolution: -0.1/-0.0
Ion Energy: -0.3
Reference File: Internal
Acquisition File:
Calibration Time: 10:54
Calibration Date: 09/13/17
Coefficients
MS1 Static: -0.000000000000*x^4 + 0.000000000656*x^3 + -0.000001077025*x^2 + 1.000599988917*x +-0.085640798905
Function 1: -0.000000000000*x^4 + 0.000000000378*x^3 + -0.000000745481*x^2 + 1.000452684494*x +-0.068499345191

Function 2: None

Parameters for C:\MassLynx\July 21.PRO\ACQUDB\POS_105-600_C_5mins.EXP

Prescan Statistics:

Initial Average Intensity	379.7540
Initial Average Std Dev	1.1398
Bunch Zero Level	0.0000
Bunch Std Dev	0.0000
Bunch Threshold	0.0000
Spike Removal Std Dev	1.1211
Ion Count Threshold:	25.0000

Data Processing:

Targeted Sampling Frequency	2
Actual Sampling Frequency	2.000
SIR Chromatogram Spike Removal	ON
SIR Smoothing	ON
Smoothing window size (scans)	3
Number of smooths	2

Method Events:

Initial Stop Flow: No Change

Initial Switch 1: No Change

Timed Events Enabled

Event	Time(Sec)	Name	Action

Instrument Parameters - Function 1:

Polarity	ES+
Calibration	Dynamic 2
Capillary (kV)	1.50
Cone (V)	10.00
Source Temperature (°C)	120
Probe Temperature (°C)	450
Calibration Temperature (°C)	0

Engineers Settings:

File: **C:\MASSLYNX\JULY 21.PRO\DATA\210826_CW-VI-171_POS.RAW**

Printed: **Friday, August 27, 2021 09:30:35 Pacific Daylight Time**

```
LM 1 Resolution         0.00
HM 1 Resolution         0.00
Low Mass Ion Energy 1   -0.91
High Mass Ion Energy 1  0.14
Low Mass Position       0.69
High Mass Position      -1.81
Low Mass Setup          0.63
High Mass Setup         -6.13
Detector Gain Positive  1.0
Detector Gain Negative  1.0
Nominal Rod Polarity    Negative
Dynamic Offset Positive Settings:
Dynamic Offset Low Mass Resolution      0.00
Dynamic Offset High Mass Resolution     0.00
Dynamic Offset Low Mass Ion Energy      0.00
Dynamic Offset High Mass Ion Energy     -0.02

Dynamic Offset Negative Settings:
Dynamic Offset Low Mass Resolution      -0.01
Dynamic Offset High Mass Resolution     0.21
Dynamic Offset Low Mass Ion Energy      0.00
Dynamic Offset High Mass Ion Energy     0.19

Dynamic Offset Settings:
Dynamic Offset Low Mass Position        -0.71
Dynamic Offset High Mass Position       -0.46
Dynamic Offset Low Mass Setup   0.55
Dynamic Offset High Mass Setup  -0.04
Dynamic Offset Linearity Gain   4.80


Set Detector Gain       1.0

Instrument Readbacks
Capillary (kV)                  1.50
Cone (V)                        14.26
Source Temperature (°C)         120
Multiplier              375
Probe Temperature (°C)          450
Calibration Temperature (°C)    29
```

Inter-scan delays:
Automatic Mode
MS Delay Table

	R	delay
<=	16.000	0.005
>	16.000	0.006

Health Check Failures:
All Health Checks Passed

Health Checks all enabled unless shown below:
Resolution Setup Required
Recalibration Required
Service Due

ACE Experimental Record

Inlet Method File: c:\masslynx\july 21.pro\acqudb\col4_pfp_fa_acn_5mins

--------------------- Run method parameters ----------------

Waters ACQUITY QSM
Solvent A Name: Water
Solvent B Name: Methanol
Solvent C Name: 0.1% FA
Solvent D Name: Acetonitrile
Low Pressure Limit: 0 psi
High Pressure Limit: 15000 psi
Seal Wash Period: 5.00 min
[Gradient Table]
Time(min) Flow Rate(mL/min) %A %B %C %D Curve
1. Initial 0.500 0.0 0.0 95.0 5.0 Initial
2. 2.00 0.500 0.0 0.0 5.0 95.0 6
3. 3.50 0.500 0.0 0.0 95.0 5.0 11
4. 5.00 0.500 0.0 0.0 95.0 5.0 11

Comment:
Flow Ramp Rate: 0.45 min
D Solvent Selection (if supported): No Change
System Pressure Data Channel: No
Flow Rate Data Channel: No
%A Data Channel: No
%B Data Channel: No
%C Data Channel: No
%D Data Channel: No
Primary Data Channel: No
Accumulator Data Channel: No
Degasser Data Channel: No
Gradient Start: At Injection
Gradient Start Volume: 0 uL
Gradient Start Time: 0.00 min
Participate in pre-analysis: No

Waters Acquity CM

Target Column Temperature: 50.0 C
Temperature Alarm Band: 5.0 C
: No
Column Valve Position: Column 4
Equilibration Time: 0.1 min
External Valve 1: No Change
External Valve 2: No Change
External Valve 3: No Change
Comment:
Column Temperature Data Channel: No
Preheater Temperature Data Channel: No

Waters Acquity PDA

Run Time: 5.00 min
PDA Detector Type: UPLC LG 500 nm
Lamp: On
Sampling Rate: 20 points/sec
Filter Time Constant: 0.2000 sec
Exposure Time: Auto msec
Interpolate 2nd order filter Region: No
Use UV Blocking Filter: Yes
3D Channel...
Range: 210 - 499
Resolution: 2.4 nm
Channel 1...
Data Mode: Absorbance at 214
Resolution: 2.4 nm
Channel 2...
Data Mode: Absorbance at 260
Resolution: 2.4 nm
Channel 3...
Data Mode: Absorbance at 350
Resolution: 2.4 nm
Initial Switch 1: No Change
Initial Switch 2: No Change

Waters ACQUITY FTN AutoSampler
Run Time: 5.00 min
Comment:
Load Ahead: Disabled
Loop Offline: Automatic min
Wash Solvent Name: Weak Wash
Pre-Inject Wash Time: 0.0 sec
Post-Inject Wash Time: 6.0 sec
Purge Solvent Name: Strong Wash
Dilution: Disabled
Dilution Volume: 0 uL
Delay Time: 0 min
Dilution Needle Placement: Automatic mm
Target Column Temperature: Off C

Target Sample Temperature: 10.0 C
Sample Temperature Alarm Band: Disabled
Syringe Draw Rate: Automatic
Needle Placement: 2.0 mm
Pre-Aspirate Air Gap: Automatic
Post-Aspirate Air Gap: Automatic
Column Temperature Data Channel: No
Room Temperature Data Channel: No
Sample Temperature Data Channel: No
Sample Organizer Temperature Data Channel: No
Sample Pressure Data Channel: No
Preheater Temperature Data Channel: No
Seal Force Data Channel: No
No Injection Mode Enabled: No
Autoaddition Mix Stroke Cycles: Automatic
Autoaddition Mix Stroke Volume: Automatic uL
Run Events: Yes

Sample Run Injection Parameter

Injection Volume (ul) - 2.00

---------------------------- oOo -----------------------------

End of experimental record.

------------------- Waters ACQUITY QSM Postrun Report ---------------

Firmware Version: 1.60.260 (Jul 9 2013)
Software Version: 1.60.1897
Checksum: 0xa9c8e138
Serial Number: D17QSM962A
Minimum System Pressure: 2958.0 psi
Maximum System Pressure: 5886.0 psi
Average System Pressure: 4651.0 psi

---------------------------- oOo ----------------------------

------------------- Waters ACQUITY FTN Postrun Report ---------------

Software Version: 1.60.1774
Firmware Version: 1.60.364 (Sep 20 2013)
Checksum: 0x35d5392b
Serial Number: D17SDI835G
Sample Syringe Size: 100.0
Extension Loop Size: 0.0
Needle Size: 30.0
Minimum Sample Temperature: 9.9
Maximum Sample Temperature: 10.2
Average Sample Temperature: 10.1
Minimum Column Temperature: -0.2
Maximum Column Temperature: 0.0
Average Column Temperature: -0.2

---------------------------- oOo ----------------------------

------------------- Waters Acquity CM Postrun Report ---------------

Software Version: 1.60.2072
Firmware Version: 1.65.142 (Apr 02 2015)
Checksum: 0x18bfb4ea
Serial Number: D17CMP604G
Valve Position: 4
Minimum Column Temperature: 50.0
Maximum Column Temperature: 50.0
Average Column Temperature: 50.0

---------------------------- oOo ----------------------------

------------------- Active eCord Data ---------------

Valve Current Position: 4
Failed to retrive Active eCord ColumnData Data

---------------------------- oOo -----------------------------

------------------- Generic Instrument Postrun Report ---------------

Software Version: 1.60.1390
Firmware Version: 1.60.6169 (Aug 13 2013)
Checksum: 0xdcfe9340
Serial Number: B17UPD132A
Lamp On/Off Event: No
Lamp Life: 3230.00 hours
Lamp Serial Number: 000133684
Exposure Time: 50.000 msec
Lambda1: 187.360
Lambda512: 502.647
Flow Cell Type: Analytical LG
Flow Cell Path Length: 10.000 mm
Flow Cell Volume: 0.500 microliters
Flow Cell Serial Number: PDA10-15625
Flow Cell Part Number: 205015017
Optics Temperature Stabilzation Setting: Normal Temperature

---------------------------- oOo -----------------------------

---------------Failed to get IECordHost2 Interface ---------

Function 1

Scans in function:	601
Cycle time (secs):	Automatic
Scan duration (secs):	0.495

File: **C:\MASSLYNX\JULY 21.PRO\DATA\210826_CW-VI-171_POS.RAW**

Printed: **Friday, August 27, 2021 09:30:35 Pacific Daylight Time**

Inter Scan Delay (secs): Automatic
Start and End Time(mins): 0.000 to 5.000
Ionization mode: ES+
Data type: Accurate Mass
Function type: Scan
Mass range: 105 to 600

Function 2
Scans in function: 6001
Function type: Diode Array
Wavelength range (nm): 210 to 499

mz not observed

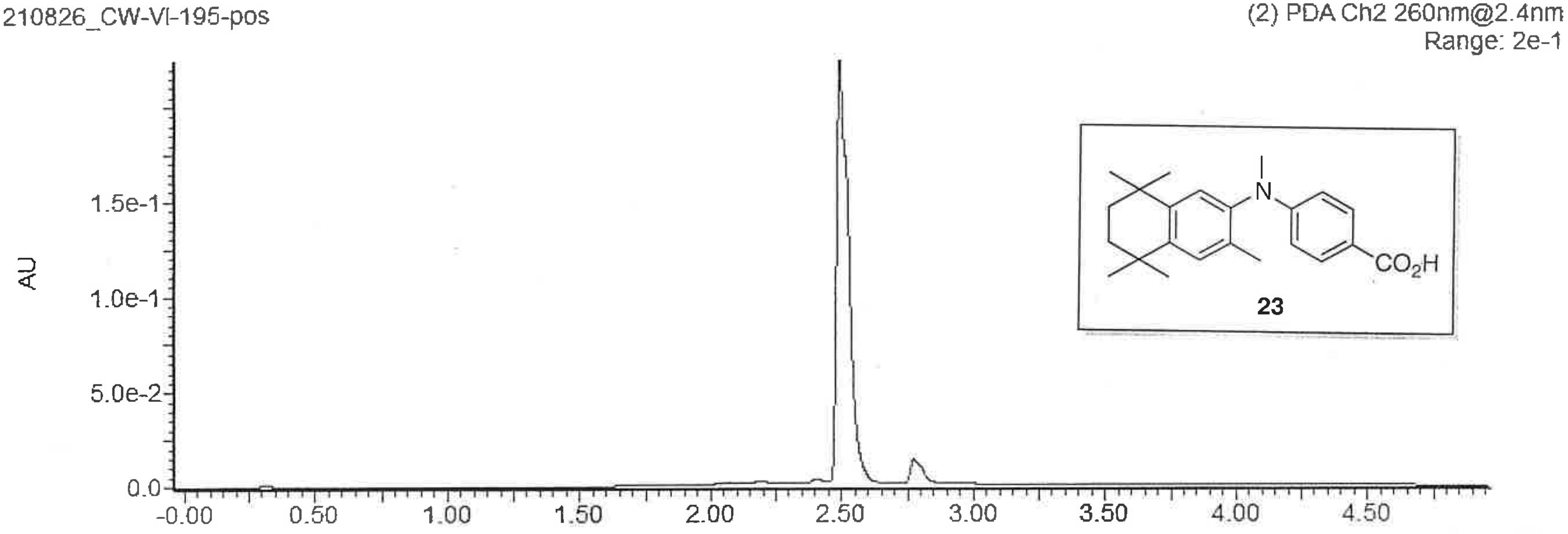

23

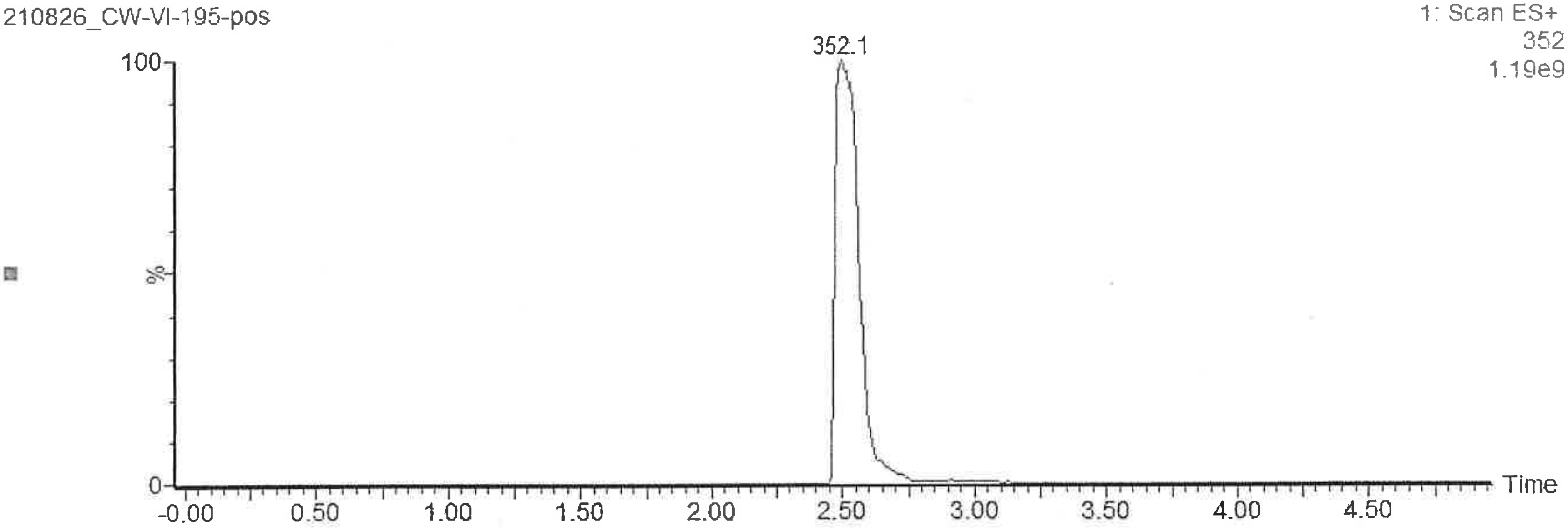

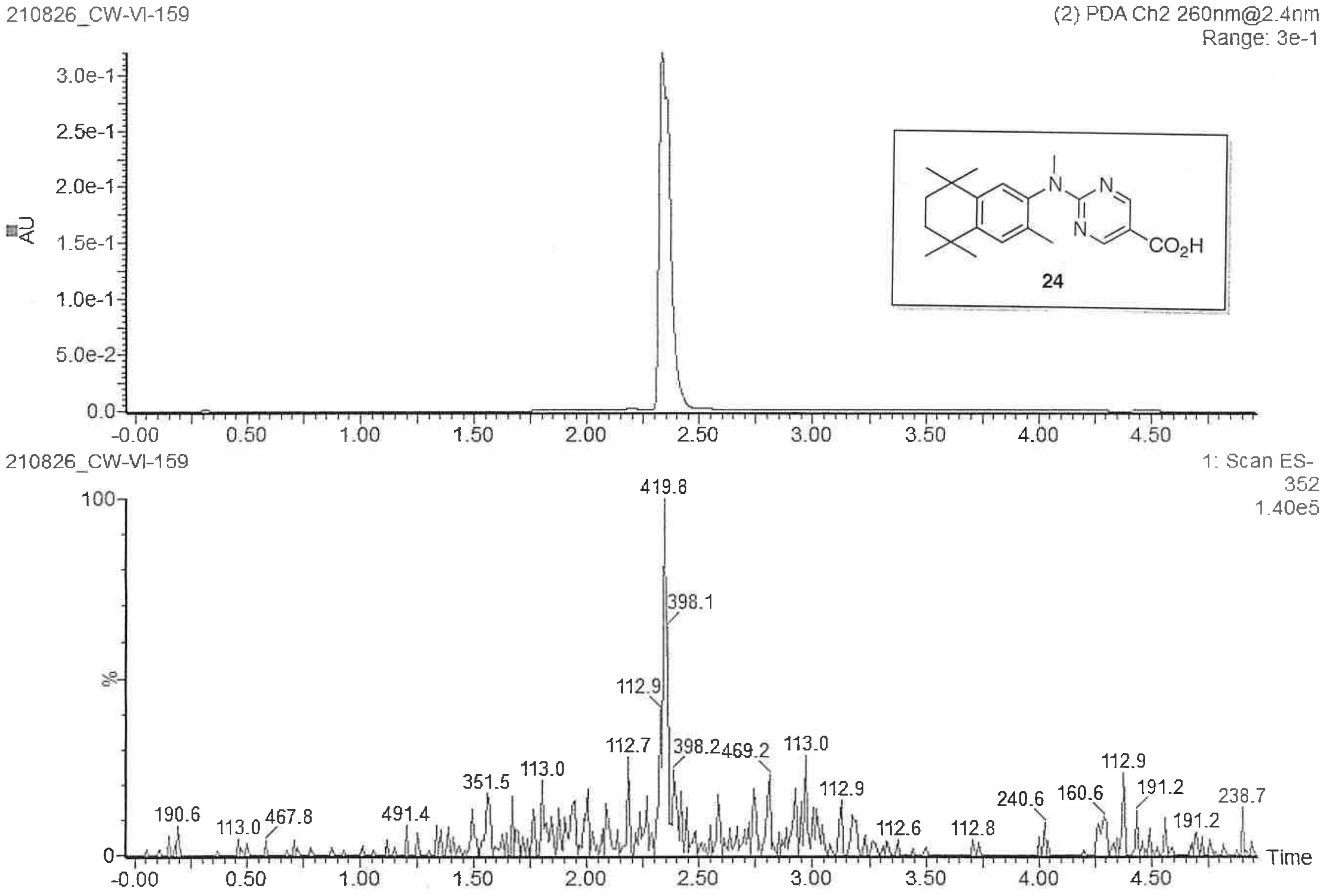
210826_CW-VI-159
(2) PDA Ch2 260nm@2.4nm
Range: 3e-1
AU
3.0e-1
2.5e-1
2.0e-1
1.5e-1
1.0e-1
5.0e-2
0.0
N
N
N
CO2H
24
-0.00
0.50
1.00
1.50
2.00
2.50
3.00
3.50
4.00
4.50
210826_CW-VI-159
1: Scan ES-
352
1.40e5
100
%
0
419.8
398.1
112.9
112.7
398.2
469.2
113.0
190.6
113.0
467.8
491.4
351.5
113.0
112.9
112.6
112.8
240.6
160.6
112.9
191.2
191.2
238.7
Time

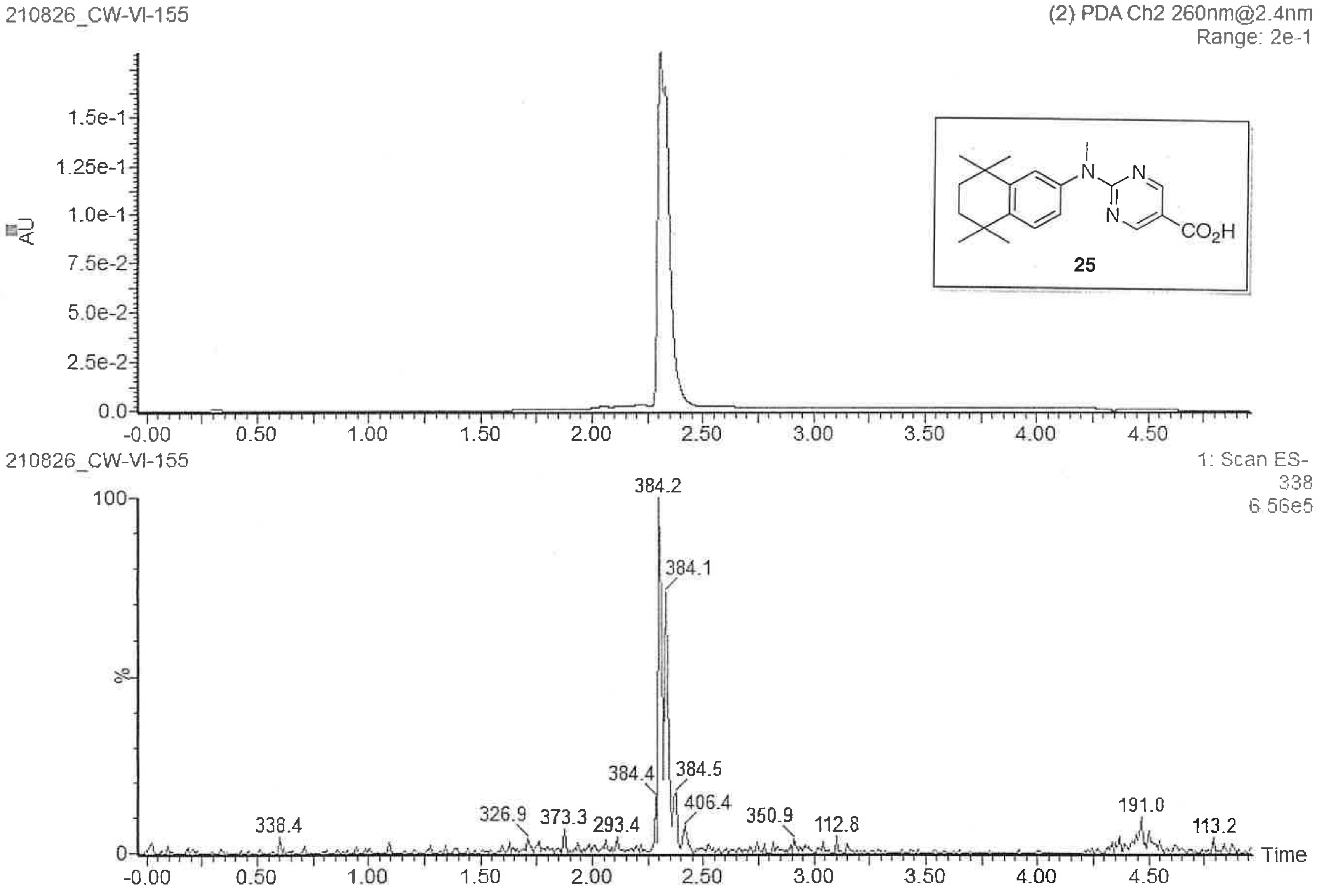
210826_CW-VI-155
(2) PDA Ch2 260nm@2.4nm
Range: 2e-1
AU
1.5e-1
1.25e-1
1.0e-1
7.5e-2
5.0e-2
2.5e-2
0.0
-0.00
0.50
1.00
1.50
2.00
2.50
3.00
3.50
4.00
4.50
N
N
N
CO_2H
25
210826_CW-VI-155
1: Scan ES-
338
6.56e5
100
%
0
384.2
384.1
384.4
384.5
406.4
338.4
326.9
373.3
293.4
350.9
112.8
191.0
113.2
Time

mz not observed

210826_CW-VI-141_pos

(2) PDA Ch2 260nm@2.4nm
Range: 6e-1

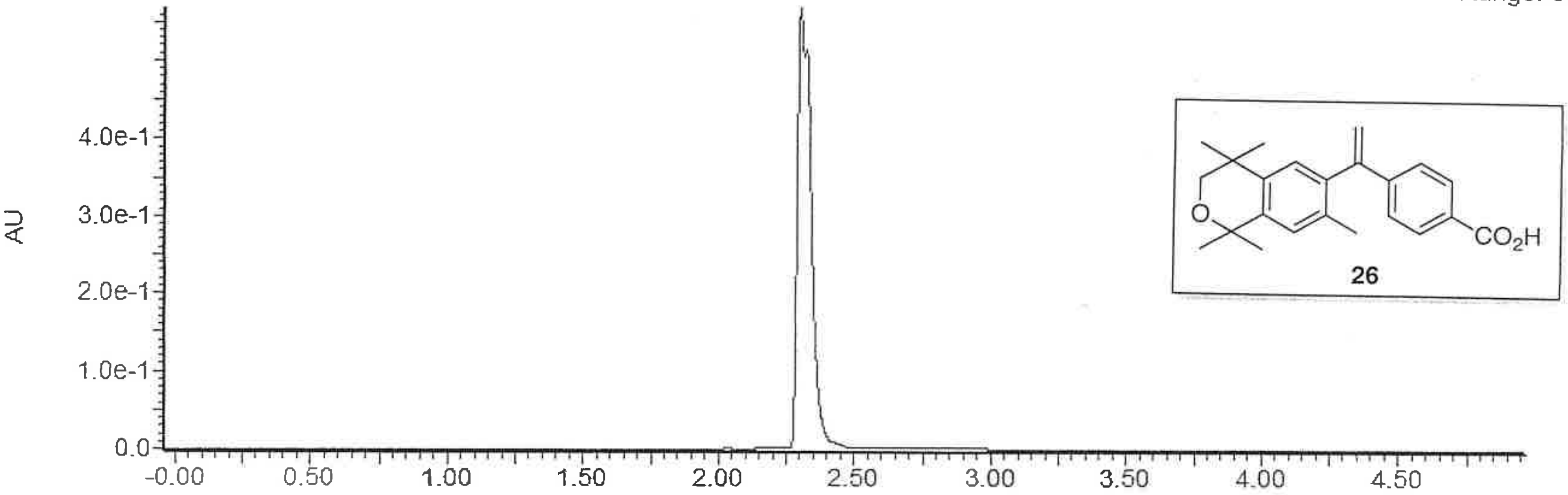

26

210826_CW-VI-141_pos

1: Scan ES+
351
6.51e7

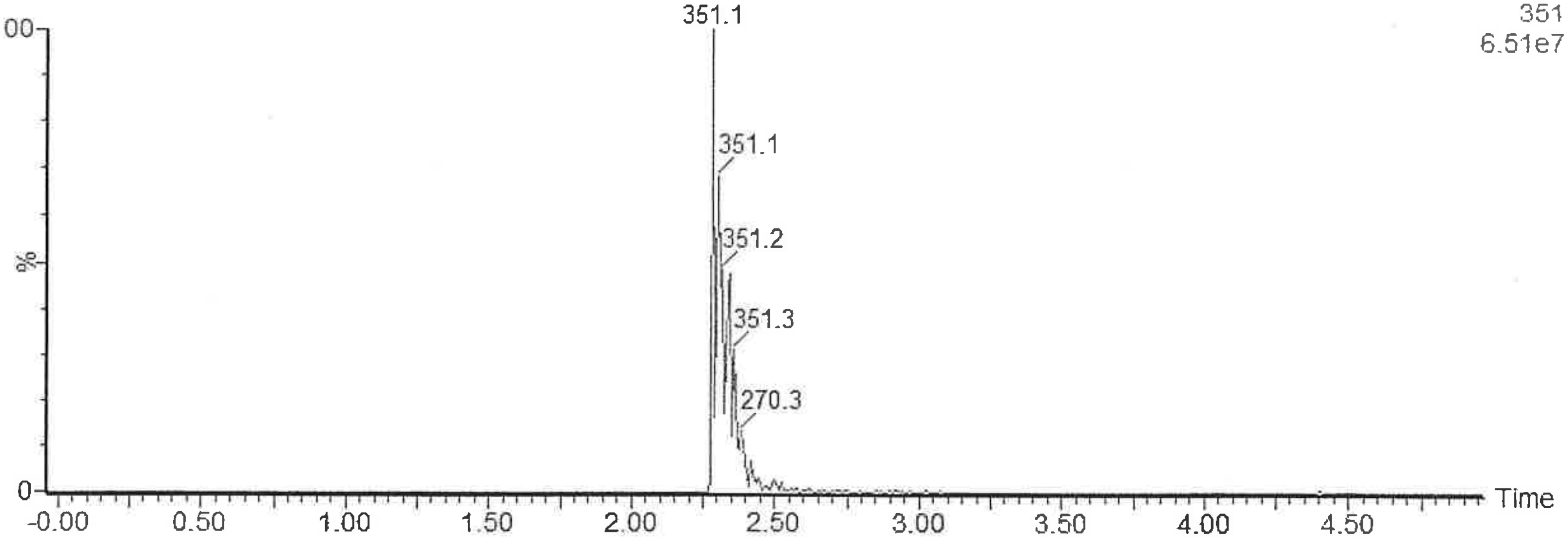

Br @ 489/491

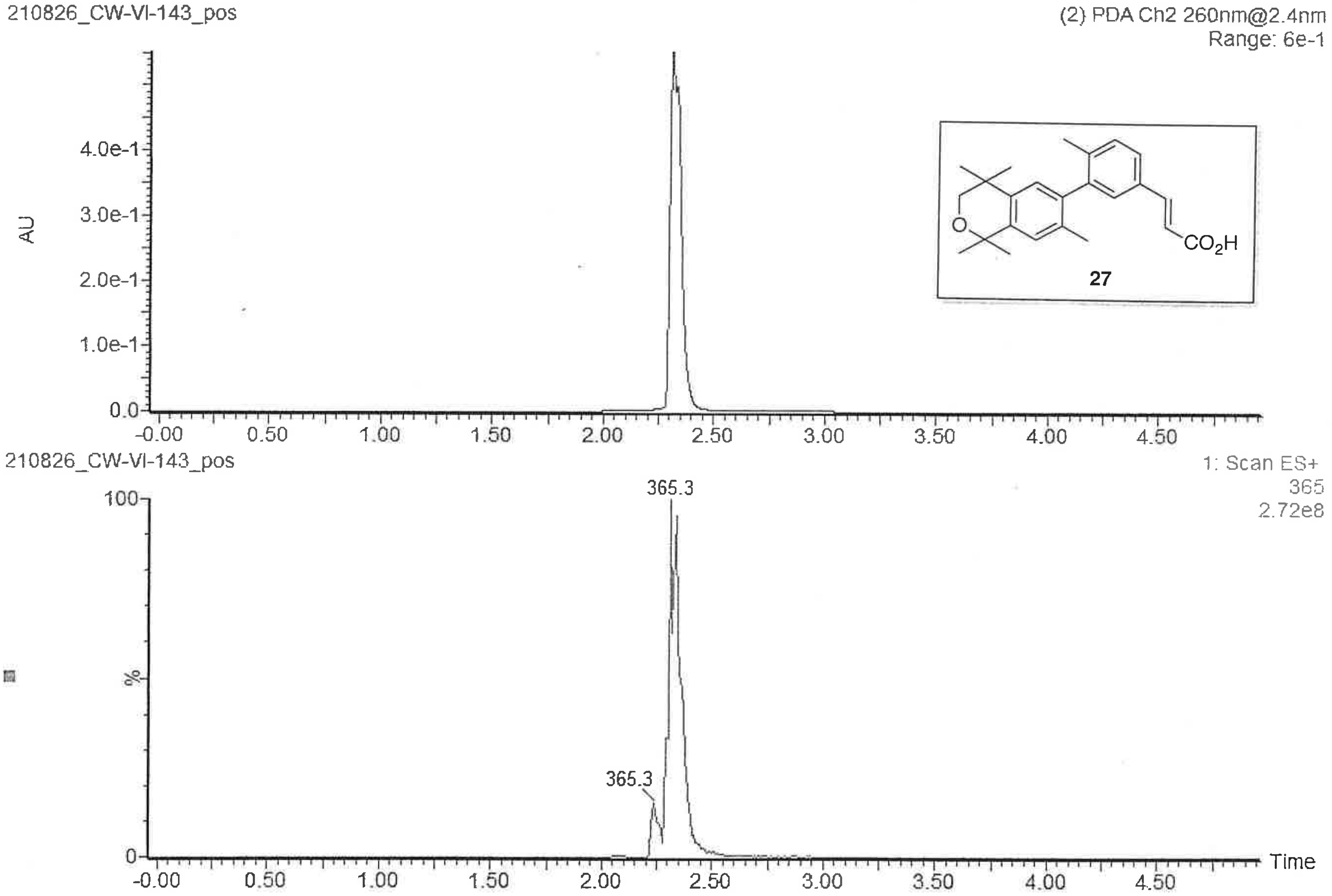
210826_CW-VI-143_pos
(2) PDA Ch2 260nm@2.4nm
Range: 6e-1
AU
4.0e-1
3.0e-1
2.0e-1
1.0e-1
0.0
CO_2H
27
-0.00
0.50
1.00
1.50
2.00
2.50
3.00
3.50
4.00
4.50
210826_CW-VI-143_pos
1: Scan ES+
365
2.72e8
100
%
0
365.3
365.3
Time

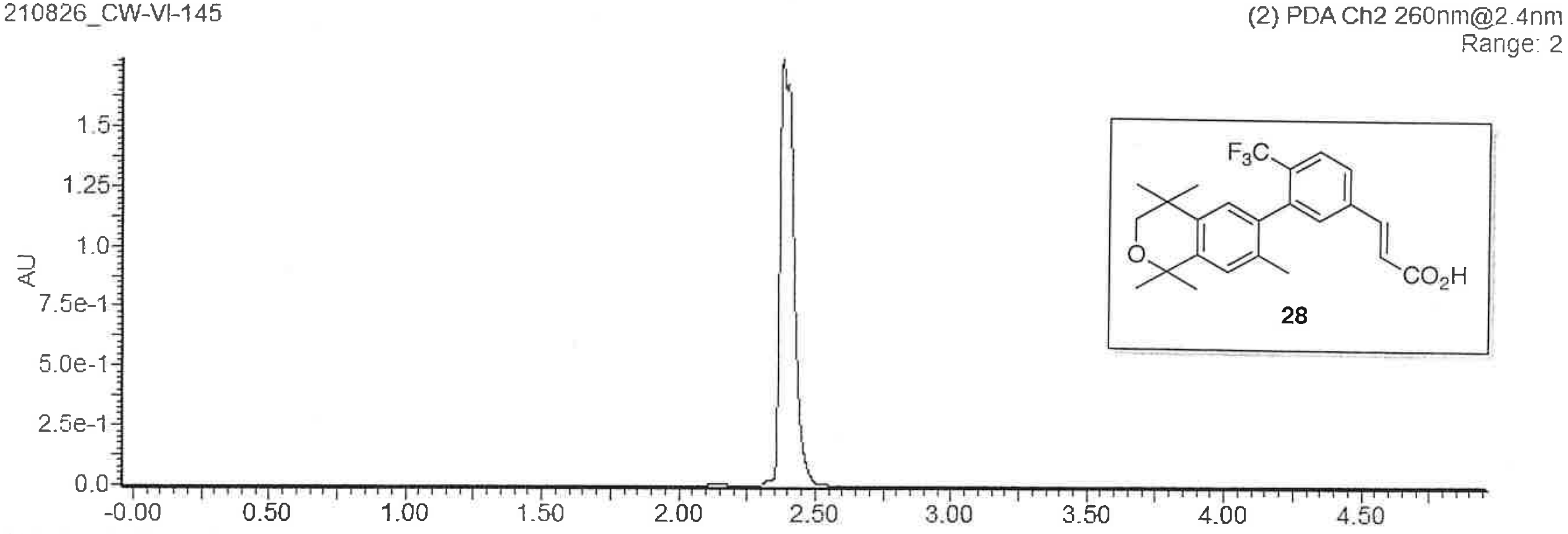

210826_CW-VI-145
(2) PDA Ch2 260nm@2.4nm
Range: 2
AU
1.5
1.25
1.0
7.5e-1
5.0e-1
2.5e-1
0.0
-0.00
0.50
1.00
1.50
2.00
2.50
3.00
3.50
4.00
4.50
F3C
CO2H
O

28

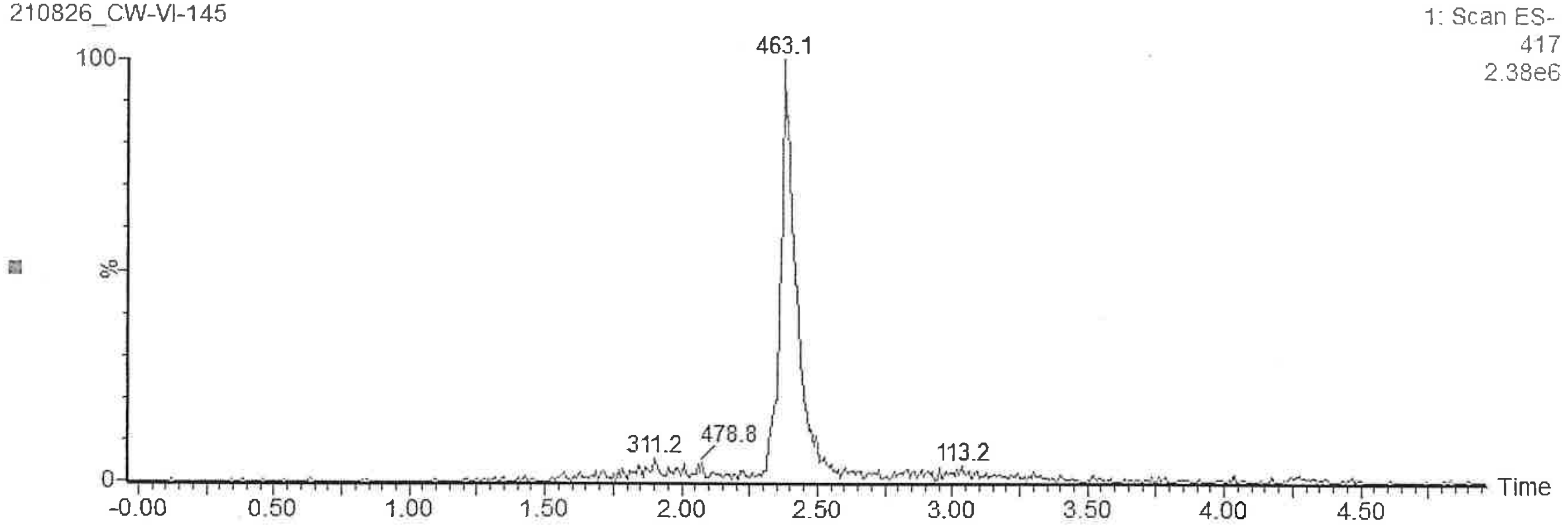

210826_CW-VI-145
1: Scan ES-
417
2.38e6
100
%
0
463.1
311.2
478.8
113.2
-0.00
0.50
1.00
1.50
2.00
2.50
3.00
3.50
4.00
4.50
Time

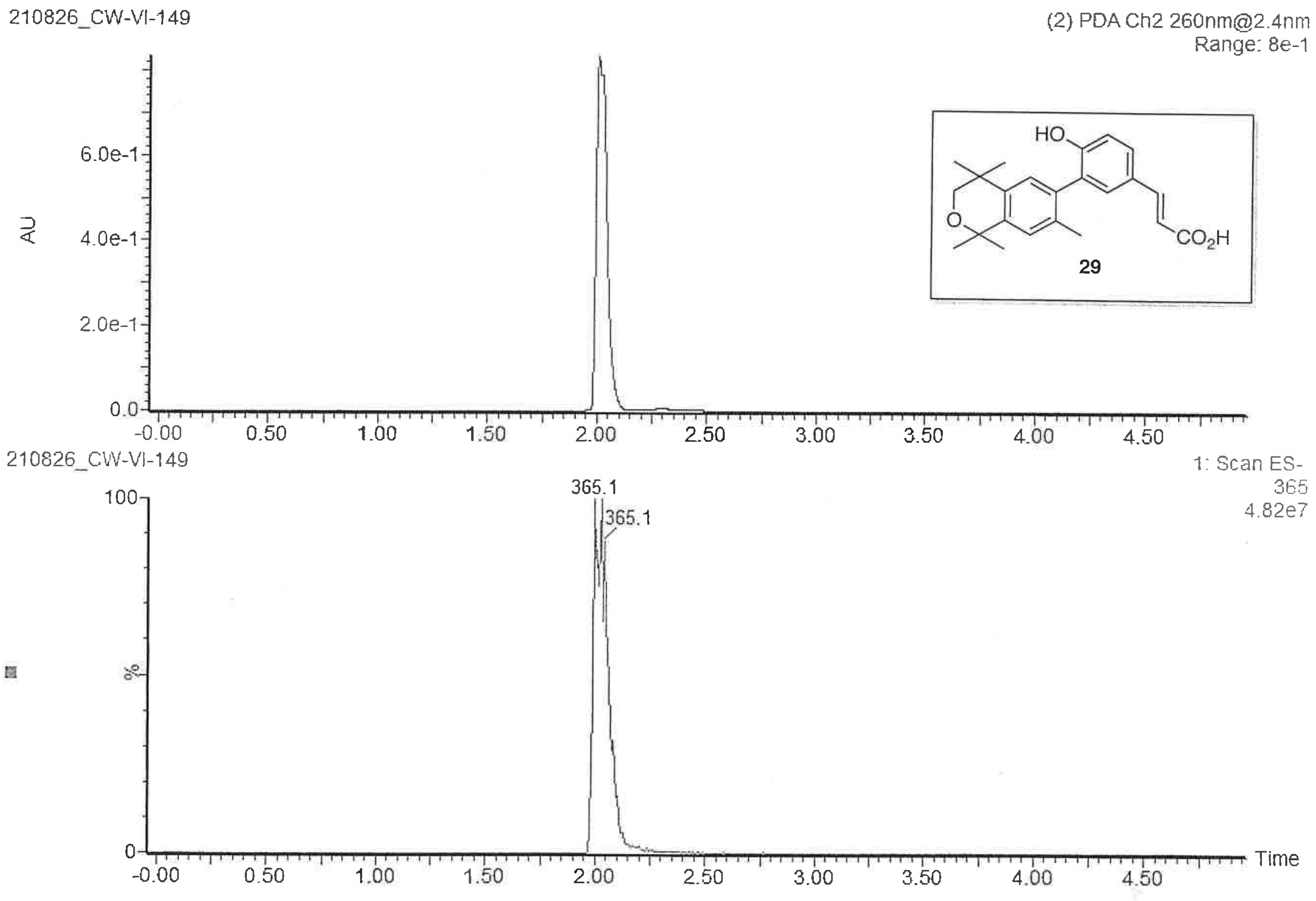
210826_CW-VI-149
(2) PDA Ch2 260nm@2.4nm
Range: 8e-1
AU
6.0e-1
4.0e-1
2.0e-1
0.0
-0.00
0.50
1.00
1.50
2.00
2.50
3.00
3.50
4.00
4.50
HO
O
CO2H
29
210826_CW-VI-149
1: Scan ES-
365
4.82e7
365.1
365.1
100
%
0
Time

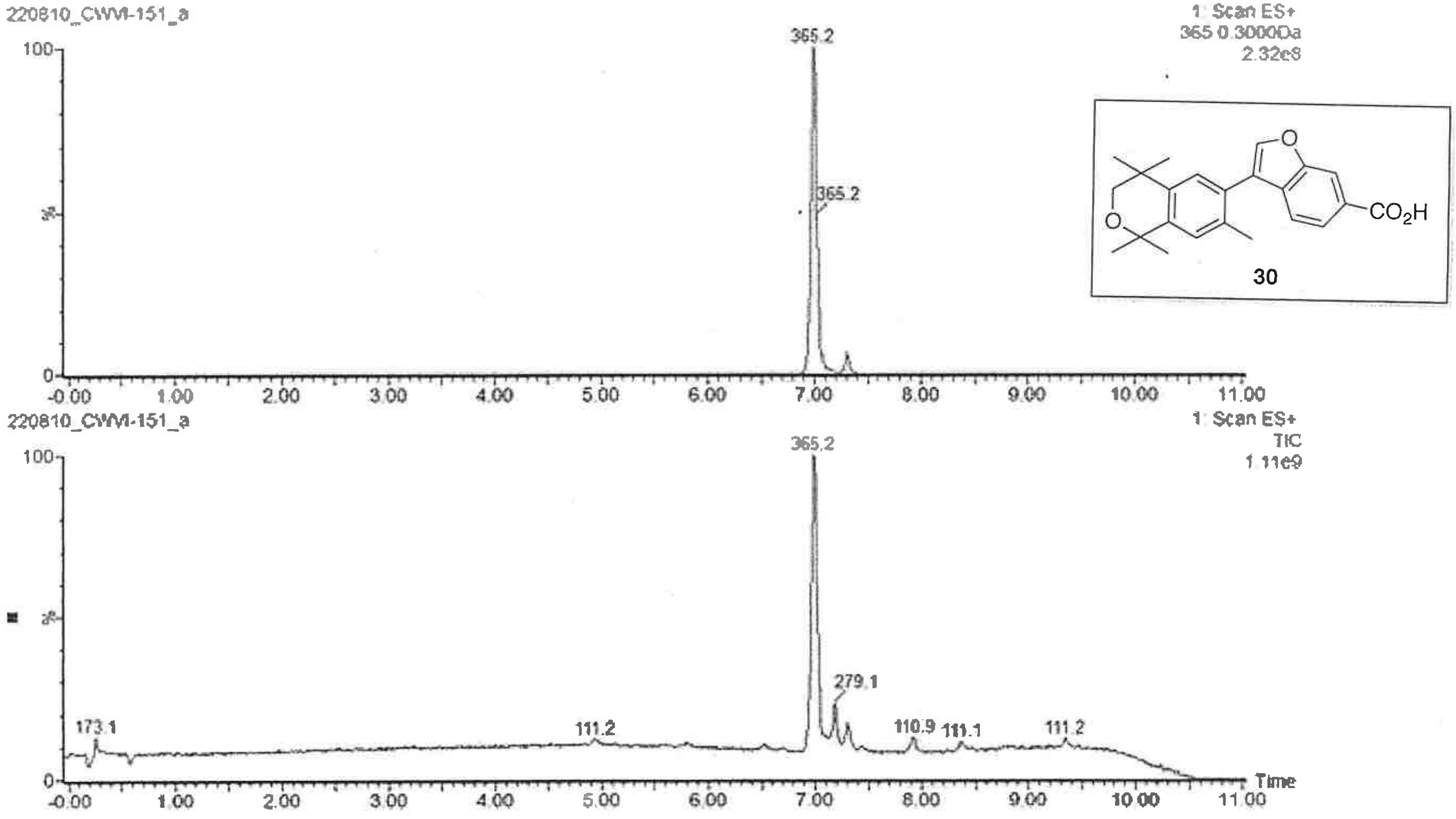

220810_CWM-151_a
1: Scan ES+
365 0.3000Da
2.32e8
100
%
0
365.2
365.2
-0.00
1.00
2.00
3.00
4.00
5.00
6.00
7.00
8.00
9.00
10.00
11.00
CO_2H
30
220810_CWM-151_a
1: Scan ES+
TIC
1.11e9
100
%
0
365.2
279.1
173.1
111.2
110.9
111.1
111.2
Time

mz not observed

210826_CW-VI-153_pos

(2) PDA Ch2 260nm@2.4nm
Range: 3e-1

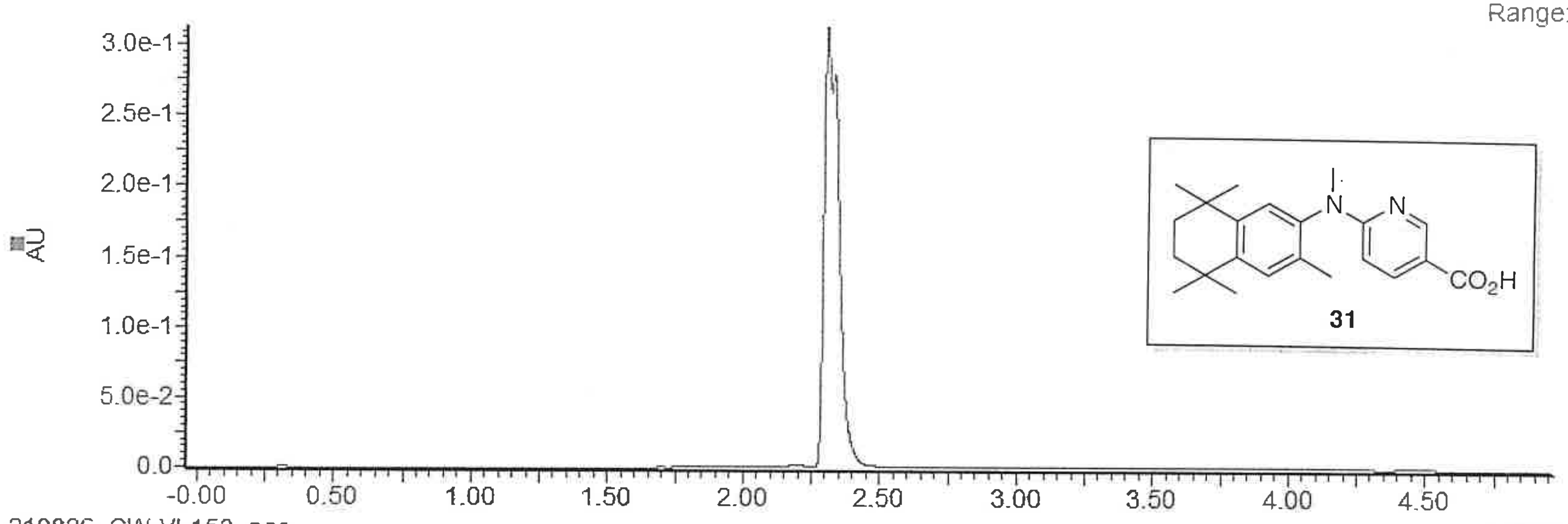

210826_CW-VI-153_pos

1: Scan ES+
353
1.33e9

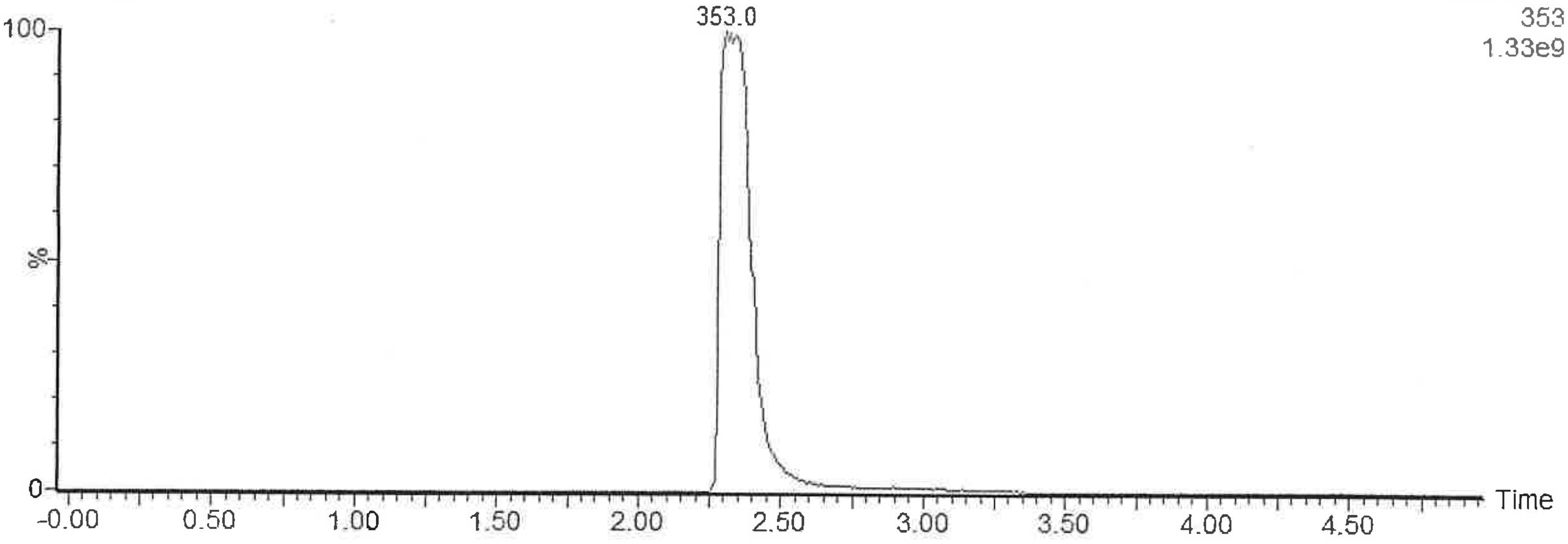

mz not observed

210826_CW-VI-173-pos

(2) PDA Ch2 260nm@2.4nm
Range: 5e-1

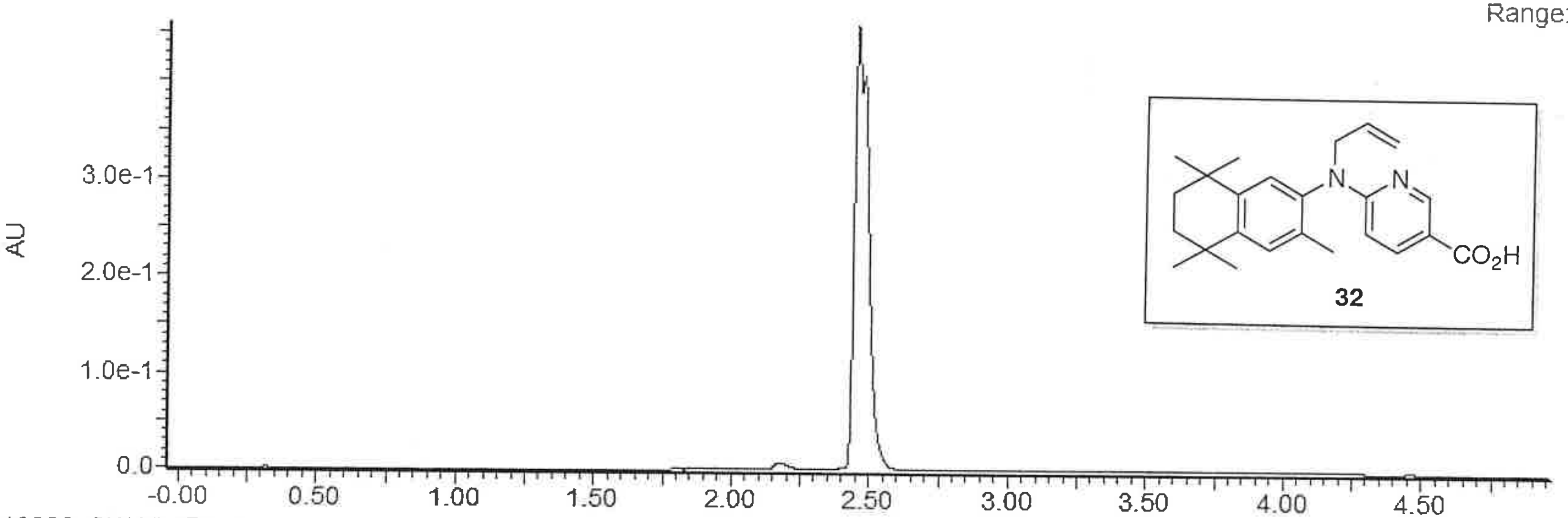

32

210826_CW-VI-173-pos

1: Scan ES+
379
1.35e9

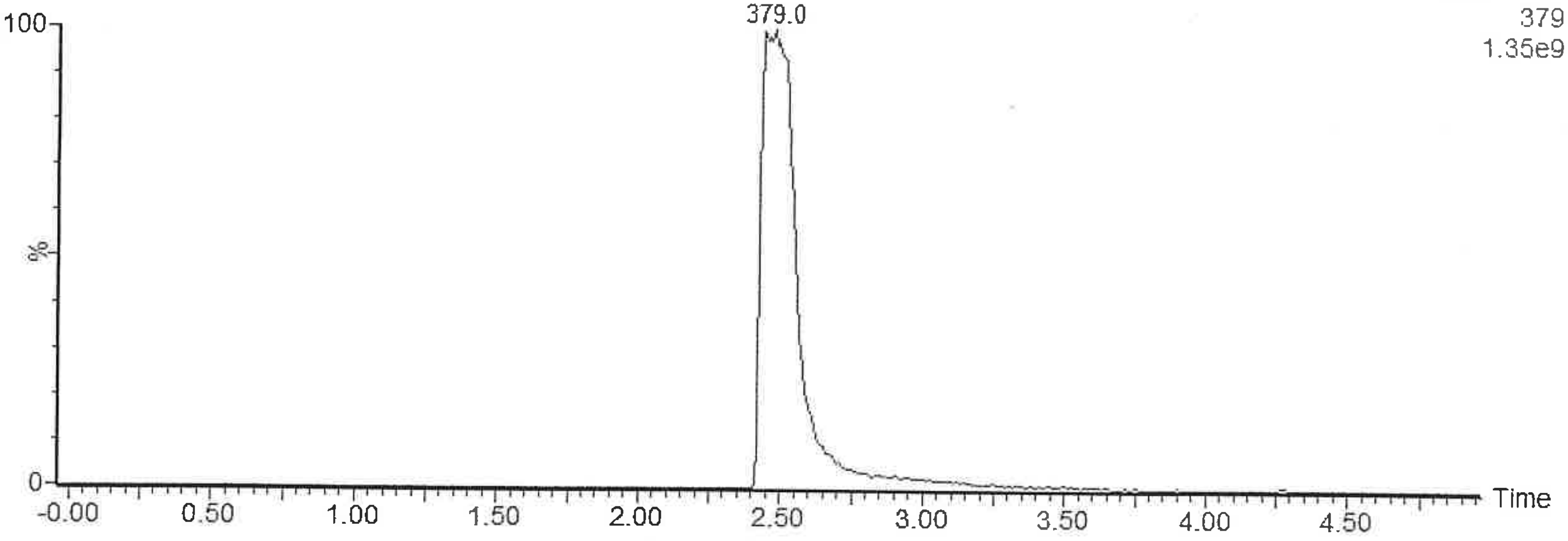

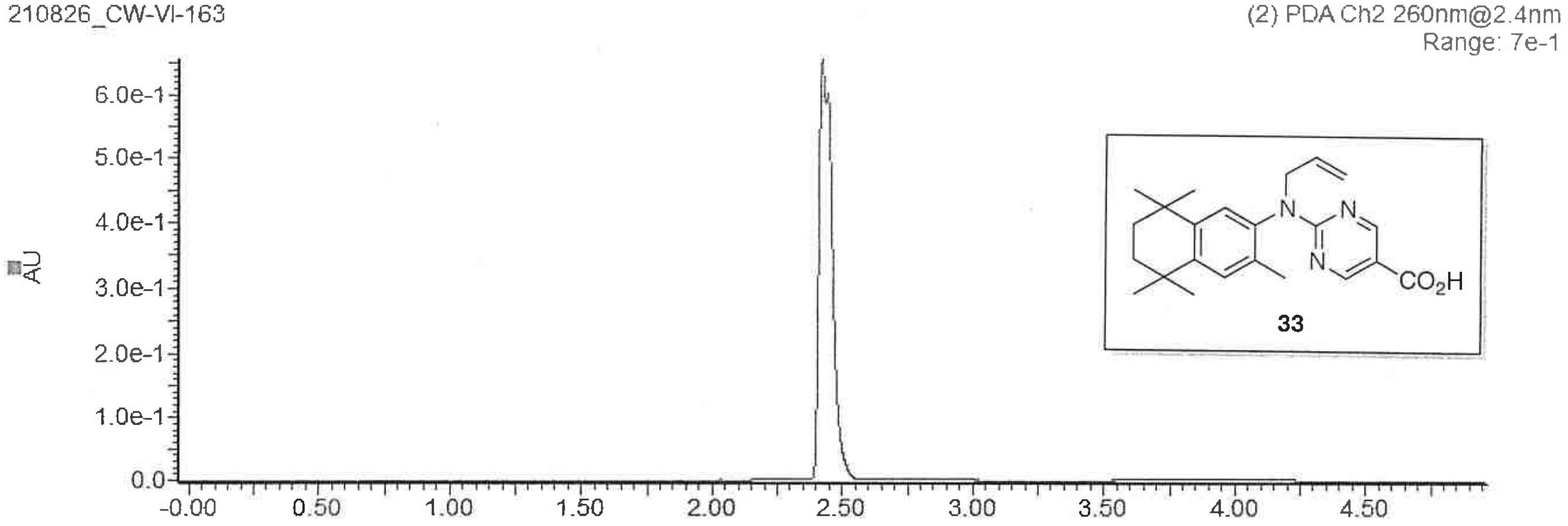
210826_CW-VI-163
(2) PDA Ch2 260nm@2.4nm
Range: 7e-1
AU
6.0e-1
5.0e-1
4.0e-1
3.0e-1
2.0e-1
1.0e-1
0.0
-0.00
0.50
1.00
1.50
2.00
2.50
3.00
3.50
4.00
4.50
N
N
N
CO_2H

33

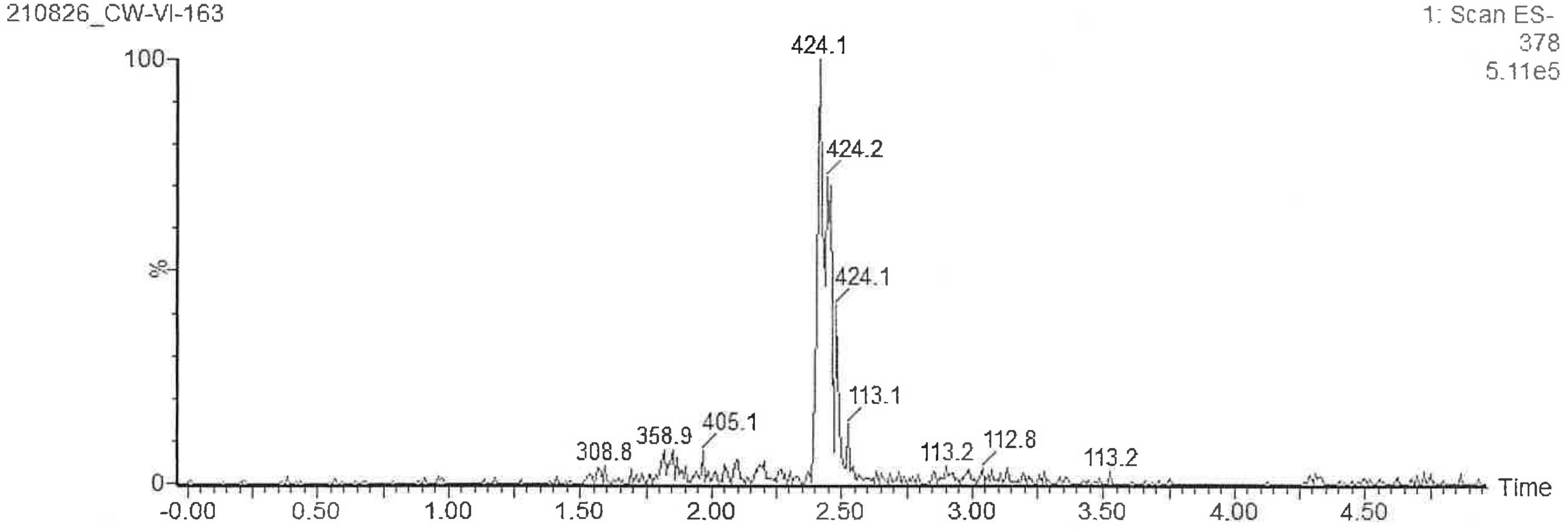
210826_CW-VI-163
1: Scan ES-
378
5.11e5
100
%
0
424.1
424.2
424.1
113.1
308.8
358.9
405.1
113.2
112.8
113.2
-0.00
0.50
1.00
1.50
2.00
2.50
3.00
3.50
4.00
4.50
Time

mz not observed

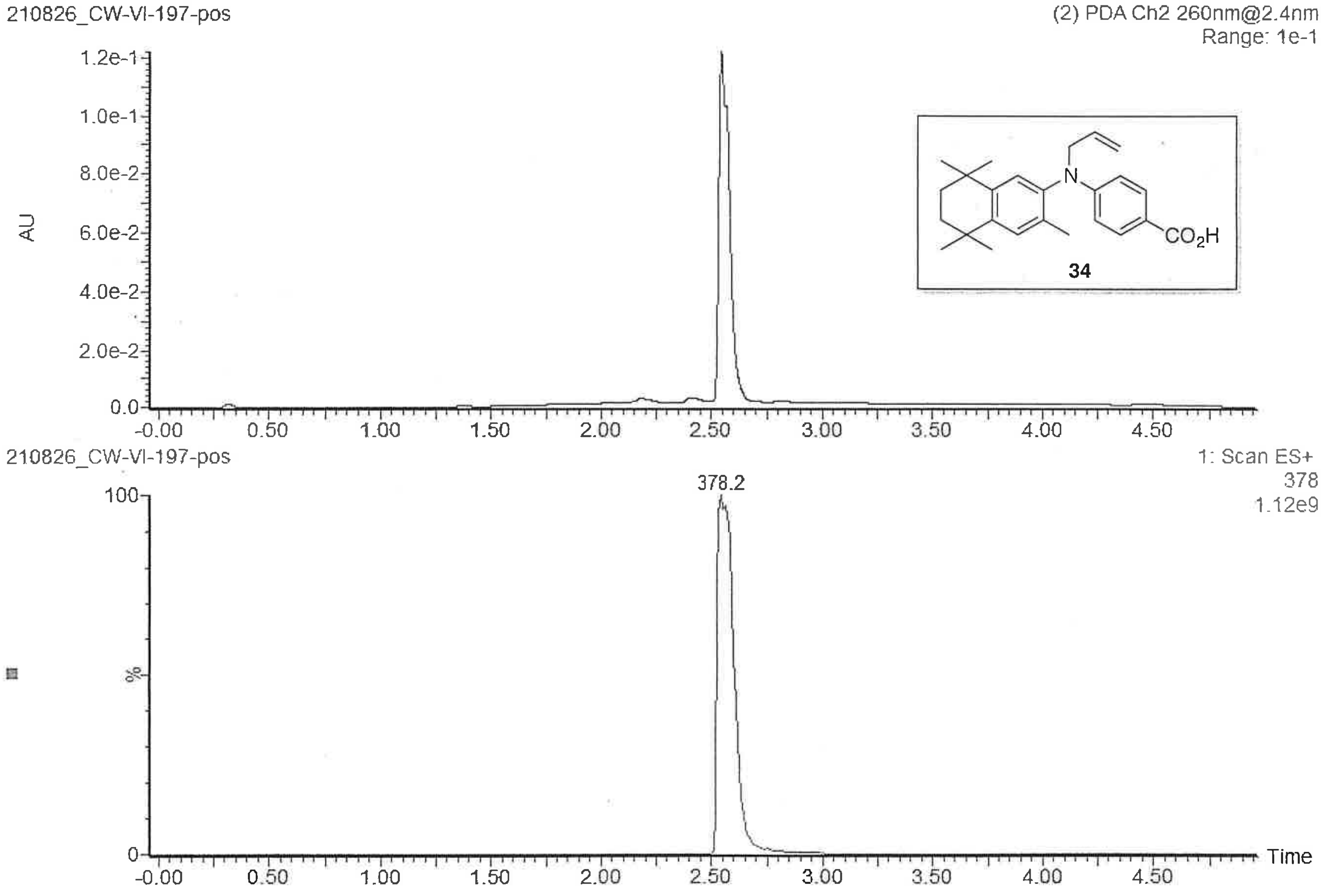

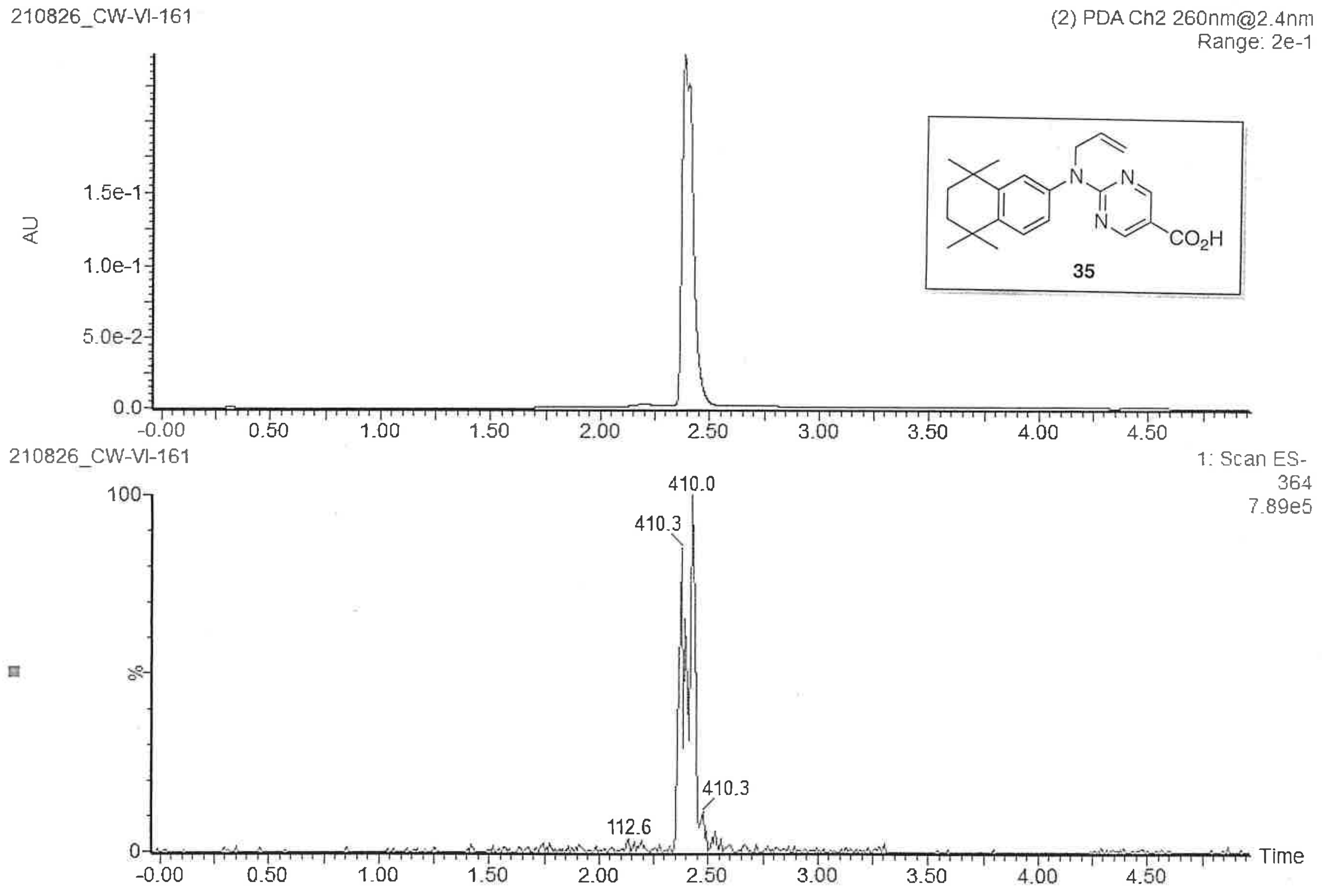
210826_CW-VI-161
(2) PDA Ch2 260nm@2.4nm
Range: 2e-1
AU
1.5e-1
1.0e-1
5.0e-2
0.0
-0.00
0.50
1.00
1.50
2.00
2.50
3.00
3.50
4.00
4.50
N
N
N
CO2H
35
210826_CW-VI-161
1: Scan ES-
364
7.89e5
100
%
0
410.0
410.3
410.3
112.6
Time

mz not observed

210826_CW-VI-171_pos

(2) PDA Ch2 260nm@2.4nm
Range: 6e-1

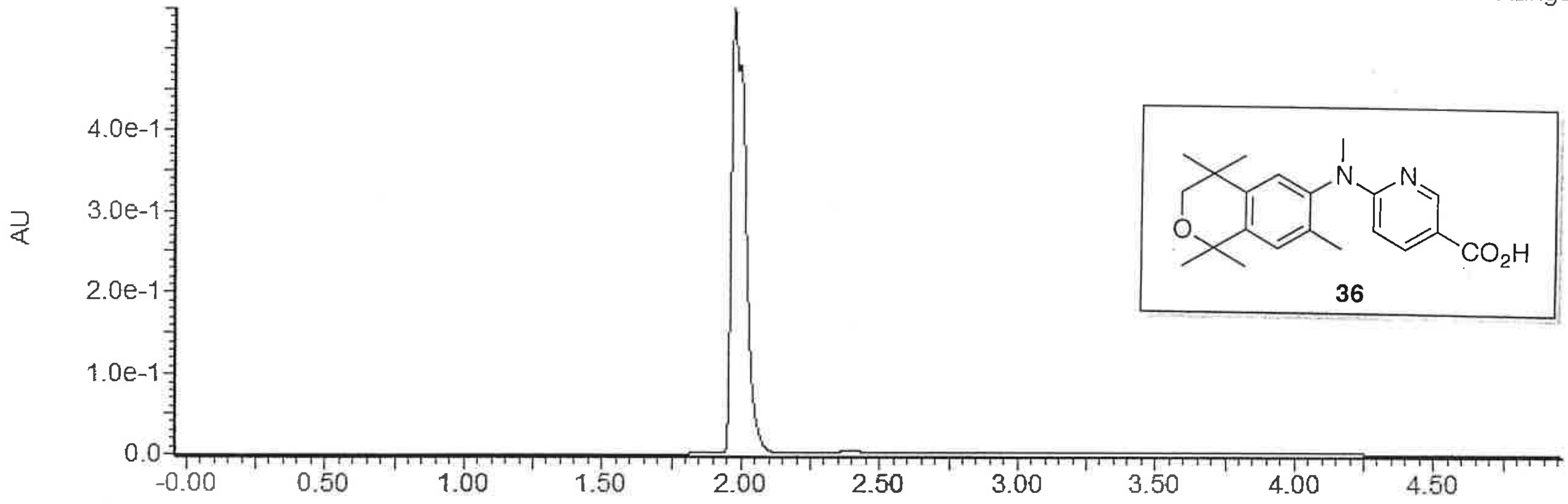

210826_CW-VI-171_pos

1: Scan ES+
355
1.27e9

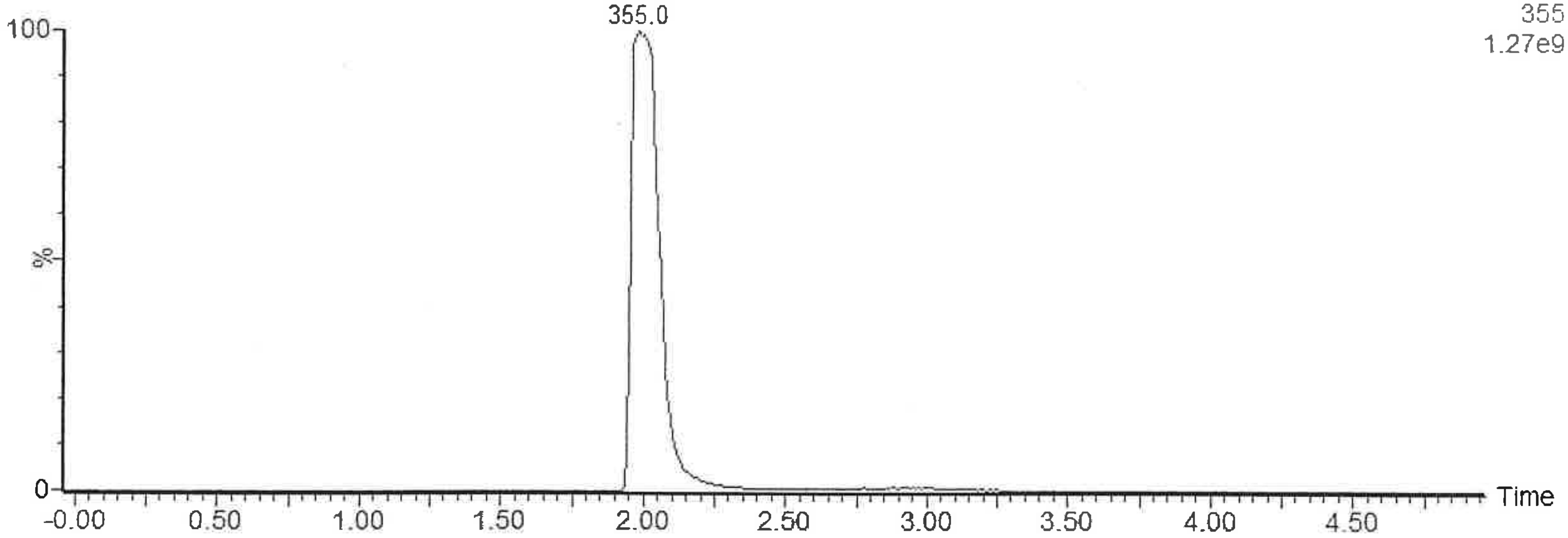

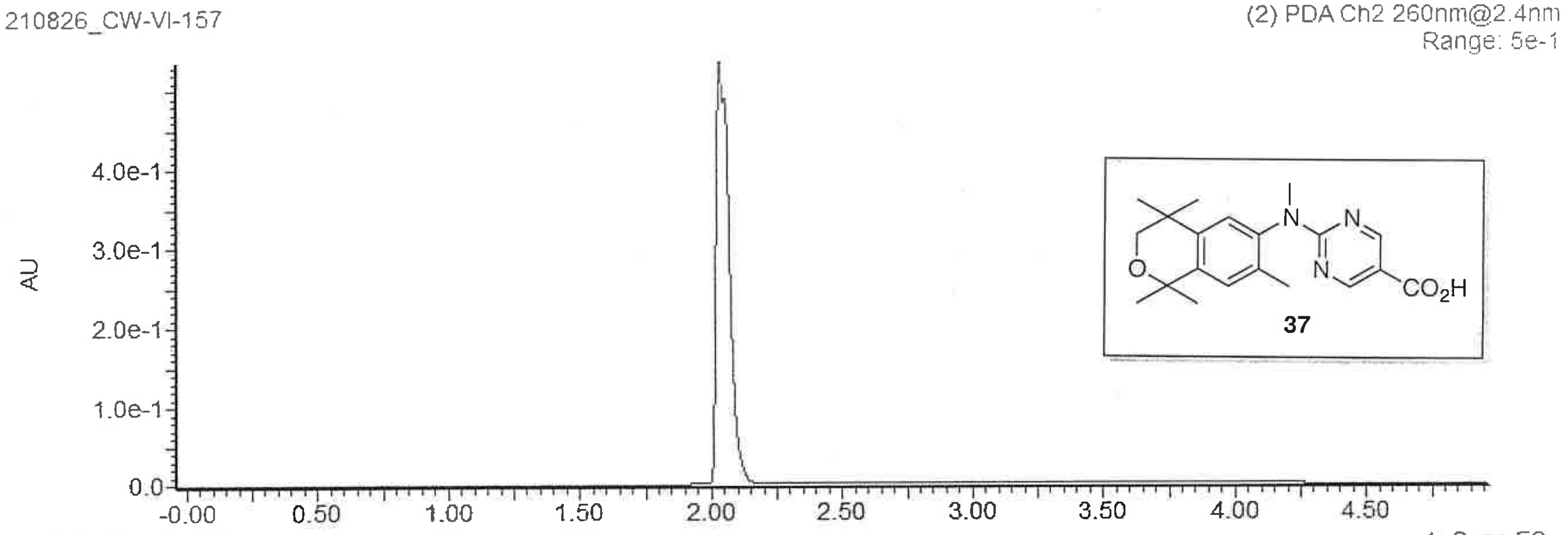
210826_CW-VI-157
(2) PDA Ch2 260nm@2.4nm
Range: 5e-1
AU
4.0e-1
3.0e-1
2.0e-1
1.0e-1
0.0
-0.00
0.50
1.00
1.50
2.00
2.50
3.00
3.50
4.00
4.50
N
N
N
O
CO_2H
37

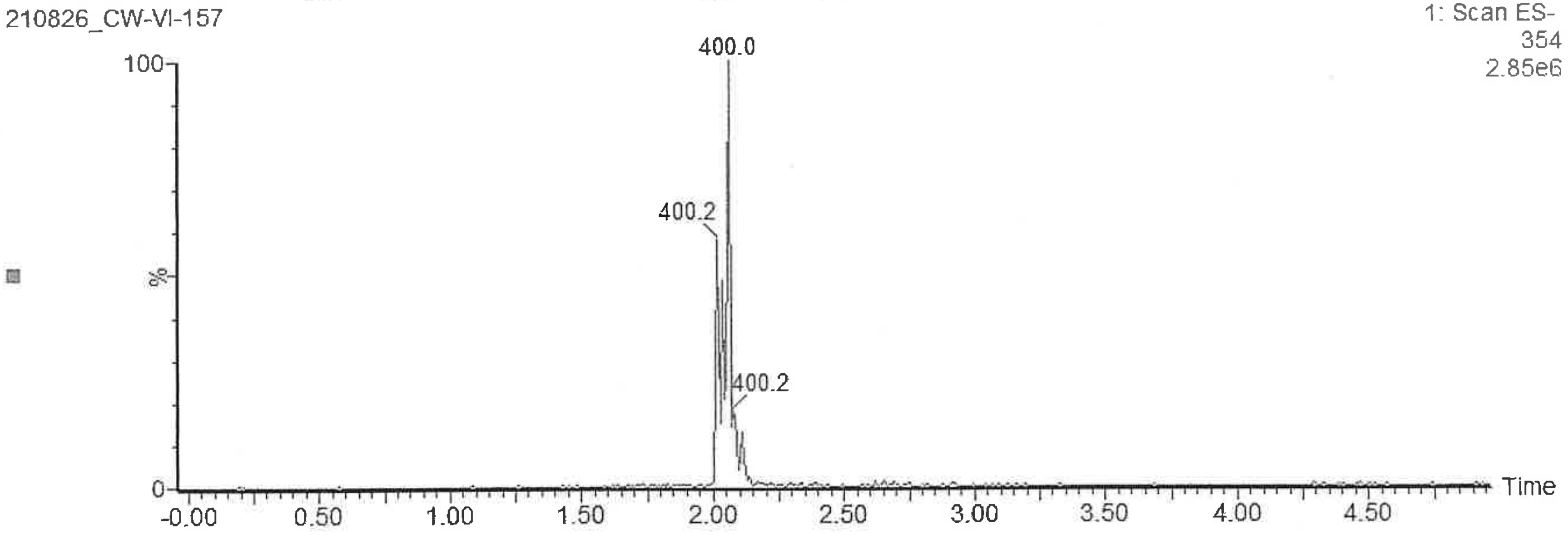
210826_CW-VI-157
1: Scan ES-
354
2.85e6
100
%
0
400.0
400.2
400.2
-0.00
0.50
1.00
1.50
2.00
2.50
3.00
3.50
4.00
4.50
Time

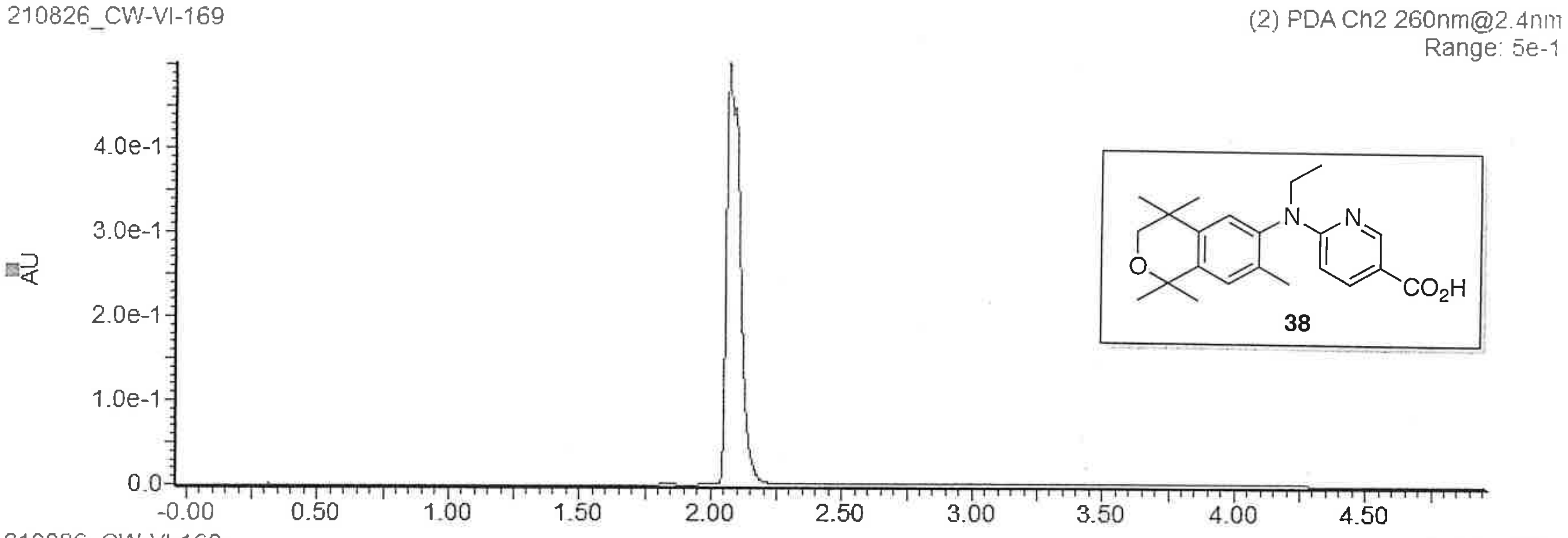

210826_CW-VI-169
(2) PDA Ch2 260nm@2.4nm
Range: 5e-1
AU
4.0e-1
3.0e-1
2.0e-1
1.0e-1
0.0
-0.00
0.50
1.00
1.50
2.00
2.50
3.00
3.50
4.00
4.50
N
O
N
CO_2H

38

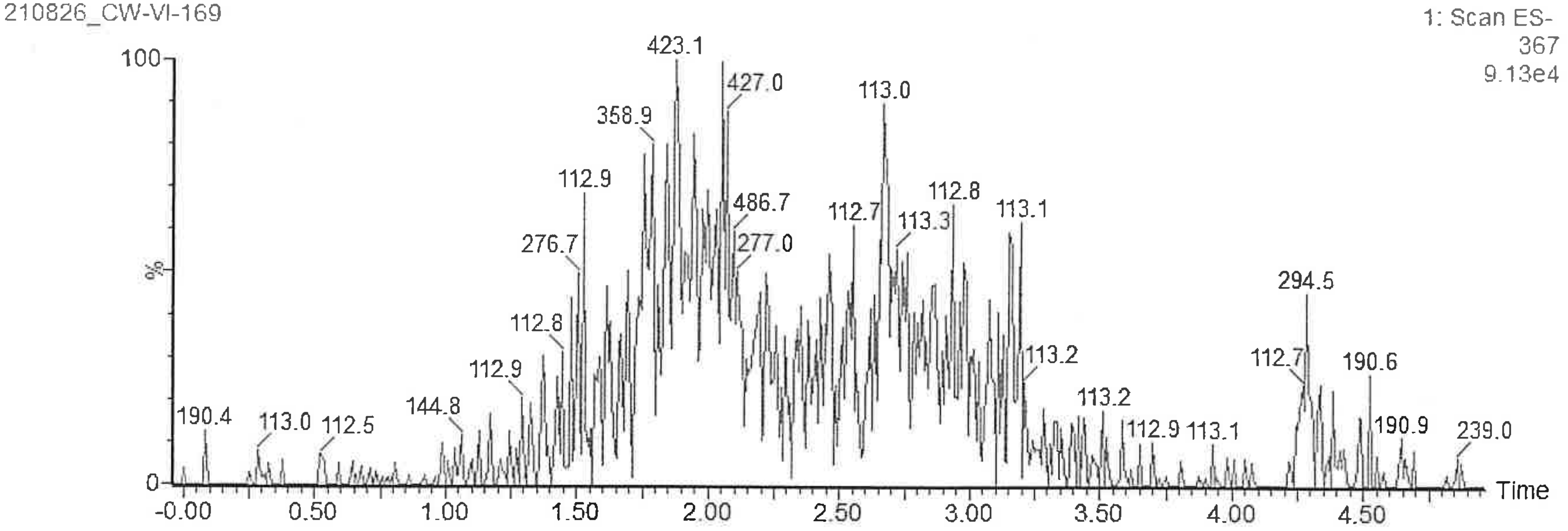

210826_CW-VI-169
1: Scan ES-
367
9.13e4
100
%
0
190.4
113.0
112.5
144.8
112.9
112.8
276.7
112.9
358.9
423.1
427.0
486.7
277.0
112.7
113.0
113.3
112.8
113.1
113.2
113.2
112.9
113.1
112.7
294.5
190.6
190.9
239.0
-0.00
0.50
1.00
1.50
2.00
2.50
3.00
3.50
4.00
4.50
Time

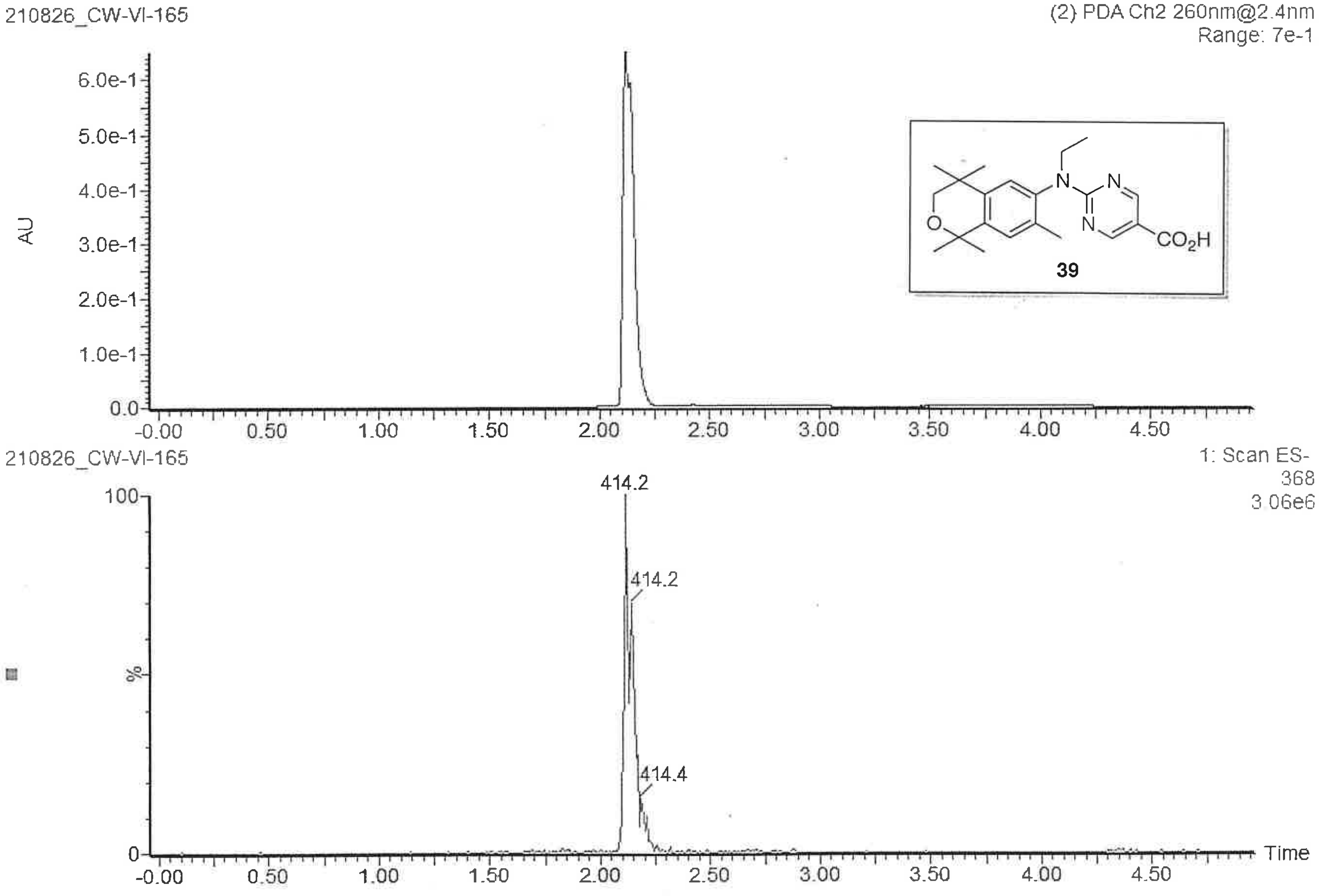
210826_CW-VI-165
(2) PDA Ch2 260nm@2.4nm
Range: 7e-1
AU
6.0e-1
5.0e-1
4.0e-1
3.0e-1
2.0e-1
1.0e-1
0.0
-0.00
0.50
1.00
1.50
2.00
2.50
3.00
3.50
4.00
4.50
N
N
N
O
CO2H
39
210826_CW-VI-165
1: Scan ES-
368
3.06e6
414.2
414.2
414.4
100
%
0
Time

mz not observed

210826_CW-VI-175-pos

(2) PDA Ch2 260nm@2.4nm
Range: 6e-1

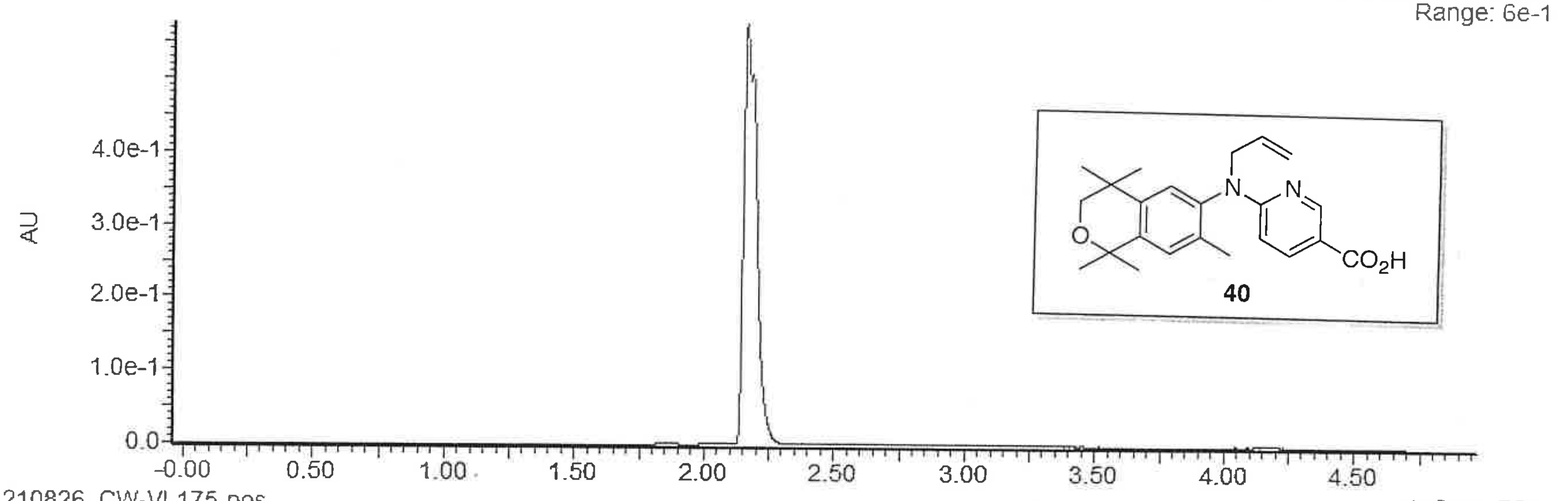

210826_CW-VI-175-pos

1: Scan ES+
381
1.30e9

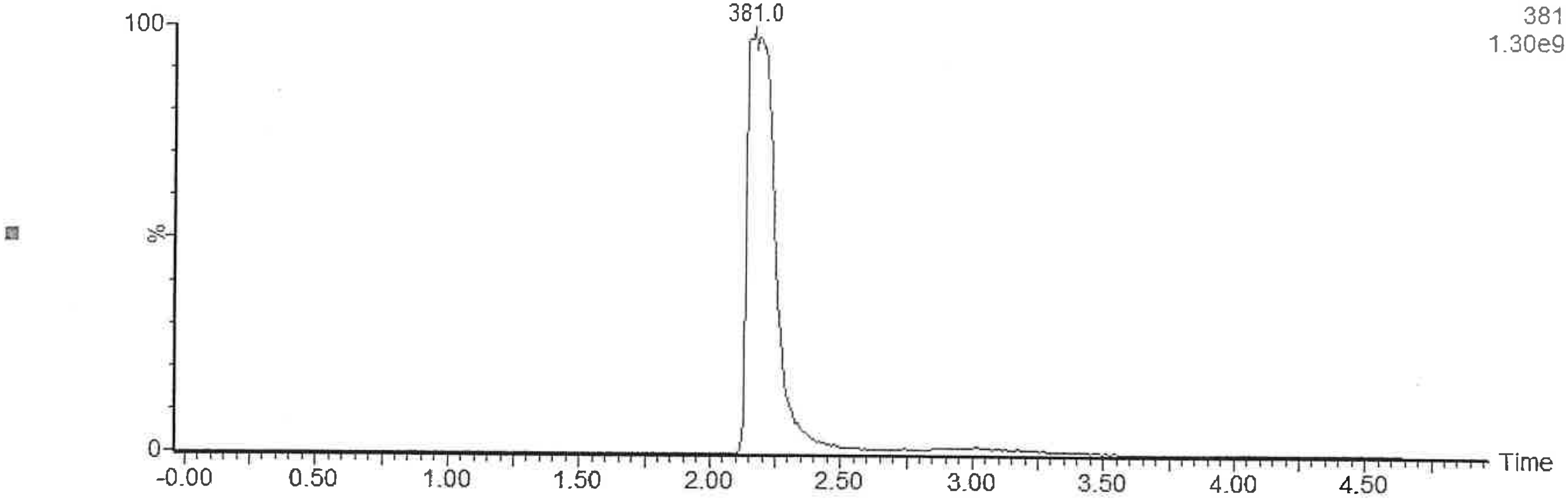

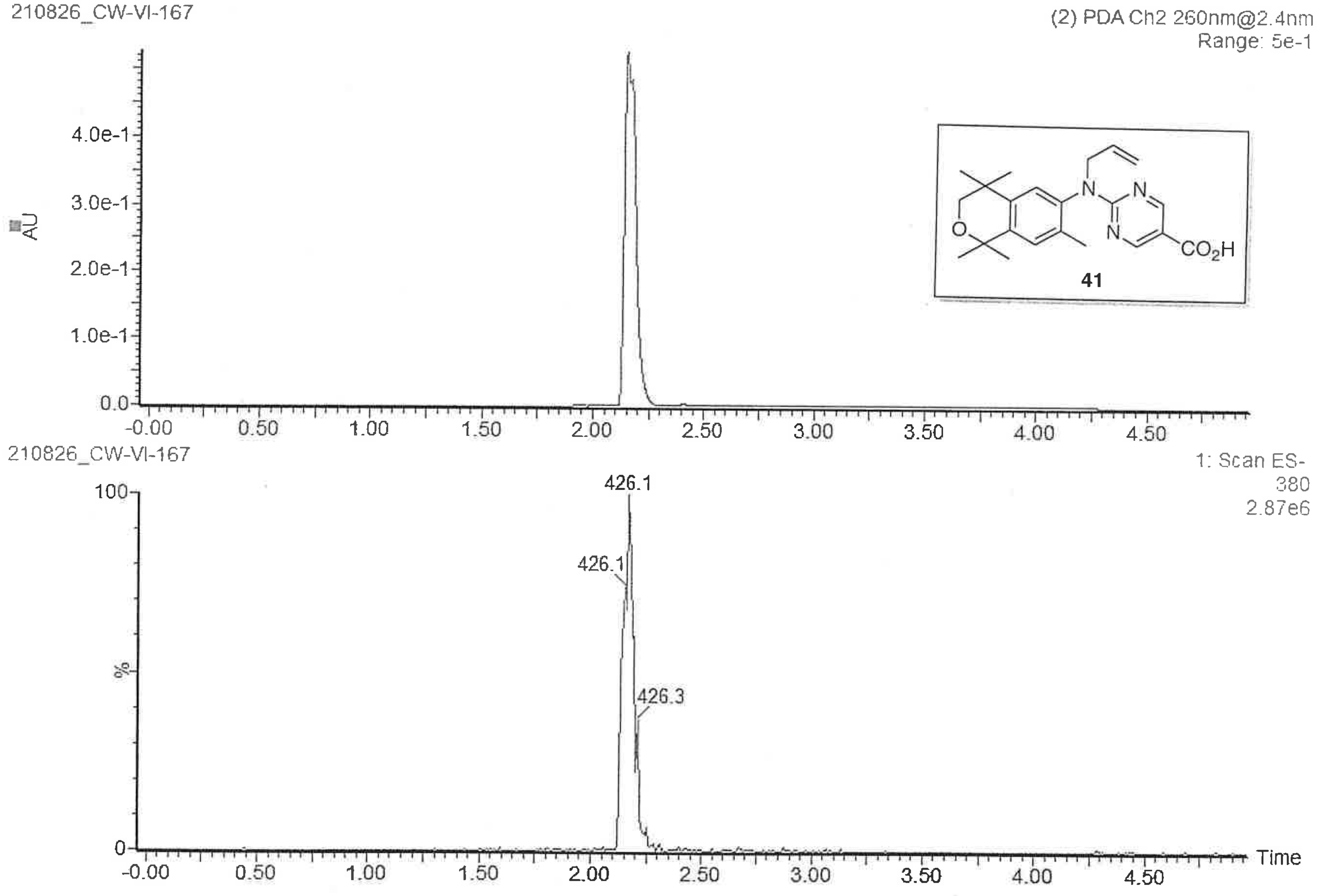
210826_CW-VI-167
(2) PDA Ch2 260nm@2.4nm
Range: 5e-1
AU
4.0e-1
3.0e-1
2.0e-1
1.0e-1
0.0
-0.00
0.50
1.00
1.50
2.00
2.50
3.00
3.50
4.00
4.50
O
N
N
N
CO_2H
41
210826_CW-VI-167
1: Scan ES-
380
2.87e6
100
%
0
426.1
426.1
426.3
-0.00
0.50
1.00
1.50
2.00
2.50
3.00
3.50
4.00
4.50
Time

mz not observed

210826_CW-VI-201-pos

(2) PDA Ch2 260nm@2.4nm
Range: 1e-1

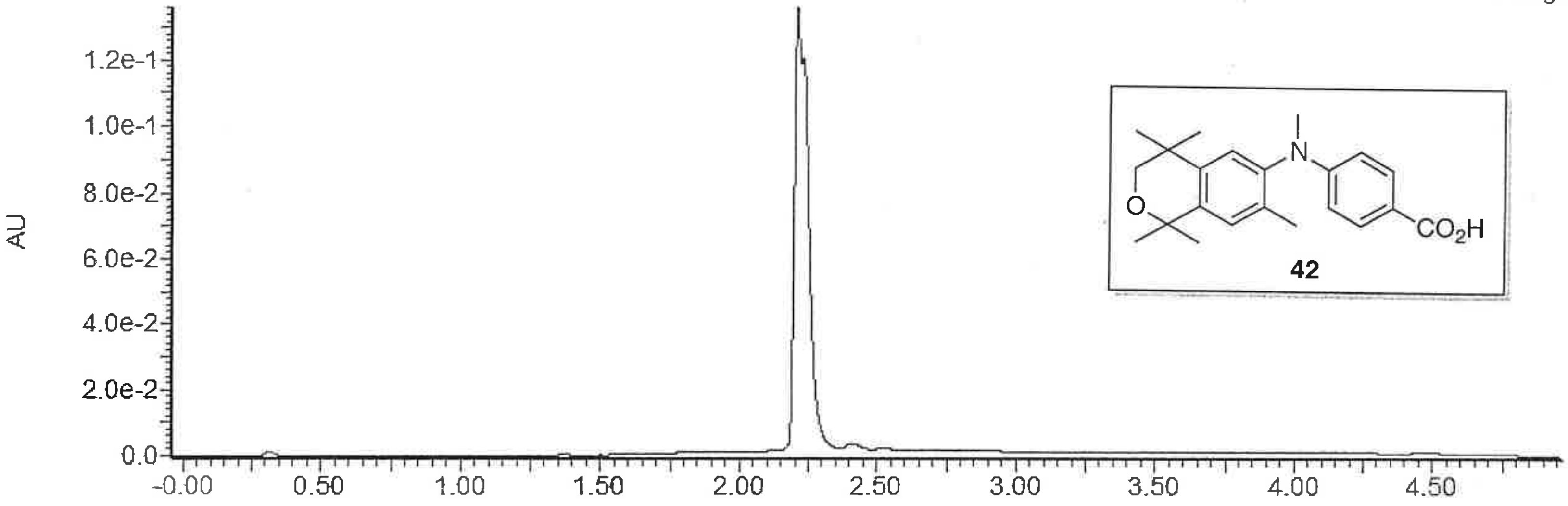

210826_CW-VI-201-pos

1: Scan ES+
354
8.78e8

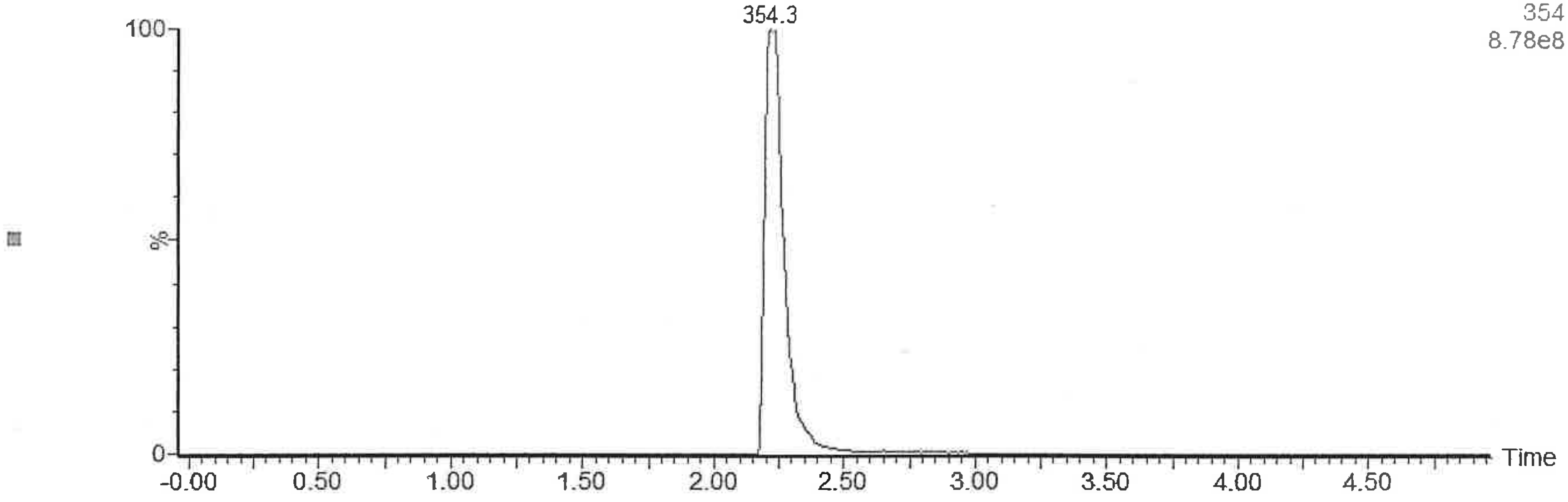

mz not observed

210826_CW-VI-199-pos

(2) PDA Ch2 260nm@2.4nm
Range: 1e-1

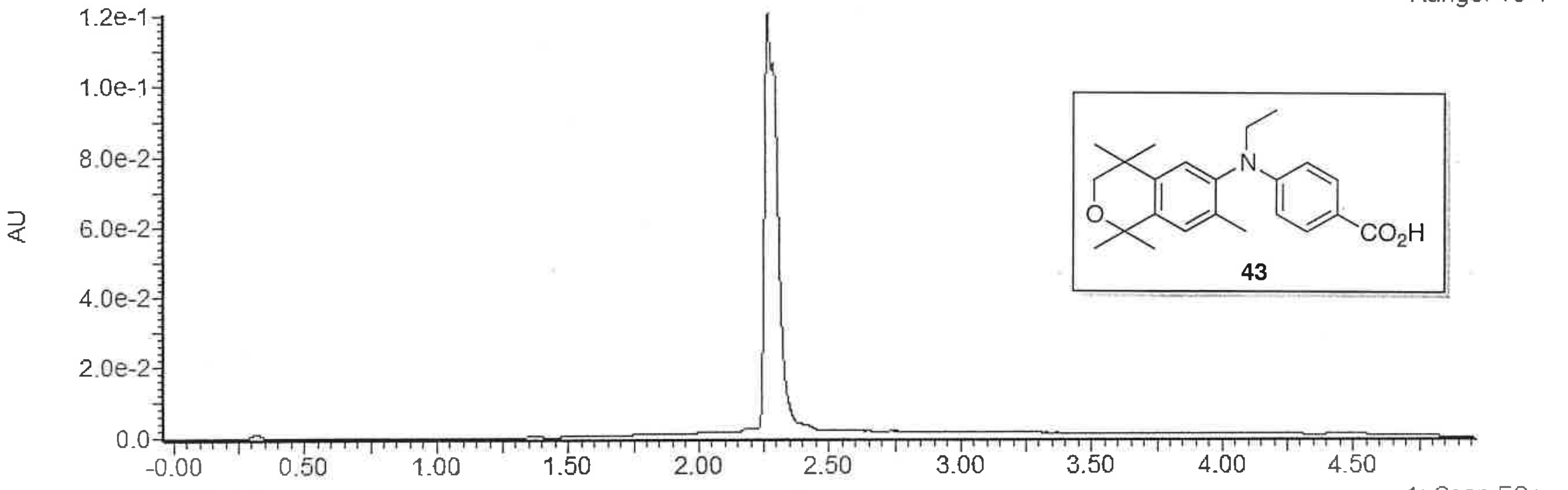

210826_CW-VI-199-pos

1: Scan ES+
368
8.65e8

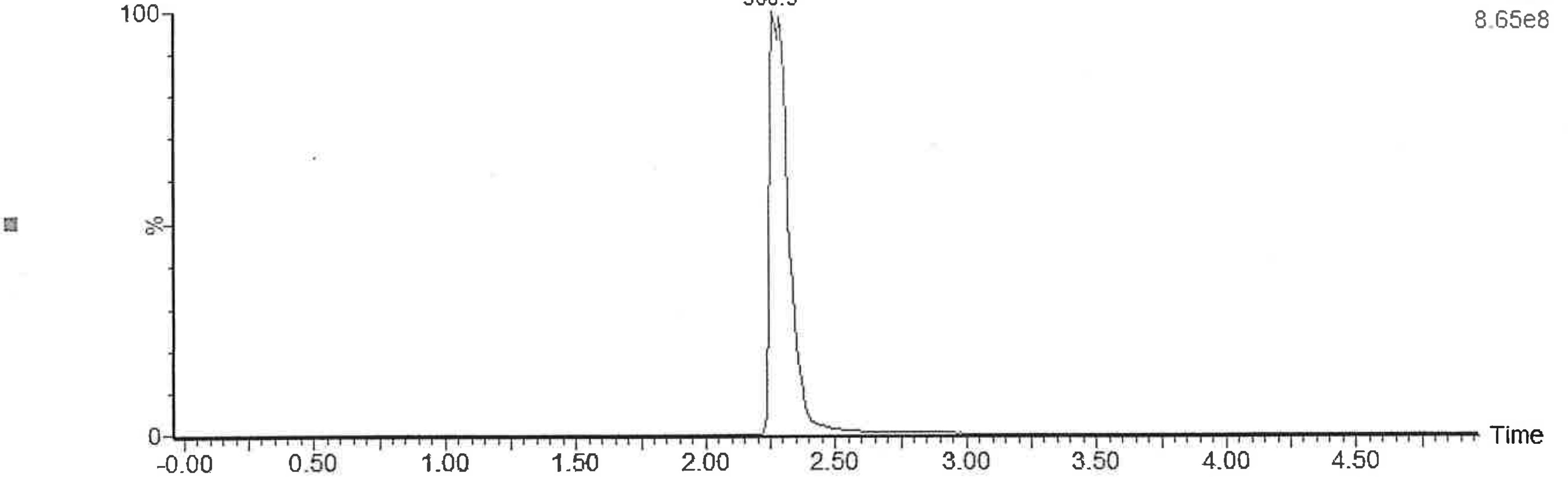

mz not observed

210826_CW-VI-203-pos

(2) PDA Ch2 260nm@2.4nm
Range: 1e-1

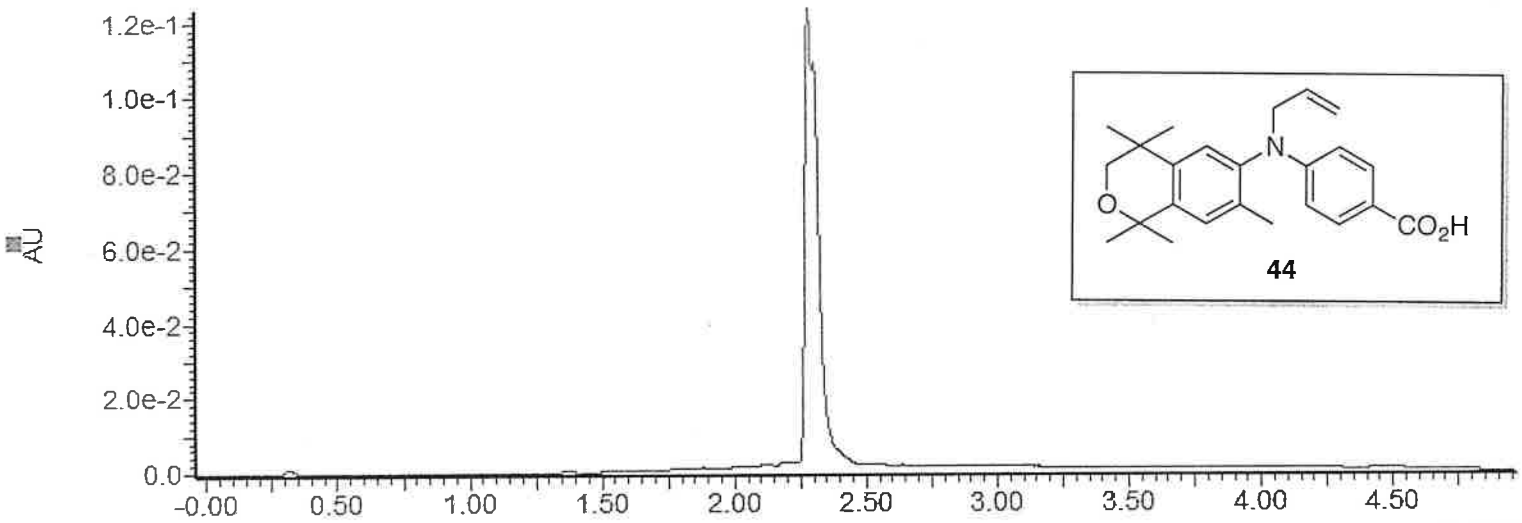

210826_CW-VI-203-pos

1: Scan ES+
380
8.24e8

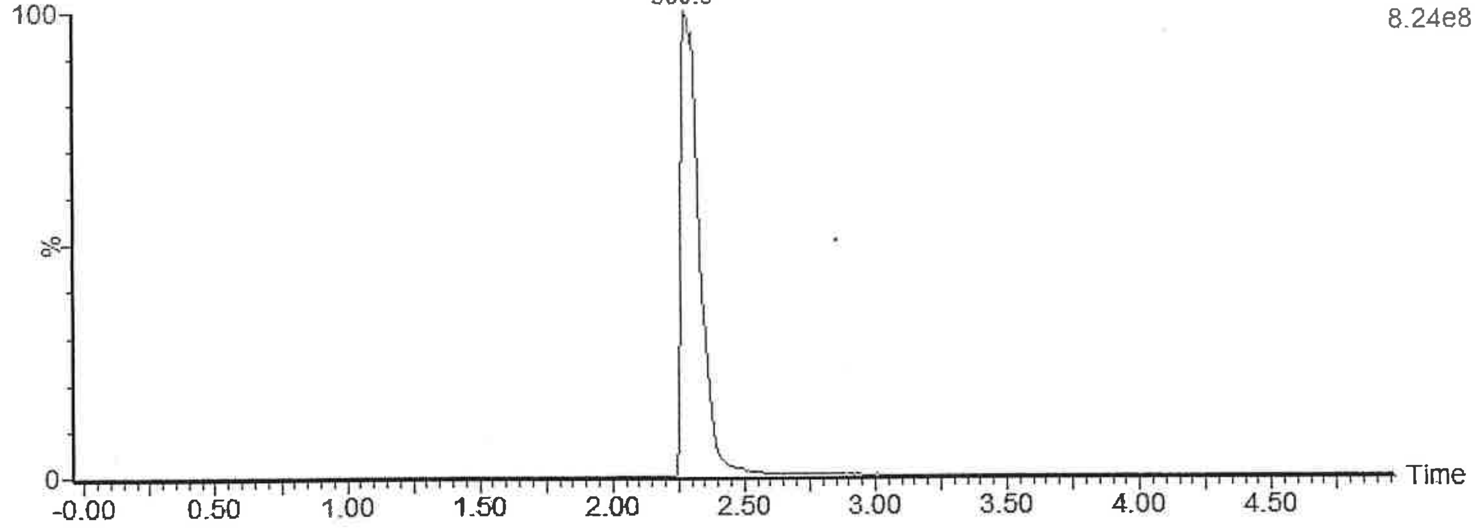

CWVI-195

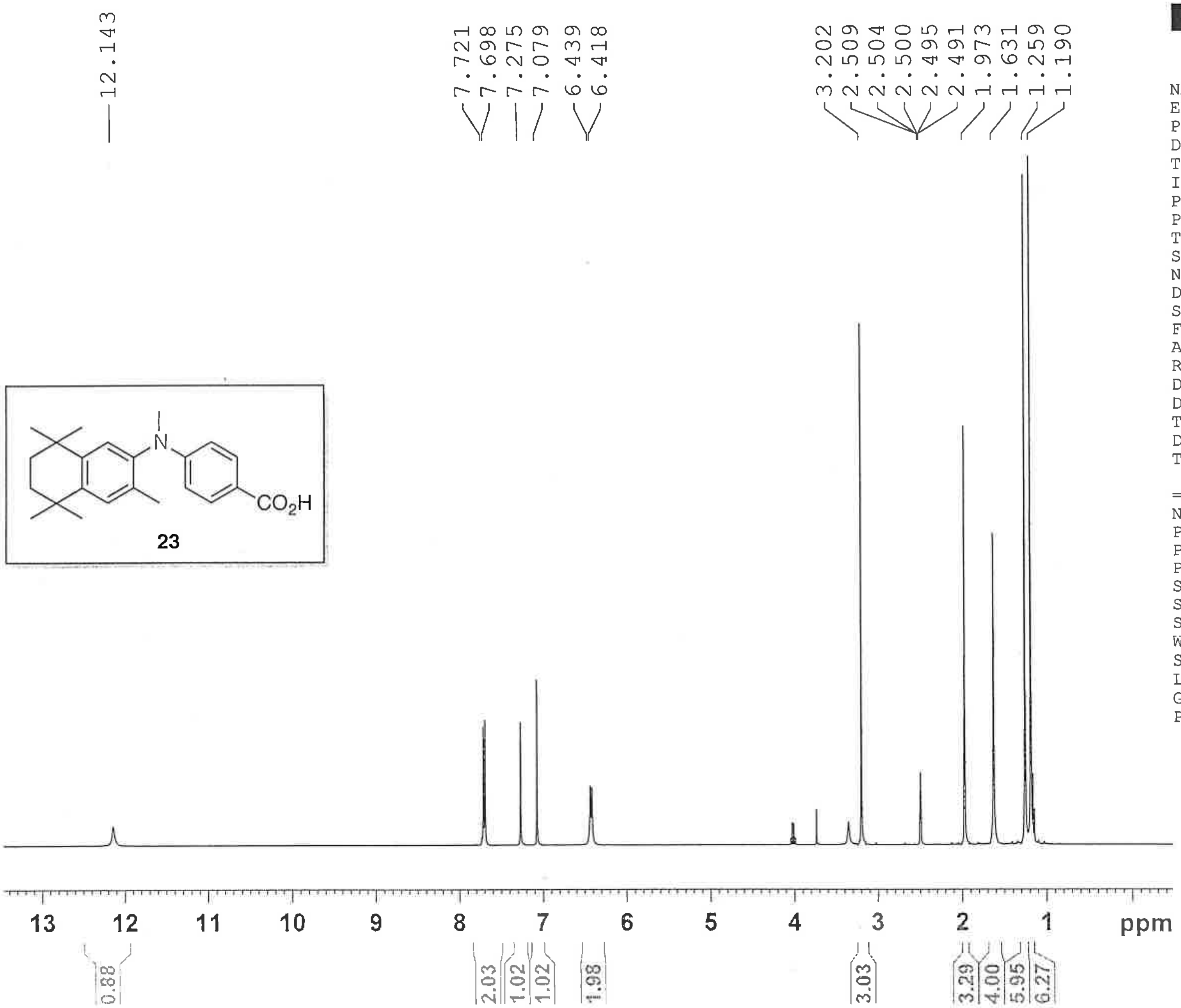

23

NAME	CWVI-195
EXPNO	1
PROCNO	1
Date_	20181010
Time	14.42
INSTRUM	spect
PROBHD	5 mm PABBO BB-
PULPROG	zg30
TD	65536
SOLVENT	DMSO
NS	16
DS	2
SWH	8223.685 Hz
FIDRES	0.125483 Hz
AQ	3.9846387 sec
RG	4
DW	60.800 usec
DE	6.50 usec
TE	292.2 K
D1	1.00000000 sec
TD0	1

======== CHANNEL f1 ========

NUC1	1H
P1	14.75 usec
PL1	0.50 dB
PL1W	12.76071072 W
SFO1	400.1324710 MHz
SI	32768
SF	400.1300034 MHz
WDW	EM
SSB	0
LB	0.30 Hz
GB	0
PC	1.00

CWVI-195

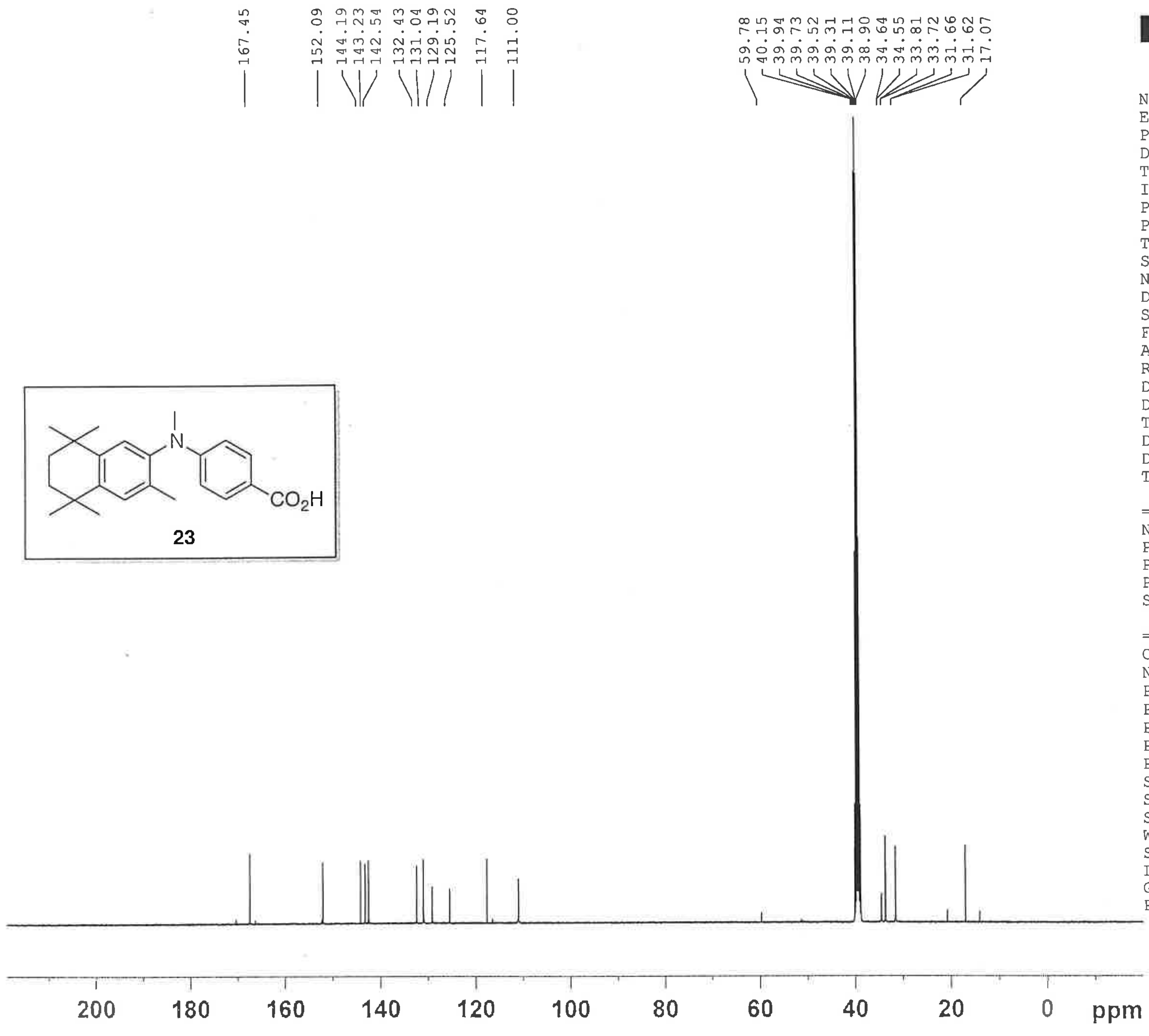

BRUKER

NAME	CWVI-195
EXPNO	2
PROCNO	1
Date_	20181010
Time_	14.47
INSTRUM	spect
PROBHD	5 mm PABBO BB-
PULPROG	zgig30
TD	65536
SOLVENT	DMSO
NS	6036
DS	4
SWH	24038.461 Hz
FIDRES	0.366798 Hz
AQ	1.3631988 sec
RG	2050
DW	20.800 usec
DE	6.50 usec
TE	292.4 K
D1	2.00000000 sec
D11	0.03000000 sec
TD0	1

======== CHANNEL f1 ========

NUC1	13C
P1	8.25 usec
PL1	-2.10 dB
PL1W	60.29227829 W
SFO1	100.6228298 MHz

======== CHANNEL f2 ========

CPDPRG2	waltz16
NUC2	1H
PCPD2	90.00 usec
PL2	0.50 dB
PL12	16.21 dB
PL2W	12.76071072 W
PL12W	0.34266910 W
SFO2	400.1316005 MHz
SI	32768
SF	100.6128128 MHz
WDW	EM
SSB	0
LB	1.00 Hz
GB	0
PC	1.40

CWVI-159

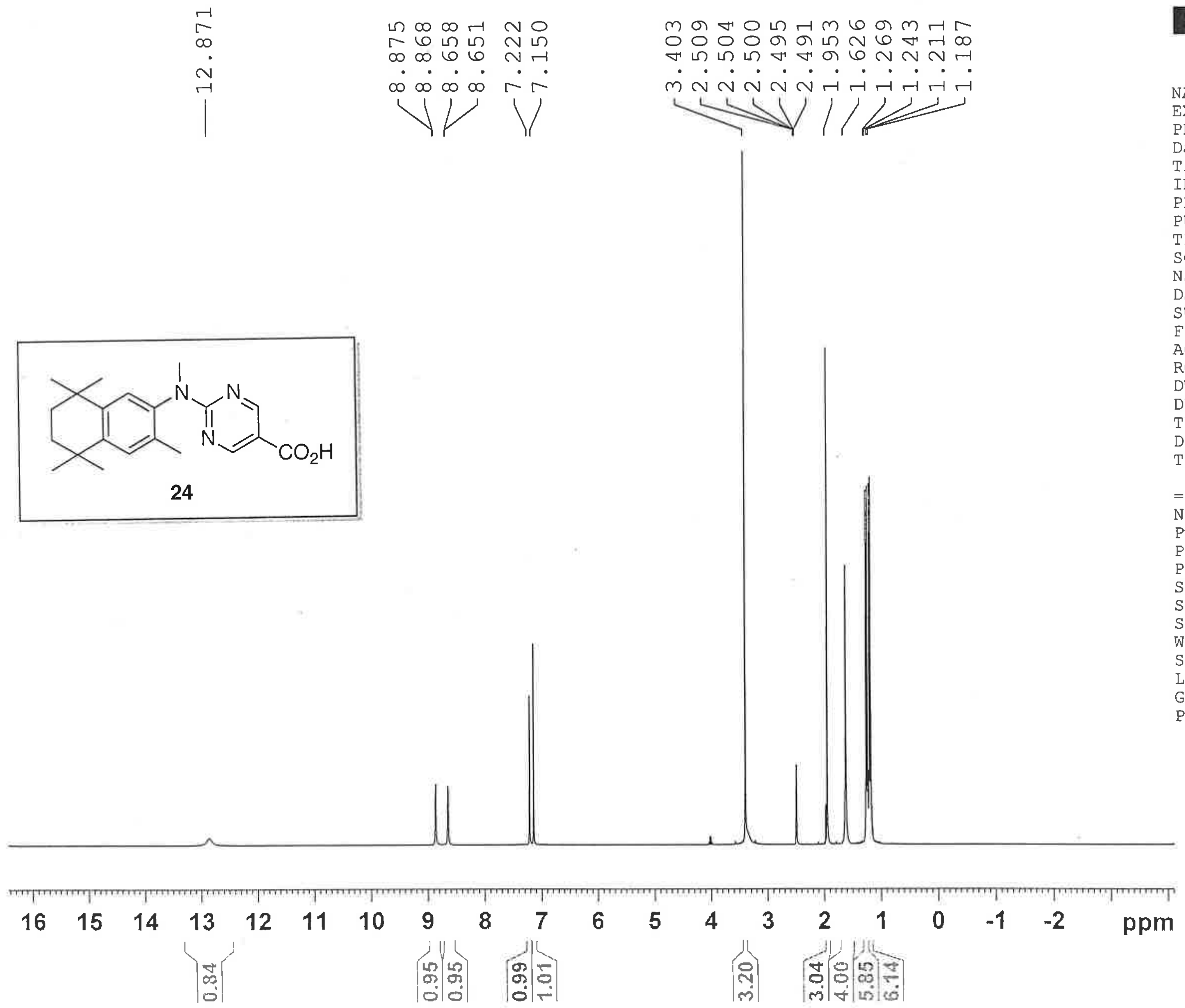

24

NAME	CWVI-159
EXPNO	1
PROCNO	1
Date_	20181010
Time	10.04
INSTRUM	spect
PROBHD	5 mm PABBO BB-
PULPROG	zg30
TD	65536
SOLVENT	DMSO
NS	16
DS	2
SWH	8223.685 Hz
FIDRES	0.125483 Hz
AQ	3.9846387 sec
RG	4
DW	60.800 usec
DE	6.50 usec
TE	291.9 K
D1	1.00000000 sec
TD0	1

======== CHANNEL f1 ========

NUC1	1H
P1	14.75 usec
PL1	0.50 dB
PL1W	12.76071072 W
SFO1	400.1324710 MHz
SI	32768
SF	400.1300034 MHz
WDW	EM
SSB	0
LB	0.30 Hz
GB	0
PC	1.00

CWVI-159

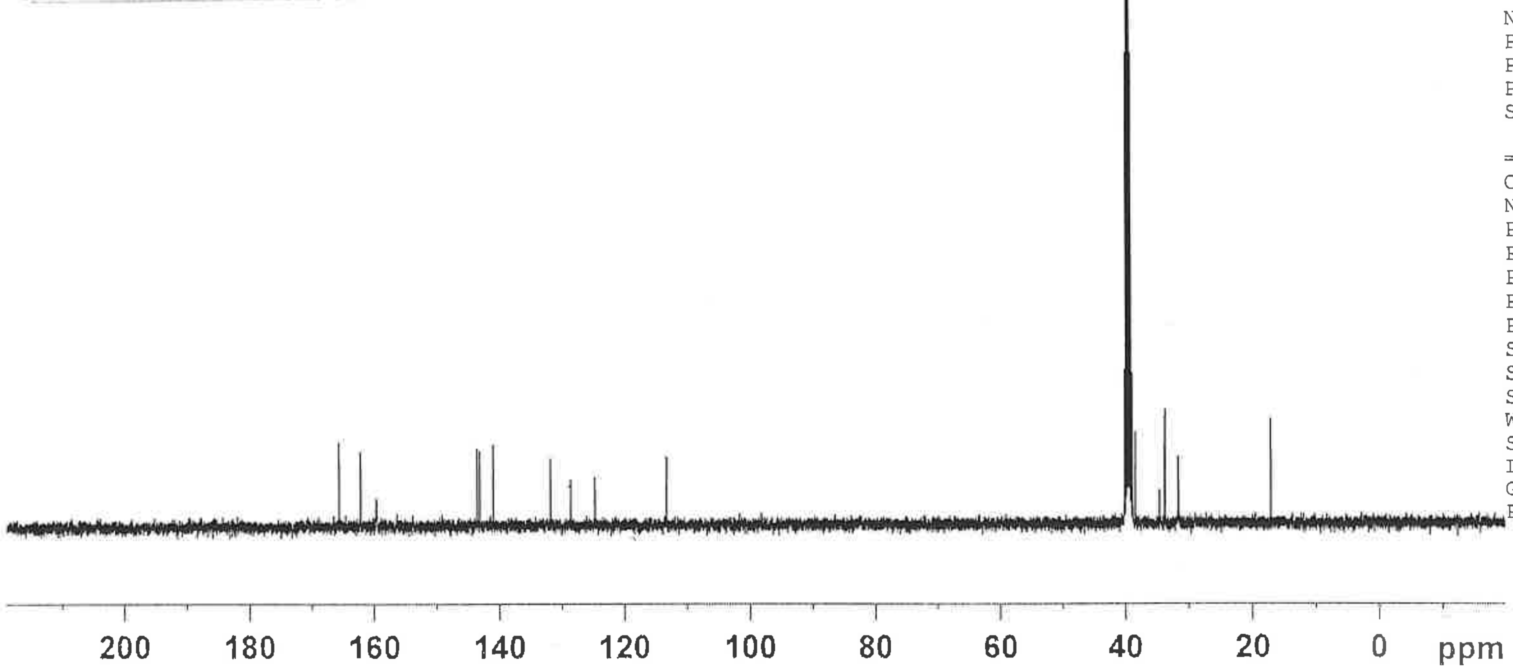

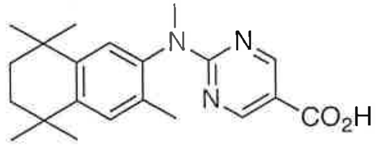

24

NAME	CWVI-159
EXPNO	2
PROCNO	1
Date_	20181010
Time	10.10
INSTRUM	spect
PROBHD	5 mm PABBO BB-
PULPROG	zgig30
TD	65536
SOLVENT	DMSO
NS	118
DS	4
SWH	24038.461 Hz
FIDRES	0.366798 Hz
AQ	1.3631988 sec
RG	2050
DW	20.800 usec
DE	6.50 usec
TE	292.1 K
D1	2.00000000 sec
D11	0.03000000 sec
TD0	1

======== CHANNEL f1 ========

NUC1	13C
P1	8.25 usec
PL1	-2.10 dB
PL1W	60.29227829 W
SFO1	100.6228298 MHz

======== CHANNEL f2 ========

CPDPRG2	waltz16
NUC2	1H
PCPD2	90.00 usec
PL2	0.50 dB
PL12	16.21 dB
PL2W	12.76071072 W
PL12W	0.34266910 W
SFO2	400.1316005 MHz
SI	32768
SF	100.6128113 MHz
WDW	EM
SSB	0
LB	1.00 Hz
GB	0
PC	1.40

CWVI-155

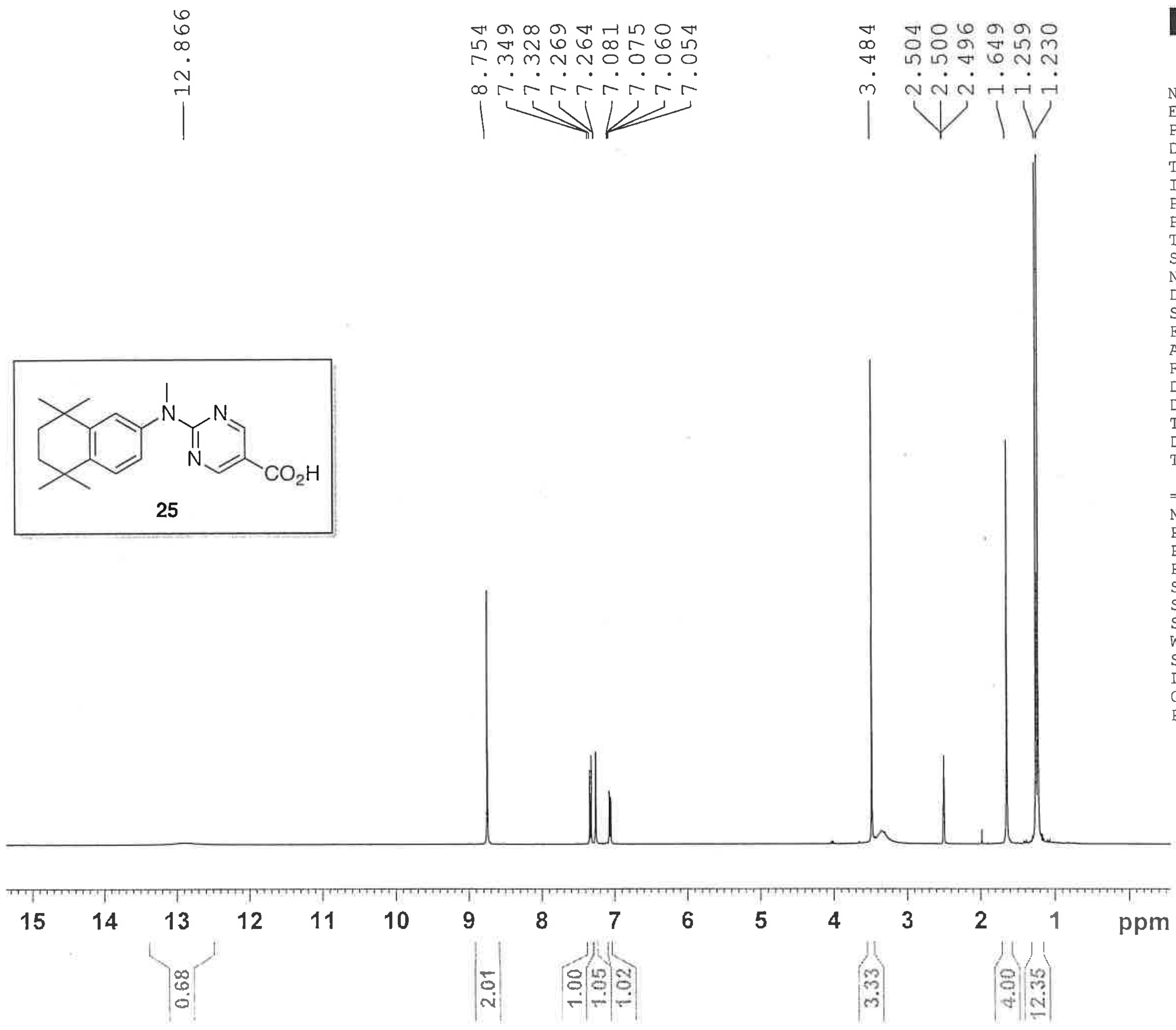

NAME	CWVI-155
EXPNO	1
PROCNO	1
Date_	20181010
Time_	20.50
INSTRUM	spect
PROBHD	5 mm PABBO BB-
PULPROG	zg30
TD	65536
SOLVENT	DMSO
NS	16
DS	2
SWH	8223.685 Hz
FIDRES	0.125483 Hz
AQ	3.9846387 sec
RG	4
DW	60.800 usec
DE	6.50 usec
TE	292.6 K
D1	1.00000000 sec
TD0	1

======== CHANNEL f1 ========

NUC1	1H
P1	14.75 usec
PL1	0.50 dB
PL1W	12.76071072 W
SFO1	400.1324710 MHz
SI	32768
SF	400.1300031 MHz
WDW	EM
SSB	0
LB	0.30 Hz
GB	.0
PC	1.00

CWVI-155

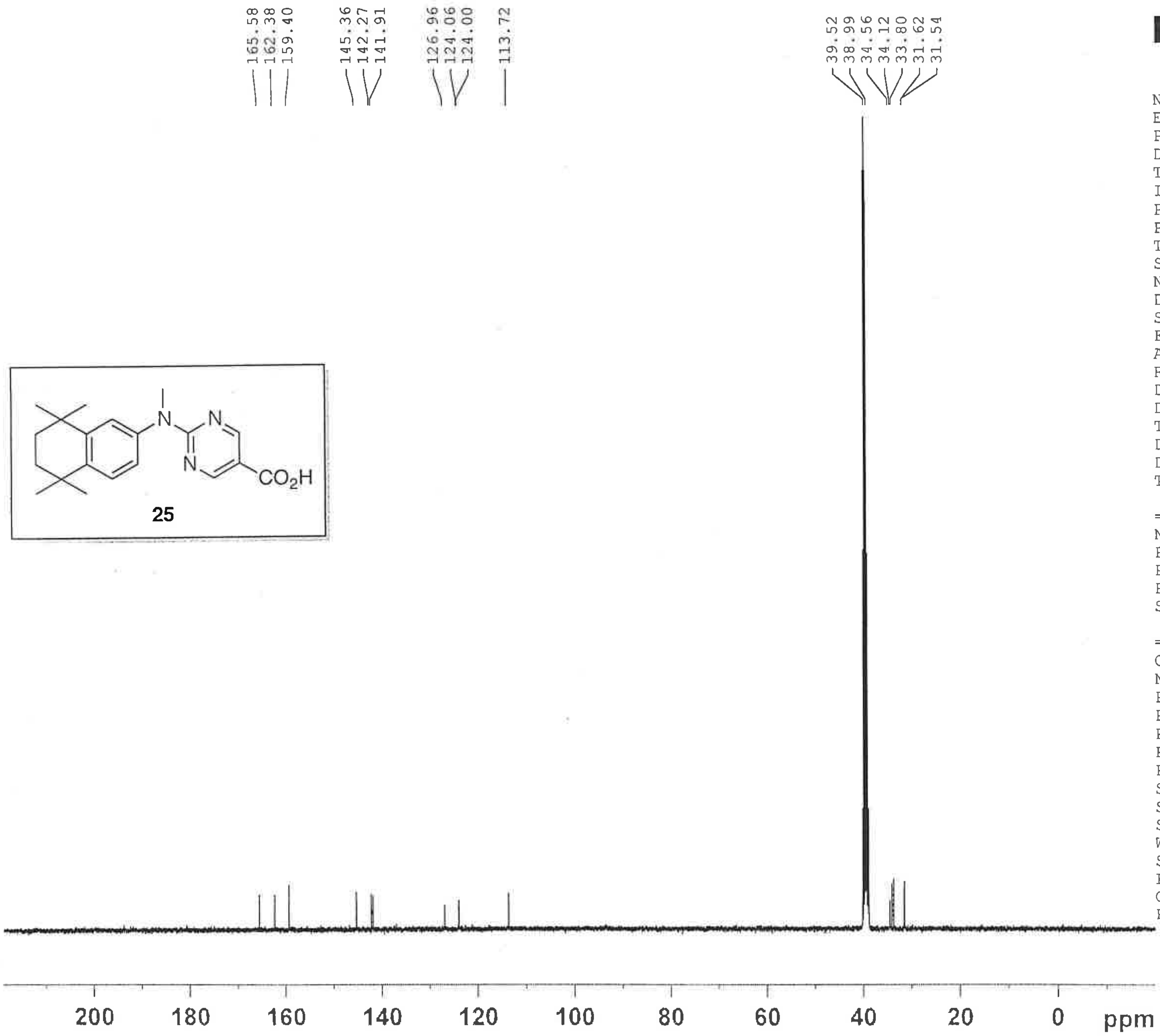

25

NAME	CWVI-155
EXPNO	2
PROCNO	1
Date_	20181010
Time	20.54
INSTRUM	spect
PROBHD	5 mm PABBO BB-
PULPROG	zgig30
TD	65536
SOLVENT	DMSO
NS	658
DS	4
SWH	24038.461 Hz
FIDRES	0.366798 Hz
AQ	1.3631988 sec
RG	2050
DW	20.800 usec
DE	6.50 usec
TE	292.4 K
D1	2.00000000 sec
D11	0.03000000 sec
TD0	1

======== CHANNEL f1 ========

NUC1	13C
P1	8.25 usec
PL1	-2.10 dB
PL1W	60.29227829 W
SFO1	100.6228298 MHz

======== CHANNEL f2 ========

CPDPRG2	waltz16
NUC2	1H
PCPD2	90.00 usec
PL2	0.50 dB
PL12	16.21 dB
PL2W	12.76071072 W
PL12W	0.34266910 W
SFO2	400.1316005 MHz
SI	32768
SF	100.6128120 MHz
WDW	EM
SSB	0
LB	1.00 Hz
GB	0
PC	1.40

CWVI-141 f6-f13 (pure)

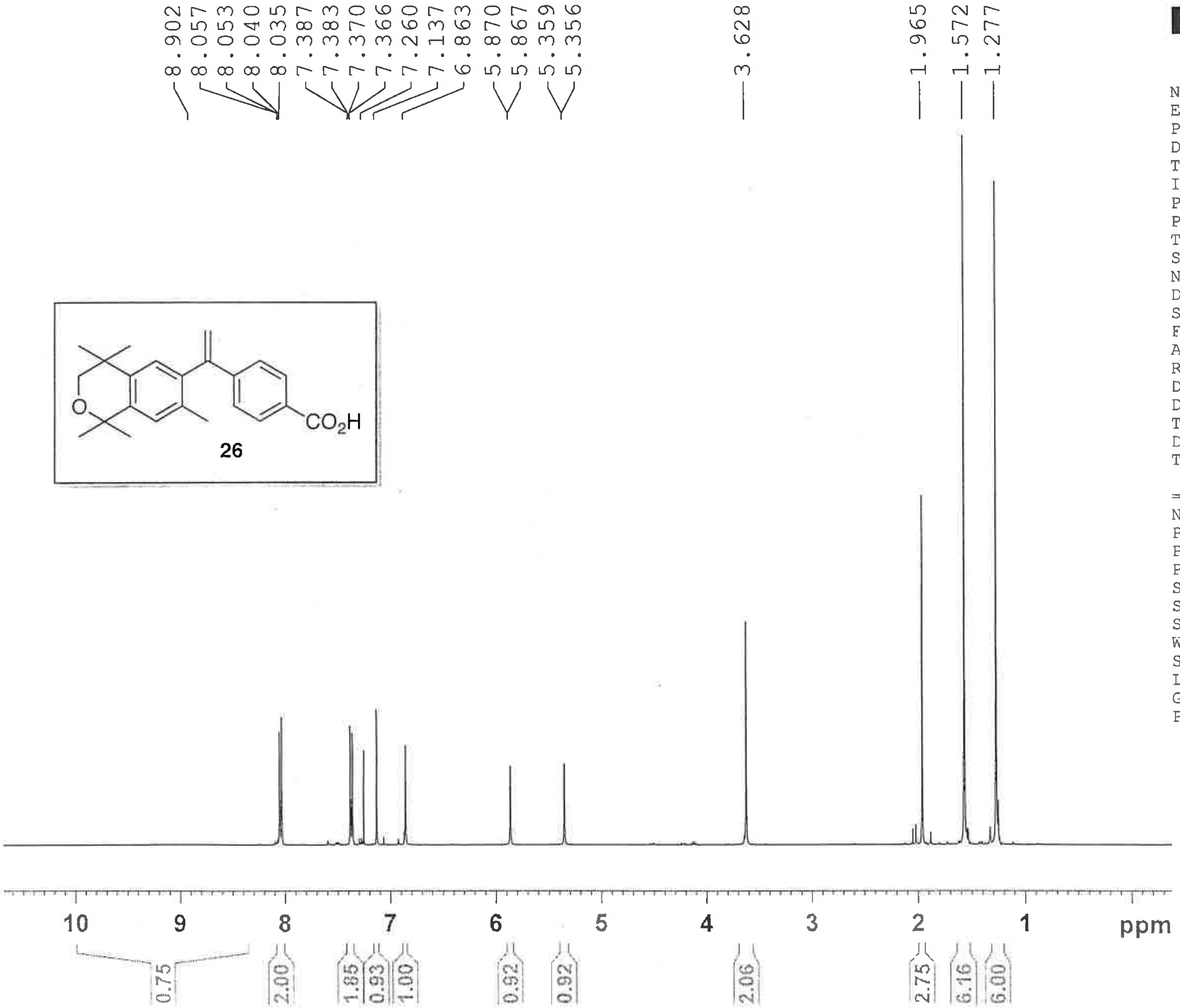

26

BRUKER

NAME	CWVI-141
EXPNO	1
PROCNO	1
Date_	20180711
Time	14.27
INSTRUM	spect
PROBHD	5 mm PABBO BB-
PULPROG	zg30
TD	65536
SOLVENT	CDCl3
NS	16
DS	2
SWH	8223.685 Hz
FIDRES	0.125483 Hz
AQ	3.9846387 sec
RG	4
DW	60.800 usec
DE	6.50 usec
TE	294.5 K
D1	1.00000000 sec
TD0	1

======== CHANNEL f1 ========

NUC1	1H
P1	14.75 usec
PL1	0.50 dB
PL1W	12.76071072 W
SFO1	400.1324710 MHz
SI	32768
SF	400.1300099 MHz
WDW	EM
SSB	0
LB	0.30 Hz
GB	0
PC	1.00

CWVI-141 f6-f13 (pure)

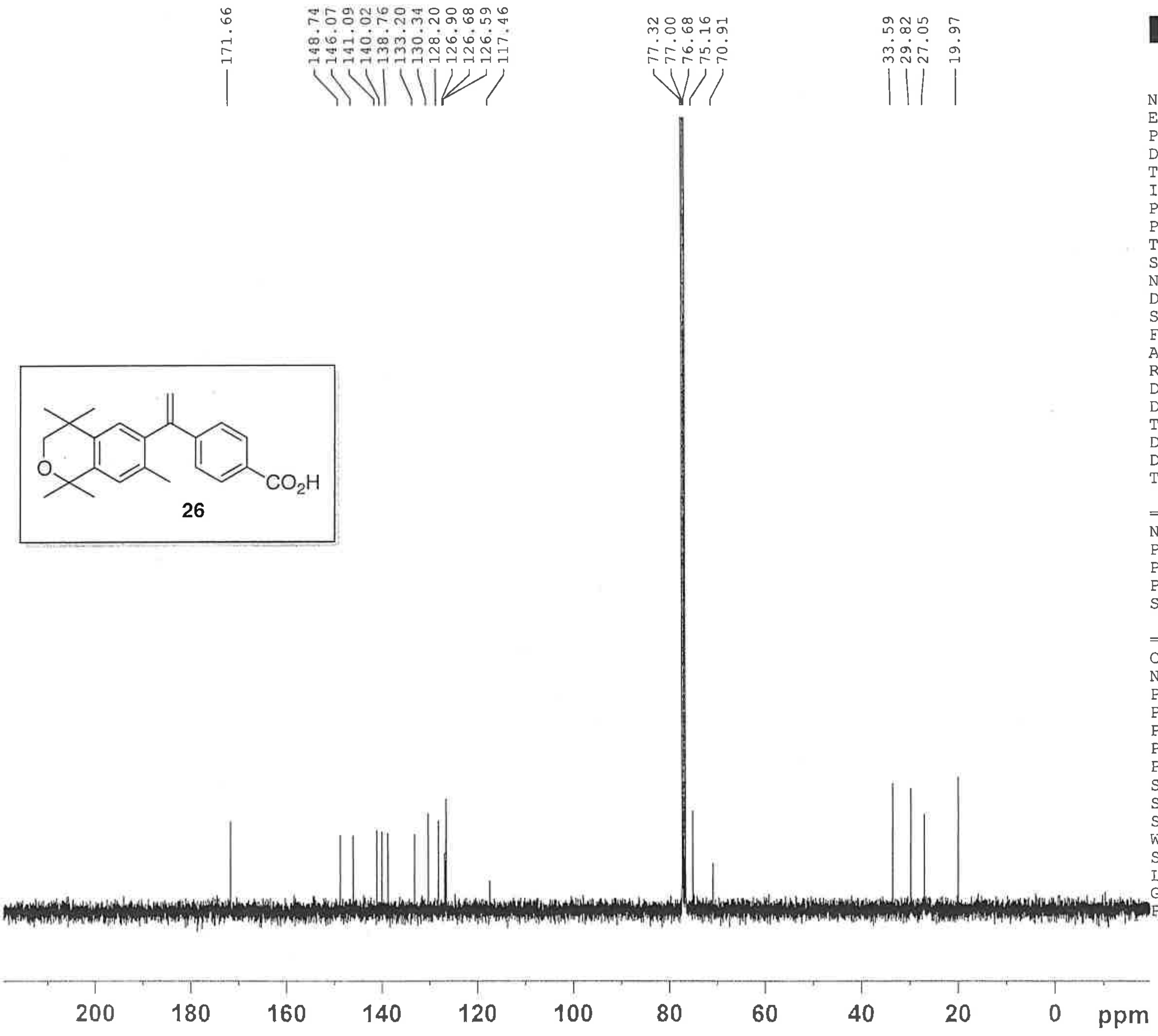

26

NAME	CWVI-141
EXPNO	2
PROCNO	1
Date_	20180711
Time	14.33
INSTRUM	spect
PROBHD	5 mm PABBO BB-
PULPROG	zgig30
TD	65536
SOLVENT	CDCl3
NS	129
DS	4
SWH	24038.461 Hz
FIDRES	0.366798 Hz
AQ	1.3631988 sec
RG	2050
DW	20.800 usec
DE	6.50 usec
TE	294.8 K
D1	2.00000000 sec
D11	0.03000000 sec
TD0	1

======== CHANNEL f1 ========

NUC1	13C
P1	8.25 usec
PL1	-2.10 dB
PL1W	60.29227829 W
SFO1	100.6228298 MHz

======== CHANNEL f2 ========

CPDPRG2	waltz16
NUC2	1H
PCPD2	90.00 usec
PL2	0.50 dB
PL12	16.21 dB
PL2W	12.76071072 W
PL12W	0.34266910 W
SFO2	400.1316005 MHz
SI	32768
SF	100.6127722 MHz
WDW	EM
SSB	0
LB	1.00 Hz
GB	0
PC	1.40

CWVI-143

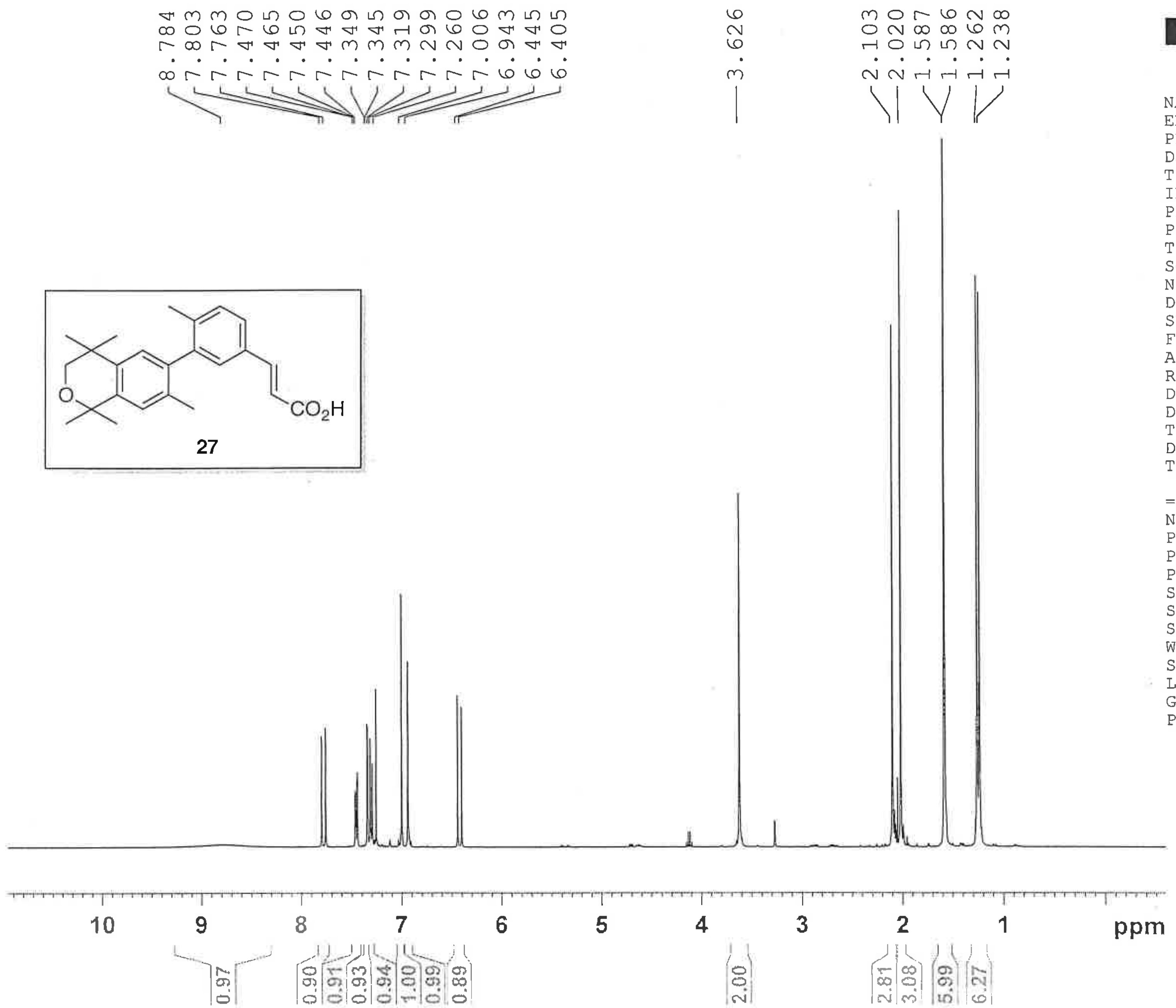

BRUKER

NAME	CWVI-143	
EXPNO	1	
PROCNO	1	
Date_	20181009	
Time	10.37	
INSTRUM	spect	
PROBHD	5 mm PABBO BB-	
PULPROG	zg30	
TD	65536	
SOLVENT	CDCl3	
NS	16	
DS	2	
SWH	8223.685	Hz
FIDRES	0.125483	Hz
AQ	3.9846387	sec
RG	4	
DW	60.800	usec
DE	6.50	usec
TE	291.9	K
D1	1.00000000	sec
TD0	1	

======== CHANNEL f1 ========

NUC1	1H	
P1	14.75	usec
PL1	0.50	dB
PL1W	12.76071072	W
SFO1	400.1324710	MHz
SI	32768	
SF	400.1300099	MHz
WDW	EM	
SSB	0	
LB	0.30	Hz
GB	0	
PC	1.00	

CWVI-143

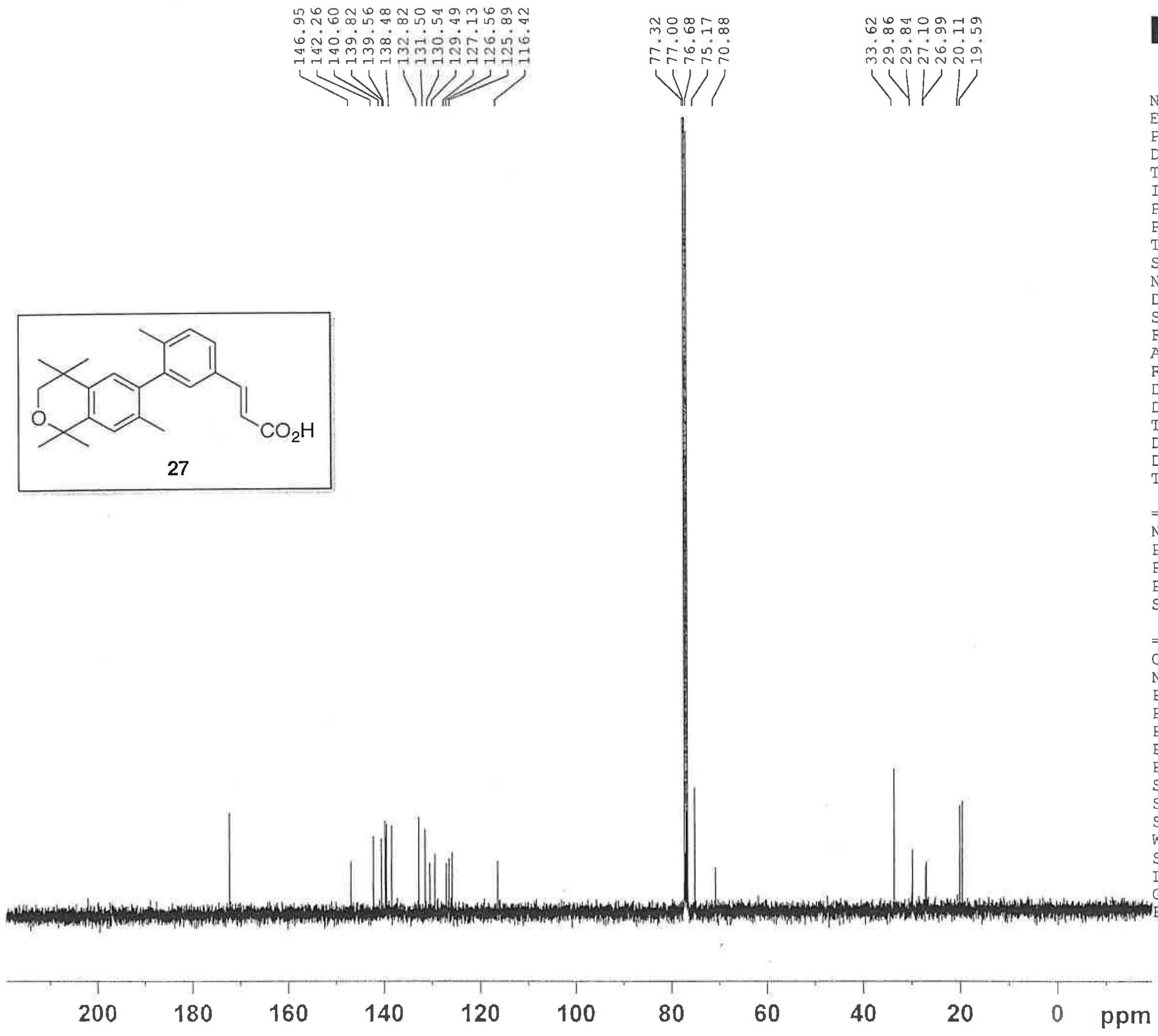

27

NAME	CWVI-143
EXPNO	2
PROCNO	1
Date_	20181009
Time	10.43
INSTRUM	spect
PROBHD	5 mm PABBO BB-
PULPROG	zgig30
TD	65536
SOLVENT	CDCl3
NS	141
DS	4
SWH	24038.461 Hz
FIDRES	0.366798 Hz
AQ	1.3631988 sec
RG	2050
DW	20.800 usec
DE	6.50 usec
TE	292.2 K
D1	2.00000000 sec
D11	0.03000000 sec
TD0	1

======== CHANNEL f1 ========

NUC1	13C
P1	8.25 usec
PL1	-2.10 dB
PL1W	60.29227829 W
SFO1	100.6228298 MHz

======== CHANNEL f2 ========

CPDPRG2	waltz16
NUC2	1H
PCPD2	90.00 usec
PL2	0.50 dB
PL12	16.21 dB
PL2W	12.76071072 W
PL12W	0.34266910 W
SFO2	400.1316005 MHz
SI	32768
SF	100.6127736 MHz
WDW	EM
SSB	0
LB	1.00 Hz
GB	0
PC	1.40

CWVI-145

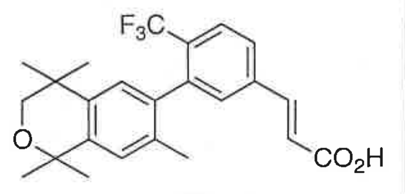

28

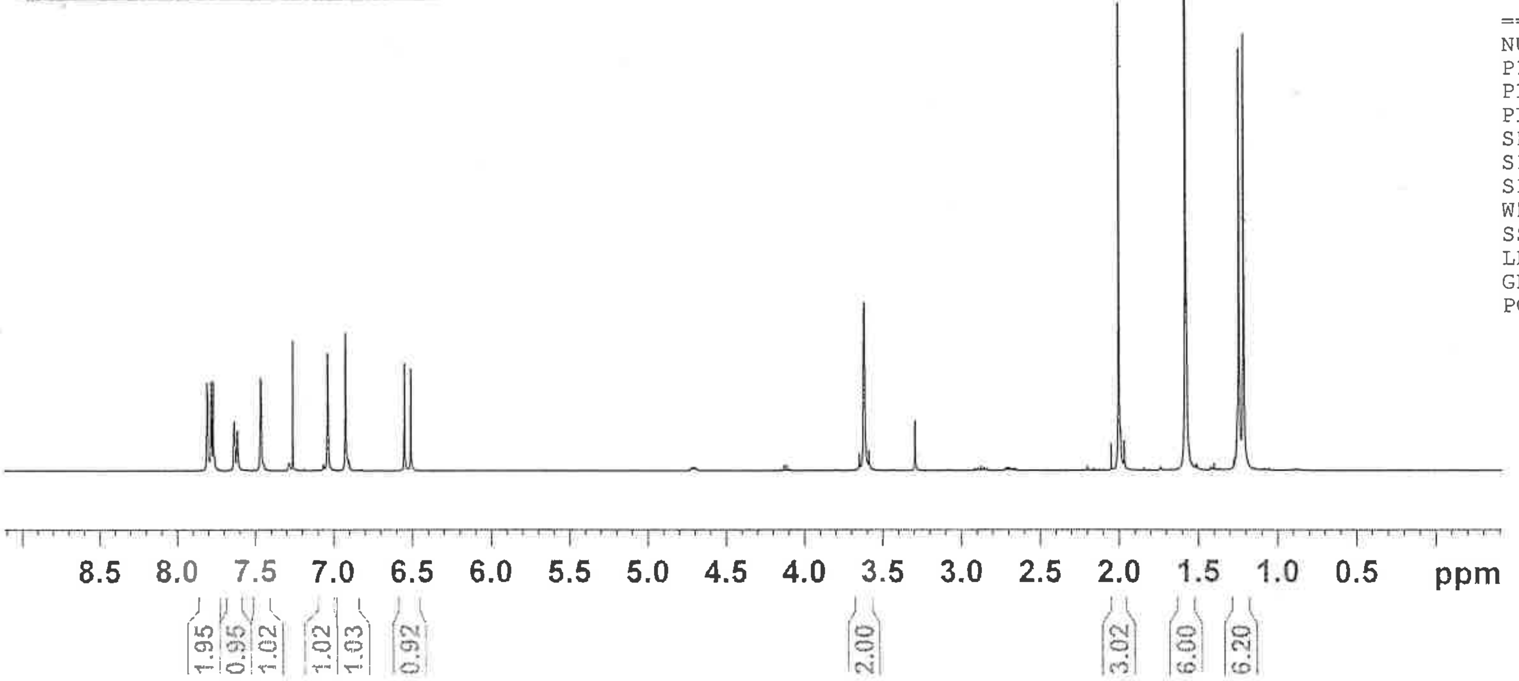

NAME	CWVI-145
EXPNO	1
PROCNO	1
Date_	20181009
Time	10.52
INSTRUM	spect
PROBHD	5 mm PABBO BB-
PULPROG	zg30
TD	65536
SOLVENT	CDCl3
NS	16
DS	2
SWH	8223.685 Hz
FIDRES	0.125483 Hz
AQ	3.9846387 sec
RG	4
DW	60.800 usec
DE	6.50 usec
TE	291.8 K
D1	1.00000000 sec
TD0	1

======== CHANNEL f1 ========

NUC1	1H
P1	14.75 usec
PL1	0.50 dB
PL1W	12.76071072 W
SFO1	400.1324710 MHz
SI	32768
SF	400.1300099 MHz
WDW	EM
SSB	0
LB	0.30 Hz
GB	0
PC	1.00

CWVI-145

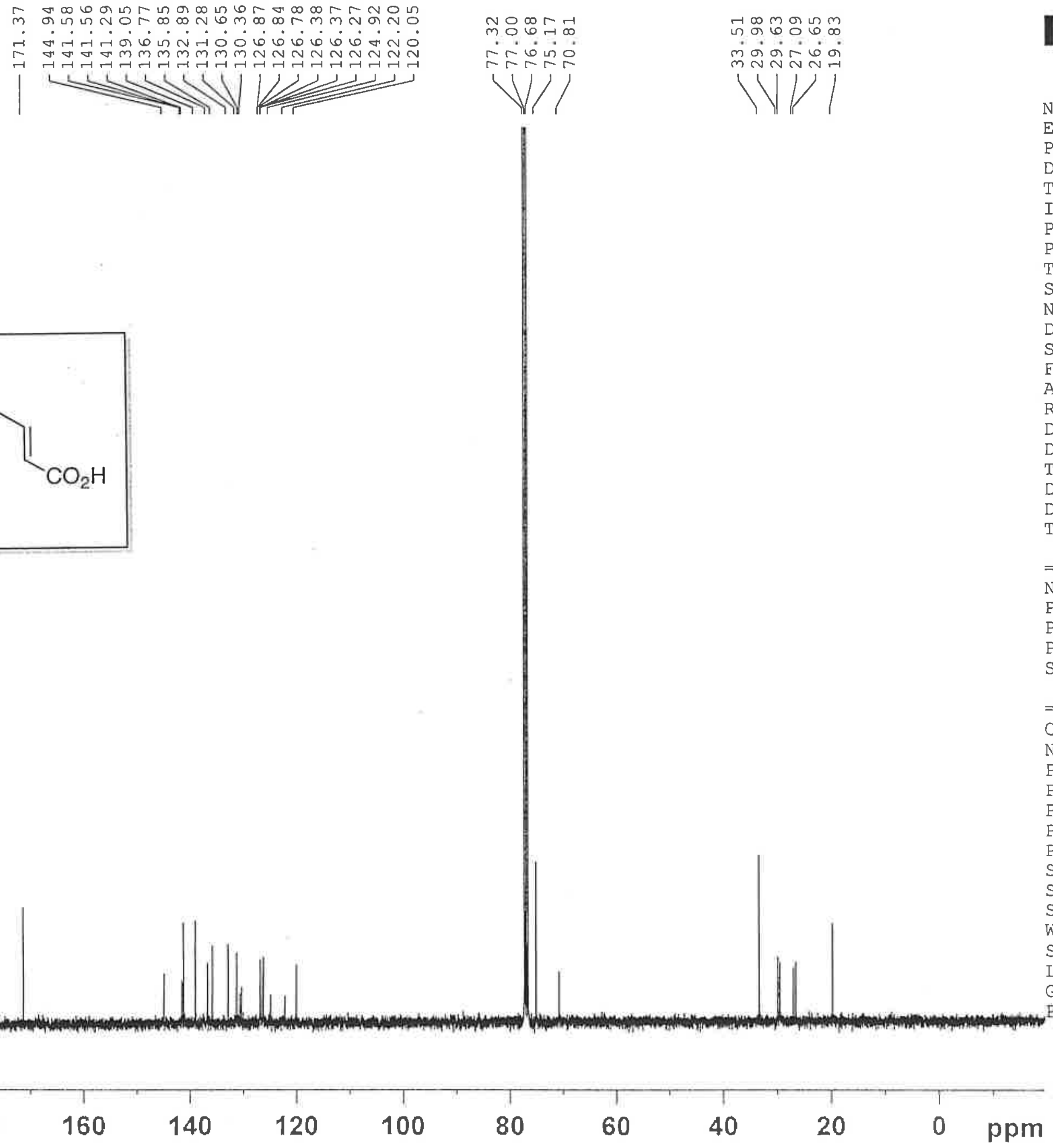

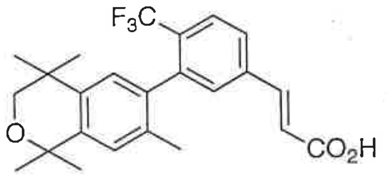

28

BRUKER

NAME	CWVI-145
EXPNO	2
PROCNO	1
Date_	20181009
Time	11.14
INSTRUM	spect
PROBHD	5 mm PABBO BB-
PULPROG	zgig30
TD	65536
SOLVENT	CDCl3
NS	478
DS	4
SWH	24038.461 Hz
FIDRES	0.366798 Hz
AQ	1.3631988 sec
RG	2050
DW	20.800 usec
DE	6.50 usec
TE	292.2 K
D1	2.00000000 sec
D11	0.03000000 sec
TD0	1
======== CHANNEL f1 ========	
NUC1	13C
P1	8.25 usec
PL1	-2.10 dB
PL1W	60.29227829 W
SFO1	100.6228298 MHz
======== CHANNEL f2 ========	
CPDPRG2	waltz16
NUC2	1H
PCPD2	90.00 usec
PL2	0.50 dB
PL12	16.21 dB
PL2W	12.76071072 W
PL12W	0.34266910 W
SFO2	400.1316005 MHz
SI	32768
SF	100.6127729 MHz
WDW	EM
SSB	0
LB	1.00 Hz
GB	0
PC	1.40

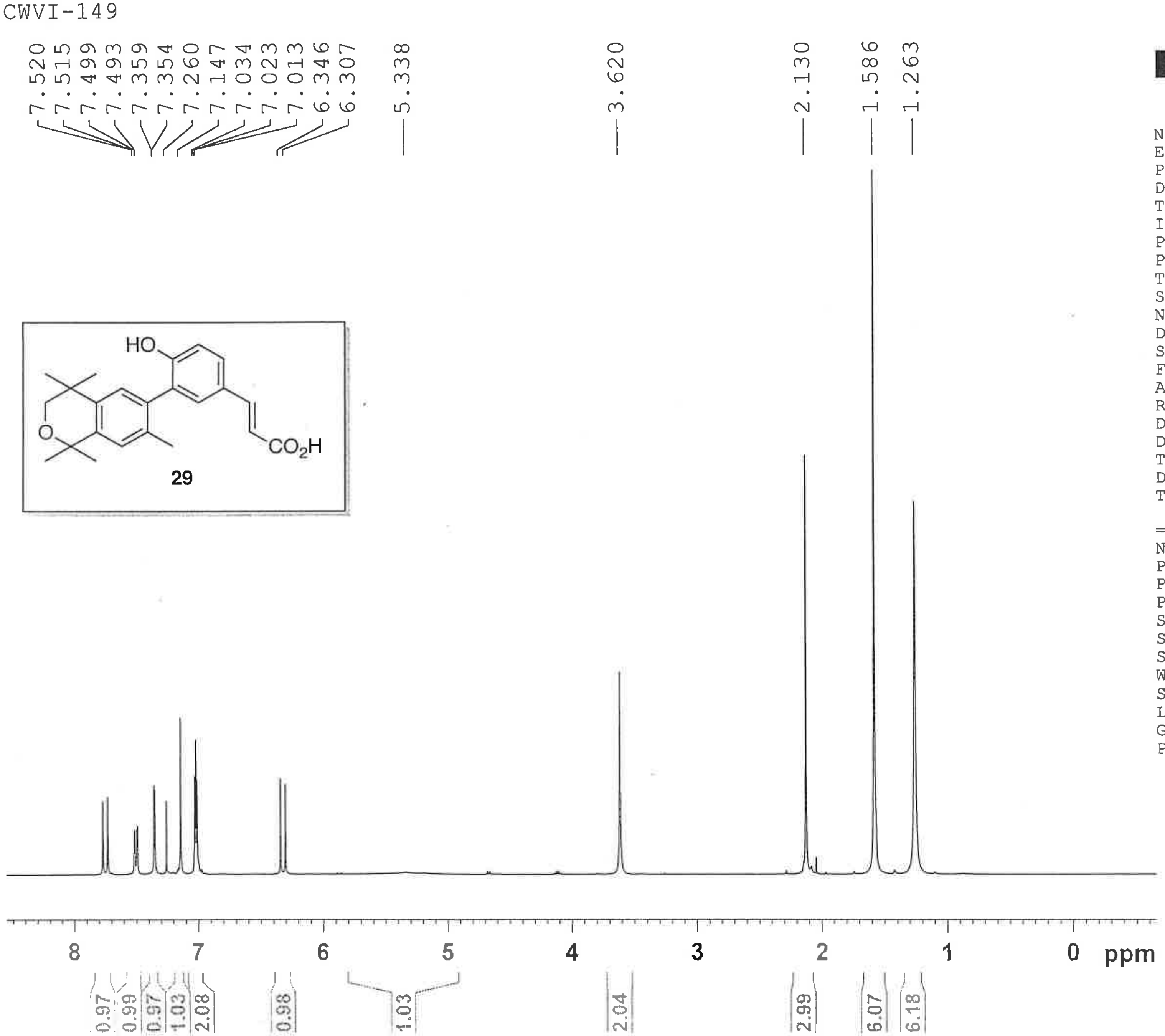

NAME	CWVI-149	
EXPNO	3	
PROCNO	1	
Date_	20181023	
Time_	12.35	
INSTRUM	spect	
PROBHD	5 mm PABBO BB-	
PULPROG	zg30	
TD	65536	
SOLVENT	CDCl3	
NS	16	
DS	2	
SWH	8223.685	Hz
FIDRES	0.125483	Hz
AQ	3.9846387	sec
RG	4	
DW	60.800	usec
DE	6.50	usec
TE	292.1	K
D1	1.00000000	sec
TD0	1	
======== CHANNEL f1 ========		
NUC1	1H	
P1	14.75	usec
PL1	0.50	dB
PL1W	12.76071072	W
SFO1	400.1324710	MHz
SI	32768	
SF	400.1300099	MHz
WDW	EM	
SSB	0	
LB	0.30	Hz
GB	0	
PC	1.00	

CWVI-149

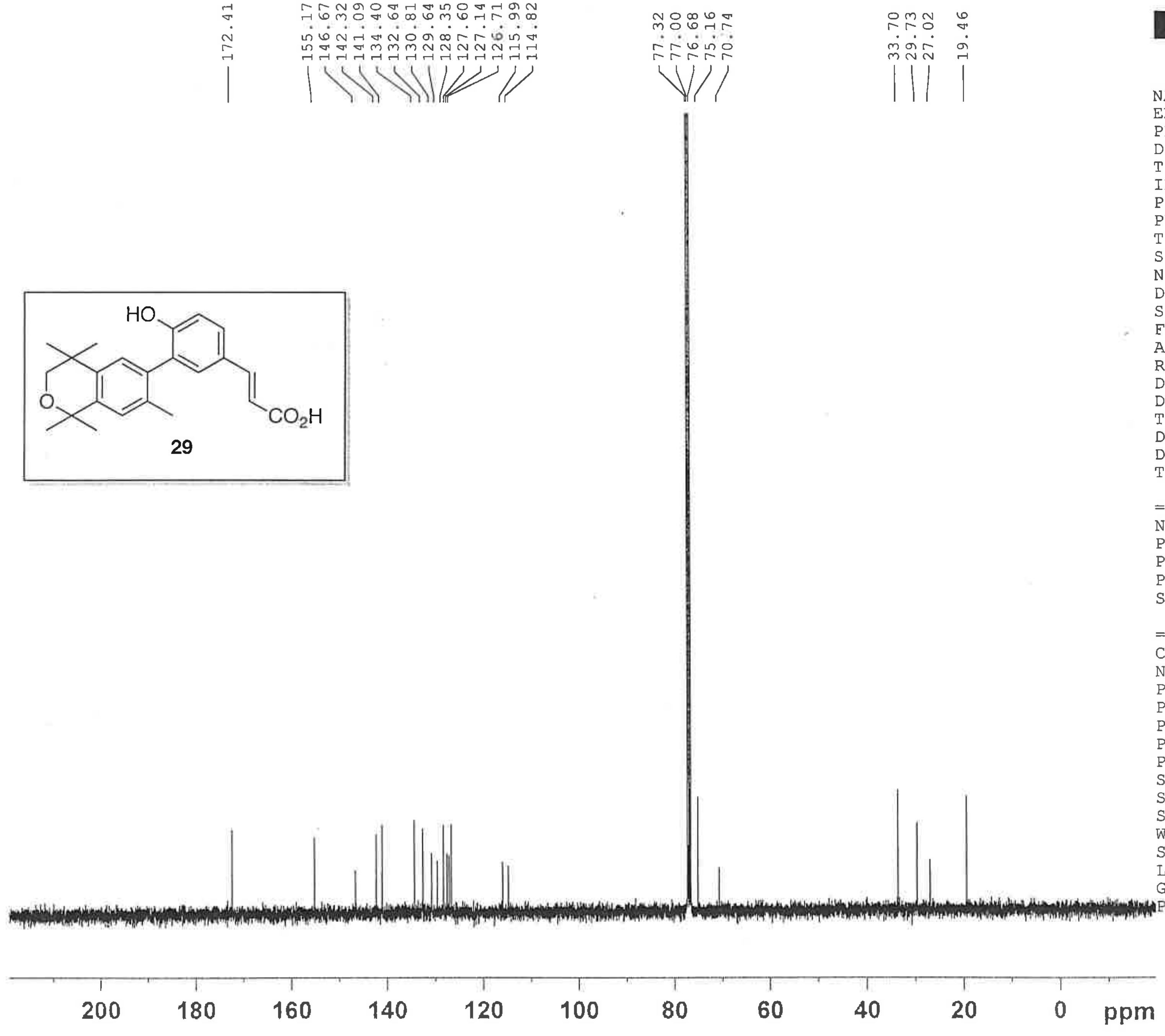

29

NAME	CWVI-149	
EXPNO	4	
PROCNO	1	
Date_	20181023	
Time	12.43	
INSTRUM	spect	
PROBHD	5 mm PABBO BB-	
PULPROG	zgig30	
TD	65536	
SOLVENT	CDCl3	
NS	213	
DS	4	
SWH	24038.461	Hz
FIDRES	0.366798	Hz
AQ	1.3631988	sec
RG	2050	
DW	20.800	usec
DE	6.50	usec
TE	292.4	K
D1	2.00000000	sec
D11	0.03000000	sec
TD0	1	
======== CHANNEL f1 ========		
NUC1	13C	
P1	8.25	usec
PL1	-2.10	dB
PL1W	60.29227829	W
SFO1	100.6228298	MHz
======== CHANNEL f2 ========		
CPDPRG2	waltz16	
NUC2	1H	
PCPD2	90.00	usec
PL2	0.50	dB
PL12	16.21	dB
PL2W	12.76071072	W
PL12W	0.34266910	W
SFO2	400.1316005	MHz
SI	32768	
SF	100.6127736	MHz
WDW	EM	
SSB	0	
LB	1.00	Hz
GB	0	
PC	1.40	

CWVI-151

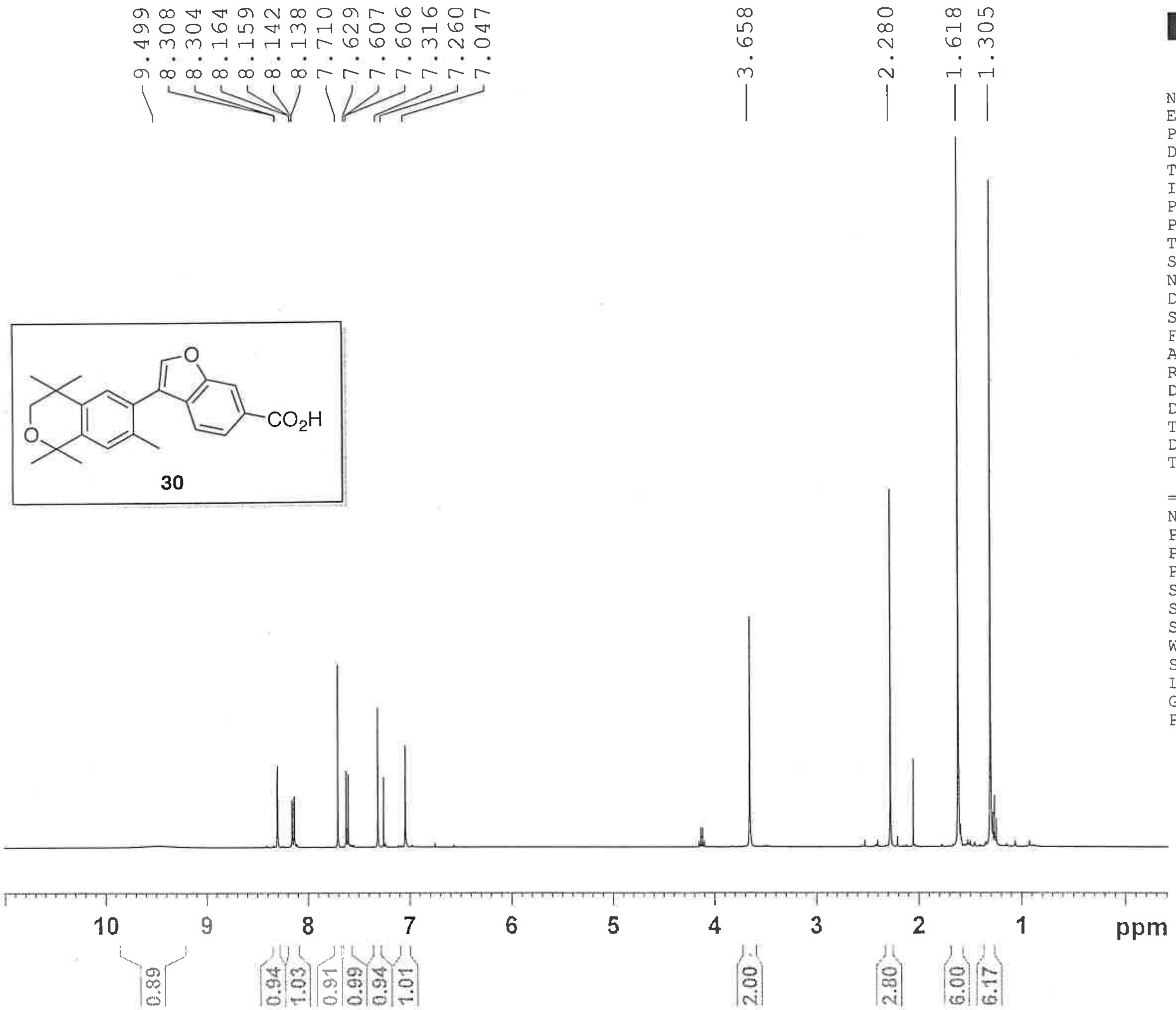

30

BRUKER

NAME	CWVI-151
EXPNO	1
PROCNO	1
Date_	20181009
Time	12.10
INSTRUM	spect
PROBHD	5 mm PABBO BB-
PULPROG	zg30
TD	65536
SOLVENT	CDCl3
NS	16
DS	2
SWH	8223.685 Hz
FIDRES	0.125483 Hz
AQ	3.9846387 sec
RG	4
DW	60.800 usec
DE	6.50 usec
TE	292.0 K
D1	1.00000000 sec
TD0	1
======== CHANNEL f1 ========	
NUC1	1H
P1	14.75 usec
PL1	0.50 dB
PL1W	12.76071072 W
SFO1	400.1324710 MHz
SI	32768
SF	400.1300099 MHz
WDW	EM
SSB	0
LB	0.30 Hz
GB	0
PC	1.00

CWVI-151

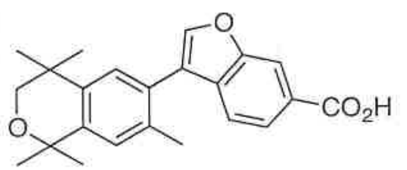

30

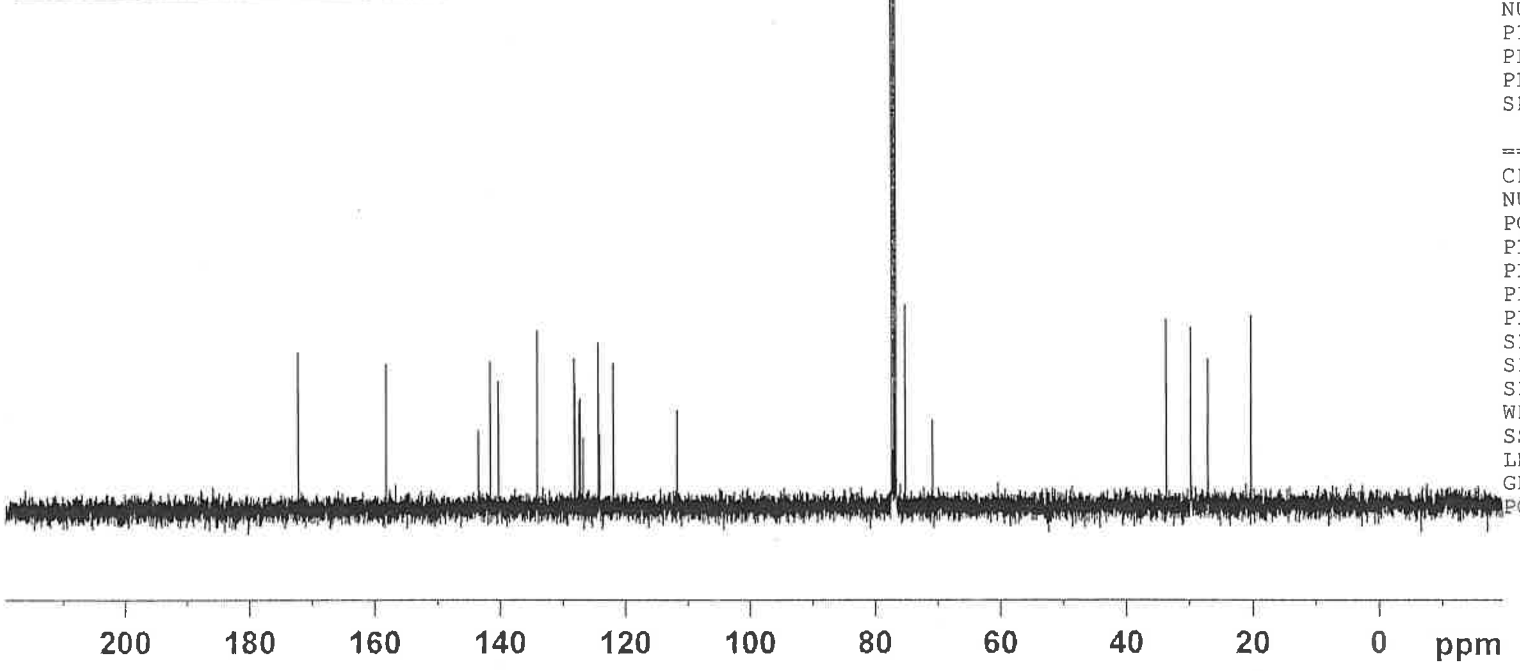

NAME	CWVI-151
EXPNO	2
PROCNO	1
Date_	20181009
Time	12.14
INSTRUM	spect
PROBHD	5 mm PABBO BB-
PULPROG	zgig30
TD	65536
SOLVENT	CDCl3
NS	117
DS	4
SWH	24038.461 Hz
FIDRES	0.366798 Hz
AQ	1.3631988 sec
RG	2050
DW	20.800 usec
DE	6.50 usec
TE	292.3 K
D1	2.00000000 sec
D11	0.03000000 sec
TD0	1
======== CHANNEL f1 ========	
NUC1	13C
P1	8.25 usec
PL1	-2.10 dB
PL1W	60.29227829 W
SFO1	100.6228298 MHz
======== CHANNEL f2 ========	
CPDPRG2	waltz16
NUC2	1H
PCPD2	90.00 usec
PL2	0.50 dB
PL12	16.21 dB
PL2W	12.76071072 W
PL12W	0.34266910 W
SFO2	400.1316005 MHz
SI	32768
SF	100.6127736 MHz
WDW	EM
SSB	0
LB	1.00 Hz
GB	0
PC	1.40

CWVI-153

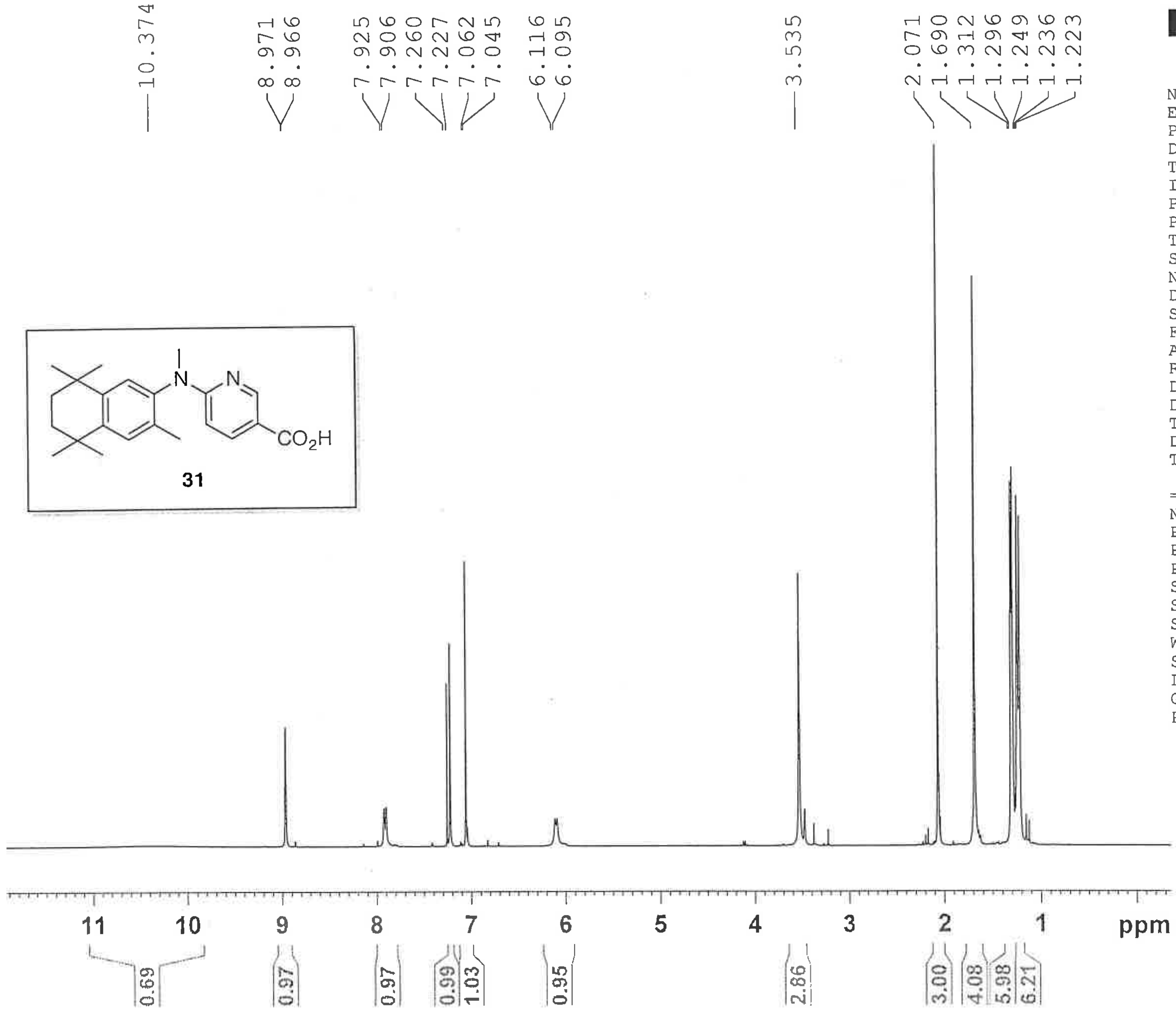

31

BRUKER

NAME	CWVI-153	
EXPNO	1	
PROCNO	1	
Date_	20181009	
Time	12.23	
INSTRUM	spect	
PROBHD	5 mm PABBO BB-	
PULPROG	zg30	
TD	65536	
SOLVENT	CDCl3	
NS	16	
DS	2	
SWH	8223.685	Hz
FIDRES	0.125483	Hz
AQ	3.9846387	sec
RG	4	
DW	60.800	usec
DE	6.50	usec
TE	292.0	K
D1	1.00000000	sec
TD0	1	

======== CHANNEL f1 ========

NUC1	1H	
P1	14.75	usec
PL1	0.50	dB
PL1W	12.76071072	W
SFO1	400.1324710	MHz
SI	32768	
SF	400.1300099	MHz
WDW	EM	
SSB	0	
LB	0.30	Hz
GB	0	
PC	1.00	

CWVI-153

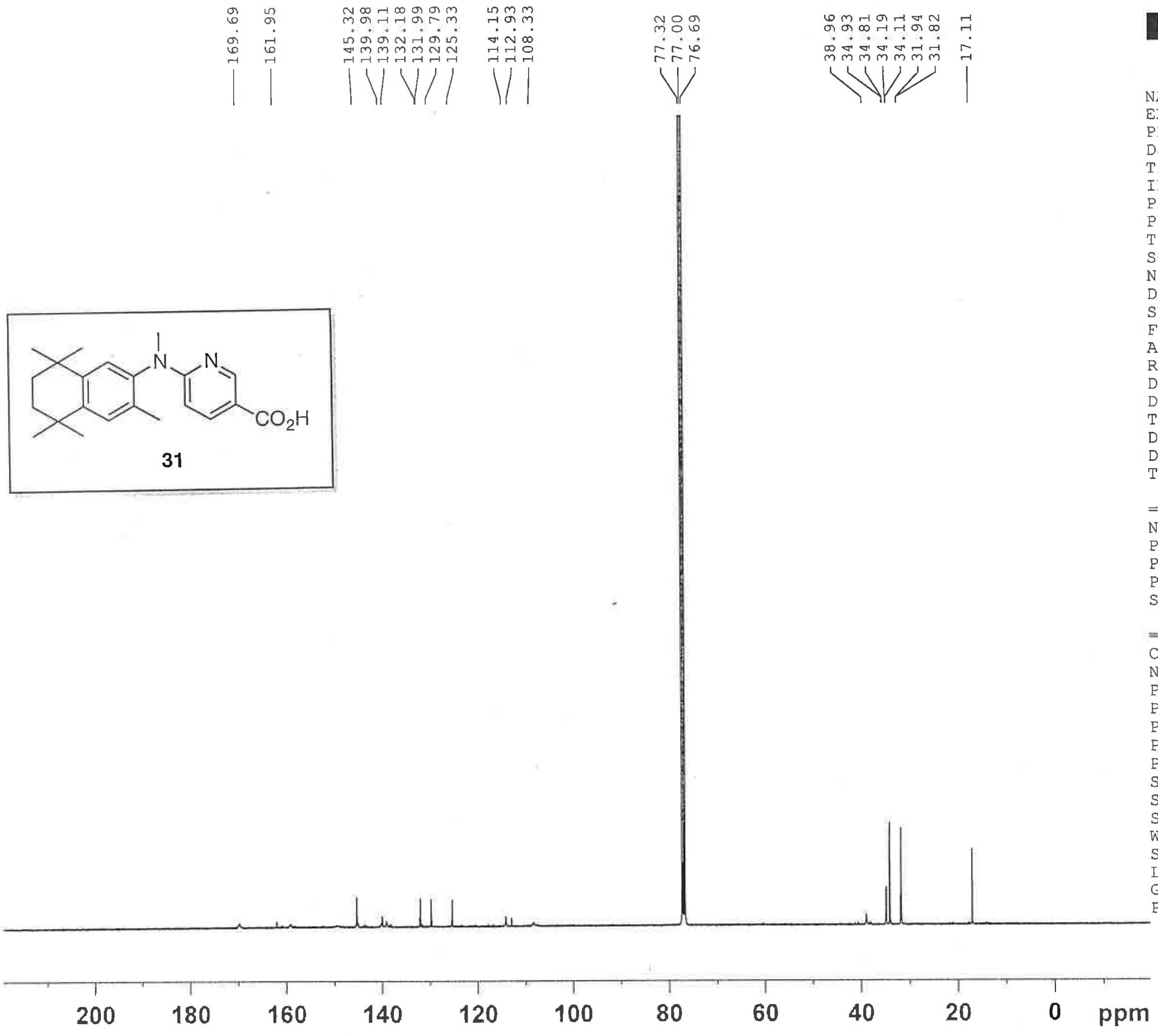

31

BRUKER

NAME	CWVI-153
EXPNO	5
PROCNO	1
Date_	20181010
Time_	5.34
INSTRUM	spect
PROBHD	5 mm PABBO BB-
PULPROG	zgig30
TD	65536
SOLVENT	CDCl3
NS	16384
DS	4
SWH	24038.461 Hz
FIDRES	0.366798 Hz
AQ	1.3631988 sec
RG	2050
DW	20.800 usec
DE	6.50 usec
TE	292.3 K
D1	2.00000000 sec
D11	0.03000000 sec
TD0	1
======== CHANNEL f1 ========	
NUC1	13C
P1	8.25 usec
PL1	-2.10 dB
PL1W	60.29227829 W
SFO1	100.6228298 MHz
======== CHANNEL f2 ========	
CPDPRG2	waltz16
NUC2	1H
PCPD2	90.00 usec
PL2	0.50 dB
PL12	16.21 dB
PL2W	12.76071072 W
PL12W	0.34266910 W
SFO2	400.1316005 MHz
SI	32768
SF	100.6127736 MHz
WDW	EM
SSB	0
LB	1.00 Hz
GB	0
PC	1.40

CWVI-173

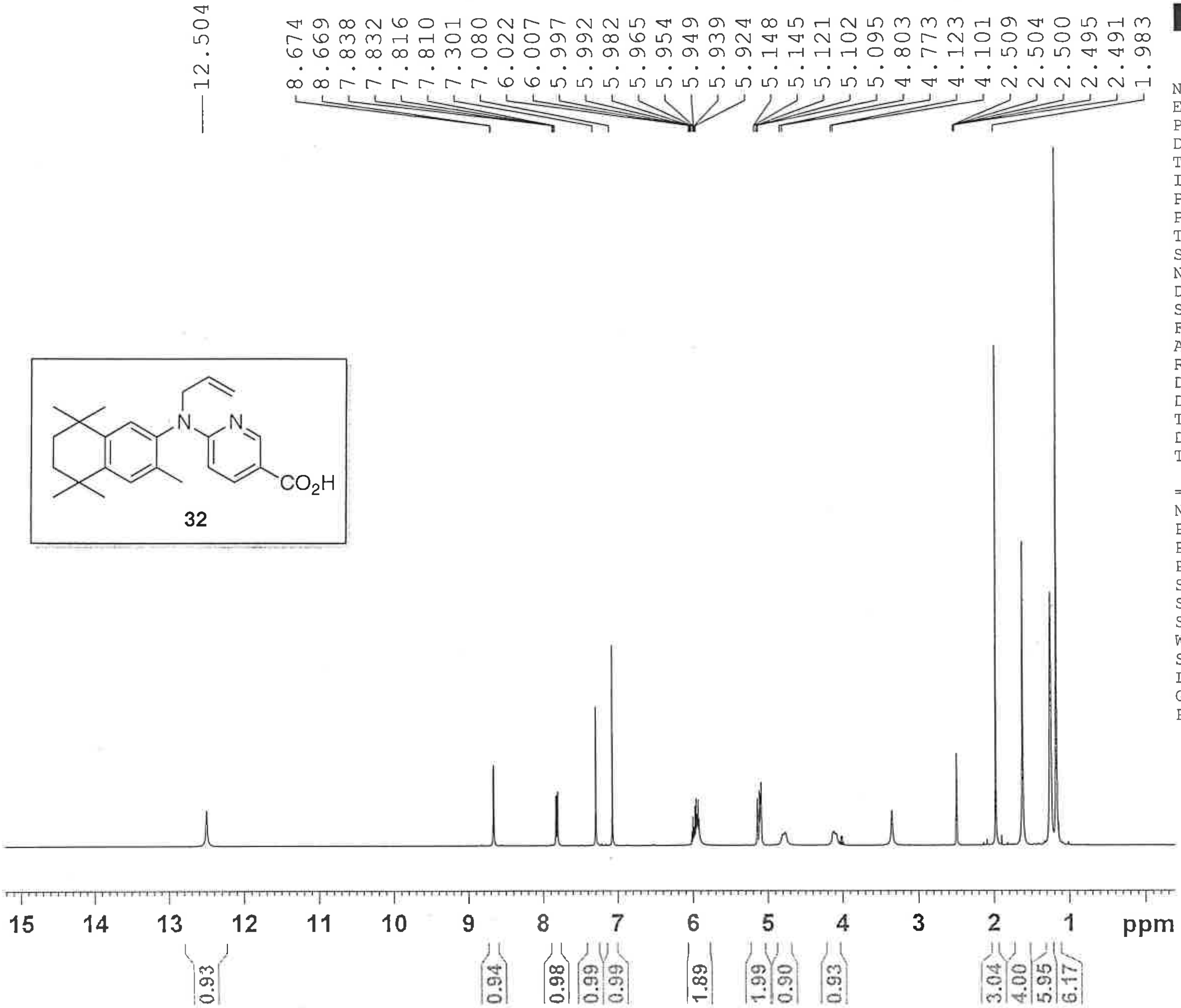

BRUKER

NAME	CWVI-173
EXPNO	1
PROCNO	1
Date_	20181010
Time_	13.45
INSTRUM	spect
PROBHD	5 mm PABBO BB-
PULPROG	zg30
TD	65536
SOLVENT	DMSO
NS	16
DS	2
SWH	8223.685 Hz
FIDRES	0.125483 Hz
AQ	3.9846387 sec
RG	4
DW	60.800 usec
DE	6.50 usec
TE	292.2 K
D1	1.00000000 sec
TD0	1

======== CHANNEL f1 ========

NUC1	1H
P1	14.75 usec
PL1	0.50 dB
PL1W	12.76071072 W
SFO1	400.1324710 MHz
SI	32768
SF	400.1300034 MHz
WDW	EM
SSB	0
LB	0.30 Hz
GB	0
PC	1.00

CWVI-173

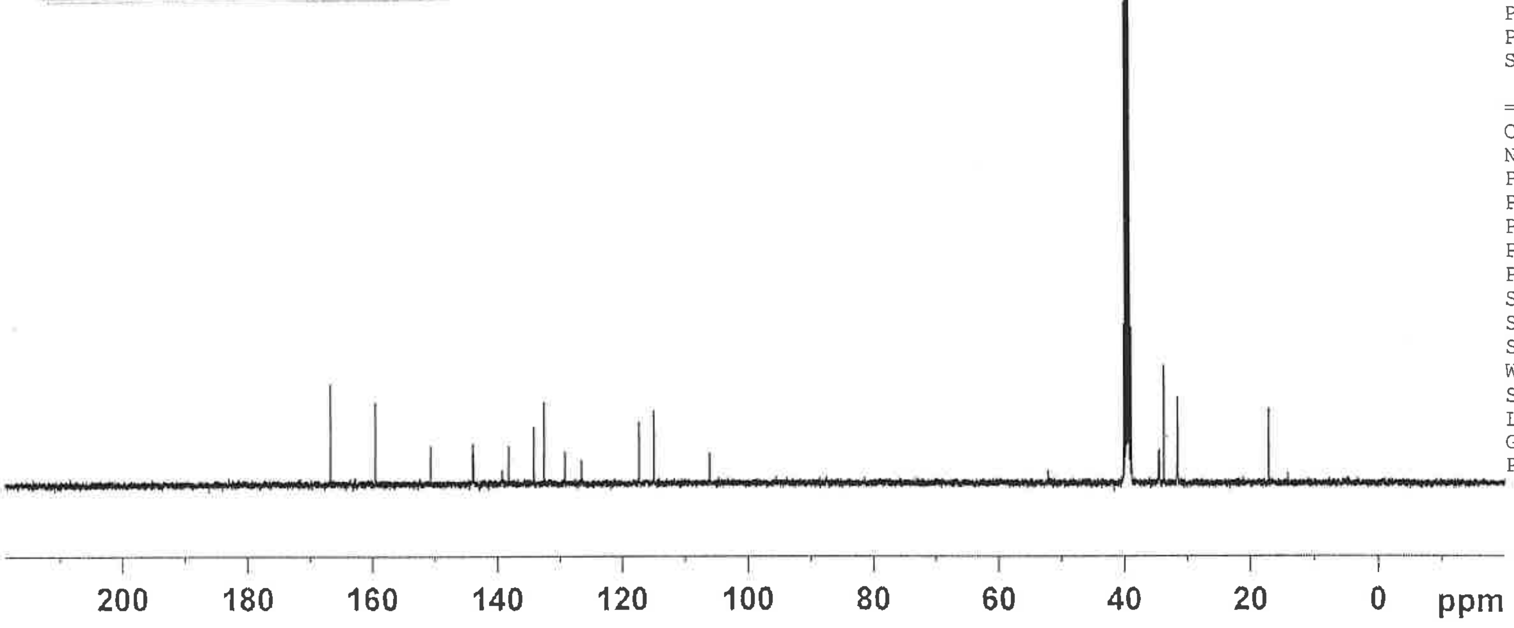

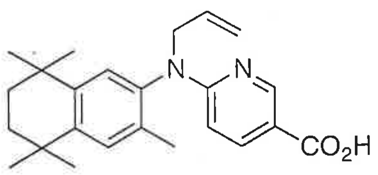

32

BRUKER

NAME	CWVI-173	
EXPNO	2	
PROCNO	1	
Date_	20181010	
Time	13.52	
INSTRUM	spect	
PROBHD	5 mm PABBO BB-	
PULPROG	zgig30	
TD	65536	
SOLVENT	DMSO	
NS	426	
DS	4	
SWH	24038.461	Hz
FIDRES	0.366798	Hz
AQ	1.3631988	sec
RG	2050	
DW	20.800	usec
DE	6.50	usec
TE	292.4	K
D1	2.00000000	sec
D11	0.03000000	sec
TD0	1	

======== CHANNEL f1 ========

NUC1	13C	
P1	8.25	usec
PL1	-2.10	dB
PL1W	60.29227829	W
SFO1	100.6228298	MHz

======== CHANNEL f2 ========

CPDPRG2	waltz16	
NUC2	1H	
PCPD2	90.00	usec
PL2	0.50	dB
PL12	16.21	dB
PL2W	12.76071072	W
PL12W	0.34266910	W
SFO2	400.1316005	MHz
SI	32768	
SF	100.6128121	MHz
WDW	EM	
SSB	0	
LB	1.00	Hz
GB	0	
PC	1.40	

CWVI-163

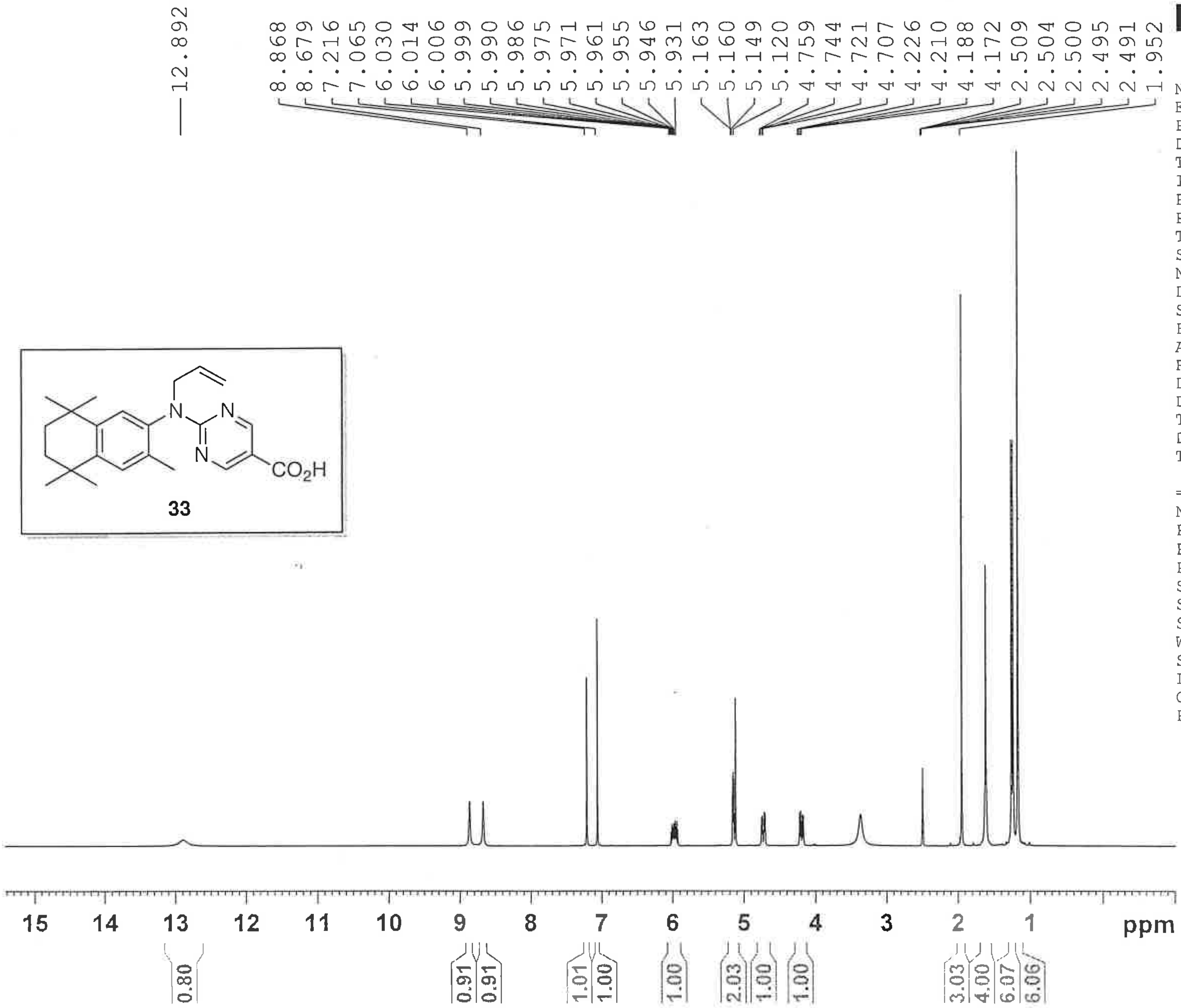

BRUKER

NAME	CWVI-163
EXPNO	1
PROCNO	1
Date_	20181010
Time	10.51
INSTRUM	spect
PROBHD	5 mm PABBO BB-
PULPROG	zg30
TD	65536
SOLVENT	DMSO
NS	16
DS	2
SWH	8223.685 Hz
FIDRES	0.125483 Hz
AQ	3.9846387 sec
RG	4
DW	60.800 usec
DE	6.50 usec
TE	292.0 K
D1	1.00000000 sec
TD0	1

======== CHANNEL f1 ========

NUC1	1H
P1	14.75 usec
PL1	0.50 dB
PL1W	12.76071072 W
SFO1	400.1324710 MHz
SI	32768
SF	400.1300034 MHz
WDW	EM
SSB	0
LB	0.30 Hz
GB	0
PC	1.00

CWVI-163

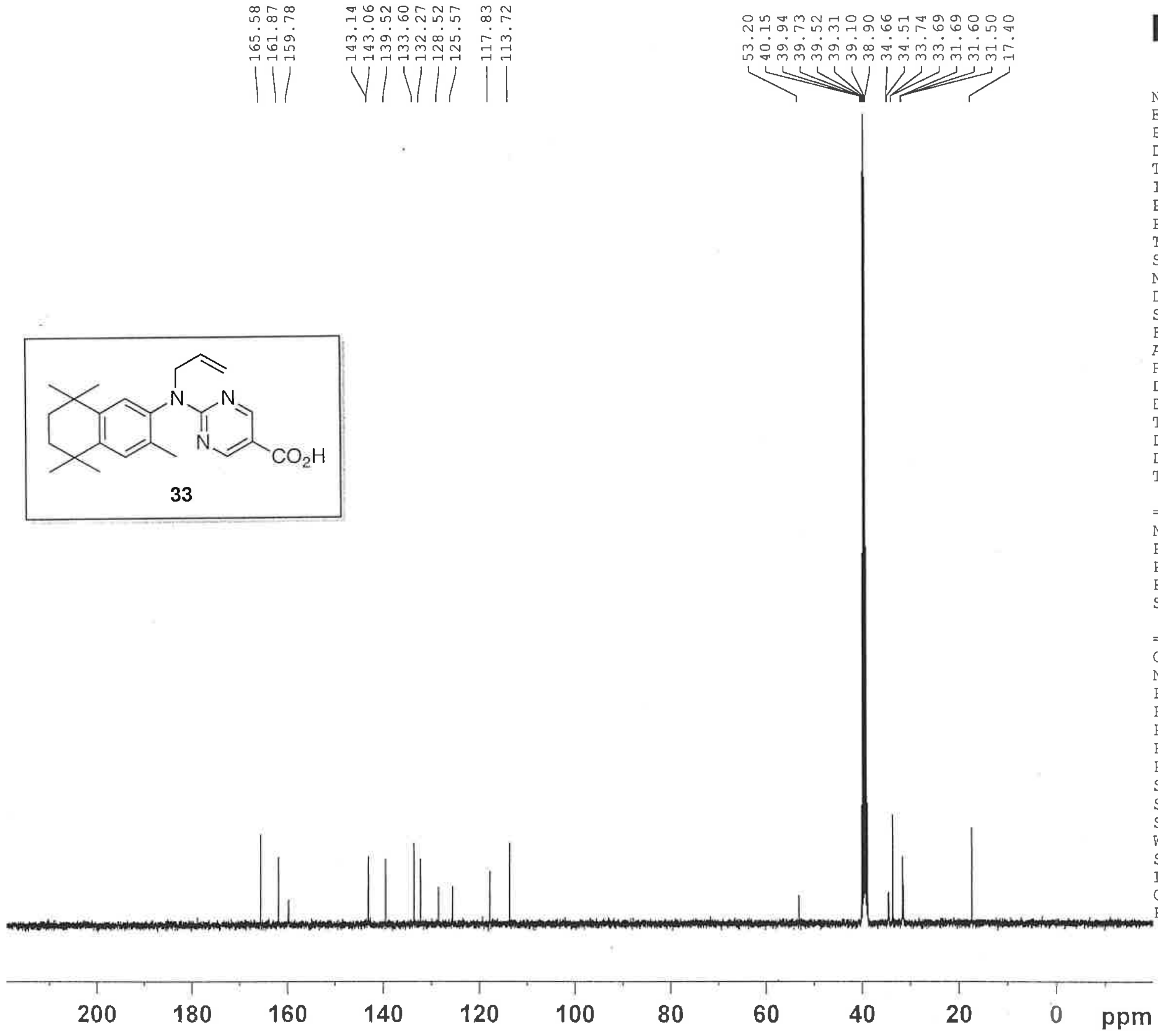

NAME	CWVI-163	
EXPNO	2	
PROCNO	1	
Date_	20181010	
Time	11.00	
INSTRUM	spect	
PROBHD	5 mm PABBO BB-	
PULPROG	zgig30	
TD	65536	
SOLVENT	DMSO	
NS	200	
DS	4	
SWH	24038.461	Hz
FIDRES	0.366798	Hz
AQ	1.3631988	sec
RG	2050	
DW	20.800	usec
DE	6.50	usec
TE	292.3	K
D1	2.00000000	sec
D11	0.03000000	sec
TD0	1	
======== CHANNEL f1 ========		
NUC1	13C	
P1	8.25	usec
PL1	-2.10	dB
PL1W	60.29227829	W
SFO1	100.6228298	MHz
======== CHANNEL f2 ========		
CPDPRG2	waltz16	
NUC2	1H	
PCPD2	90.00	usec
PL2	0.50	dB
PL12	16.21	dB
PL2W	12.76071072	W
PL12W	0.34266910	W
SFO2	400.1316005	MHz
SI	32768	
SF	100.6128106	MHz
WDW	EM	
SSB	0	
LB	1.00	Hz
GB	0	
PC	1.40	

CWVI-197

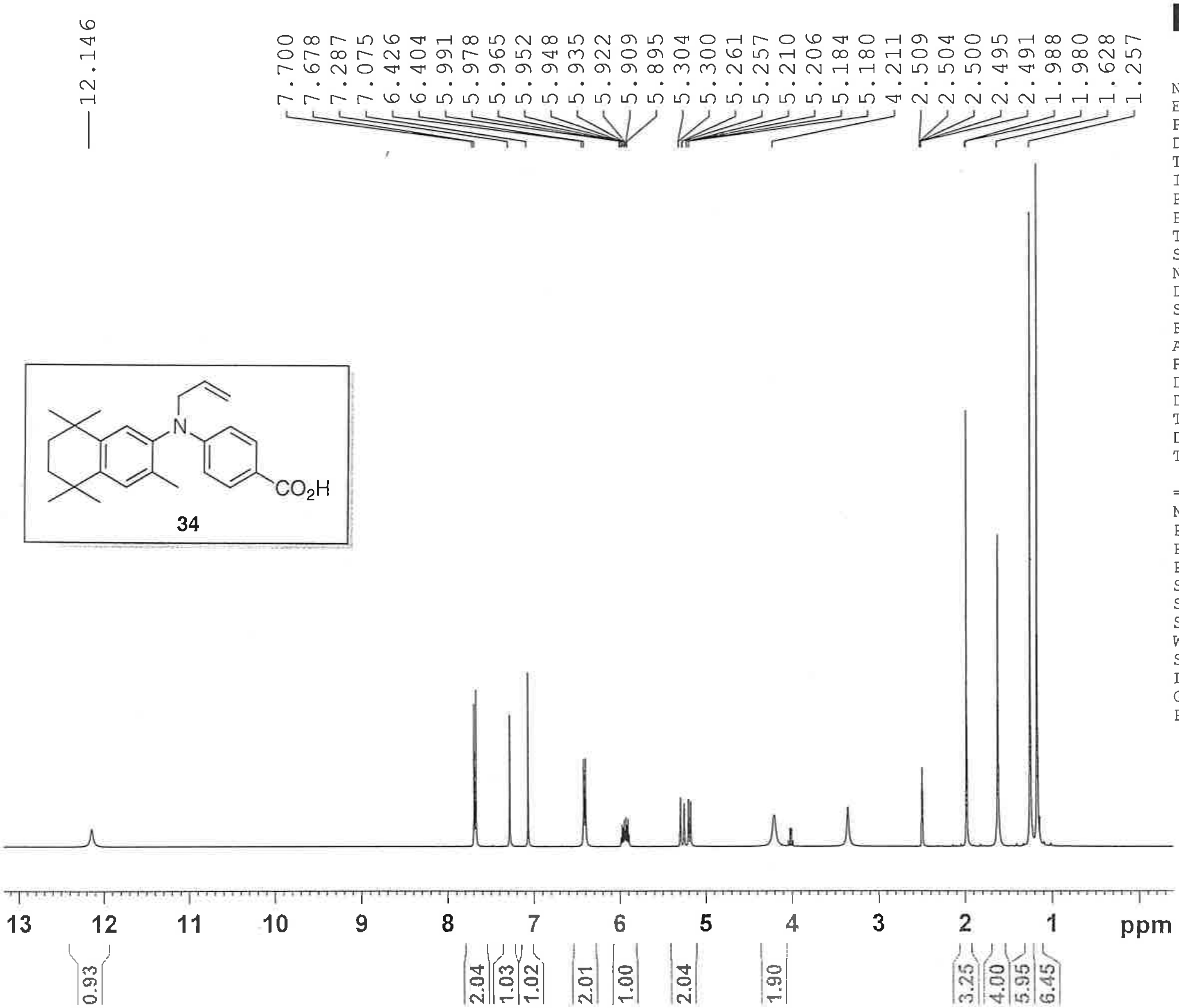

NAME	CWVI-197
EXPNO	1
PROCNO	1
Date_	20181010
Time_	20.29
INSTRUM	spect
PROBHD	5 mm PABBO BB-
PULPROG	zg30
TD	65536
SOLVENT	DMSO
NS	16
DS	2
SWH	8223.685 Hz
FIDRES	0.125483 Hz
AQ	3.9846387 sec
RG	4
DW	60.800 usec
DE	6.50 usec
TE	292.2 K
D1	1.00000000 sec
TD0	1

======== CHANNEL f1 ========

NUC1	1H
P1	14.75 usec
PL1	0.50 dB
PL1W	12.76071072 W
SFO1	400.1324710 MHz
SI	32768
SF	400.1300034 MHz
WDW	EM
SSB	0
LB	0.30 Hz
GB	0
PC	1.00

CWVI-197

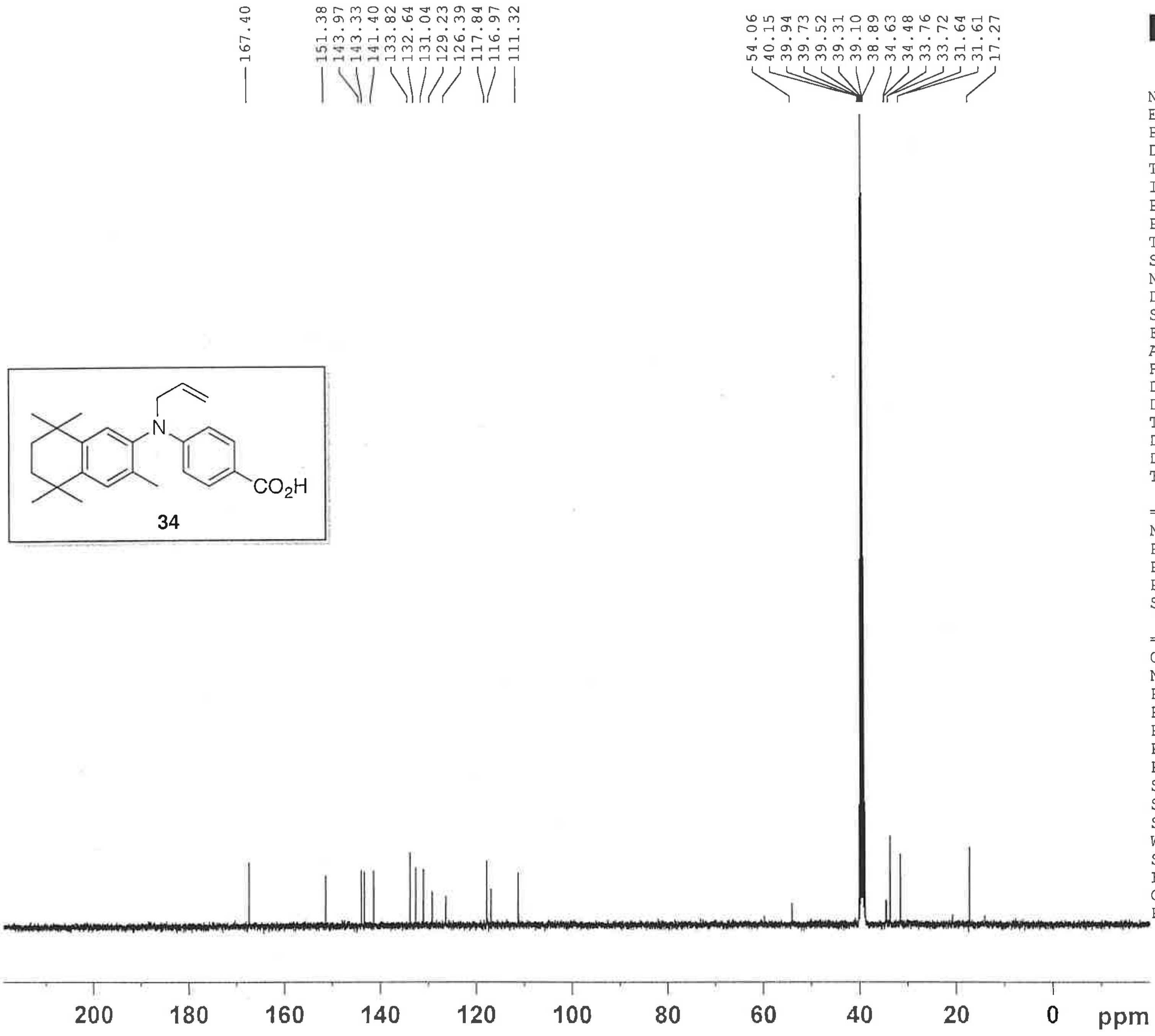

NAME	CWVI-197
EXPNO	2
PROCNO	1
Date_	20181010
Time	20.34
INSTRUM	spect
PROBHD	5 mm PABBO BB-
PULPROG	zgig30
TD	65536
SOLVENT	DMSO
NS	230
DS	4
SWH	24038.461 Hz
FIDRES	0.366798 Hz
AQ	1.3631988 sec
RG	2050
DW	20.800 usec
DE	6.50 usec
TE	292.4 K
D1	2.00000000 sec
D11	0.03000000 sec
TD0	1
======== CHANNEL f1 ========	
NUC1	13C
P1	8.25 usec
PL1	-2.10 dB
PL1W	60.29227829 W
SFO1	100.6228298 MHz
======== CHANNEL f2 ========	
CPDPRG2	waltz16
NUC2	1H
PCPD2	90.00 usec
PL2	0.50 dB
PL12	16.21 dB
PL2W	12.76071072 W
PL12W	0.34266910 W
SFO2	400.1316005 MHz
SI	32768
SF	100.6128121 MHz
WDW	EM
SSB	0
LB	1.00 Hz
GB	0
PC	1.40

CWVI-161

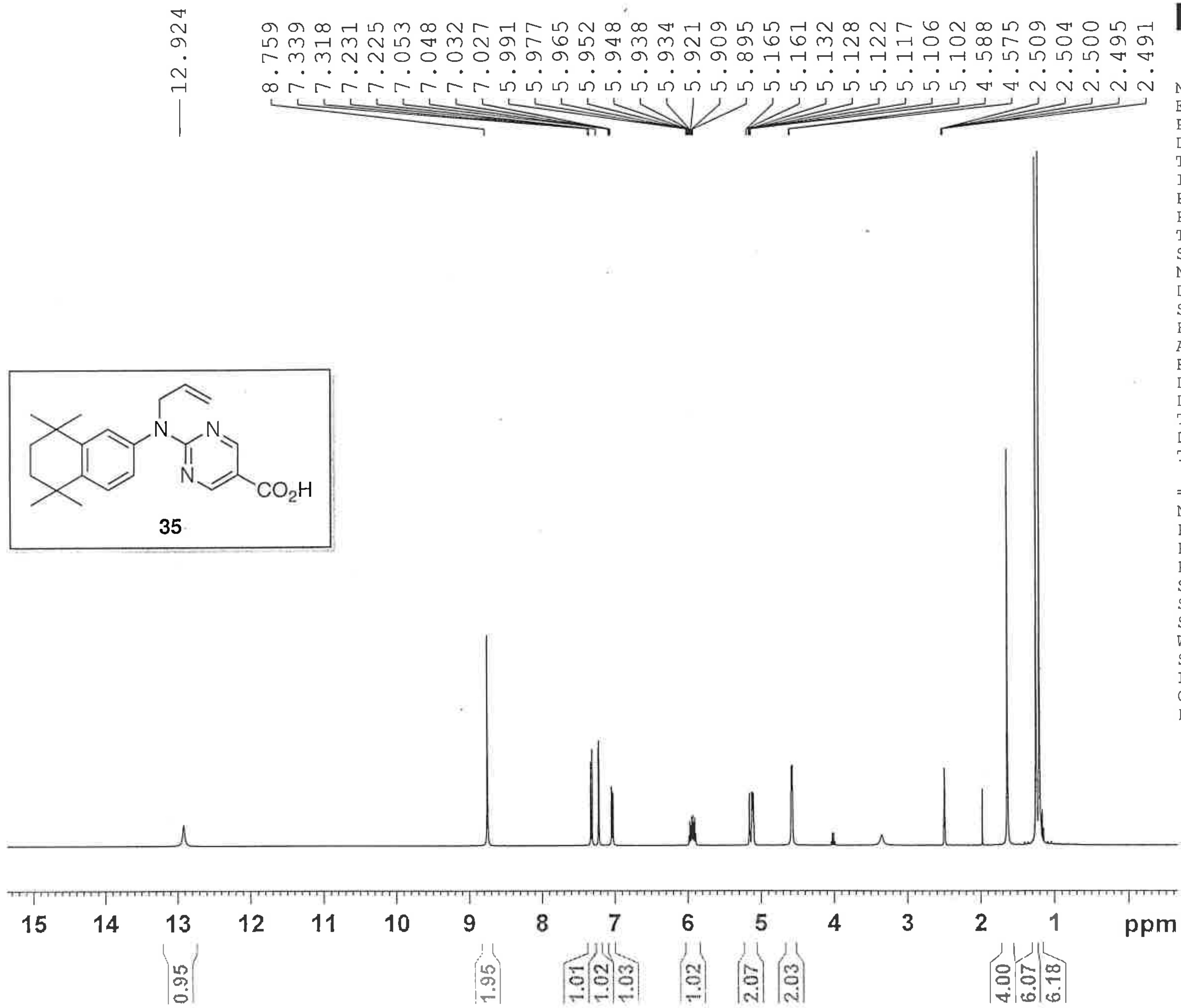

35

NAME	CWVI-161
EXPNO	1
PROCNO	1
Date_	20181010
Time_	10.36
INSTRUM	spect
PROBHD	5 mm PABBO BB-
PULPROG	zg30
TD	65536
SOLVENT	DMSO
NS	16
DS	2
SWH	8223.685 Hz
FIDRES	0.125483 Hz
AQ	3.9846387 sec
RG	4
DW	60.800 usec
DE	6.50 usec
TE	292.0 K
D1	1.00000000 sec
TD0	1

======== CHANNEL f1 ========

NUC1	1H
P1	14.75 usec
PL1	0.50 dB
PL1W	12.76071072 W
SFO1	400.1324710 MHz
SI	32768
SF	400.1300034 MHz
WDW	EM
SSB	0
LB	0.30 Hz
GB	0
PC	1.00

CWVI-161

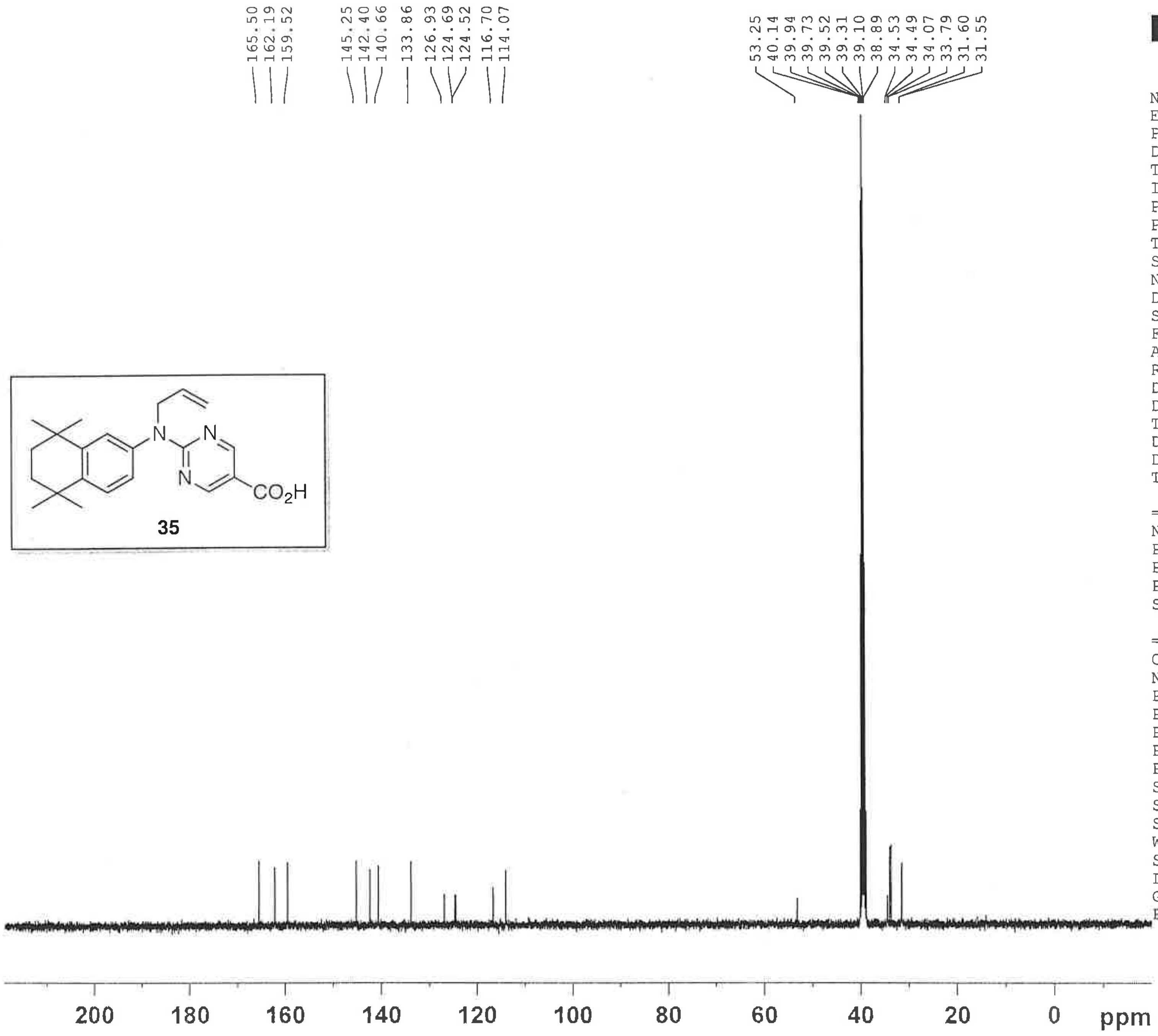

35

NAME	CWVI-161
EXPNO	2
PROCNO	1
Date_	20181010
Time_	10.44
INSTRUM	spect
PROBHD	5 mm PABBO BB-
PULPROG	zgig30
TD	65536
SOLVENT	DMSO
NS	169
DS	4
SWH	24038.461 Hz
FIDRES	0.366798 Hz
AQ	1.3631988 sec
RG	2050
DW	20.800 usec
DE	6.50 usec
TE	292.2 K
D1	2.00000000 sec
D11	0.03000000 sec
TD0	1
======== CHANNEL f1 ========	
NUC1	13C
P1	8.25 usec
PL1	-2.10 dB
PL1W	60.29227829 W
SFO1	100.6228298 MHz
======== CHANNEL f2 ========	
CPDPRG2	waltz16
NUC2	1H
PCPD2	90.00 usec
PL2	0.50 dB
PL12	16.21 dB
PL2W	12.76071072 W
PL12W	0.34266910 W
SFO2	400.1316005 MHz
SI	32768
SF	100.6128121 MHz
WDW	EM
SSB	0
LB	1.00 Hz
GB	0
PC	1.40

CWVI-171

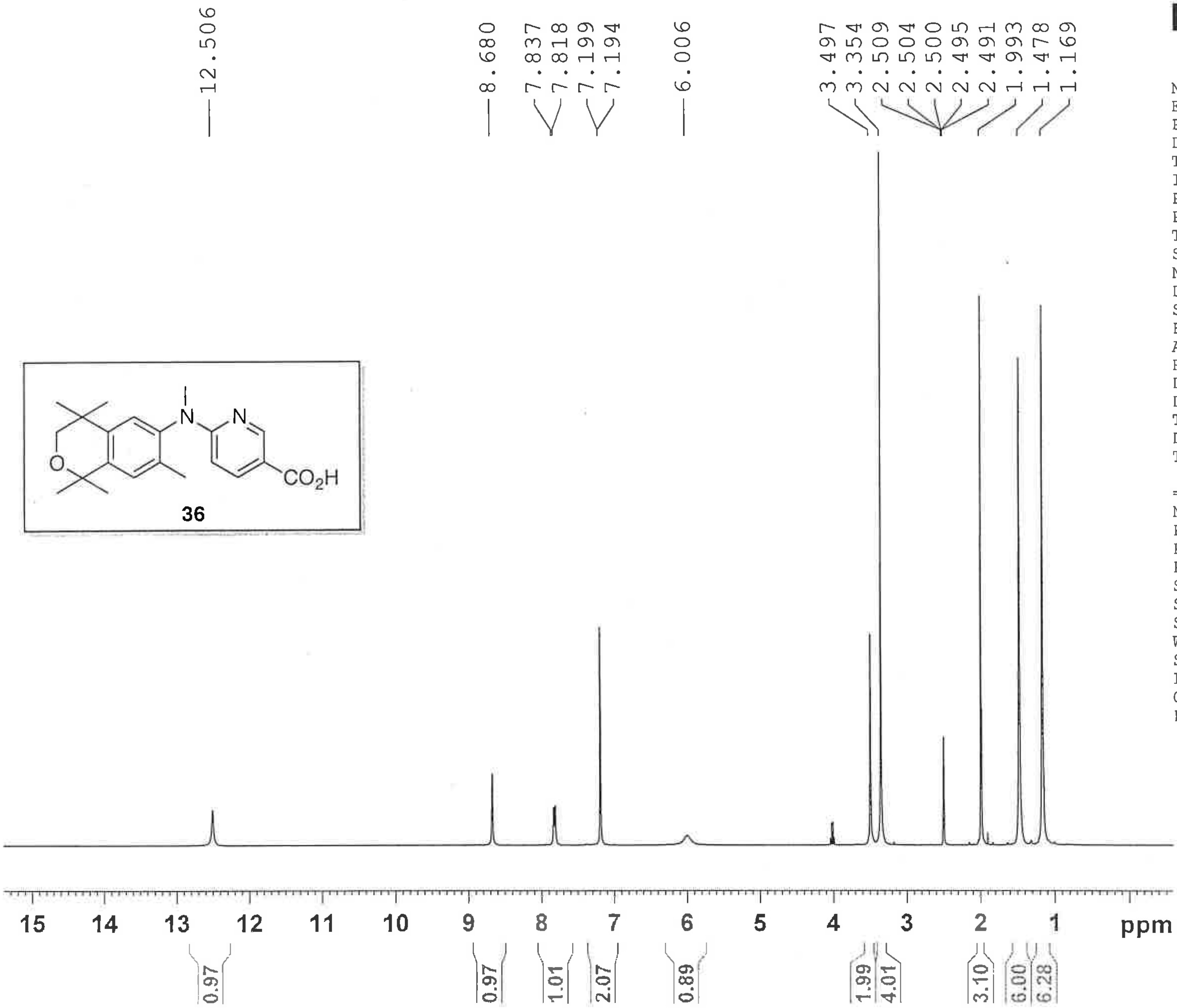

36

BRUKER

NAME	CWVI-171
EXPNO	1
PROCNO	1
Date_	20181010
Time_	11.45
INSTRUM	spect
PROBHD	5 mm PABBO BB-
PULPROG	zg30
TD	65536
SOLVENT	DMSO
NS	16
DS	2
SWH	8223.685 Hz
FIDRES	0.125483 Hz
AQ	3.9846387 sec
RG	4
DW	60.800 usec
DE	6.50 usec
TE	292.1 K
D1	1.00000000 sec
TD0	1

======== CHANNEL f1 ========

NUC1	1H
P1	14.75 usec
PL1	0.50 dB
PL1W	12.76071072 W
SFO1	400.1324710 MHz
SI	32768
SF	400.1300034 MHz
WDW	EM
SSB	0
LB	0.30 Hz
GB	0
PC	1.00

CWVI-171

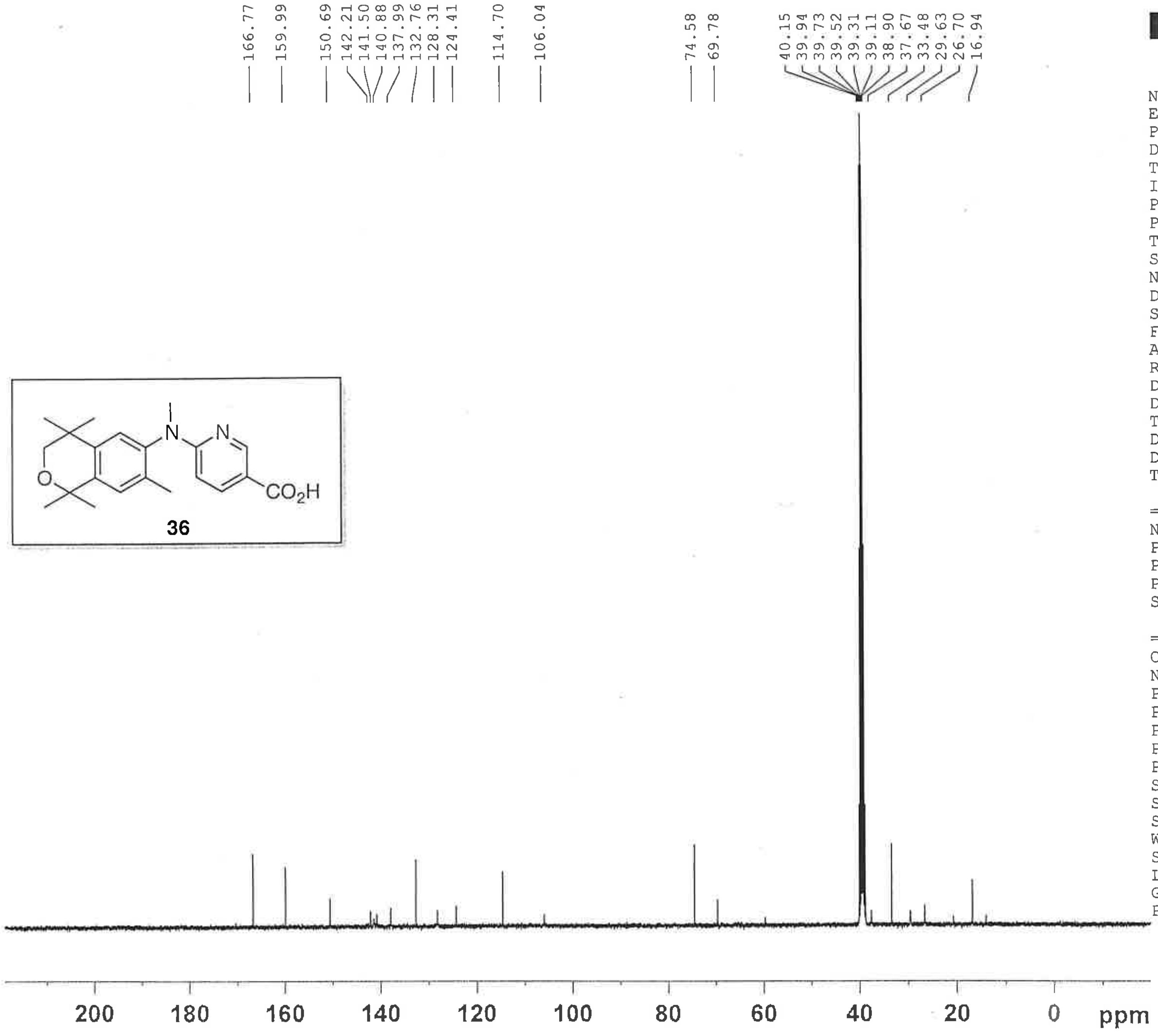

36

NAME	CWVI-171
EXPNO	2
PROCNO	1
Date_	20181010
Time	11.50
INSTRUM	spect
PROBHD	5 mm PABBO BB-
PULPROG	zgig30
TD	65536
SOLVENT	DMSO
NS	663
DS	4
SWH	24038.461 Hz
FIDRES	0.366798 Hz
AQ	1.3631988 sec
RG	2050
DW	20.800 usec
DE	6.50 usec
TE	292.2 K
D1	2.00000000 sec
D11	0.03000000 sec
TD0	1

======== CHANNEL f1 ========

NUC1	13C
P1	8.25 usec
PL1	-2.10 dB
PL1W	60.29227829 W
SFO1	100.6228298 MHz

======== CHANNEL f2 ========

CPDPRG2	waltz16
NUC2	1H
PCPD2	90.00 usec
PL2	0.50 dB
PL12	16.21 dB
PL2W	12.76071072 W
PL12W	0.34266910 W
SFO2	400.1316005 MHz
SI	32768
SF	100.6128113 MHz
WDW	EM
SSB	0
LB	1.00 Hz
GB	0
PC	1.40

CWVI-157

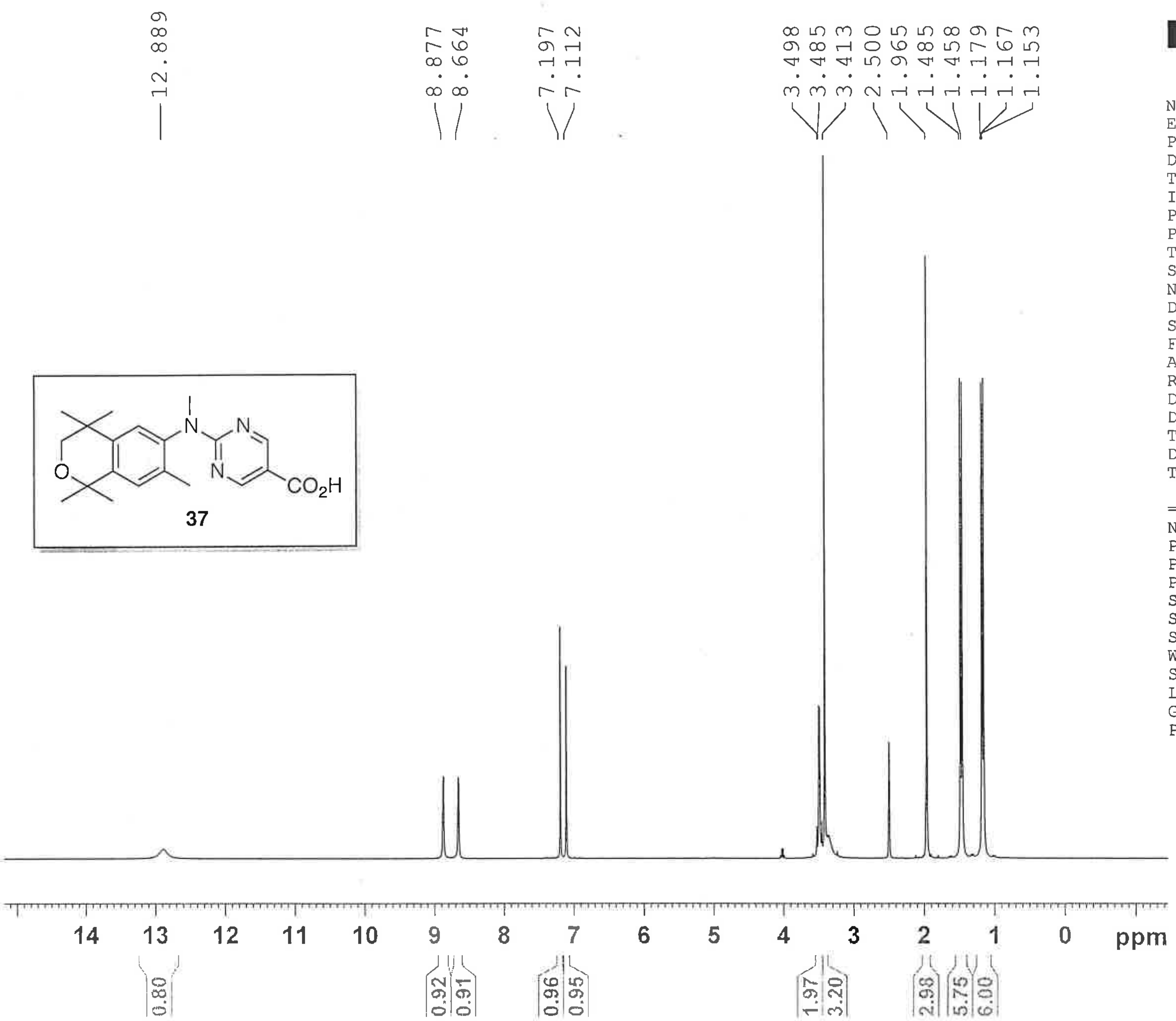

NAME	CWVI-157	
EXPNO	1	
PROCNO	1	
Date_	20181010	
Time	10.18	
INSTRUM	spect	
PROBHD	5 mm PABBO BB-	
PULPROG	zg30	
TD	65536	
SOLVENT	DMSO	
NS	16	
DS	2	
SWH	8223.685	Hz
FIDRES	0.125483	Hz
AQ	3.9846387	sec
RG	4	
DW	60.800	usec
DE	6.50	usec
TE	291.9	K
D1	1.00000000	sec
TD0	1	
======== CHANNEL f1 ========		
NUC1	1H	
P1	14.75	usec
PL1	0.50	dB
PL1W	12.76071072	W
SFO1	400.1324710	MHz
SI	32768	
SF	400.1300034	MHz
WDW	EM	
SSB	0	
LB	0.30	Hz
GB	0	
PC	1.00	

CWVI-157

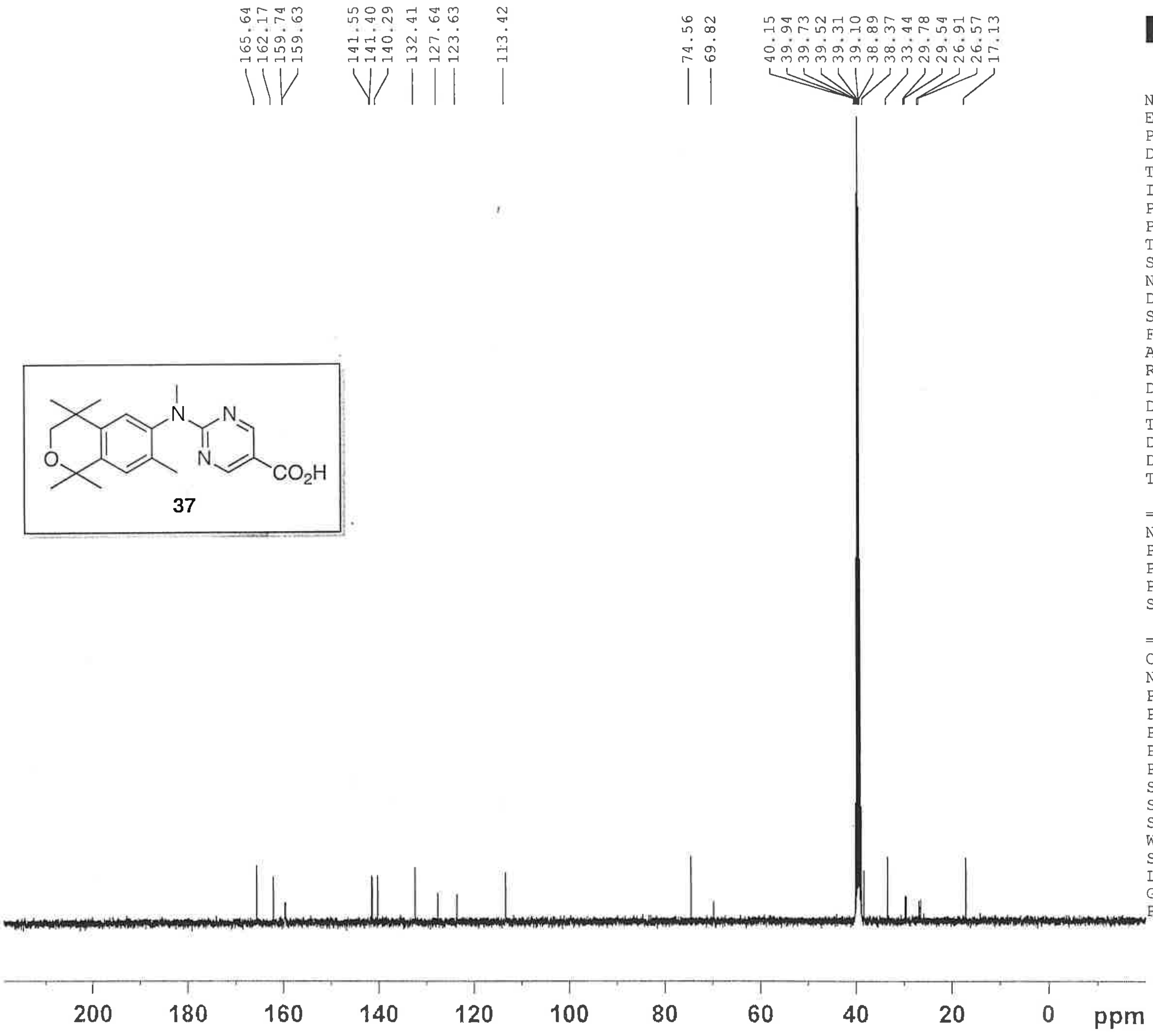

BRUKER

NAME	CWVI-157	
EXPNO	2	
PROCNO	1	
Date_	20181010	
Time	10.22	
INSTRUM	spect	
PROBHD	5 mm PABBO BB-	
PULPROG	zgig30	
TD	65536	
SOLVENT	DMSO	
NS	201	
DS	4	
SWH	24038.461	Hz
FIDRES	0.366798	Hz
AQ	1.3631988	sec
RG	2050	
DW	20.800	usec
DE	6.50	usec
TE	292.1	K
D1	2.00000000	sec
D11	0.03000000	sec
TD0	1	
======== CHANNEL f1 ========		
NUC1	13C	
P1	8.25	usec
PL1	-2.10	dB
PL1W	60.29227829	W
SFO1	100.6228298	MHz
======== CHANNEL f2 ========		
CPDPRG2	waltz16	
NUC2	1H	
PCPD2	90.00	usec
PL2	0.50	dB
PL12	16.21	dB
PL2W	12.76071072	W
PL12W	0.34266910	W
SFO2	400.1316005	MHz
SI	32768	
SF	100.6128113	MHz
WDW	EM	
SSB	0	
LB	1.00	Hz
GB	0	
PC	1.40	

CWVI-169

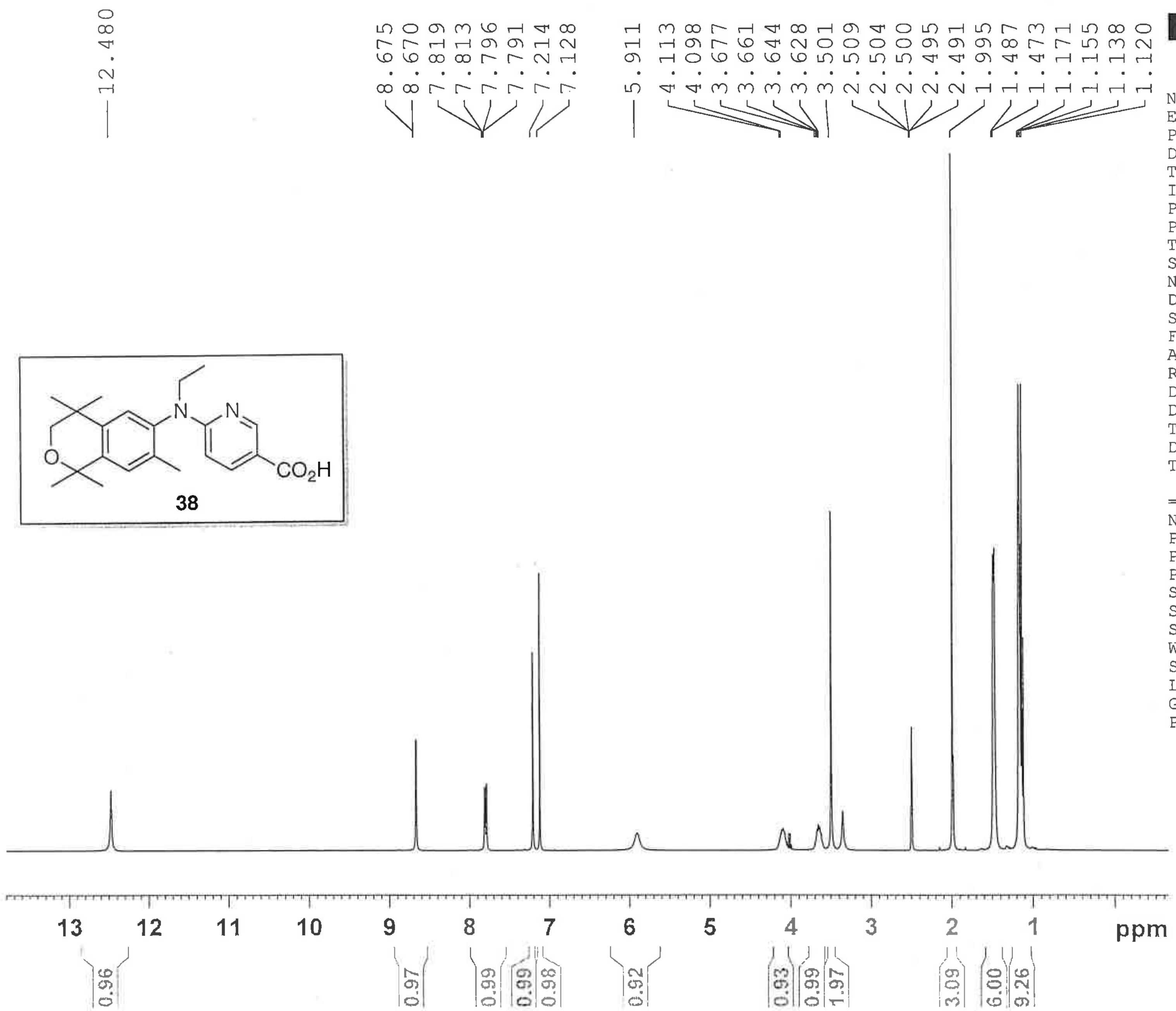

BRUKER

NAME	CWVI-169
EXPNO	1
PROCNO	1
Date_	20181010
Time_	13.13
INSTRUM	spect
PROBHD	5 mm PABBO BB-
PULPROG	zg30
TD	65536
SOLVENT	DMSO
NS	16
DS	2
SWH	8223.685 Hz
FIDRES	0.125483 Hz
AQ	3.9846387 sec
RG	4
DW	60.800 usec
DE	6.50 usec
TE	292.1 K
D1	1.00000000 sec
TD0	1

======== CHANNEL f1 ========

NUC1	1H
P1	14.75 usec
PL1	0.50 dB
PL1W	12.76071072 W
SFO1	400.1324710 MHz
SI	32768
SF	400.1300034 MHz
WDW	EM
SSB	0
LB	0.30 Hz
GB	0
PC	1.00

CWVI-169

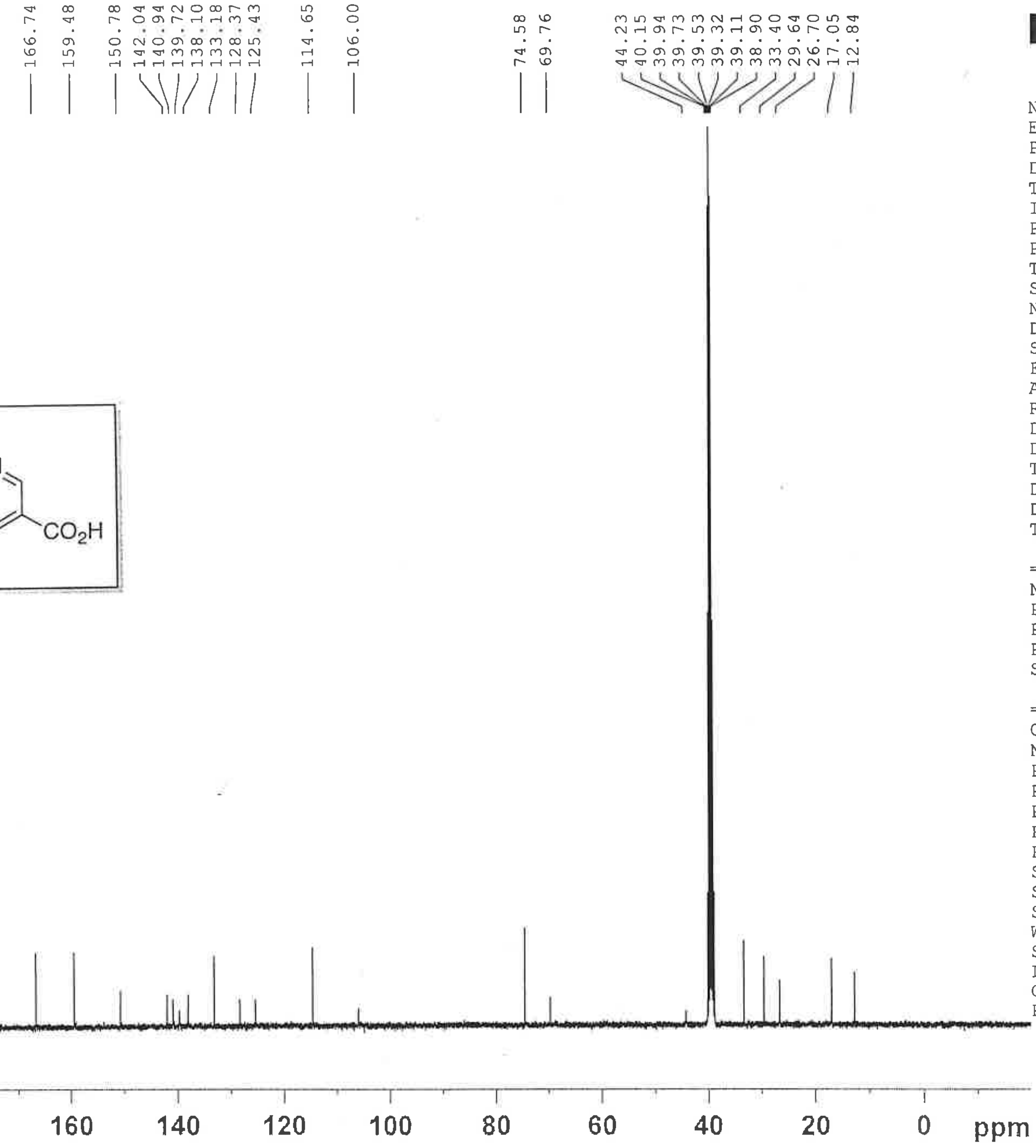

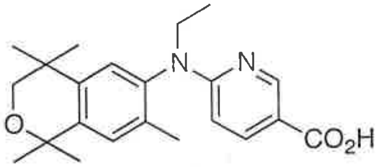

38

NAME	CWVI-169
EXPNO	2
PROCNO	1
Date_	20181010
Time	13.20
INSTRUM	spect
PROBHD	5 mm PABBO BB-
PULPROG	zgig30
TD	65536
SOLVENT	DMSO
NS	443
DS	4
SWH	24038.461 Hz
FIDRES	0.366798 Hz
AQ	1.3631988 sec
RG	2050
DW	20.800 usec
DE	6.50 usec
TE	292.4 K
D1	2.00000000 sec
D11	0.03000000 sec
TD0	1
======== CHANNEL f1 ========	
NUC1	13C
P1	8.25 usec
PL1	-2.10 dB
PL1W	60.29227829 W
SFO1	100.6228298 MHz
======== CHANNEL f2 ========	
CPDPRG2	waltz16
NUC2	1H
PCPD2	90.00 usec
PL2	0.50 dB
PL12	16.21 dB
PL2W	12.76071072 W
PL12W	0.34266910 W
SFO2	400.1316005 MHz
SI	32768
SF	100.6128113 MHz
WDW	EM
SSB	0
LB	1.00 Hz
GB	0
PC	1.40

CWVI-165

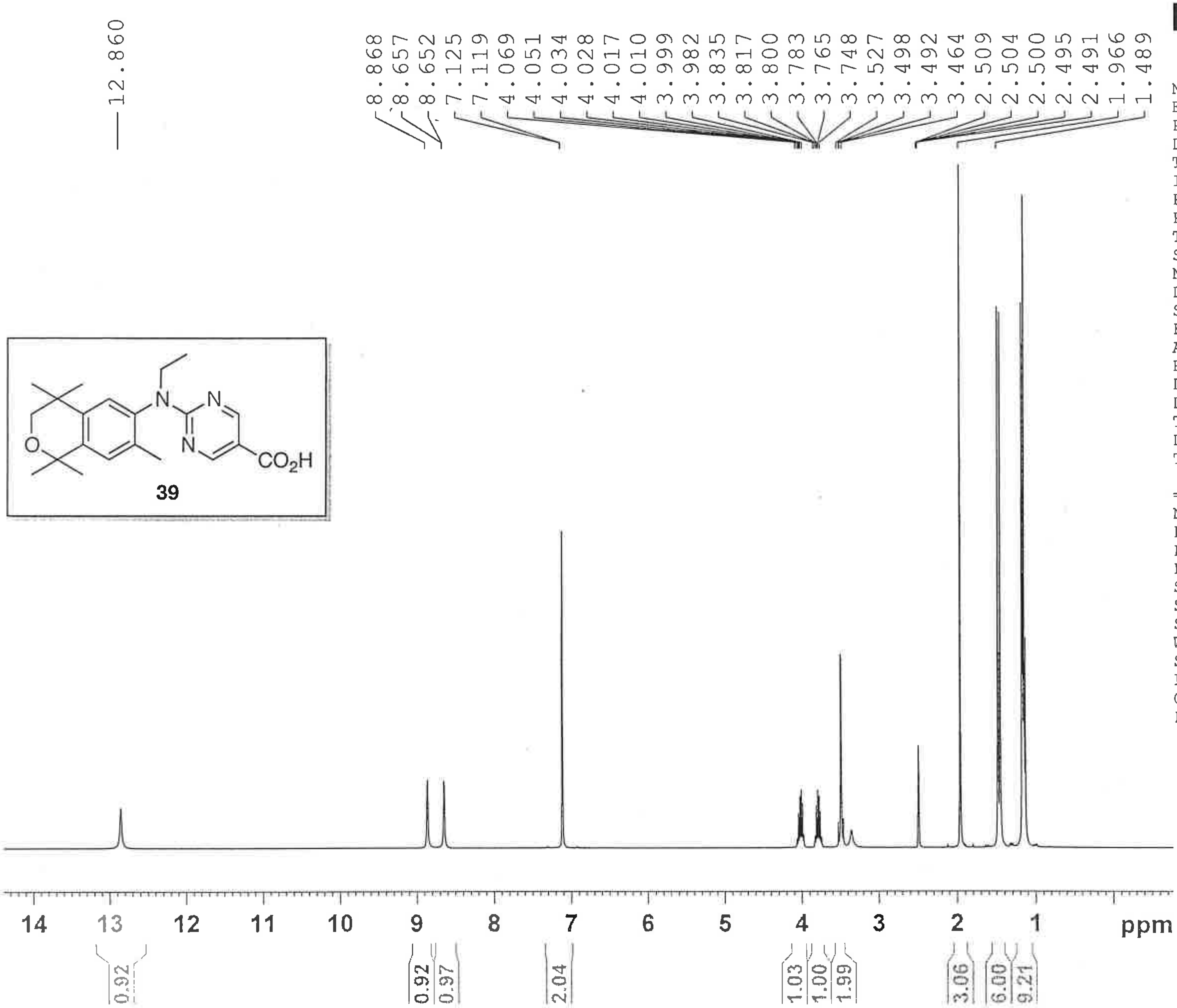

39

NAME	CWVI-165
EXPNO	1
PROCNO	1
Date_	20181010
Time	12.28
INSTRUM	spect
PROBHD	5 mm PABBO BB-
PULPROG	zg30
TD	65536
SOLVENT	DMSO
NS	16
DS	2
SWH	8223.685 Hz
FIDRES	0.125483 Hz
AQ	3.9846387 sec
RG	4
DW	60.800 usec
DE	6.50 usec
TE	292.1 K
D1	1.00000000 sec
TD0	1

======== CHANNEL f1 ========

NUC1	1H
P1	14.75 usec
PL1	0.50 dB
PL1W	12.76071072 W
SFO1	400.1324710 MHz
SI	32768
SF	400.1300034 MHz
WDW	EM
SSB	0
LB	0.30 Hz
GB	0
PC	1.00

CWVI-165

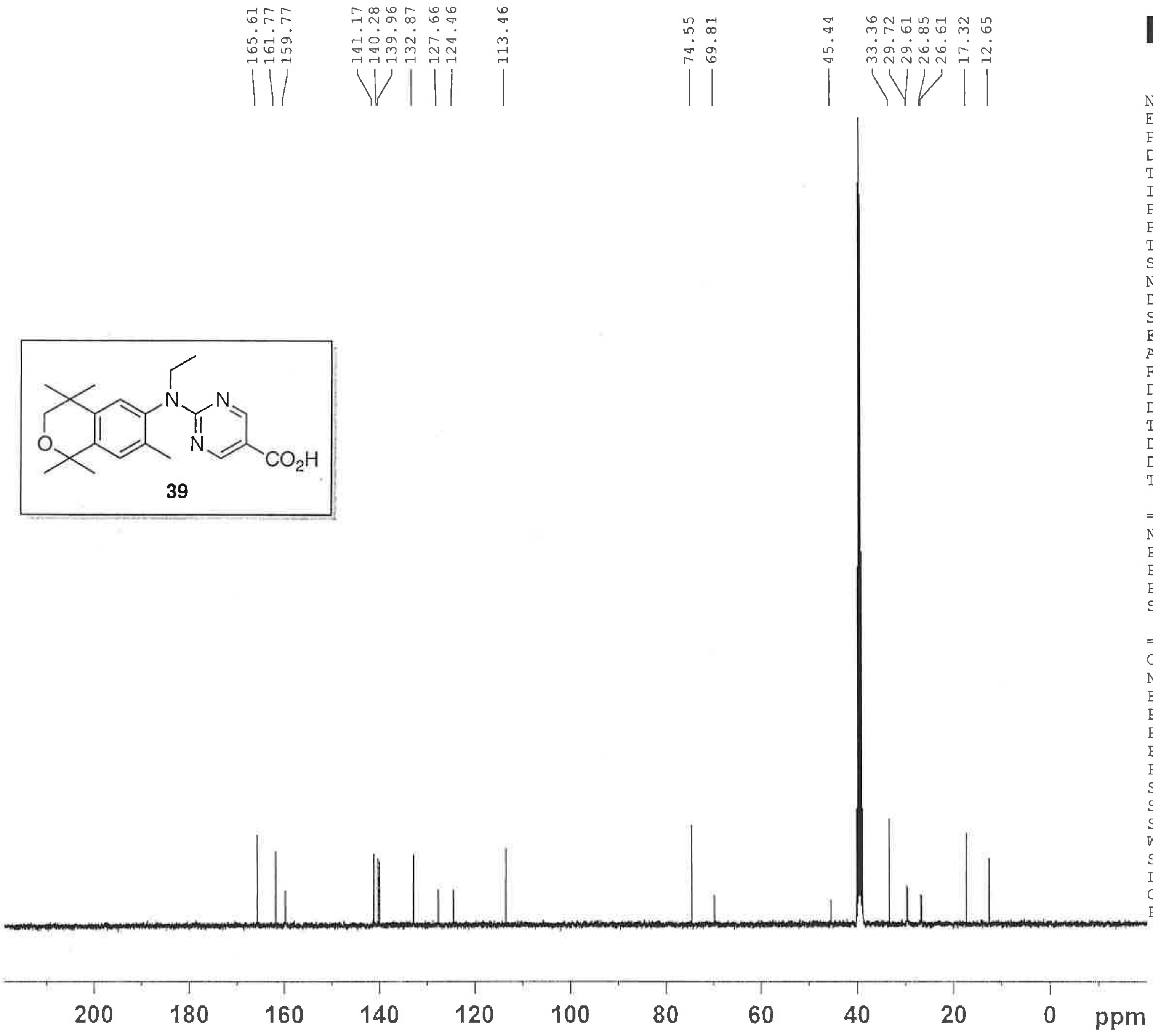

39

NAME	CWVI-165	
EXPNO	2	
PROCNO	1	
Date_	20181010	
Time	12.46	
INSTRUM	spect	
PROBHD	5 mm PABBO BB-	
PULPROG	zgig30	
TD	65536	
SOLVENT	DMSO	
NS	354	
DS	4	
SWH	24038.461	Hz
FIDRES	0.366798	Hz
AQ	1.3631988	sec
RG	2050	
DW	20.800	usec
DE	6.50	usec
TE	292.4	K
D1	2.00000000	sec
D11	0.03000000	sec
TD0	1	
======== CHANNEL f1 ========		
NUC1	13C	
P1	8.25	usec
PL1	-2.10	dB
PL1W	60.29227829	W
SFO1	100.6228298	MHz
======== CHANNEL f2 ========		
CPDPRG2	waltz16	
NUC2	1H	
PCPD2	90.00	usec
PL2	0.50	dB
PL12	16.21	dB
PL2W	12.76071072	W
PL12W	0.34266910	W
SFO2	400.1316005	MHz
SI	32768	
SF	100.6128113	MHz
WDW	EM	
SSB	0	
LB	1.00	Hz
GB	0	
PC	1.40	

CWVI-175

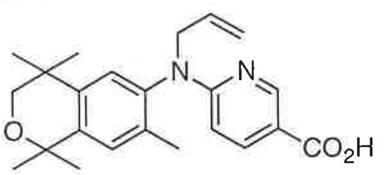

40

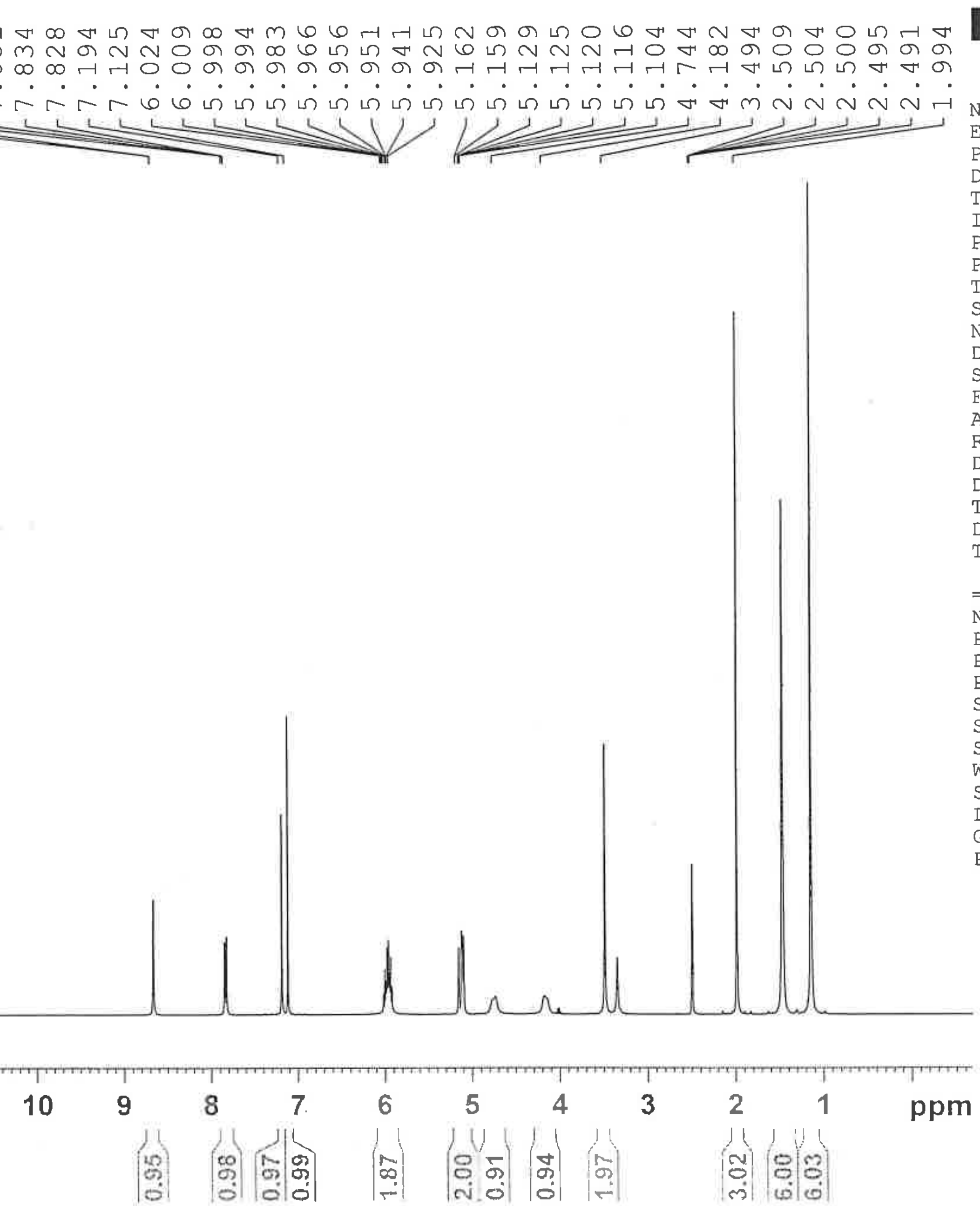

NAME	CWVI-175
EXPNO	1
PROCNO	1
Date_	20181010
Time_	14.15
INSTRUM	spect
PROBHD	5 mm PABBO BB-
PULPROG	zg30
TD	65536
SOLVENT	DMSO
NS	16
DS	2
SWH	8223.685 Hz
FIDRES	0.125483 Hz
AQ	3.9846387 sec
RG	4
DW	60.800 usec
DE	6.50 usec
TE	292.2 K
D1	1.00000000 sec
TD0	1

======== CHANNEL f1 ========

NUC1	1H
P1	14.75 usec
PL1	0.50 dB
PL1W	12.76071072 W
SFO1	400.1324710 MHz
SI	32768
SF	400.1300034 MHz
WDW	EM
SSB	0
LB	0.30 Hz
GB	0
PC	1.00

CWVI-175

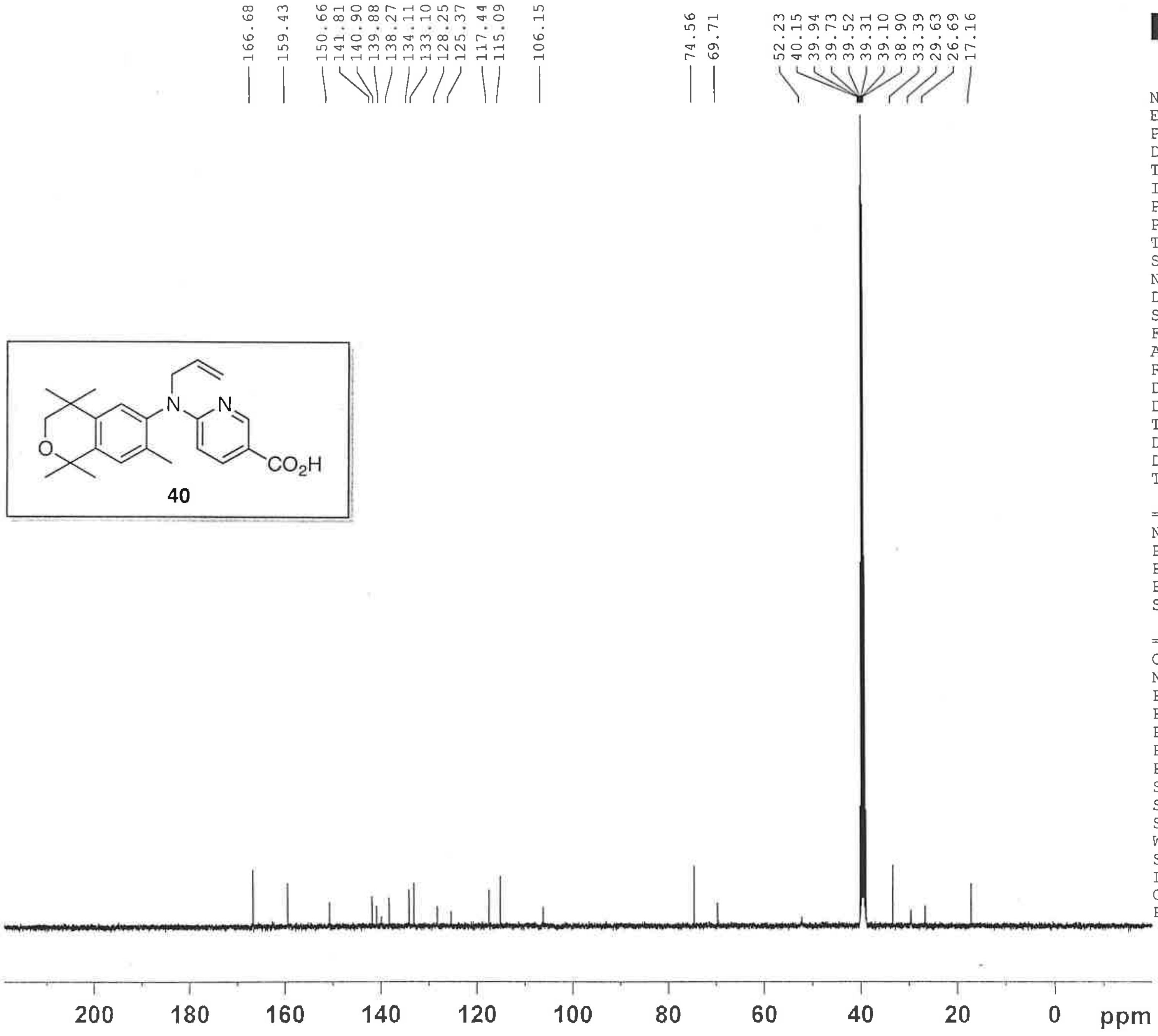

40

NAME	CWVI-175	
EXPNO	2	
PROCNO	1	
Date_	20181010	
Time	14.22	
INSTRUM	spect	
PROBHD	5 mm PABBO BB-	
PULPROG	zgig30	
TD	65536	
SOLVENT	DMSO	
NS	379	
DS	4	
SWH	24038.461	Hz
FIDRES	0.366798	Hz
AQ	1.3631988	sec
RG	2050	
DW	20.800	usec
DE	6.50	usec
TE	292.4	K
D1	2.00000000	sec
D11	0.03000000	sec
TD0	1	
======== CHANNEL f1 ========		
NUC1	13C	
P1	8.25	usec
PL1	-2.10	dB
PL1W	60.29227829	W
SFO1	100.6228298	MHz
======== CHANNEL f2 ========		
CPDPRG2	waltz16	
NUC2	1H	
PCPD2	90.00	usec
PL2	0.50	dB
PL12	16.21	dB
PL2W	12.76071072	W
PL12W	0.34266910	W
SFO2	400.1316005	MHz
SI	32768	
SF	100.6128121	MHz
WDW	EM	
SSB	0	
LB	1.00	Hz
GB	0	
PC	1.40	

CWVI-167

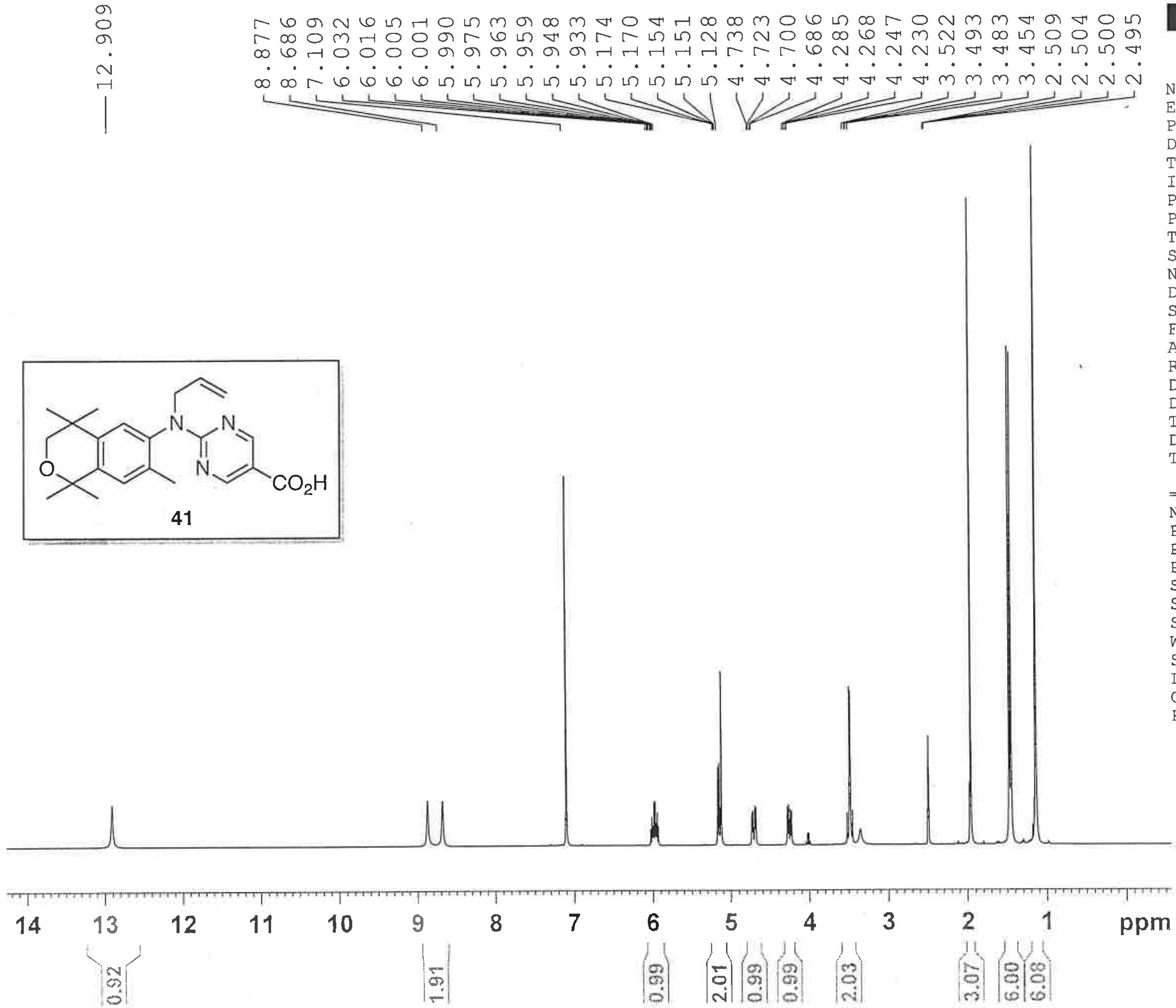

NAME	CWVI-167
EXPNO	1
PROCNO	1
Date_	20181010
Time	12.58
INSTRUM	spect
PROBHD	5 mm PABBO BB-
PULPROG	zg30
TD	65536
SOLVENT	DMSO
NS	16
DS	2
SWH	8223.685 Hz
FIDRES	0.125483 Hz
AQ	3.9846387 sec
RG	4
DW	60.800 usec
DE	6.50 usec
TE	292.2 K
D1	1.00000000 sec
TD0	1

======== CHANNEL f1 ========

NUC1	1H
P1	14.75 usec
PL1	0.50 dB
PL1W	12.76071072 W
SFO1	400.1324710 MHz
SI	32768
SF	400.1300034 MHz
WDW	EM
SSB	0
LB	0.30 Hz
GB	0
PC	1.00

CWVI-167

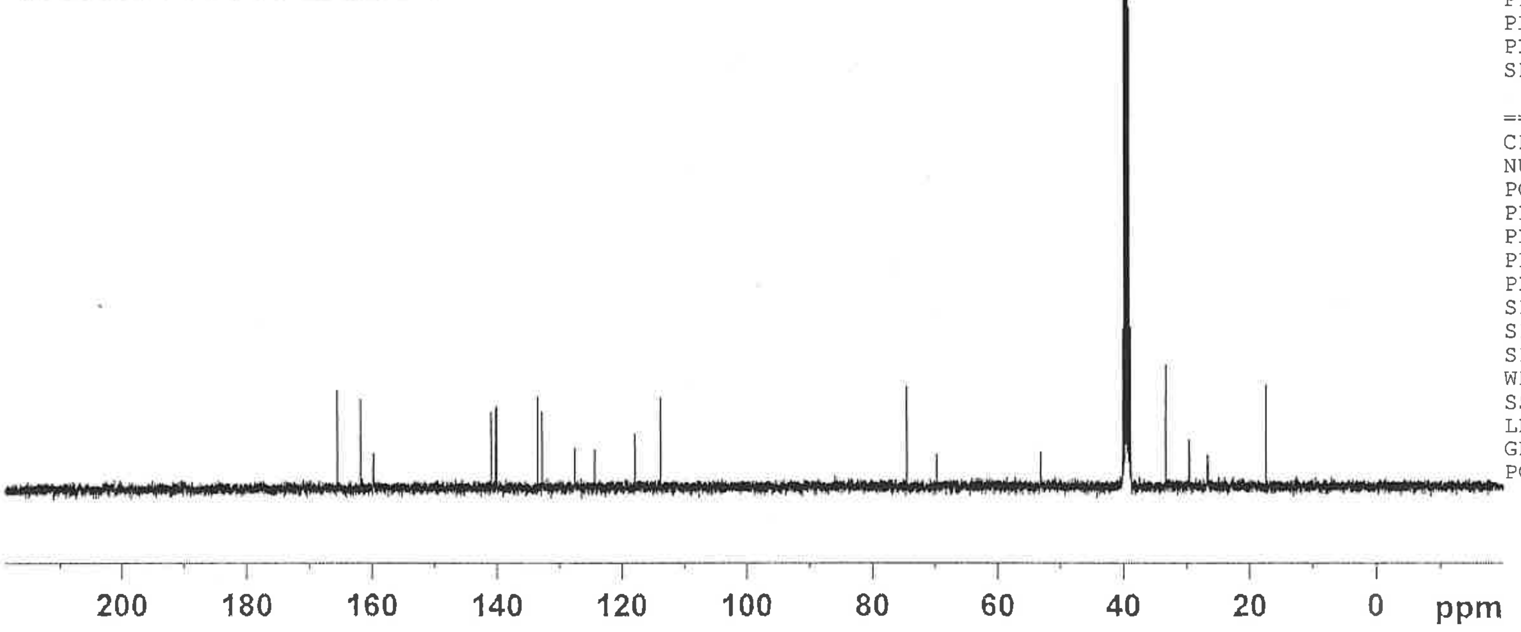

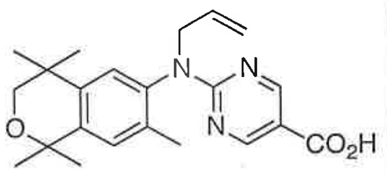

41

NAME	CWVI-167
EXPNO	2
PROCNO	1
Date_	20181010
Time	13.04
INSTRUM	spect
PROBHD	5 mm PABBO BB-
PULPROG	zgig30
TD	65536
SOLVENT	DMSO
NS	161
DS	4
SWH	24038.461 Hz
FIDRES	0.366798 Hz
AQ	1.3631988 sec
RG	2050
DW	20.800 usec
DE	6.50 usec
TE	292.4 K
D1	2.00000000 sec
D11	0.03000000 sec
TD0	1

======== CHANNEL f1 ========

NUC1	13C
P1	8.25 usec
PL1	-2.10 dB
PL1W	60.29227829 W
SFO1	100.6228298 MHz

======== CHANNEL f2 ========

CPDPRG2	waltz16
NUC2	1H
PCPD2	90.00 usec
PL2	0.50 dB
PL12	16.21 dB
PL2W	12.76071072 W
PL12W	0.34266910 W
SFO2	400.1316005 MHz
SI	32768
SF	100.6128113 MHz
WDW	EM
SSB	0
LB	1.00 Hz
GB	0
PC	1.40

CWVI-201

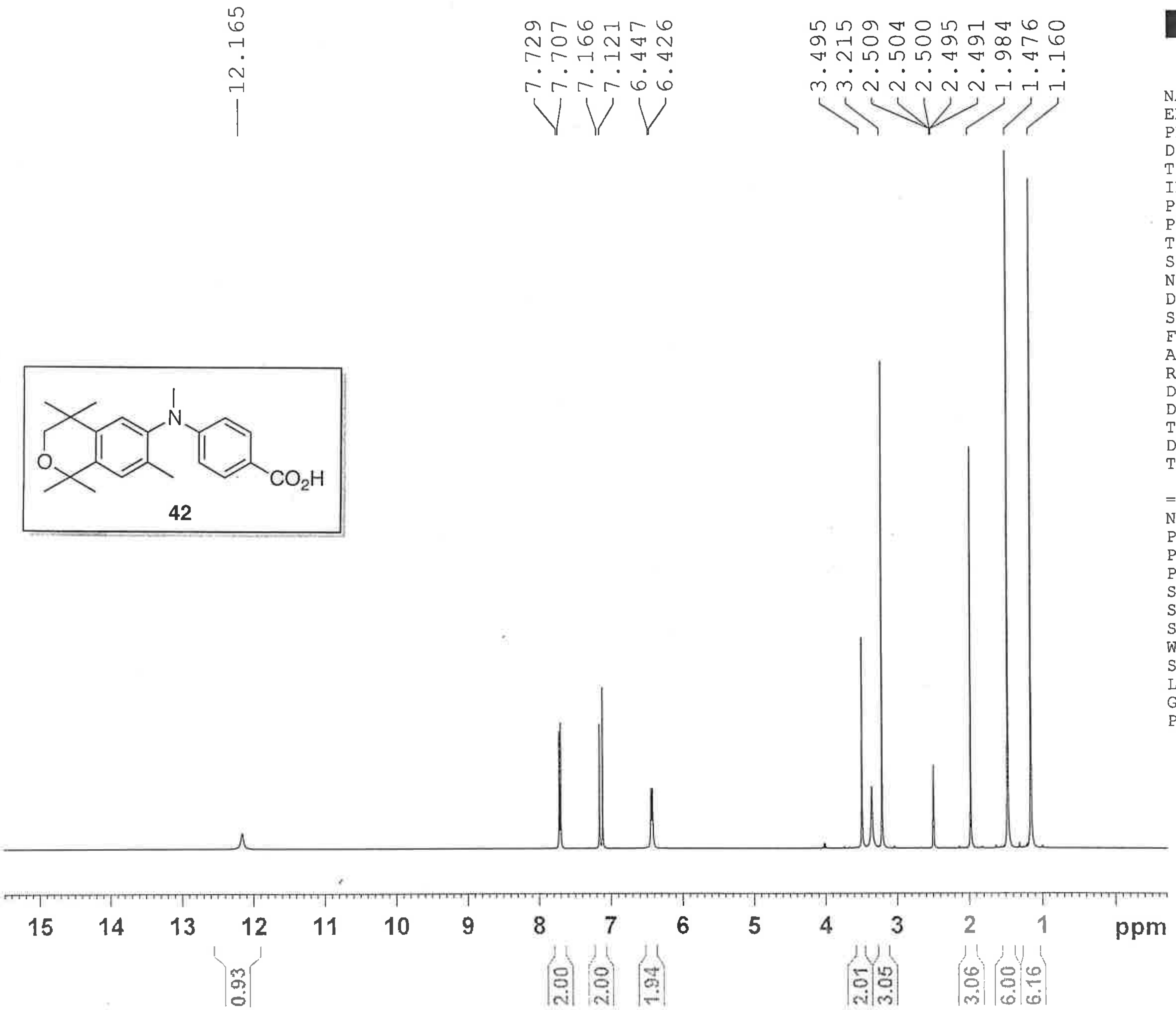

NAME	CWVI-201
EXPNO	1
PROCNO	1
Date_	20181010
Time_	22.23
INSTRUM	spect
PROBHD	5 mm PABBO BB-
PULPROG	zg30
TD	65536
SOLVENT	DMSO
NS	16
DS	2
SWH	8223.685 Hz
FIDRES	0.125483 Hz
AQ	3.9846387 sec
RG	4
DW	60.800 usec
DE	6.50 usec
TE	292.2 K
D1	1.00000000 sec
TD0	1

======== CHANNEL f1 ========

NUC1	1H
P1	14.75 usec
PL1	0.50 dB
PL1W	12.76071072 W
SFO1	400.1324710 MHz
SI	32768
SF	400.1300034 MHz
WDW	EM
SSB	0
LB	0.30 Hz
GB	0
PC	1.00

CWVI-201

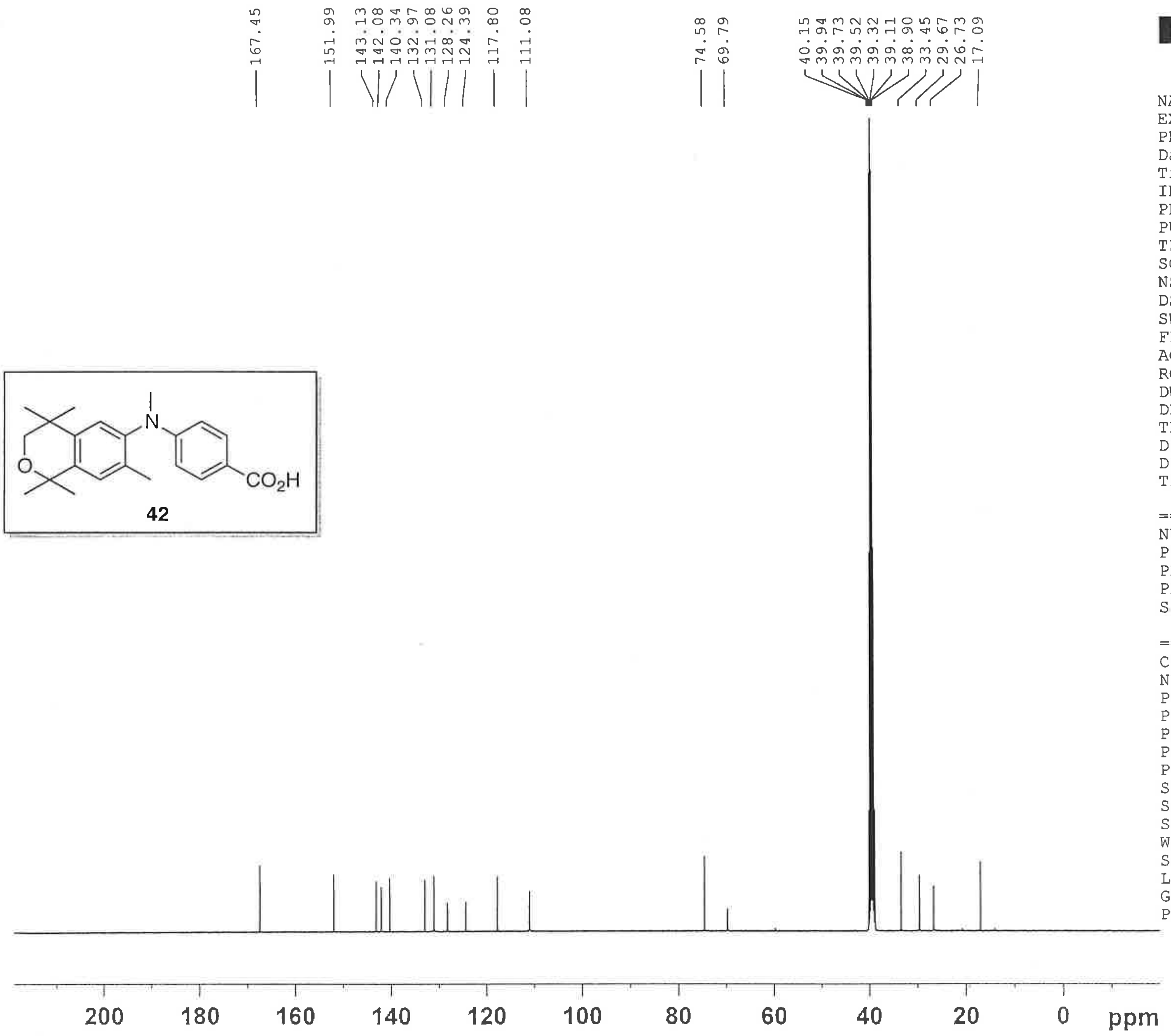

BRUKER

NAME	CWVI-201
EXPNO	2
PROCNO	1
Date_	20181010
Time_	22.29
INSTRUM	spect
PROBHD	5 mm PABBO BB-
PULPROG	zgig30
TD	65536
SOLVENT	DMSO
NS	14728
DS	4
SWH	24038.461 Hz
FIDRES	0.366798 Hz
AQ	1.3631988 sec
RG	2050
DW	20.800 usec
DE	6.50 usec
TE	292.4 K
D1	2.00000000 sec
D11	0.03000000 sec
TD0	1
======== CHANNEL f1 ========	
NUC1	13C
P1	8.25 usec
PL1	-2.10 dB
PL1W	60.29227829 W
SFO1	100.6228298 MHz
======== CHANNEL f2 ========	
CPDPRG2	waltz16
NUC2	1H
PCPD2	90.00 usec
PL2	0.50 dB
PL12	16.21 dB
PL2W	12.76071072 W
PL12W	0.34266910 W
SFO2	400.1316005 MHz
SI	32768
SF	100.6128120 MHz
WDW	EM
SSB	0
LB	1.00 Hz
GB	0
PC	1.40

CWVI-199

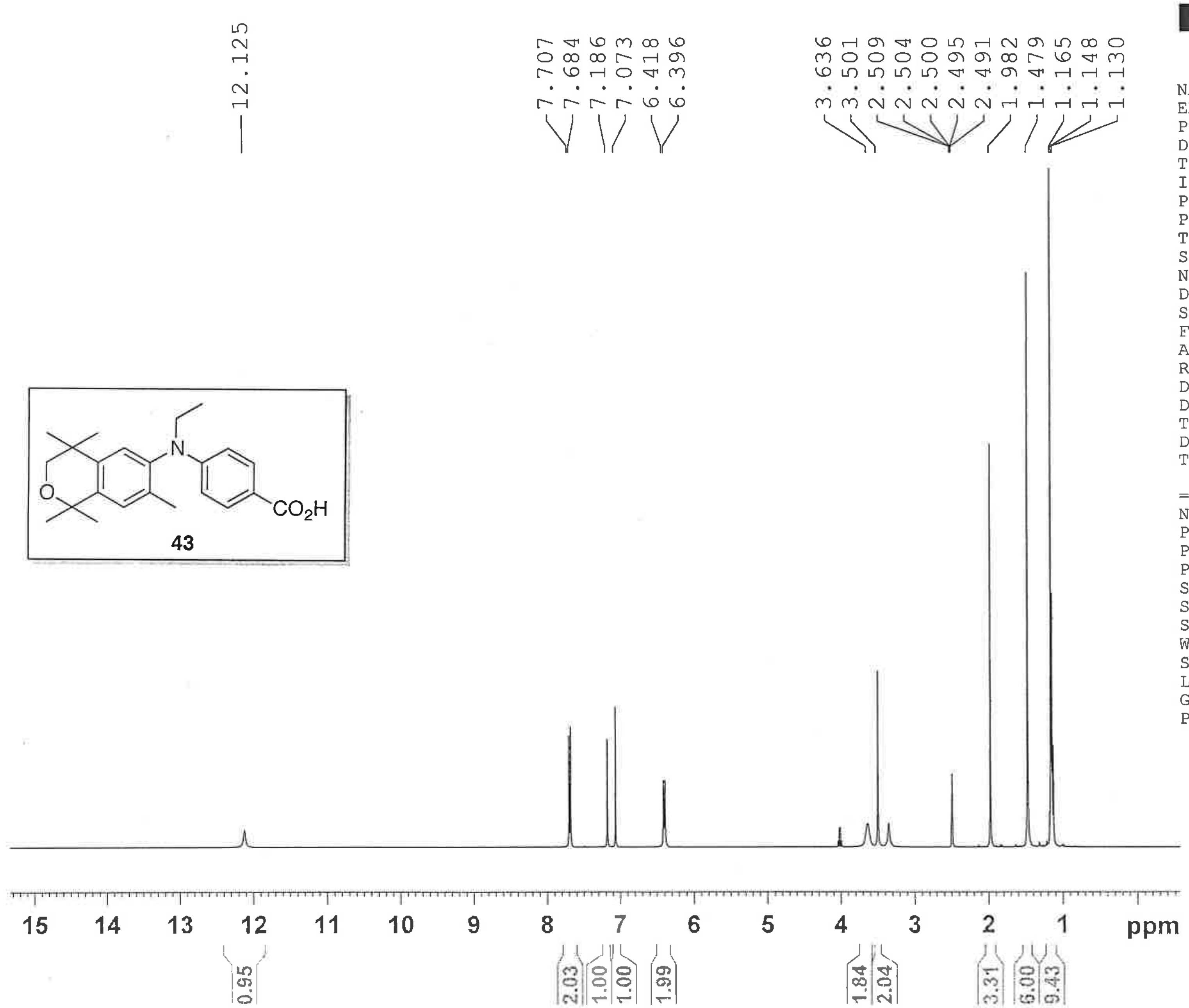

BRUKER

NAME	CWVI-199
EXPNO	1
PROCNO	1
Date_	20181010
Time	22.03
INSTRUM	spect
PROBHD	5 mm PABBO BB-
PULPROG	zg30
TD	65536
SOLVENT	DMSO
NS	16
DS	2
SWH	8223.685 Hz
FIDRES	0.125483 Hz
AQ	3.9846387 sec
RG	4
DW	60.800 usec
DE	6.50 usec
TE	292.2 K
D1	1.00000000 sec
TD0	1

======== CHANNEL f1 ========

NUC1	1H
P1	14.75 usec
PL1	0.50 dB
PL1W	12.76071072 W
SFO1	400.1324710 MHz
SI	32768
SF	400.1300034 MHz
WDW	EM
SSB	0
LB	0.30 Hz
GB	0
PC	1.00

CWVI-199

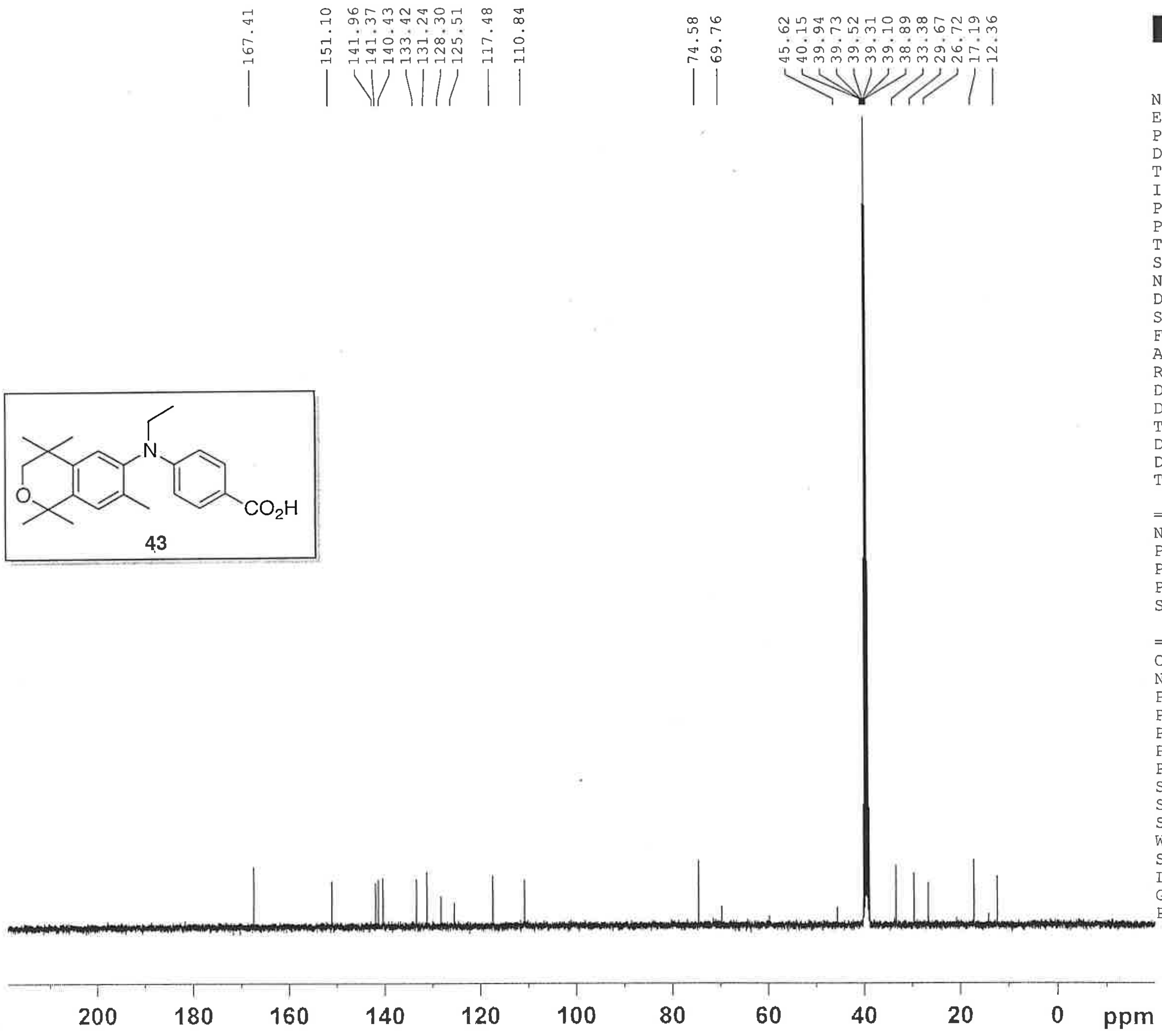

BRUKER

NAME	CWVI-199
EXPNO	2
PROCNO	1
Date_	20181010
Time	22.10
INSTRUM	spect
PROBHD	5 mm PABBO BB-
PULPROG	zgig30
TD	65536
SOLVENT	DMSO
NS	225
DS	4
SWH	24038.461 Hz
FIDRES	0.366798 Hz
AQ	1.3631988 sec
RG	2050
DW	20.800 usec
DE	6.50 usec
TE	292.4 K
D1	2.00000000 sec
D11	0.03000000 sec
TD0	1
======== CHANNEL f1 ========	
NUC1	13C
P1	8.25 usec
PL1	-2.10 dB
PL1W	60.29227829 W
SFO1	100.6228298 MHz
======== CHANNEL f2 ========	
CPDPRG2	waltz16
NUC2	1H
PCPD2	90.00 usec
PL2	0.50 dB
PL12	16.21 dB
PL2W	12.76071072 W
PL12W	0.34266910 W
SFO2	400.1316005 MHz
SI	32768
SF	100.6128121 MHz
WDW	EM
SSB	0
LB	1.00 Hz
GB	0
PC	1.40

CWVI-203

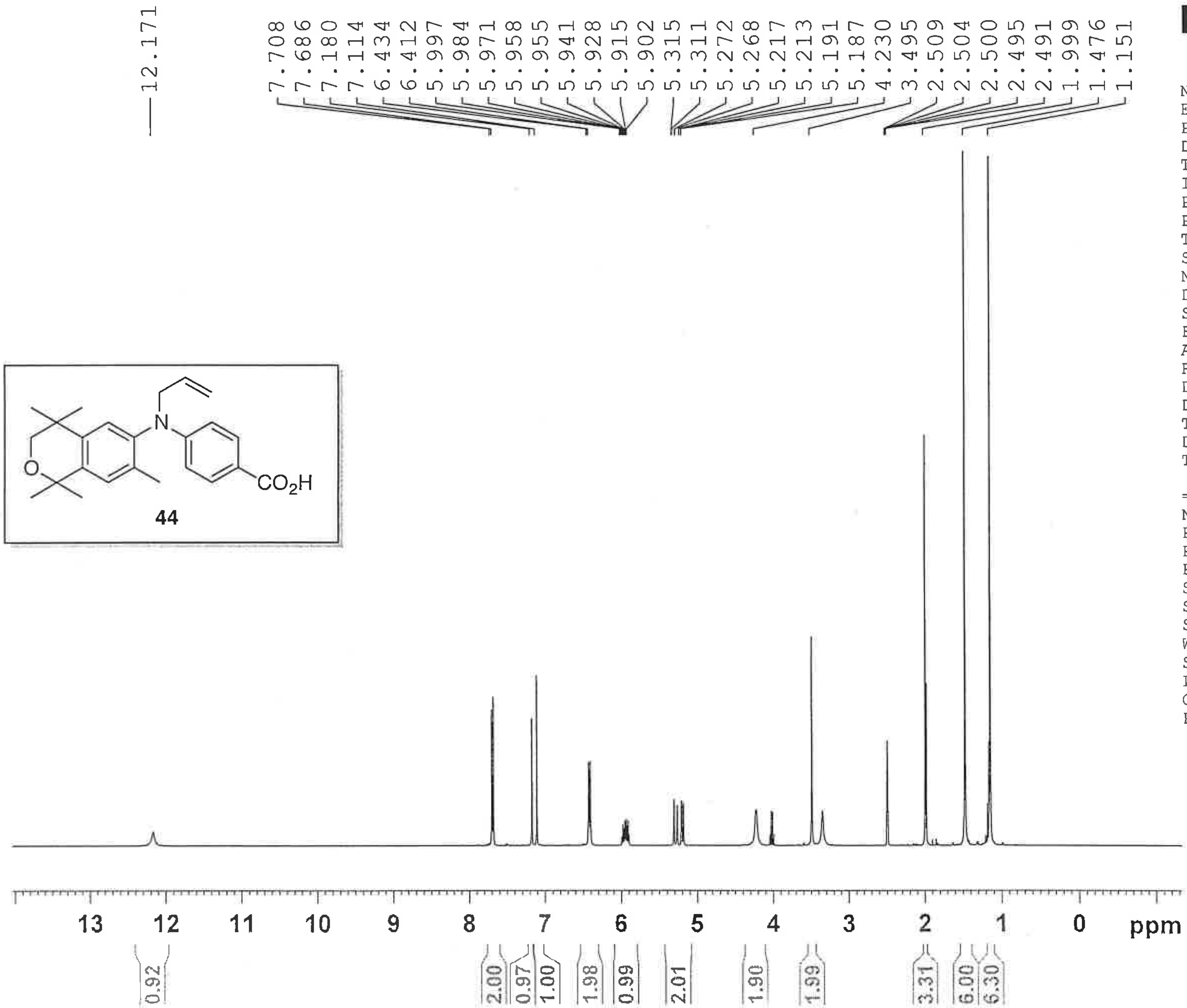

NAME	CWVI-203
EXPNO	1
PROCNO	1
Date_	20181011
Time_	12.23
INSTRUM	spect
PROBHD	5 mm PABBO BB-
PULPROG	zg30
TD	65536
SOLVENT	DMSO
NS	16
DS	2
SWH	8223.685 Hz
FIDRES	0.125483 Hz
AQ	3.9846387 sec
RG	4
DW	60.800 usec
DE	6.50 usec
TE	291.9 K
D1	1.00000000 sec
TD0	1
======== CHANNEL f1 ========	
NUC1	1H
P1	14.75 usec
PL1	0.50 dB
PL1W	12.76071072 W
SFO1	400.1324710 MHz
SI	32768
SF	400.1300034 MHz
WDW	EM
SSB	0
LB	0.30 Hz
GB	0
PC	1.00

CWVI-203

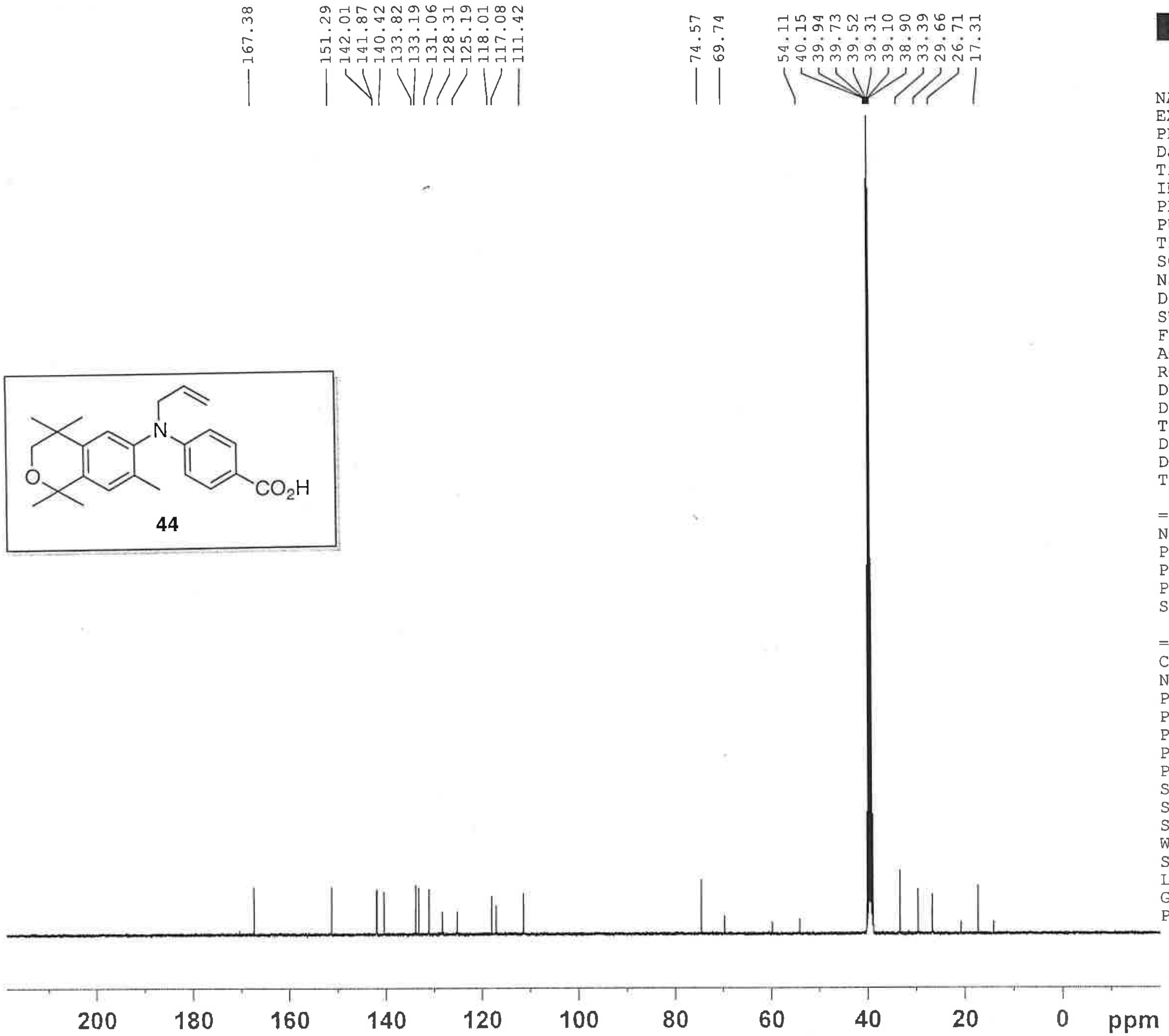

BRUKER

NAME	CWVI-203
EXPNO	2
PROCNO	1
Date_	20181011
Time	13.50
INSTRUM	spect
PROBHD	5 mm PABBO BB-
PULPROG	zgig30
TD	65536
SOLVENT	DMSO
NS	1515
DS	4
SWH	24038.461 Hz
FIDRES	0.366798 Hz
AQ	1.3631988 sec
RG	2050
DW	20.800 usec
DE	6.50 usec
TE	292.2 K
D1	2.00000000 sec
D11	0.03000000 sec
TD0	1

======== CHANNEL f1 ========

NUC1	13C
P1	8.25 usec
PL1	-2.10 dB
PL1W	60.29227829 W
SFO1	100.6228298 MHz

======== CHANNEL f2 ========

CPDPRG2	waltz16
NUC2	1H
PCPD2	90.00 usec
PL2	0.50 dB
PL12	16.21 dB
PL2W	12.76071072 W
PL12W	0.34266910 W
SFO2	400.1316005 MHz
SI	32768
SF	100.6128121 MHz
WDW	EM
SSB	0
LB	1.00 Hz
GB	0
PC	1.40

CWVI-077 Product (6-bromo-1,1,4,4,7-pentamethyl-isochroman)

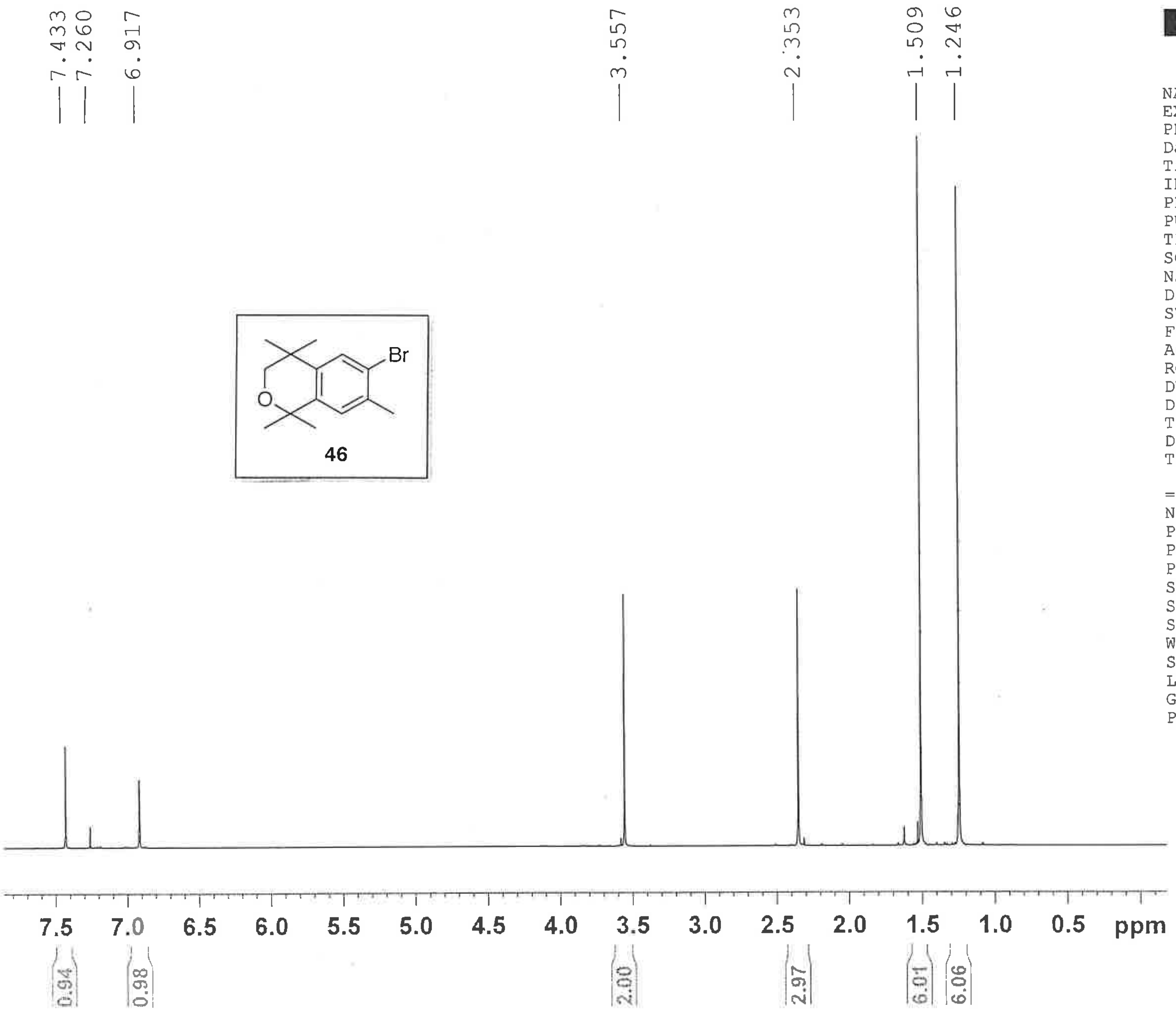

46

BRUKER

NAME	CWVI-077
EXPNO	5
PROCNO	1
Date_	20170721
Time	16.47
INSTRUM	spect
PROBHD	5 mm PABBO BB-
PULPROG	zg30
TD	65536
SOLVENT	CDCl3
NS	16
DS	2
SWH	8223.685 Hz
FIDRES	0.125483 Hz
AQ	3.9846387 sec
RG	4
DW	60.800 usec
DE	6.50 usec
TE	298.2 K
D1	1.00000000 sec
TD0	1

======== CHANNEL f1 ========

NUC1	1H
P1	14.75 usec
PL1	0.50 dB
PL1W	12.76071072 W
SFO1	400.1324710 MHz
SI	32768
SF	400.1300101 MHz
WDW	EM
SSB	0
LB	0.30 Hz
GB	0
PC	1.00

CWVI-077 Product (6-bromo-1,1,4,4,7-pentamethyl-isochroman)

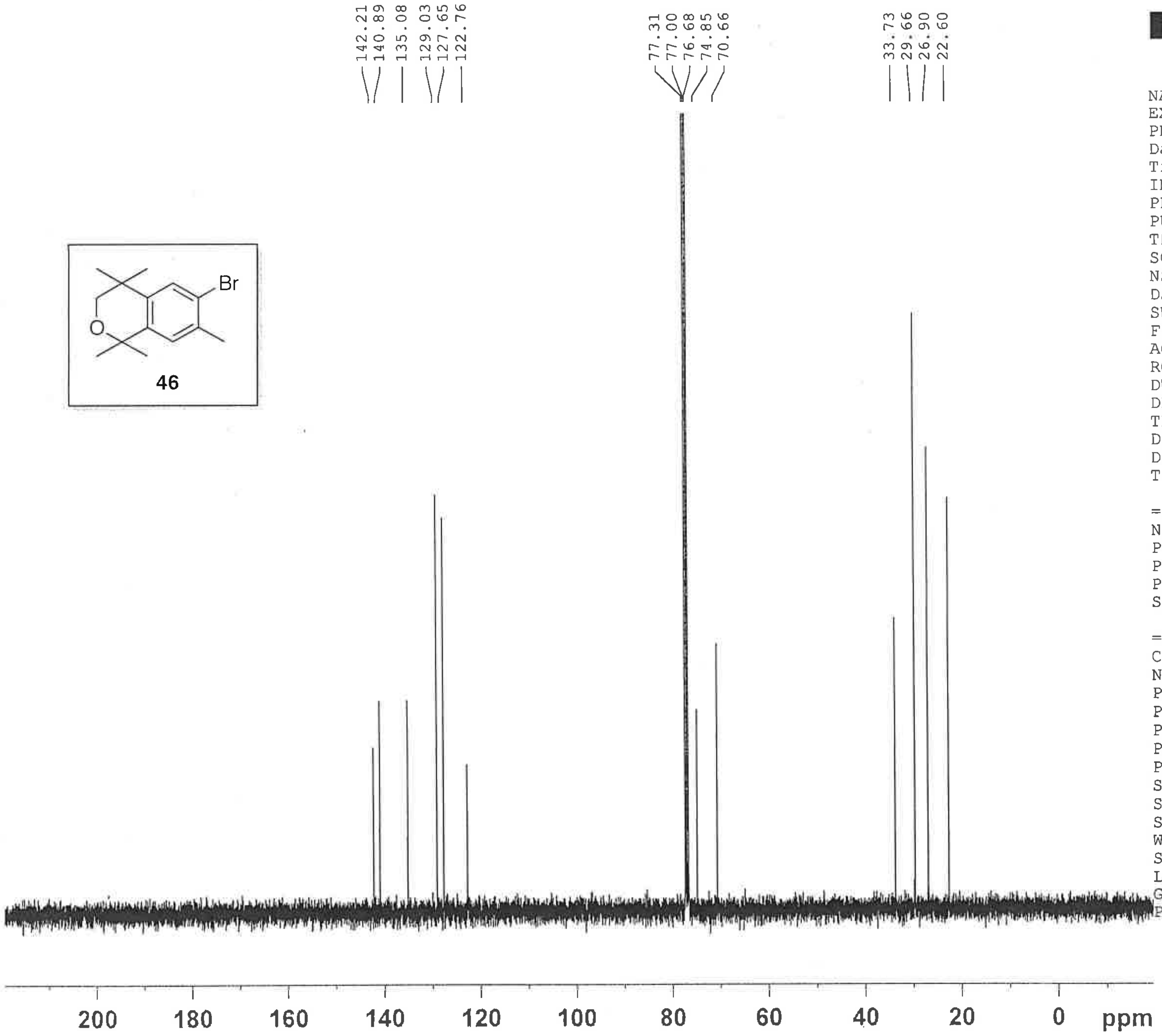

BRUKER

NAME	CWVI-077
EXPNO	6
PROCNO	1
Date_	20170721
Time_	16.53
INSTRUM	spect
PROBHD	5 mm PABBO BB-
PULPROG	zgig30
TD	65536
SOLVENT	CDCl3
NS	91
DS	4
SWH	24038.461 Hz
FIDRES	0.366798 Hz
AQ	1.3631988 sec
RG	2050
DW	20.800 usec
DE	6.50 usec
TE	298.2 K
D1	2.00000000 sec
D11	0.03000000 sec
TD0	1

======== CHANNEL f1 ========

NUC1	13C
P1	8.50 usec
PL1	-2.10 dB
PL1W	60.29227829 W
SFO1	100.6228298 MHz

======== CHANNEL f2 ========

CPDPRG2	waltz16
NUC2	1H
PCPD2	90.00 usec
PL2	0.50 dB
PL12	16.21 dB
PL2W	12.76071072 W
PL12W	0.34266910 W
SFO2	400.1316005 MHz
SI	32768
SF	100.6127722 MHz
WDW	EM
SSB	0
LB	1.00 Hz
GB	0
PC	1.40

CWVI-111

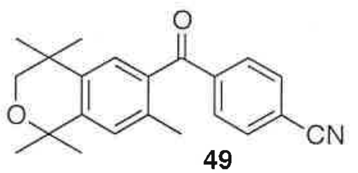
49

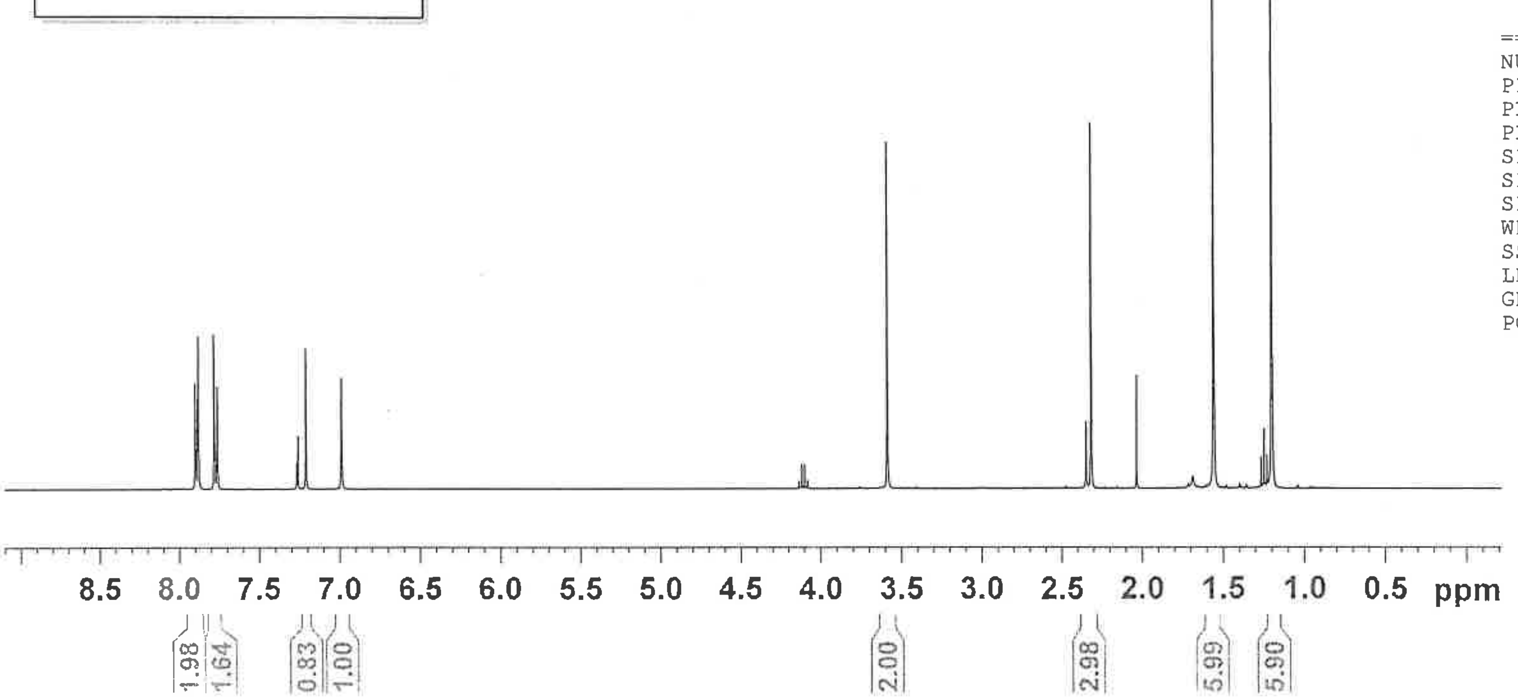

NAME	CWVI-111
EXPNO	1
PROCNO	1
Date_	20180706
Time	16.16
INSTRUM	spect
PROBHD	5 mm PABBO BB-
PULPROG	zg30
TD	65536
SOLVENT	CDCl3
NS	16
DS	2
SWH	8223.685 Hz
FIDRES	0.125483 Hz
AQ	3.9846387 sec
RG	4
DW	60.800 usec
DE	6.50 usec
TE	293.9 K
D1	1.00000000 sec
TD0	1

======== CHANNEL f1 ========

NUC1	1H
P1	14.75 usec
PL1	0.50 dB
PL1W	12.76071072 W
SFO1	400.1324710 MHz
SI	32768
SF	400.1300096 MHz
WDW	EM
SSB	0
LB	0.30 Hz
GB	0
PC	1.00

CWVI-111

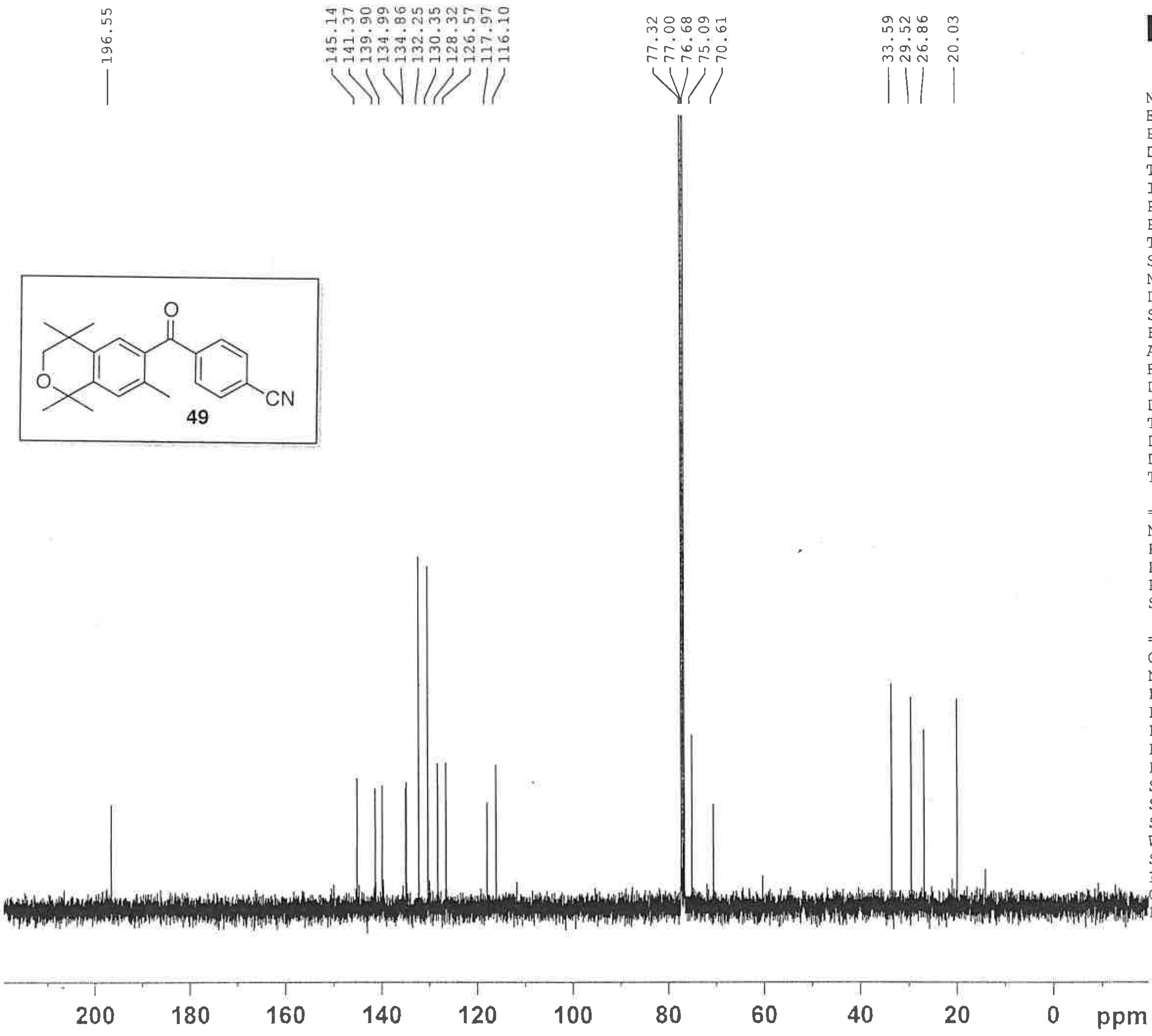

NAME	CWVI-111
EXPNO	2
PROCNO	1
Date_	20180706
Time	16.23
INSTRUM	spect
PROBHD	5 mm PABBO BB-
PULPROG	zgig30
TD	65536
SOLVENT	CDCl3
NS	60
DS	4
SWH	24038.461 Hz
FIDRES	0.366798 Hz
AQ	1.3631988 sec
RG	2050
DW	20.800 usec
DE	6.50 usec
TE	294.1 K
D1	2.00000000 sec
D11	0.03000000 sec
TD0	1
======== CHANNEL f1 ========	
NUC1	13C
P1	8.25 usec
PL1	-2.10 dB
PL1W	60.29227829 W
SFO1	100.6228298 MHz
======== CHANNEL f2 ========	
CPDPRG2	waltz16
NUC2	1H
PCPD2	90.00 usec
PL2	0.50 dB
PL12	16.21 dB
PL2W	12.76071072 W
PL12W	0.34266910 W
SFO2	400.1316005 MHz
SI	32768
SF	100.6127744 MHz
WDW	EM
SSB	0
LB	1.00 Hz
GB	0
PC	1.40

CWVI-139

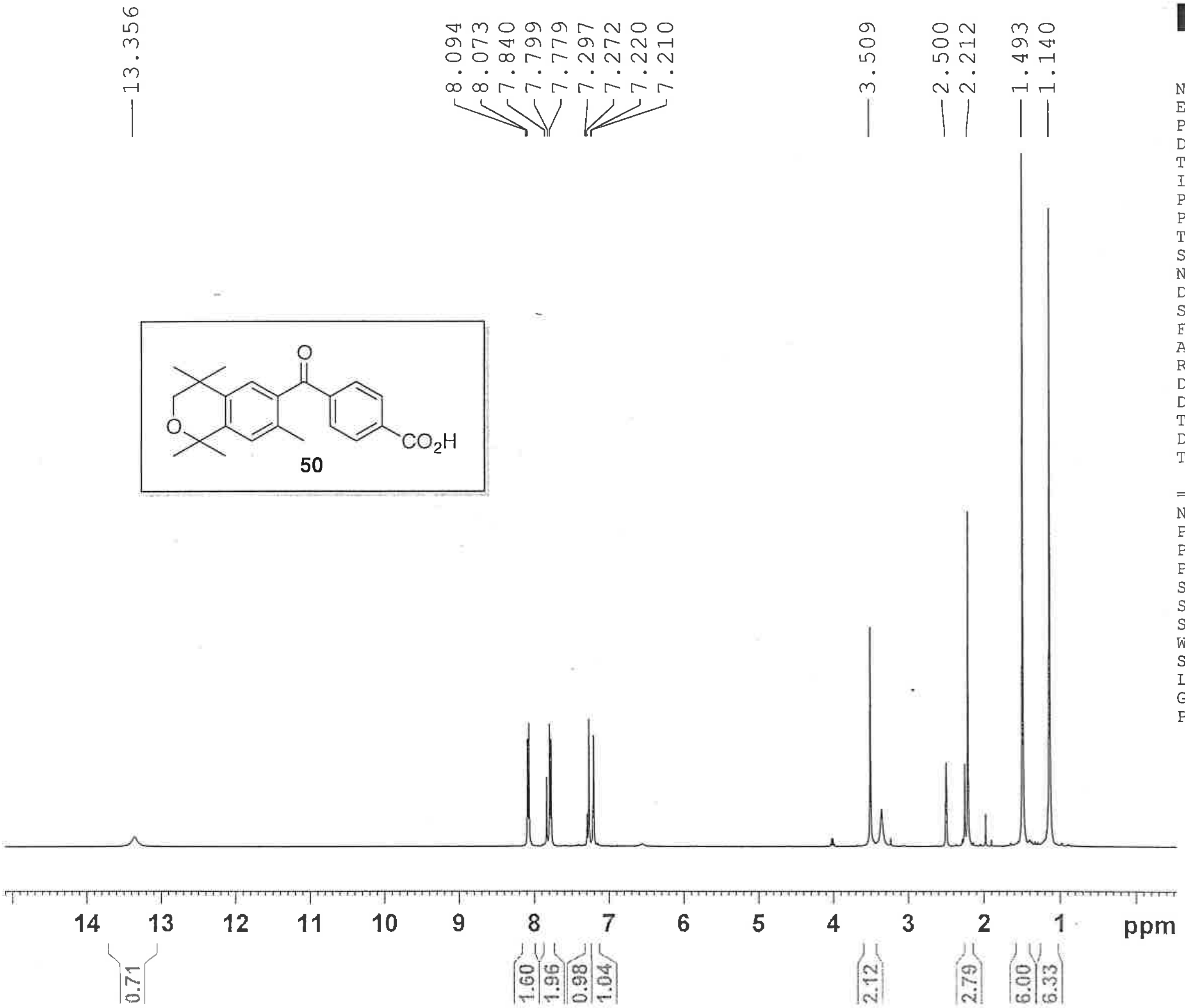

50

BRUKER

NAME	CWVI-139	
EXPNO	1	
PROCNO	1	
Date_	20181010	
Time	21.35	
INSTRUM	spect	
PROBHD	5 mm PABBO BB-	
PULPROG	zg30	
TD	65536	
SOLVENT	DMSO	
NS	16	
DS	2	
SWH	8223.685	Hz
FIDRES	0.125483	Hz
AQ	3.9846387	sec
RG	4	
DW	60.800	usec
DE	6.50	usec
TE	292.2	K
D1	1.00000000	sec
TD0	1	

======== CHANNEL f1 ========

NUC1	1H	
P1	14.75	usec
PL1	0.50	dB
PL1W	12.76071072	W
SFO1	400.1324710	MHz
SI	32768	
SF	400.1300031	MHz
WDW	EM	
SSB	0	
LB	0.30	Hz
GB	0	
PC	1.00	

CWVI-139

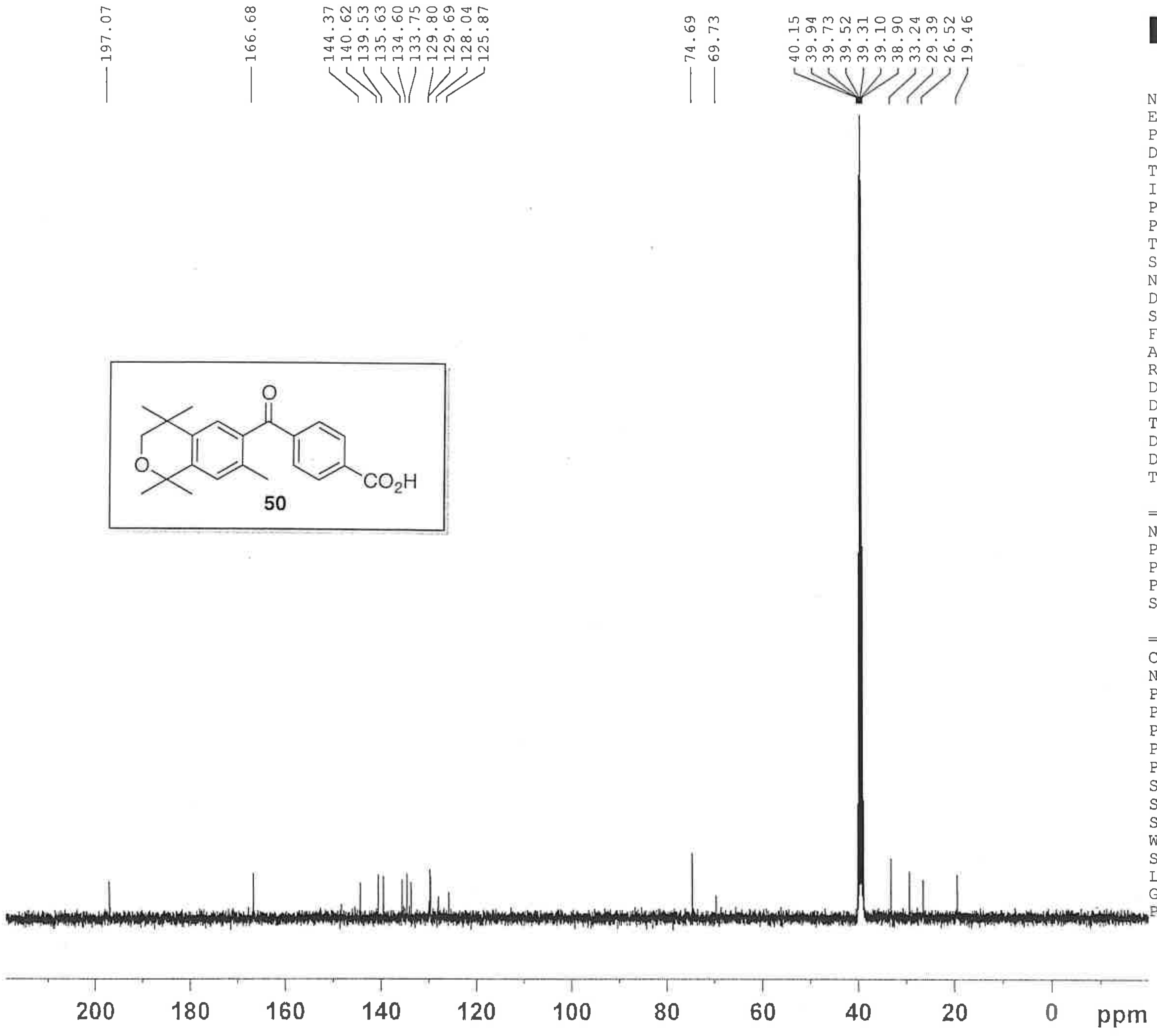

BRUKER

NAME	CWVI-139	
EXPNO	2	
PROCNO	1	
Date_	20181010	
Time	21.40	
INSTRUM	spect	
PROBHD	5 mm PABBO BB-	
PULPROG	zgig30	
TD	65536	
SOLVENT	DMSO	
NS	74	
DS	4	
SWH	24038.461	Hz
FIDRES	0.366798	Hz
AQ	1.3631988	sec
RG	2050	
DW	20.800	usec
DE	6.50	usec
TE	292.4	K
D1	2.00000000	sec
D11	0.03000000	sec
TD0	1	
======== CHANNEL f1 ========		
NUC1	13C	
P1	8.25	usec
PL1	-2.10	dB
PL1W	60.29227829	W
SFO1	100.6228298	MHz
======== CHANNEL f2 ========		
CPDPRG2	waltz16	
NUC2	1H	
PCPD2	90.00	usec
PL2	0.50	dB
PL12	16.21	dB
PL2W	12.76071072	W
PL12W	0.34266910	W
SFO2	400.1316005	MHz
SI	32768	
SF	100.6128113	MHz
WDW	EM	
SSB	0	
LB	1.00	Hz
GB	0	
PC	1.40	

CWVI-129

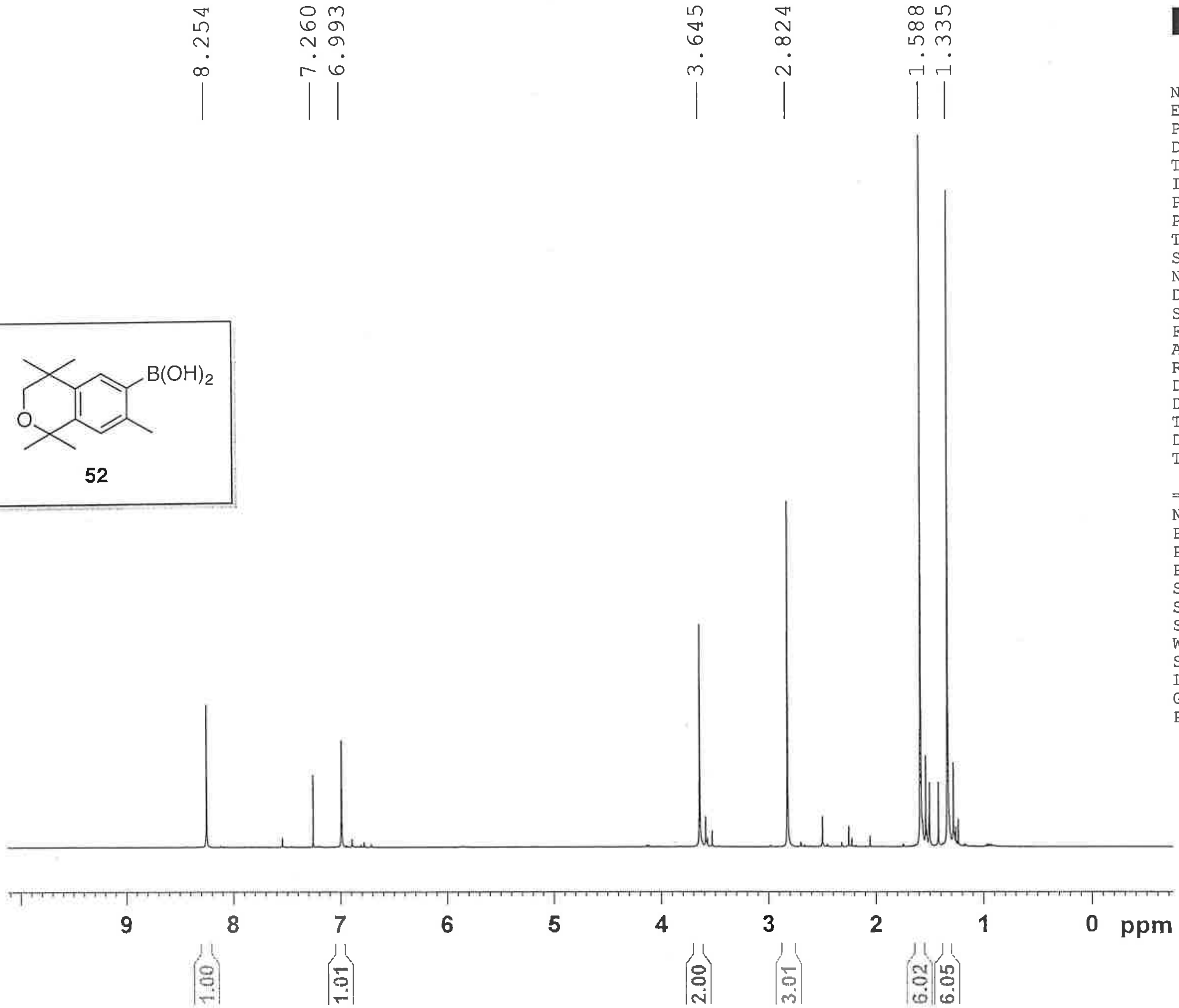

52

NAME	CWVI-129
EXPNO	1
PROCNO	1
Date_	20180725
Time	14.13
INSTRUM	spect
PROBHD	5 mm PABBO BB-
PULPROG	zg30
TD	65536
SOLVENT	CDCl3
NS	16
DS	2
SWH	8223.685 Hz
FIDRES	0.125483 Hz
AQ	3.9846387 sec
RG	4
DW	60.800 usec
DE	6.50 usec
TE	294.6 K
D1	1.00000000 sec
TD0	1

======== CHANNEL f1 ========

NUC1	1H
P1	14.75 usec
PL1	0.50 dB
PL1W	12.76071072 W
SFO1	400.1324710 MHz
SI	32768
SF	400.1300099 MHz
WDW	EM
SSB	0
LB	0.30 Hz
GB	0
PC	1.00

CWVI-129

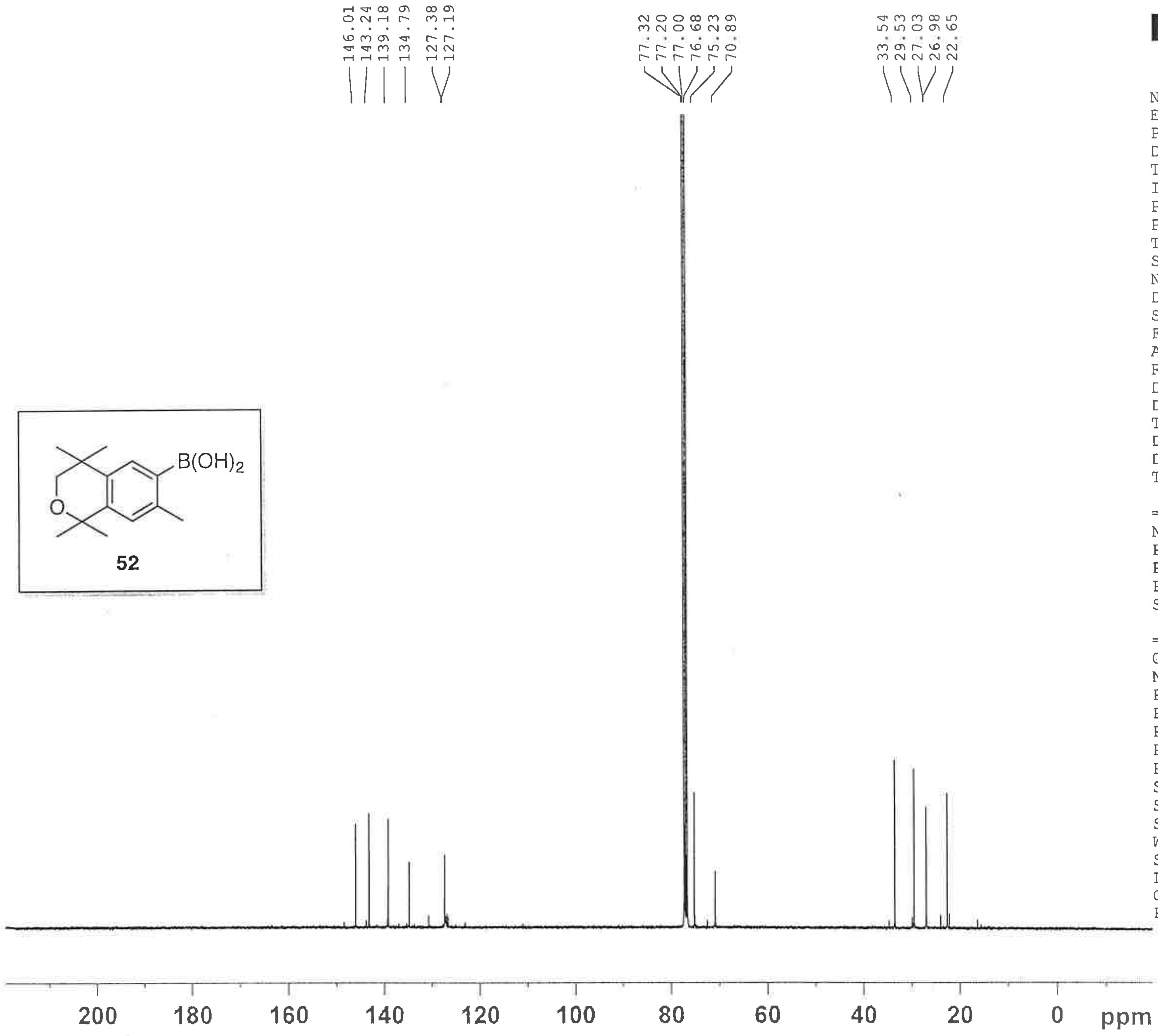

52

BRUKER

NAME	CWVI-129
EXPNO	3
PROCNO	1
Date_	20180727
Time	15.19
INSTRUM	spect
PROBHD	5 mm PABBO BB-
PULPROG	zgig30
TD	65536
SOLVENT	CDCl3
NS	5359
DS	4
SWH	24038.461 Hz
FIDRES	0.366798 Hz
AQ	1.3631988 sec
RG	2050
DW	20.800 usec
DE	6.50 usec
TE	295.7 K
D1	2.00000000 sec
D11	0.03000000 sec
TD0	1
======== CHANNEL f1 ========	
NUC1	13C
P1	8.25 usec
PL1	-2.10 dB
PL1W	60.29227829 W
SFO1	100.6228298 MHz
======== CHANNEL f2 ========	
CPDPRG2	waltz16
NUC2	1H
PCPD2	90.00 usec
PL2	0.50 dB
PL12	16.21 dB
PL2W	12.76071072 W
PL12W	0.34266910 W
SFO2	400.1316005 MHz
SI	32768
SF	100.6127729 MHz
WDW	EM
SSB	0
LB	1.00 Hz
GB	0
PC	1.40

CWVI-131 (pure)

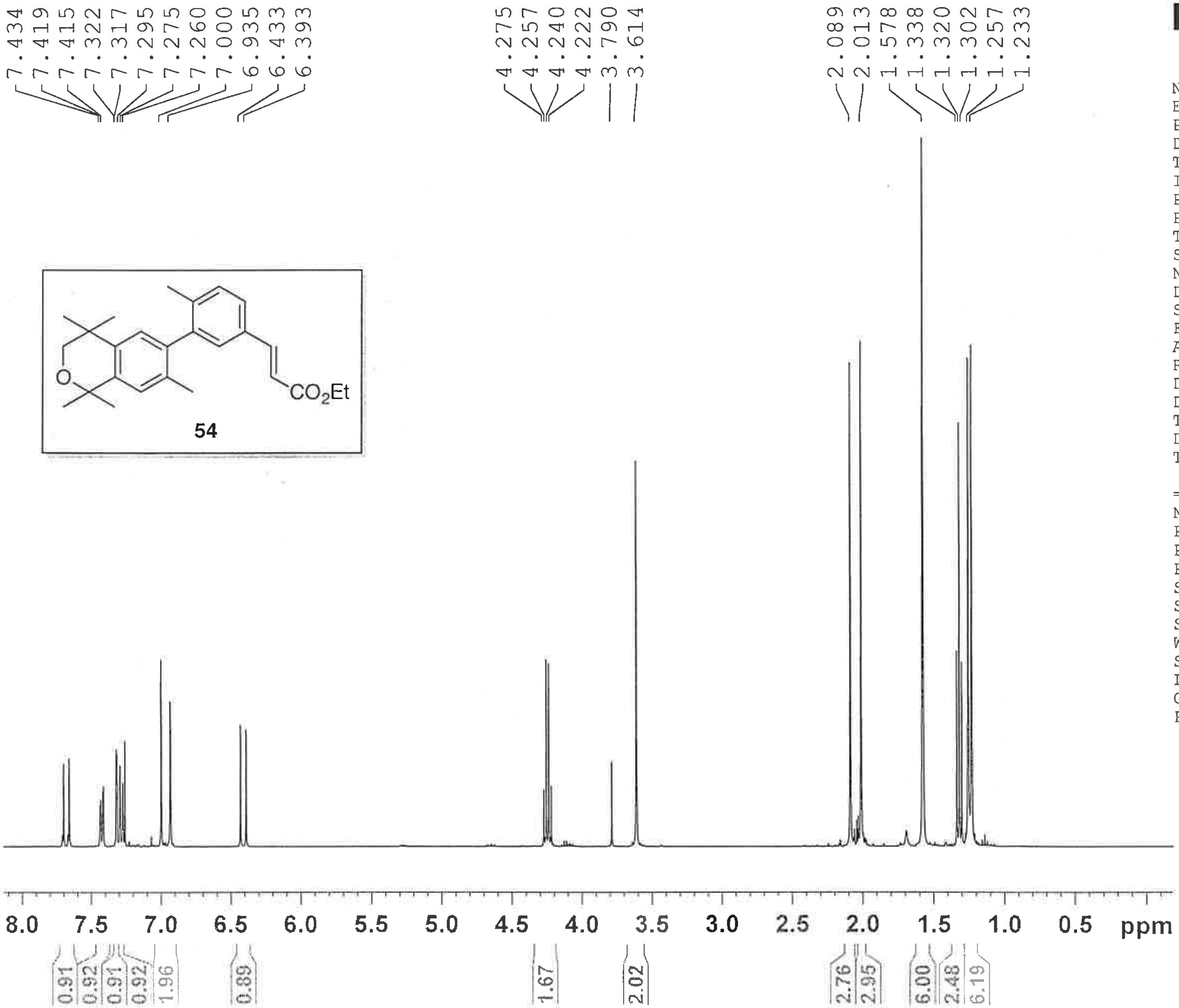

54

NAME	CWVI-131
EXPNO	1
PROCNO	1
Date_	20180712
Time	10.23
INSTRUM	spect
PROBHD	5 mm PABBO BB-
PULPROG	zg30
TD	65536
SOLVENT	CDCl3
NS	16
DS	2
SWH	8223.685 Hz
FIDRES	0.125483 Hz
AQ	3.9846387 sec
RG	4
DW	60.800 usec
DE	6.50 usec
TE	295.1 K
D1	1.00000000 sec
TD0	1

======== CHANNEL f1 ========

NUC1	1H
P1	14.75 usec
PL1	0.50 dB
PL1W	12.76071072 W
SFO1	400.1324710 MHz
SI	32768
SF	400.1300099 MHz
WDW	EM
SSB	0
LB	0.30 Hz
GB	0
PC	1.00

CWVI-131 (pure)

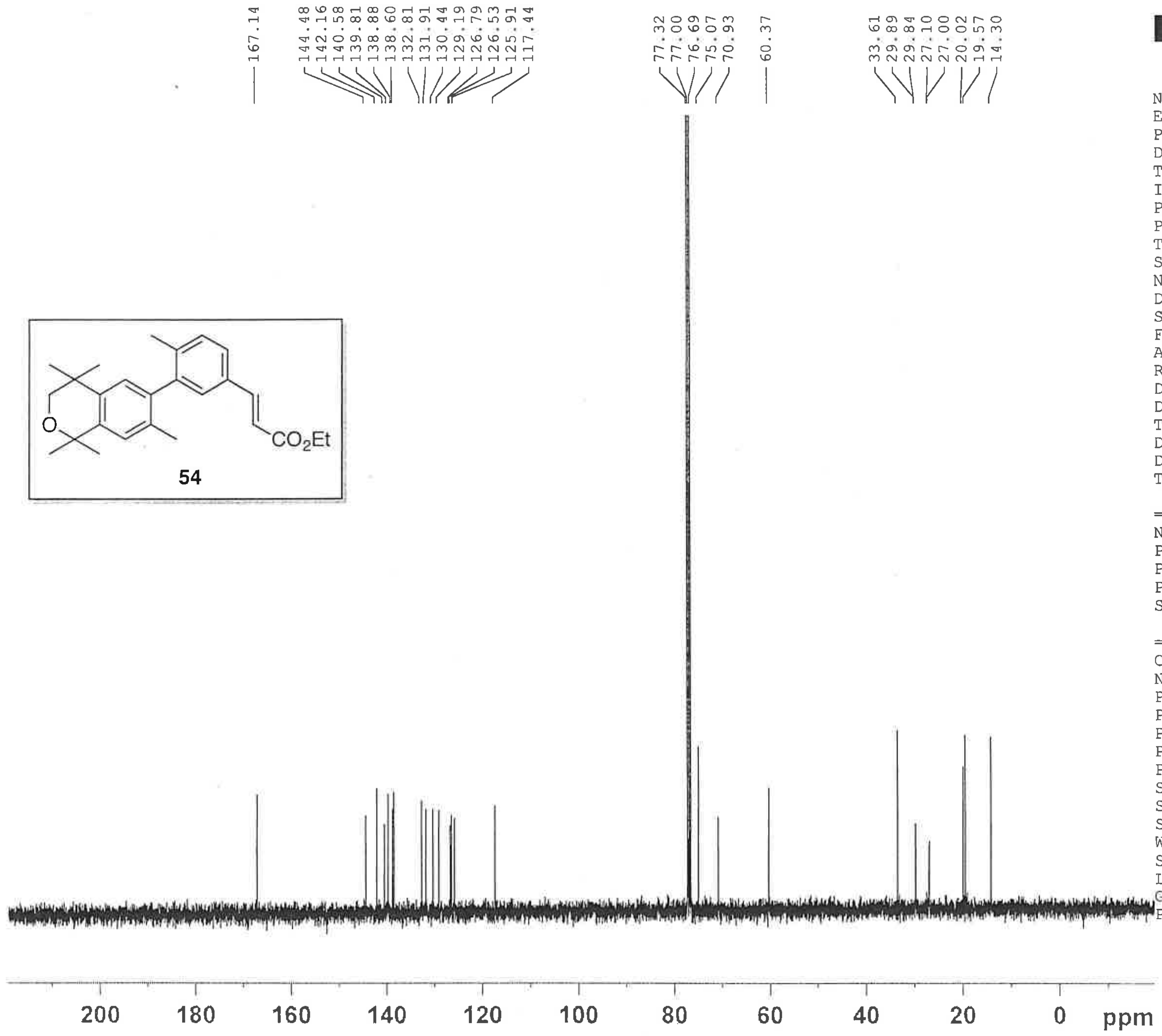

54

BRUKER

NAME	CWVI-131
EXPNO	2
PROCNO	1
Date_	20180712
Time	10.32
INSTRUM	spect
PROBHD	5 mm PABBO BB-
PULPROG	zgig30
TD	65536
SOLVENT	CDCl3
NS	143
DS	4
SWH	24038.461 Hz
FIDRES	0.366798 Hz
AQ	1.3631988 sec
RG	2050
DW	20.800 usec
DE	6.50 usec
TE	295.4 K
D1	2.00000000 sec
D11	0.03000000 sec
TD0	1
======== CHANNEL f1 ========	
NUC1	13C
P1	8.25 usec
PL1	-2.10 dB
PL1W	60.29227829 W
SFO1	100.6228298 MHz
======== CHANNEL f2 ========	
CPDPRG2	waltz16
NUC2	1H
PCPD2	90.00 usec
PL2	0.50 dB
PL12	16.21 dB
PL2W	12.76071072 W
PL12W	0.34266910 W
SFO2	400.1316005 MHz
SI	32768
SF	100.6127729 MHz
WDW	EM
SSB	0
LB	1.00 Hz
GB	0
PC	1.40

CWVI-135

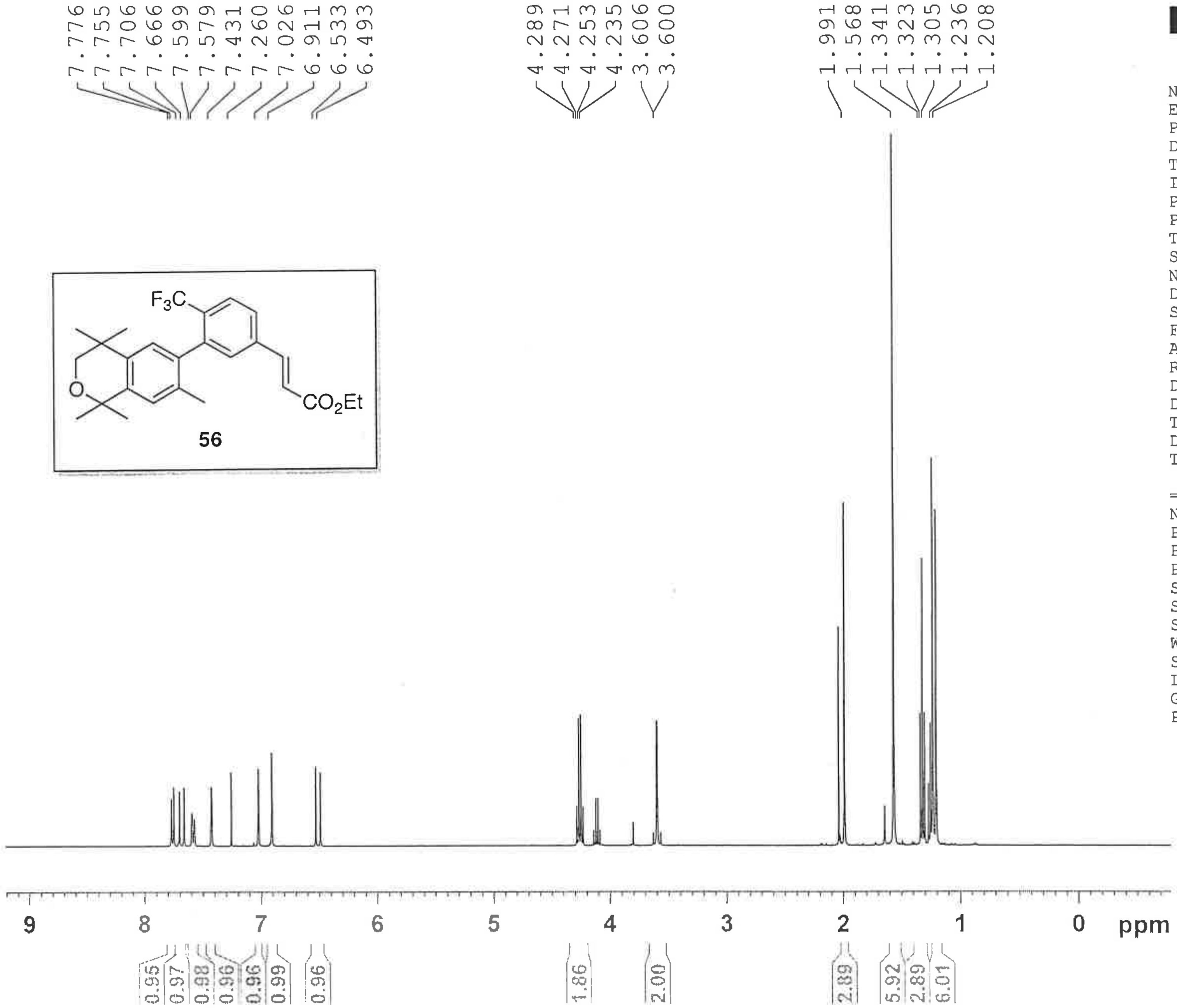

56

NAME	CWVI-135
EXPNO	1
PROCNO	1
Date_	20180712
Time	10.40
INSTRUM	spect
PROBHD	5 mm PABBO BB-
PULPROG	zg30
TD	65536
SOLVENT	CDCl3
NS	16
DS	2
SWH	8223.685 Hz
FIDRES	0.125483 Hz
AQ	3.9846387 sec
RG	4
DW	60.800 usec
DE	6.50 usec
TE	295.1 K
D1	1.00000000 sec
TD0	1
======== CHANNEL f1 ========	
NUC1	1H
P1	14.75 usec
PL1	0.50 dB
PL1W	12.76071072 W
SFO1	400.1324710 MHz
SI	32768
SF	400.1300099 MHz
WDW	EM
SSB	0
LB	0.30 Hz
GB	0
PC	1.00

CWVI-135

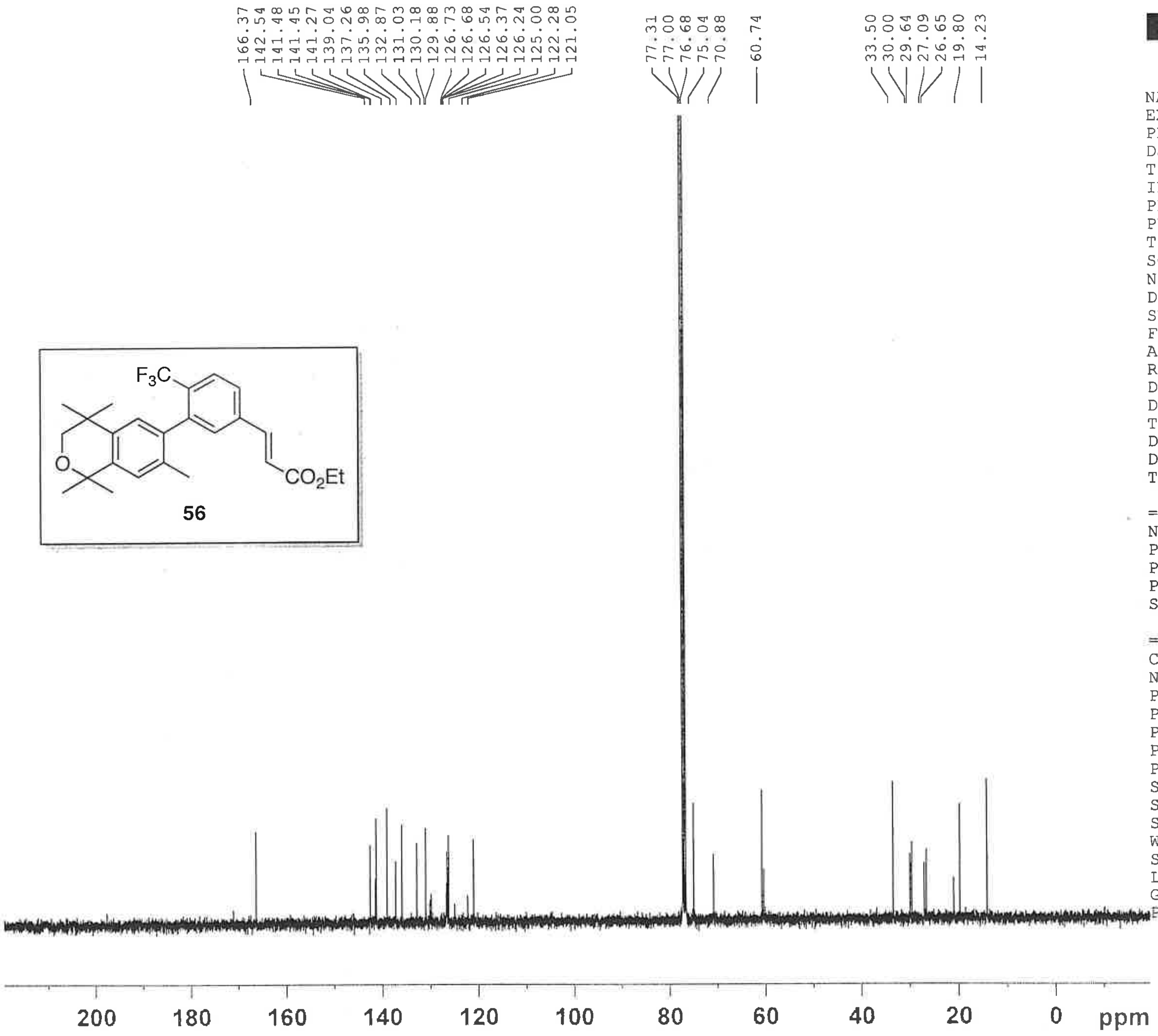

56

NAME	CWVI-135
EXPNO	2
PROCNO	1
Date_	20180712
Time	10.48
INSTRUM	spect
PROBHD	5 mm PABBO BB-
PULPROG	zgig30
TD	65536
SOLVENT	CDCl3
NS	363
DS	4
SWH	24038.461 Hz
FIDRES	0.366798 Hz
AQ	1.3631988 sec
RG	2050
DW	20.800 usec
DE	6.50 usec
TE	295.5 K
D1	2.00000000 sec
D11	0.03000000 sec
TD0	1
======== CHANNEL f1 ========	
NUC1	13C
P1	8.25 usec
PL1	-2.10 dB
PL1W	60.29227829 W
SFO1	100.6228298 MHz
======== CHANNEL f2 ========	
CPDPRG2	waltz16
NUC2	1H
PCPD2	90.00 usec
PL2	0.50 dB
PL12	16.21 dB
PL2W	12.76071072 W
PL12W	0.34266910 W
SFO2	400.1316005 MHz
SI	32768
SF	100.6127722 MHz
WDW	EM
SSB	0
LB	1.00 Hz
GB	0
PC	1.40

CWVI-133

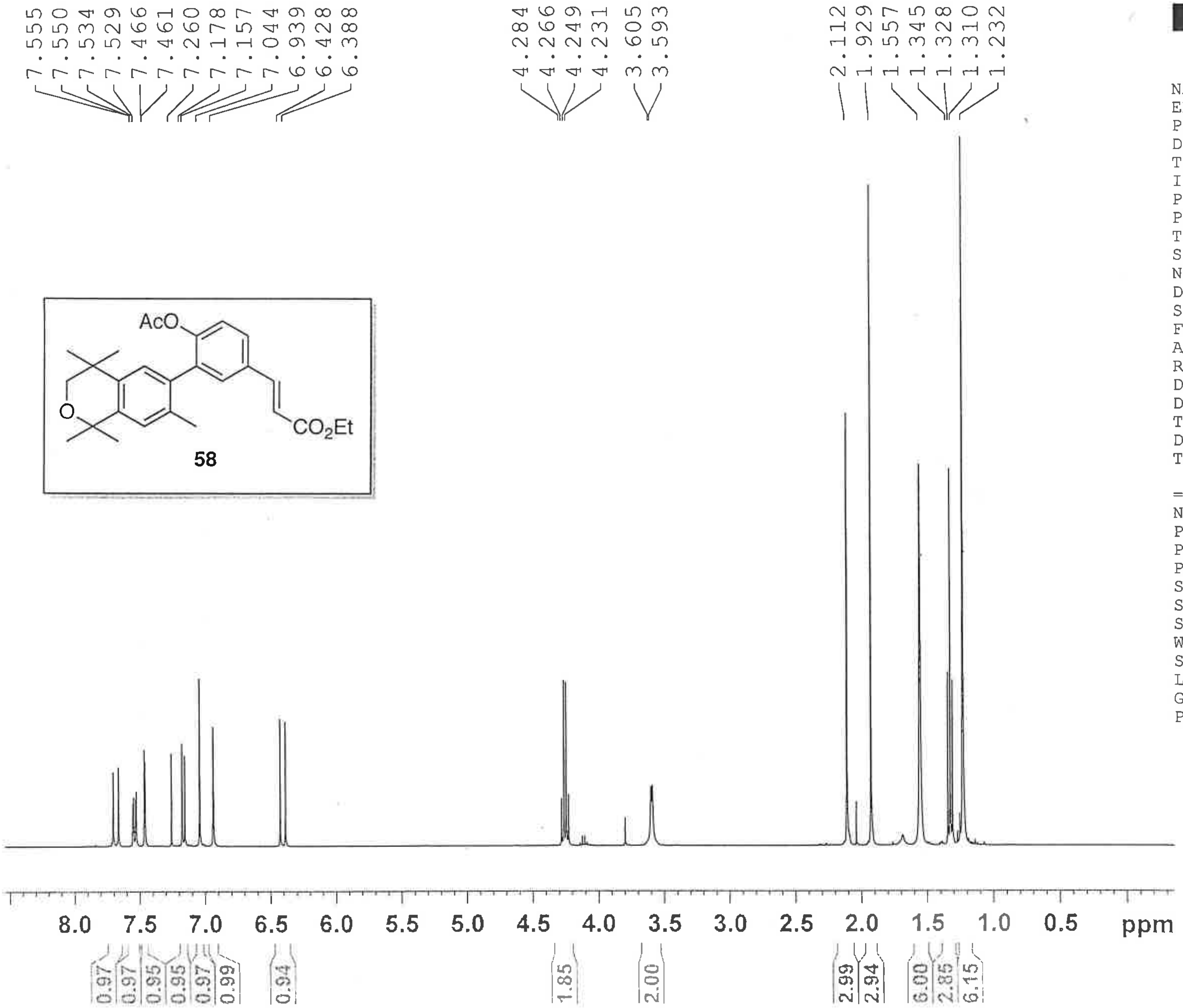

NAME	CWVI-133	
EXPNO	1	
PROCNO	1	
Date_	20180717	
Time_	16.24	
INSTRUM	spect	
PROBHD	5 mm PABBO BB-	
PULPROG	zg30	
TD	65536	
SOLVENT	CDCl3	
NS	16	
DS	2	
SWH	8223.685	Hz
FIDRES	0.125483	Hz
AQ	3.9846387	sec
RG	4	
DW	60.800	usec
DE	6.50	usec
TE	294.7	K
D1	1.00000000	sec
TD0	1	
======== CHANNEL f1 ========		
NUC1	1H	
P1	14.75	usec
PL1	0.50	dB
PL1W	12.76071072	W
SFO1	400.1324710	MHz
SI	32768	
SF	400.1300099	MHz
WDW	EM	
SSB	0	
LB	0.30	Hz
GB	0	
PC	1.00	

CWVI-133

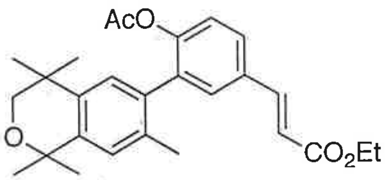

58

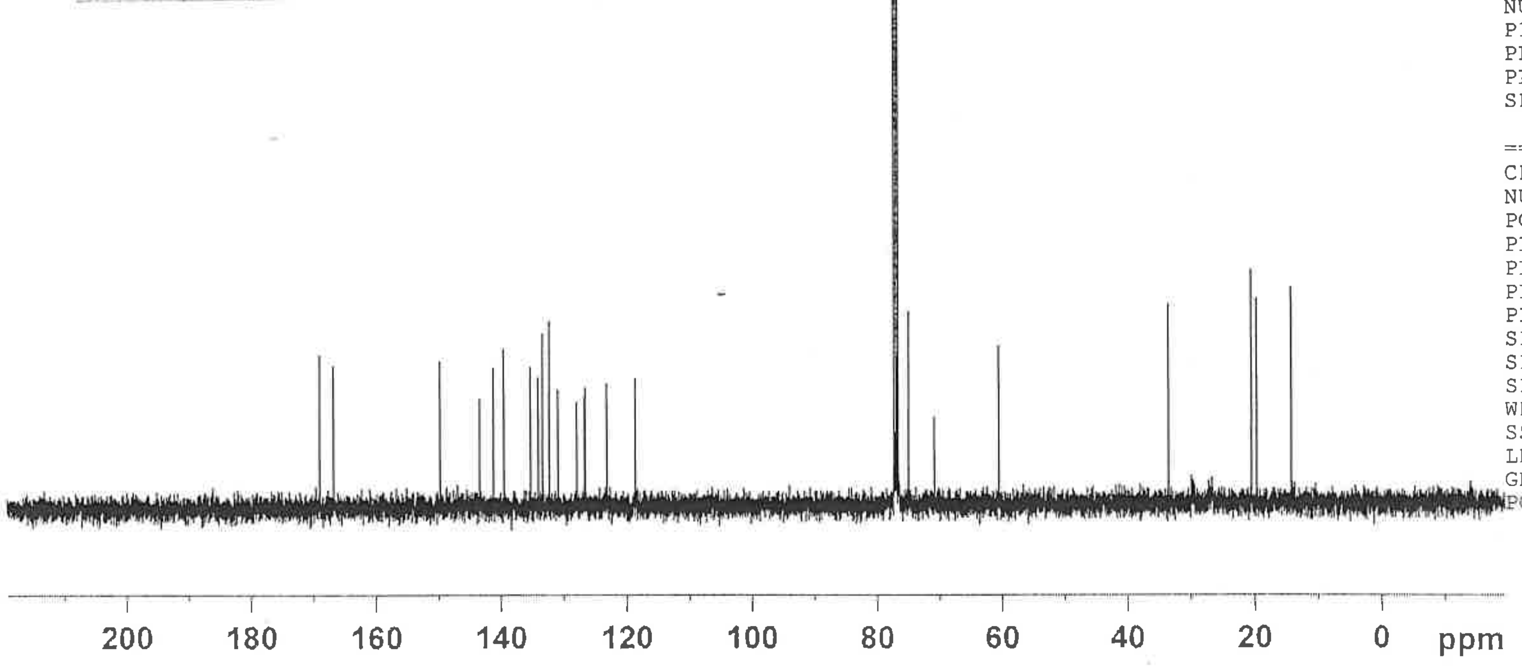

NAME	CWVI-133
EXPNO	2
PROCNO	1
Date_	20180717
Time	16.31
INSTRUM	spect
PROBHD	5 mm PABBO BB-
PULPROG	zgig30
TD	65536
SOLVENT	CDCl3
NS	128
DS	4
SWH	24038.461 Hz
FIDRES	0.366798 Hz
AQ	1.3631988 sec
RG	2050
DW	20.800 usec
DE	6.50 usec
TE	295.0 K
D1	2.00000000 sec
D11	0.03000000 sec
TD0	1
======== CHANNEL f1 ========	
NUC1	13C
P1	8.25 usec
PL1	-2.10 dB
PL1W	60.29227829 W
SFO1	100.6228298 MHz
======== CHANNEL f2 ========	
CPDPRG2	waltz16
NUC2	1H
PCPD2	90.00 usec
PL2	0.50 dB
PL12	16.21 dB
PL2W	12.76071072 W
PL12W	0.34266910 W
SFO2	400.1316005 MHz
SI	32768
SF	100.6127736 MHz
WDW	EM
SSB	0
LB	1.00 Hz
GB	0
PC	1.40

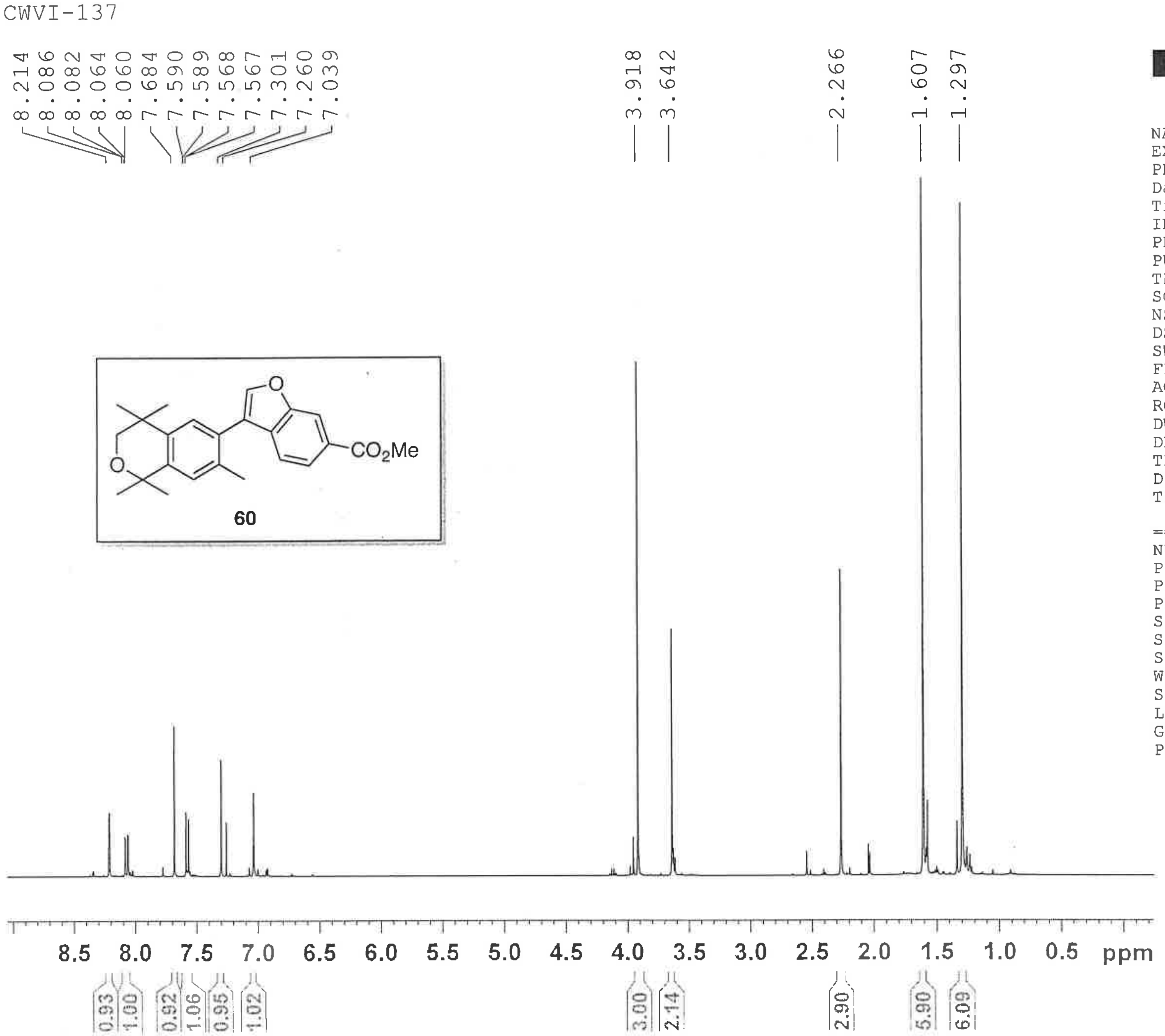

NAME	CWVI-137
EXPNO	1
PROCNO	1
Date_	20180717
Time	16.39
INSTRUM	spect
PROBHD	5 mm PABBO BB-
PULPROG	zg30
TD	65536
SOLVENT	CDCl3
NS	16
DS	2
SWH	8223.685 Hz
FIDRES	0.125483 Hz
AQ	3.9846387 sec
RG	4
DW	60.800 usec
DE	6.50 usec
TE	294.7 K
D1	1.00000000 sec
TD0	1

======== CHANNEL f1 ========

NUC1	1H
P1	14.75 usec
PL1	0.50 dB
PL1W	12.76071072 W
SFO1	400.1324710 MHz
SI	32768
SF	400.1300099 MHz
WDW	EM
SSB	0
LB	0.30 Hz
GB	0
PC	1.00

CWVI-137

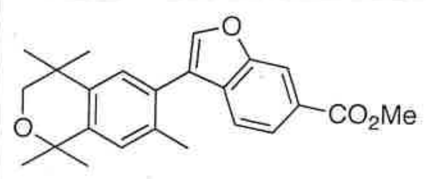

60

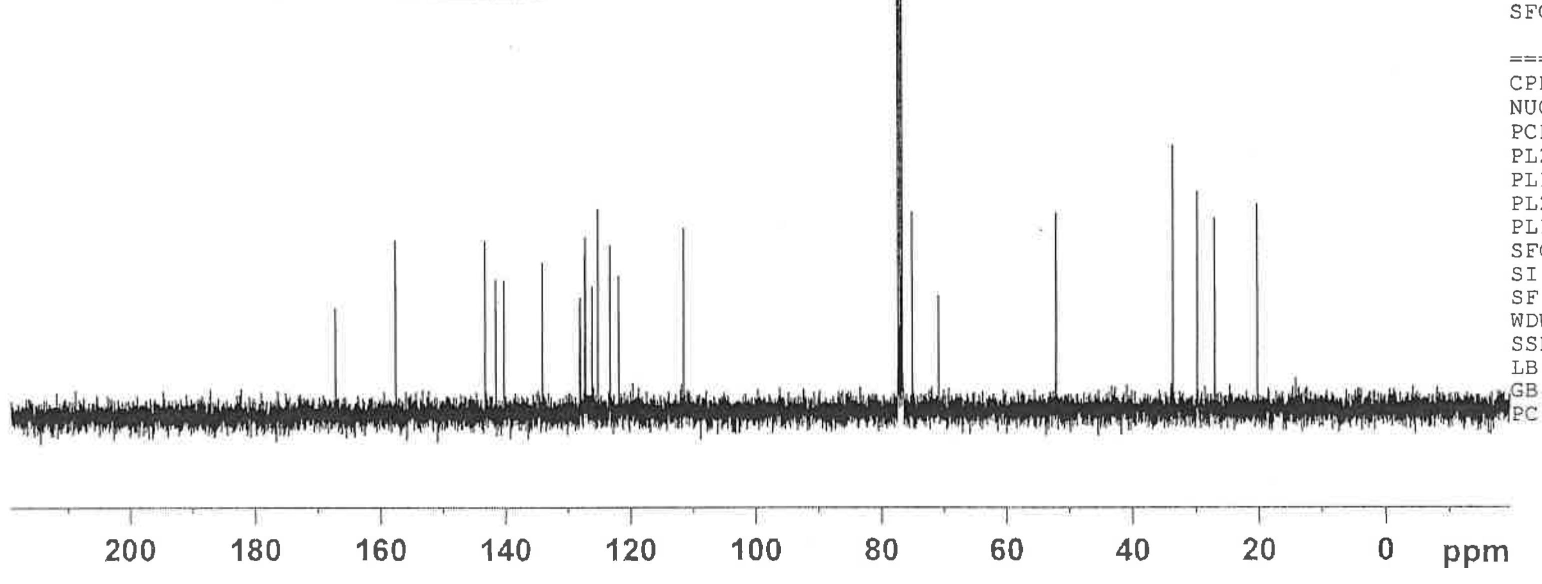

NAME	CWVI-137
EXPNO	2
PROCNO	1
Date_	20180717
Time_	16.43
INSTRUM	spect
PROBHD	5 mm PABBO BB-
PULPROG	zgig30
TD	65536
SOLVENT	CDCl3
NS	82
DS	4
SWH	24038.461 Hz
FIDRES	0.366798 Hz
AQ	1.3631988 sec
RG	2050
DW	20.800 usec
DE	6.50 usec
TE	295.0 K
D1	2.00000000 sec
D11	0.03000000 sec
TD0	1

======== CHANNEL f1 ========

NUC1	13C
P1	8.25 usec
PL1	-2.10 dB
PL1W	60.29227829 W
SFO1	100.6228298 MHz

======== CHANNEL f2 ========

CPDPRG2	waltz16
NUC2	1H
PCPD2	90.00 usec
PL2	0.50 dB
PL12	16.21 dB
PL2W	12.76071072 W
PL12W	0.34266910 W
SFO2	400.1316005 MHz
SI	32768
SF	100.6127736 MHz
WDW	EM
SSB	0
LB	1.00 Hz
GB	0
PC	1.40

CWVI-185

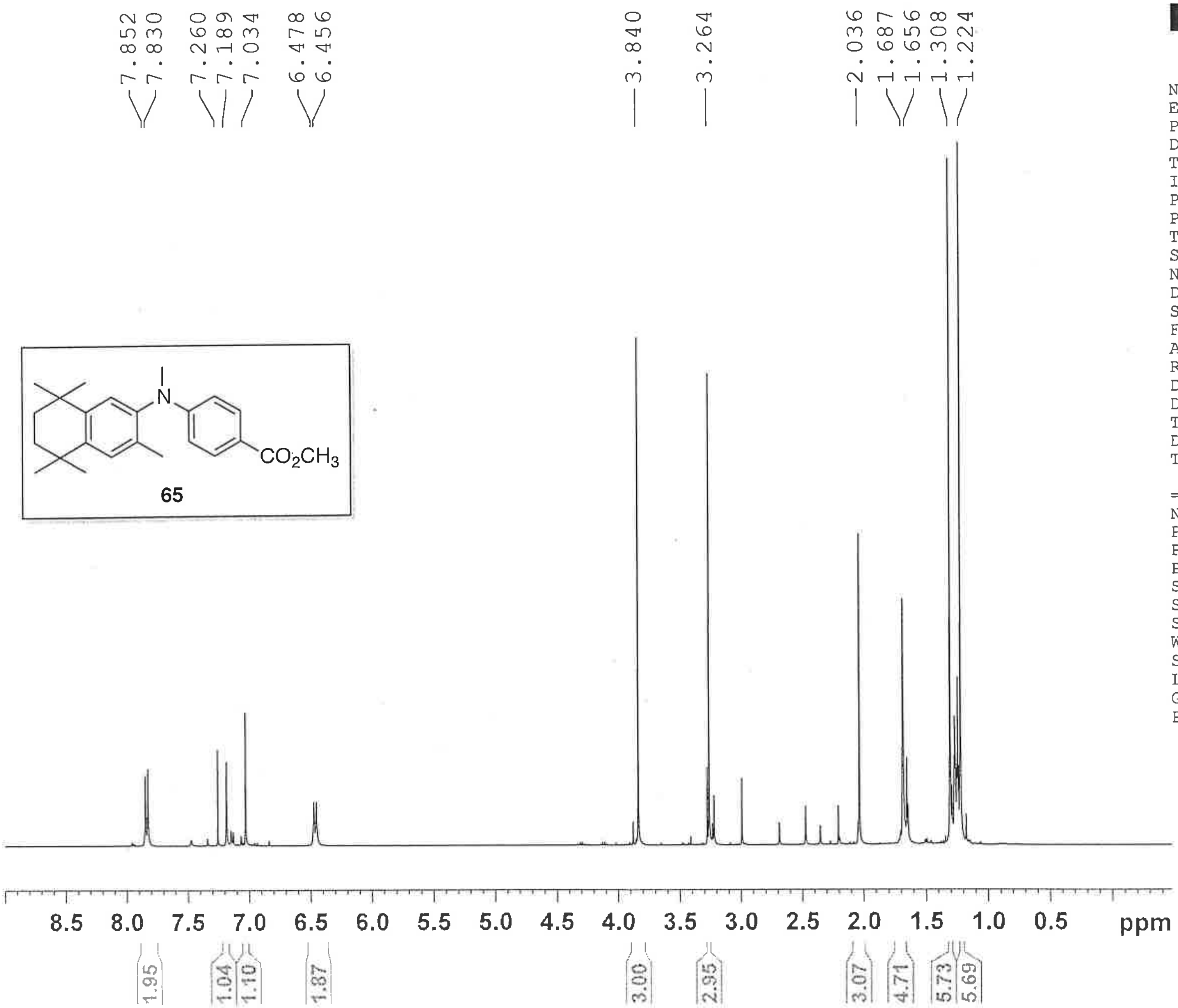

NAME	CWVI-185
EXPNO	1
PROCNO	1
Date_	20180828
Time	14.02
INSTRUM	spect
PROBHD	5 mm PABBO BB-
PULPROG	zg30
TD	65536
SOLVENT	CDCl3
NS	16
DS	2
SWH	8223.685 Hz
FIDRES	0.125483 Hz
AQ	3.9846387 sec
RG	4
DW	60.800 usec
DE	6.50 usec
TE	293.0 K
D1	1.00000000 sec
TD0	1

======== CHANNEL f1 ========

NUC1	1H
P1	14.75 usec
PL1	0.50 dB
PL1W	12.76071072 W
SFO1	400.1324710 MHz
SI	32768
SF	400.1300099 MHz
WDW	EM
SSB	0
LB	0.30 Hz
GB	0
PC	1.00

CWVI-185

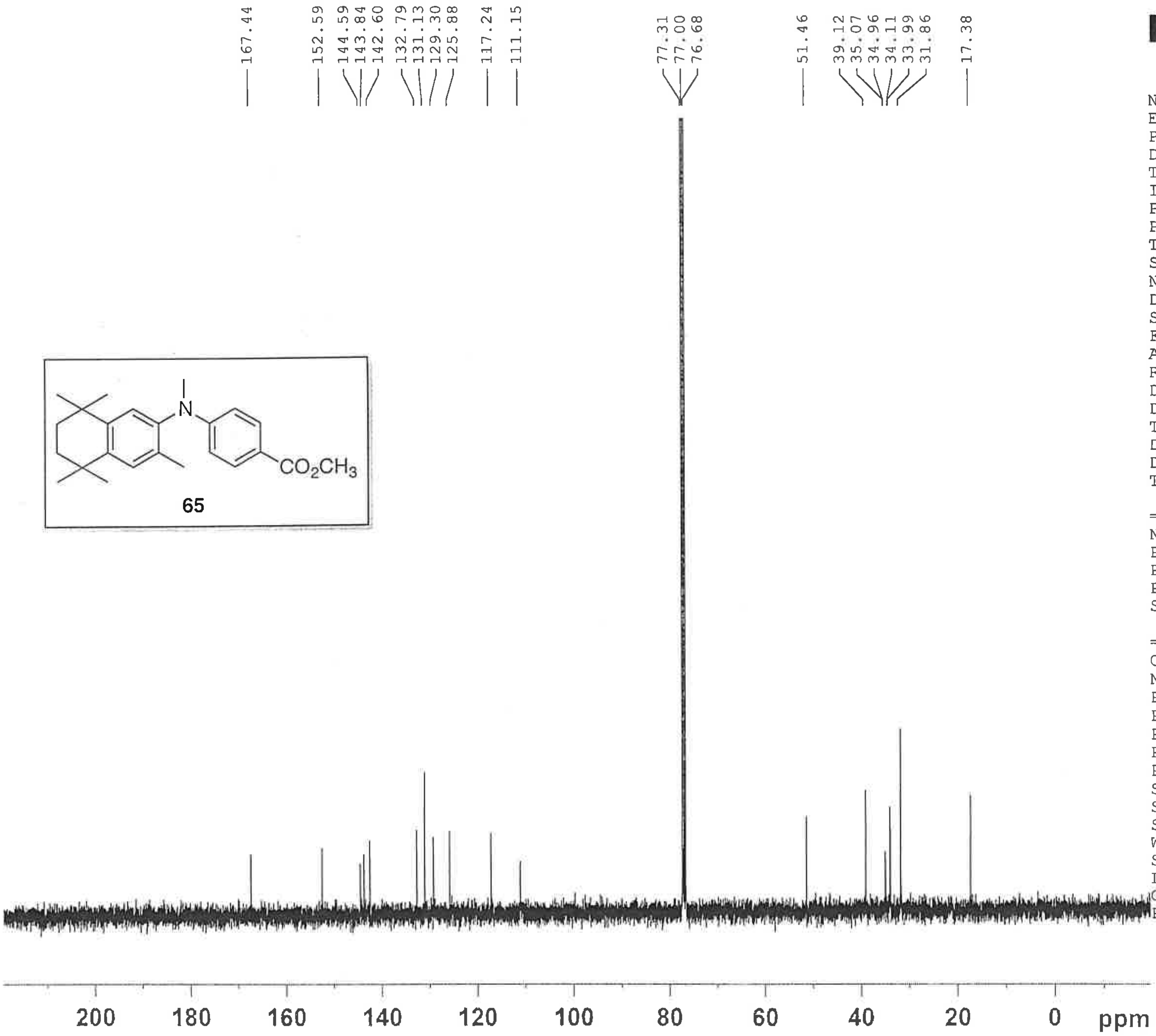

65

NAME	CWVI-185
EXPNO	2
PROCNO	1
Date_	20180828
Time	14.06
INSTRUM	spect
PROBHD	5 mm PABBO BB-
PULPROG	zgig30
TD	65536
SOLVENT	CDCl3
NS	102
DS	4
SWH	24038.461 Hz
FIDRES	0.366798 Hz
AQ	1.3631988 sec
RG	2050
DW	20.800 usec
DE	6.50 usec
TE	293.3 K
D1	2.00000000 sec
D11	0.03000000 sec
TD0	1

======== CHANNEL f1 ========

NUC1	13C
P1	8.25 usec
PL1	-2.10 dB
PL1W	60.29227829 W
SFO1	100.6228298 MHz

======== CHANNEL f2 ========

CPDPRG2	waltz16
NUC2	1H
PCPD2	90.00 usec
PL2	0.50 dB
PL12	16.21 dB
PL2W	12.76071072 W
PL12W	0.34266910 W
SFO2	400.1316005 MHz
SI	32768
SF	100.6127744 MHz
WDW	EM
SSB	0
LB	1.00 Hz
GB	0
PC	1.40

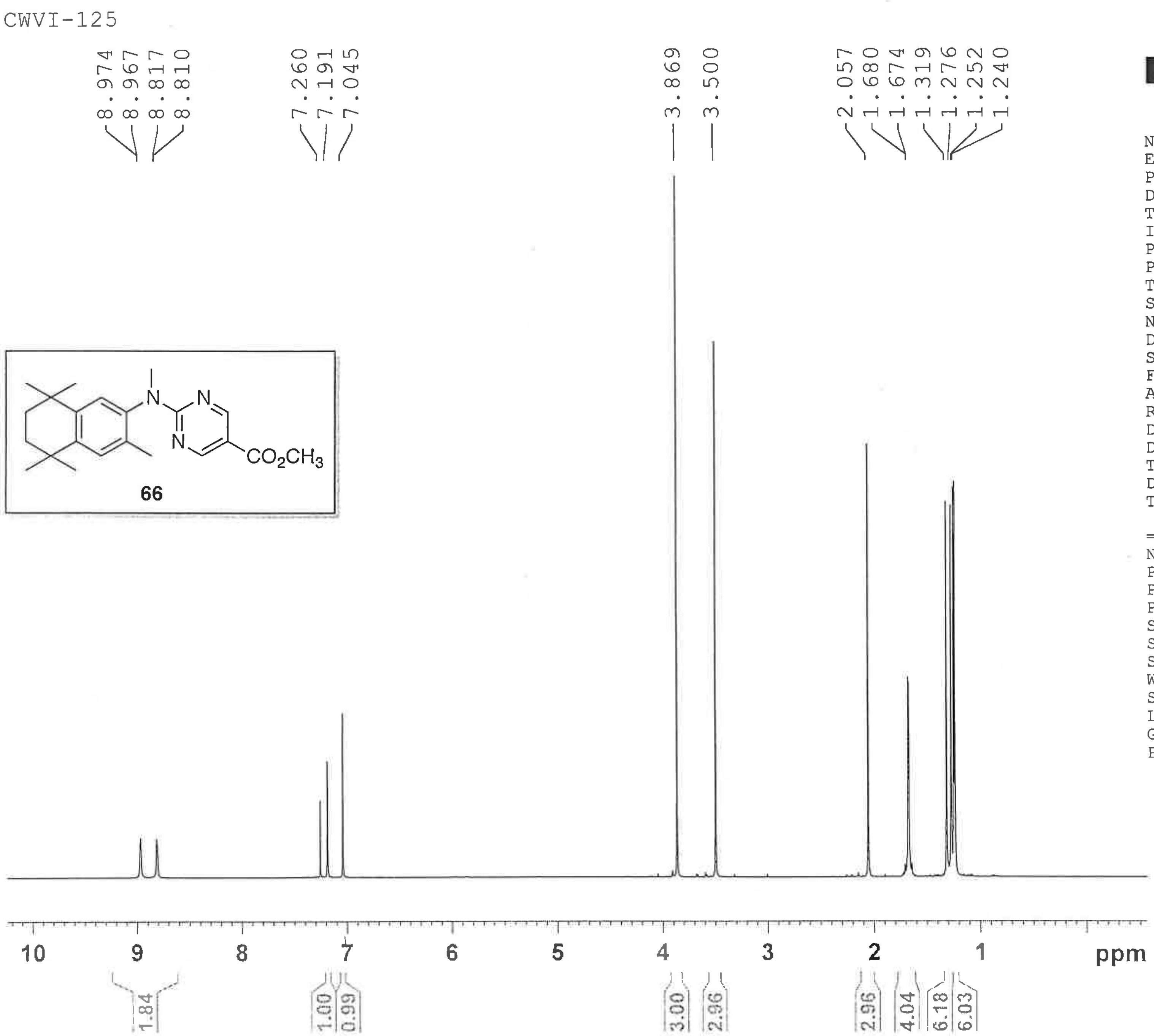

BRUKER

NAME	CWVI-125
EXPNO	1
PROCNO	1
Date_	20180725
Time	20.59
INSTRUM	spect
PROBHD	5 mm PABBO BB-
PULPROG	zg30
TD	65536
SOLVENT	CDCl3
NS	16
DS	2
SWH	8223.685 Hz
FIDRES	0.125483 Hz
AQ	3.9846387 sec
RG	4
DW	60.800 usec
DE	6.50 usec
TE	295.0 K
D1	1.00000000 sec
TD0	1

======== CHANNEL f1 ========

NUC1	1H
P1	14.75 usec
PL1	0.50 dB
PL1W	12.76071072 W
SFO1	400.1324710 MHz
SI	32768
SF	400.1300099 MHz
WDW	EM
SSB	0
LB	0.30 Hz
GB	0
PC	1.00

CWVI-125

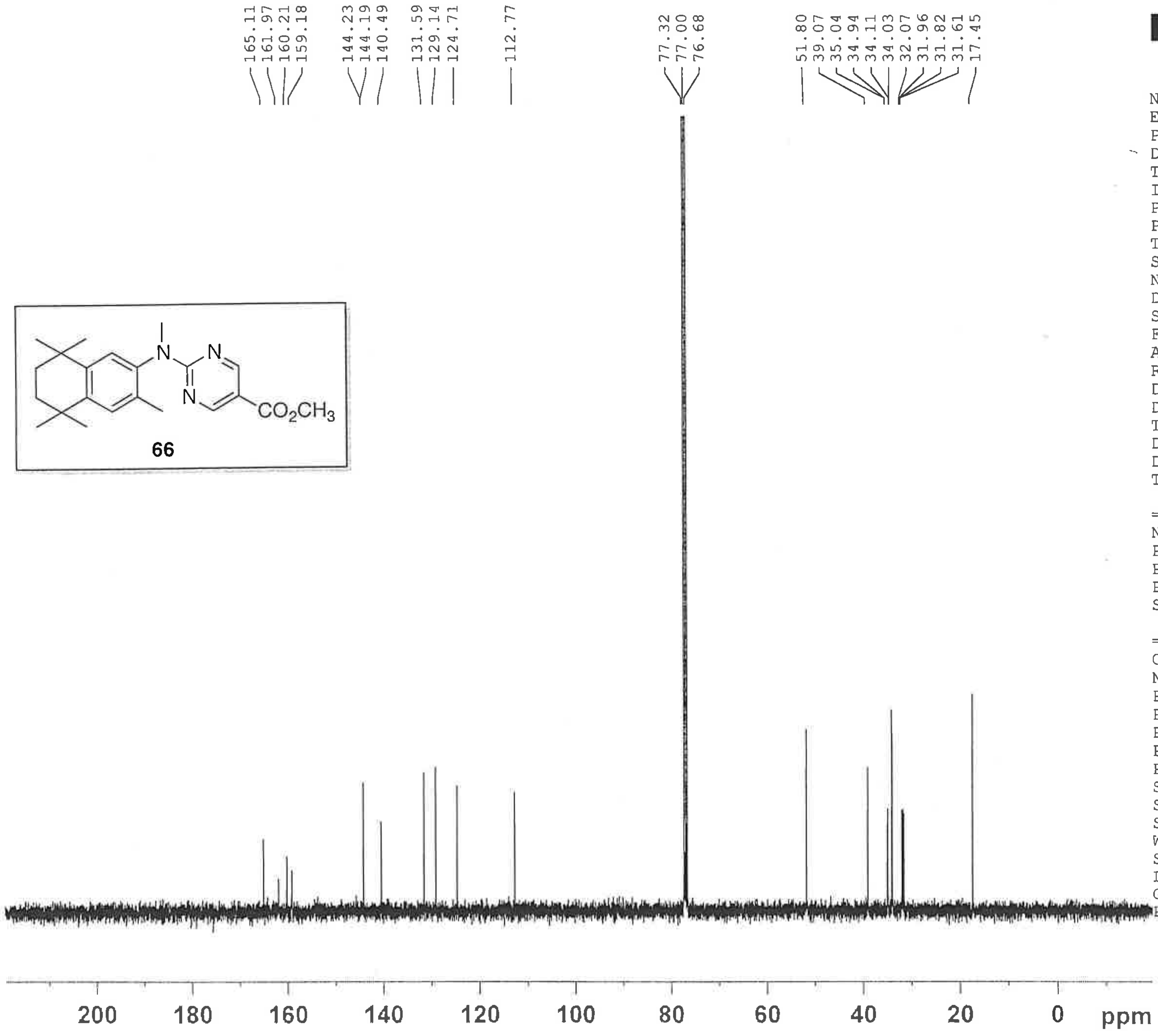

BRUKER

NAME	CWVI-125
EXPNO	2
PROCNO	1
Date_	20180725
Time_	21.02
INSTRUM	spect
PROBHD	5 mm PABBO BB-
PULPROG	zgig30
TD	65536
SOLVENT	CDCl3
NS	166
DS	4
SWH	24038.461 Hz
FIDRES	0.366798 Hz
AQ	1.3631988 sec
RG	2050
DW	20.800 usec
DE	6.50 usec
TE	295.2 K
D1	2.00000000 sec
D11	0.03000000 sec
TD0	1
======== CHANNEL f1 ========	
NUC1	13C
P1	8.25 usec
PL1	-2.10 dB
PL1W	60.29227829 W
SFO1	100.6228298 MHz
======== CHANNEL f2 ========	
CPDPRG2	waltz16
NUC2	1H
PCPD2	90.00 usec
PL2	0.50 dB
PL12	16.21 dB
PL2W	12.76071072 W
PL12W	0.34266910 W
SFO2	400.1316005 MHz
SI	32768
SF	100.6127736 MHz
WDW	EM
SSB	0
LB	1.00 Hz
GB	0
PC	1.40

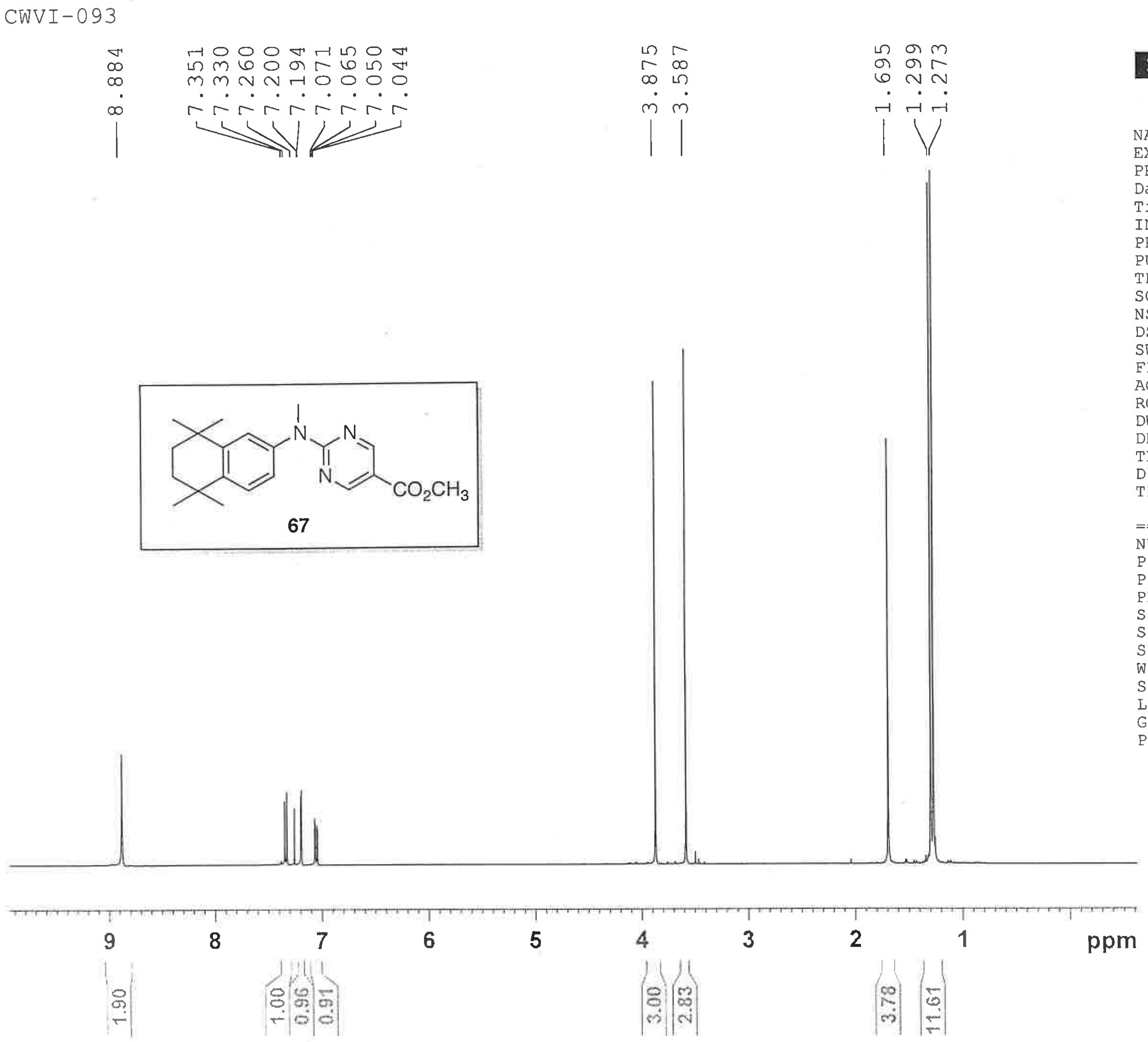

NAME	CWVI-093
EXPNO	1
PROCNO	1
Date_	20180725
Time_	14.24
INSTRUM	spect
PROBHD	5 mm PABBO BB-
PULPROG	zg30
TD	65536
SOLVENT	CDCl3
NS	16
DS	2
SWH	8223.685 Hz
FIDRES	0.125483 Hz
AQ	3.9846387 sec
RG	4
DW	60.800 usec
DE	6.50 usec
TE	294.6 K
D1	1.00000000 sec
TD0	1

======== CHANNEL f1 ========

NUC1	1H
P1	14.75 usec
PL1	0.50 dB
PL1W	12.76071072 W
SFO1	400.1324710 MHz
SI	32768
SF	400.1300099 MHz
WDW	EM
SSB	0
LB	0.30 Hz
GB	0
PC	1.00

CWVI-093

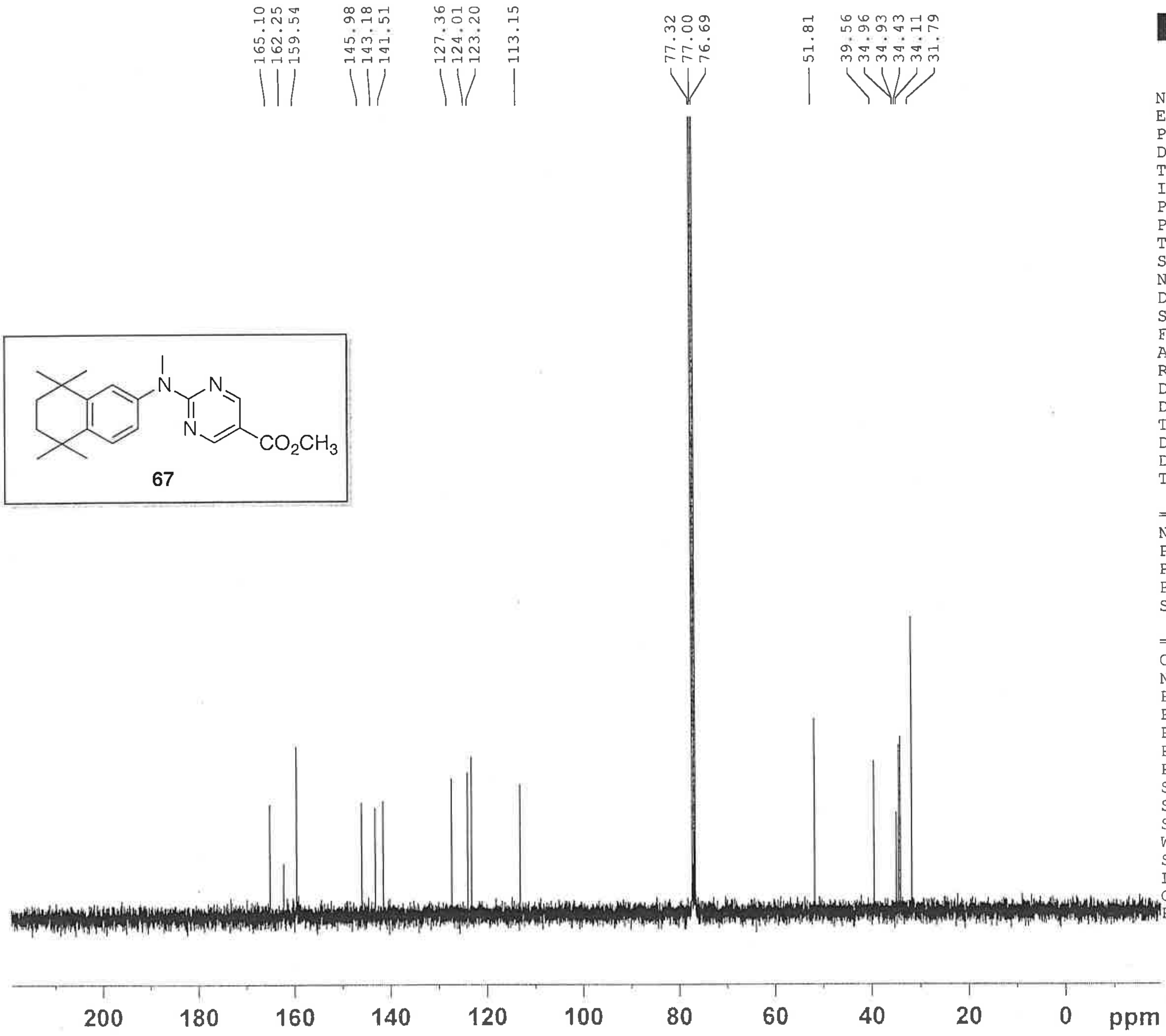

67

BRUKER

NAME	CWVI-093
EXPNO	2
PROCNO	1
Date_	20180725
Time	14.27
INSTRUM	spect
PROBHD	5 mm PABBO BB-
PULPROG	zgig30
TD	65536
SOLVENT	CDCl3
NS	100
DS	4
SWH	24038.461 Hz
FIDRES	0.366798 Hz
AQ	1.3631988 sec
RG	2050
DW	20.800 usec
DE	6.50 usec
TE	294.8 K
D1	2.00000000 sec
D11	0.03000000 sec
TD0	1
======== CHANNEL f1 ========	
NUC1	13C
P1	8.25 usec
PL1	-2.10 dB
PL1W	60.29227829 W
SFO1	100.6228298 MHz
======== CHANNEL f2 ========	
CPDPRG2	waltz16
NUC2	1H
PCPD2	90.00 usec
PL2	0.50 dB
PL12	16.21 dB
PL2W	12.76071072 W
PL12W	0.34266910 W
SFO2	400.1316005 MHz
SI	32768
SF	100.6127736 MHz
WDW	EM
SSB	0
LB	1.00 Hz
GB	0
PC	1.40

CWVI-079

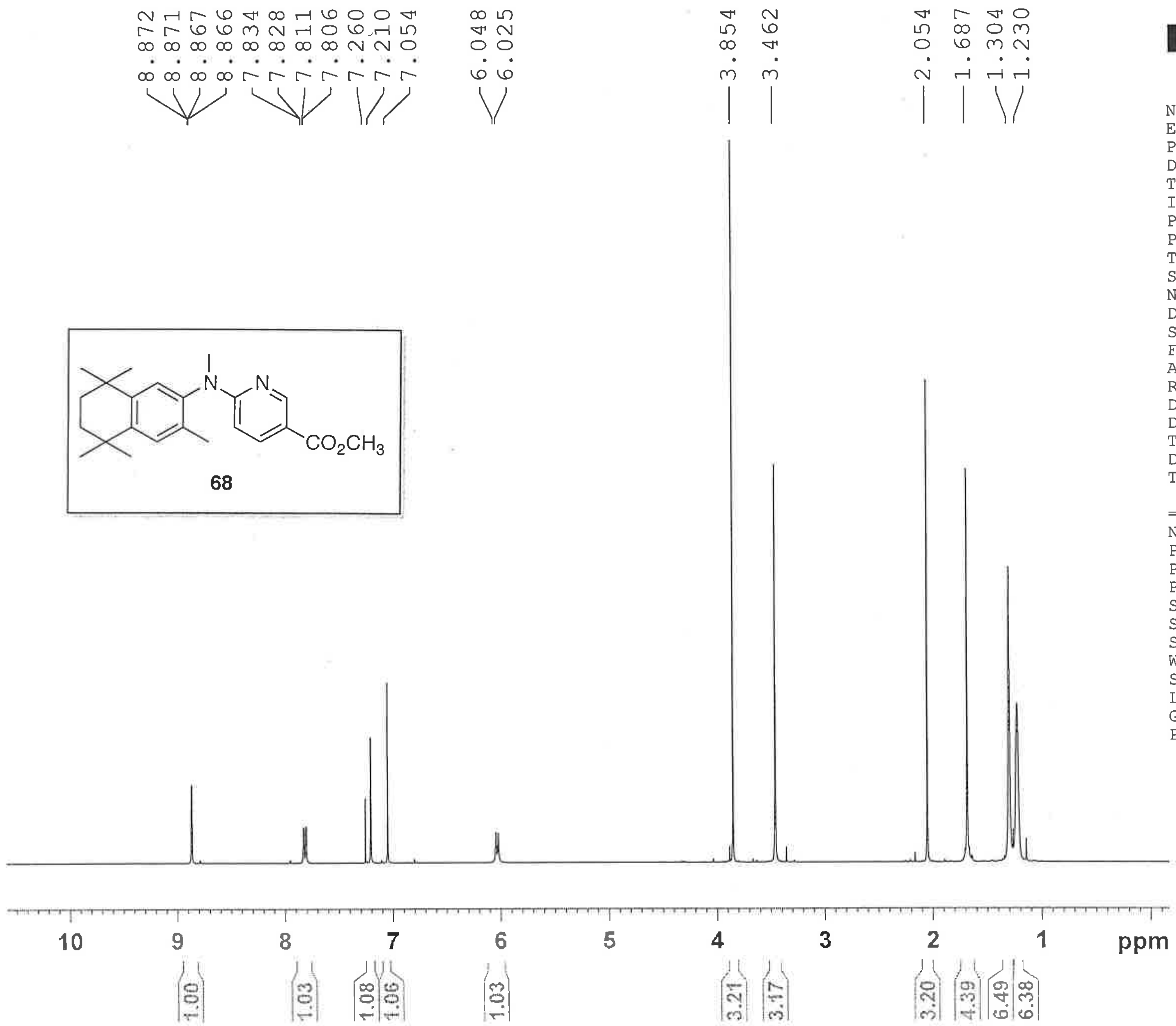

NAME	CWVI-079
EXPNO	1
PROCNO	1
Date_	20170906
Time	12.53
INSTRUM	spect
PROBHD	5 mm PABBO BB-
PULPROG	zg30
TD	65536
SOLVENT	CDCl3
NS	16
DS	2
SWH	8223.685 Hz
FIDRES	0.125483 Hz
AQ	3.9846387 sec
RG	4
DW	60.800 usec
DE	6.50 usec
TE	298.2 K
D1	1.00000000 sec
TD0	1

======== CHANNEL f1 ========

NUC1	1H
P1	14.75 usec
PL1	0.50 dB
PL1W	12.76071072 W
SFO1	400.1324710 MHz
SI	32768
SF	400.1300101 MHz
WDW	EM
SSB	0
LB	0.30 Hz
GB	0
PC	1.00

CWVI-079

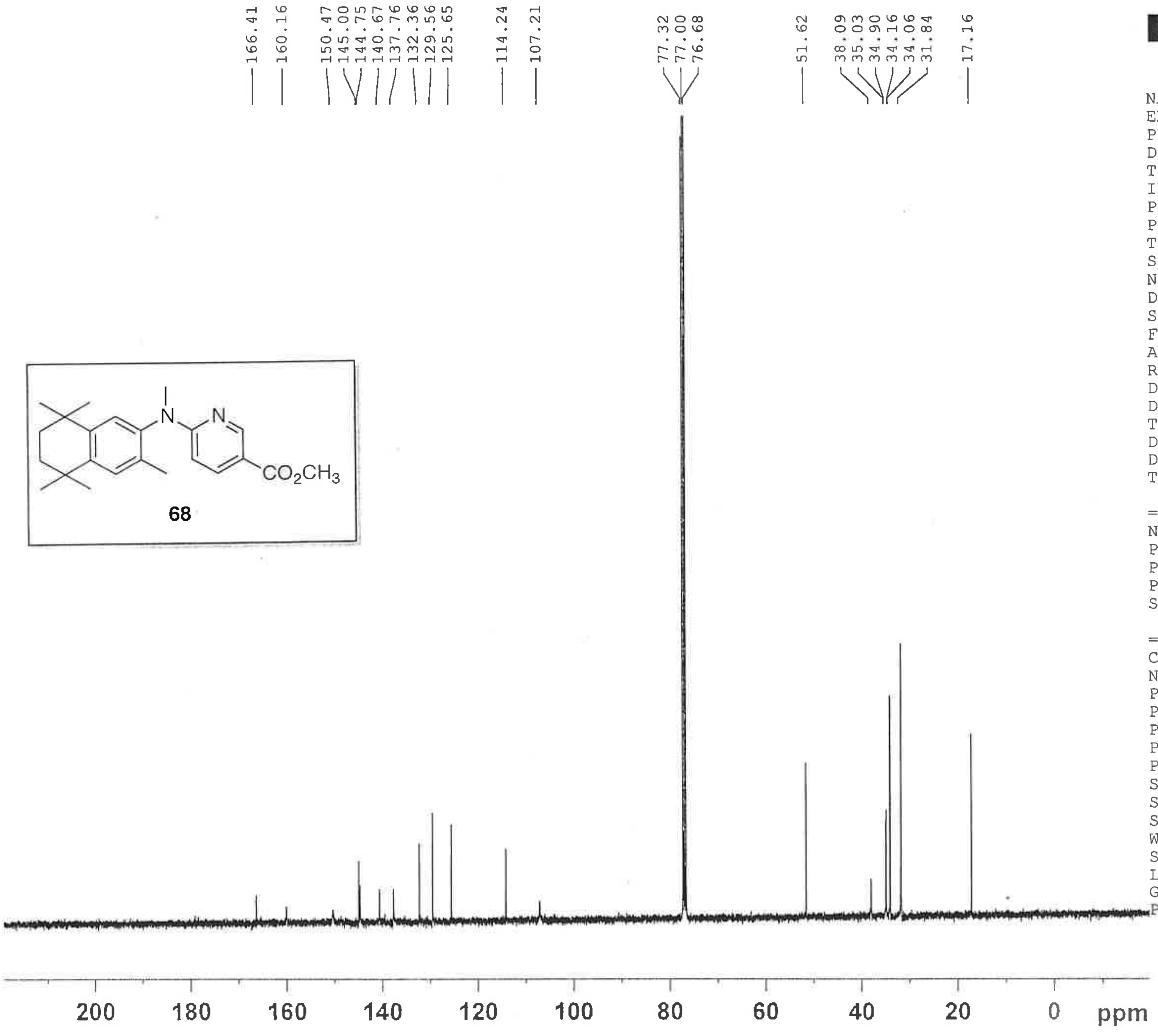

NAME	CWVI-079
EXPNO	2
PROCNO	1
Date_	20170906
Time_	13.00
INSTRUM	spect
PROBHD	5 mm PABBO BB-
PULPROG	zgig30
TD	65536
SOLVENT	CDCl3
NS	1024
DS	4
SWH	24038.461 Hz
FIDRES	0.366798 Hz
AQ	1.3631988 sec
RG	2050
DW	20.800 usec
DE	6.50 usec
TE	298.2 K
D1	2.00000000 sec
D11	0.03000000 sec
TD0	1

======== CHANNEL f1 ========

NUC1	13C
P1	8.50 usec
PL1	-2.10 dB
PL1W	60.29227829 W
SFO1	100.6228298 MHz

======== CHANNEL f2 ========

CPDPRG2	waltz16
NUC2	1H
PCPD2	90.00 usec
PL2	0.50 dB
PL12	16.21 dB
PL2W	12.76071072 W
PL12W	0.34266910 W
SFO2	400.1316005 MHz
SI	32768
SF	100.6127722 MHz
WDW	EM
SSB	0
LB	1.00 Hz
GB	0
PC	1.40

CWVI-083

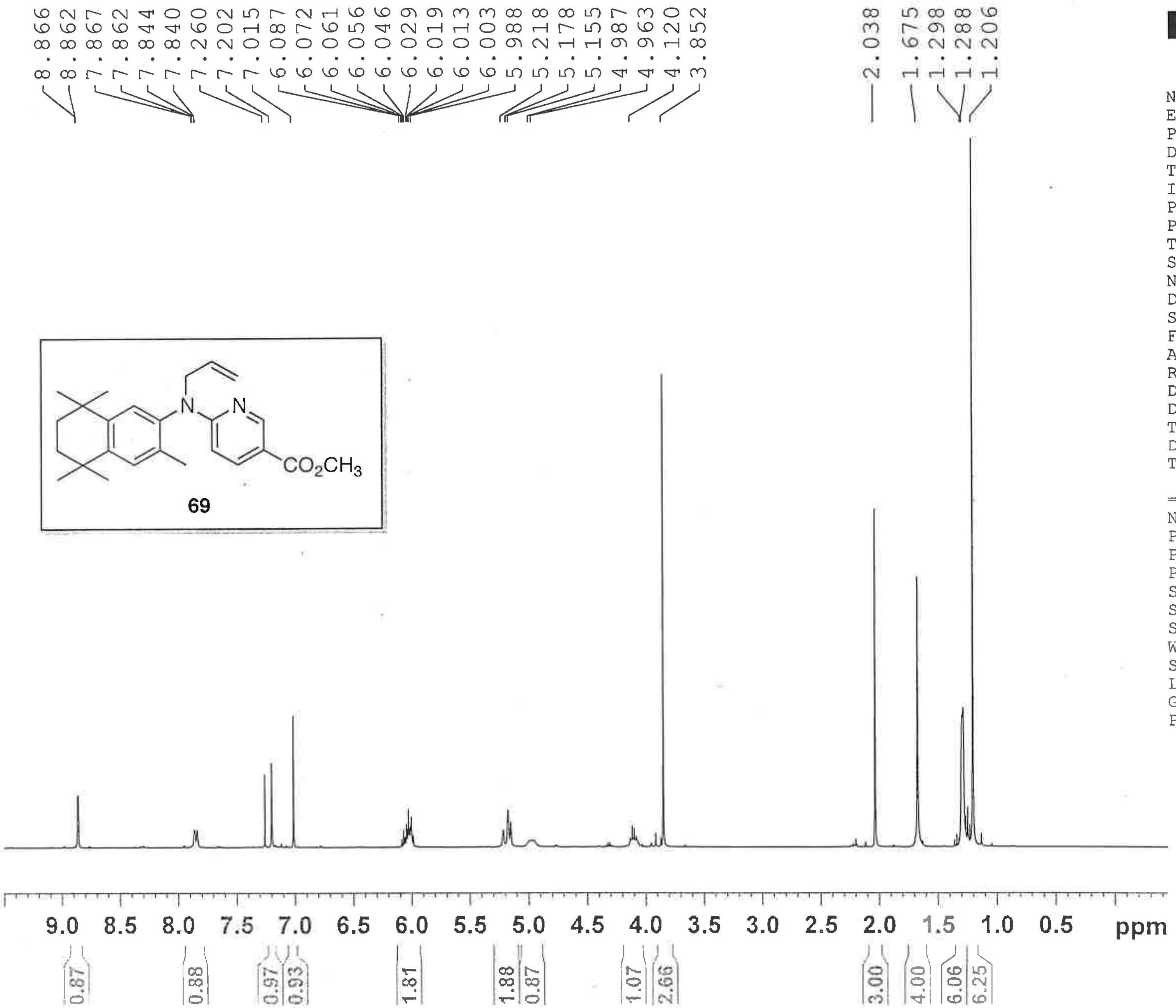

69

BRUKER

NAME	CWVI-083
EXPNO	1
PROCNO	1
Date_	20180725
Time	21.31
INSTRUM	spect
PROBHD	5 mm PABBO BB-
PULPROG	zg30
TD	65536
SOLVENT	CDCl3
NS	16
DS	2
SWH	8223.685 Hz
FIDRES	0.125483 Hz
AQ	3.9846387 sec
RG	4
DW	60.800 usec
DE	6.50 usec
TE	294.8 K
D1	1.00000000 sec
TD0	1

======== CHANNEL f1 ========

NUC1	1H
P1	14.75 usec
PL1	0.50 dB
PL1W	12.76071072 W
SFO1	400.1324710 MHz
SI	32768
SF	400.1300099 MHz
WDW	EM
SSB	0
LB	0.30 Hz
GB	0
PC	1.00

CWVI-083

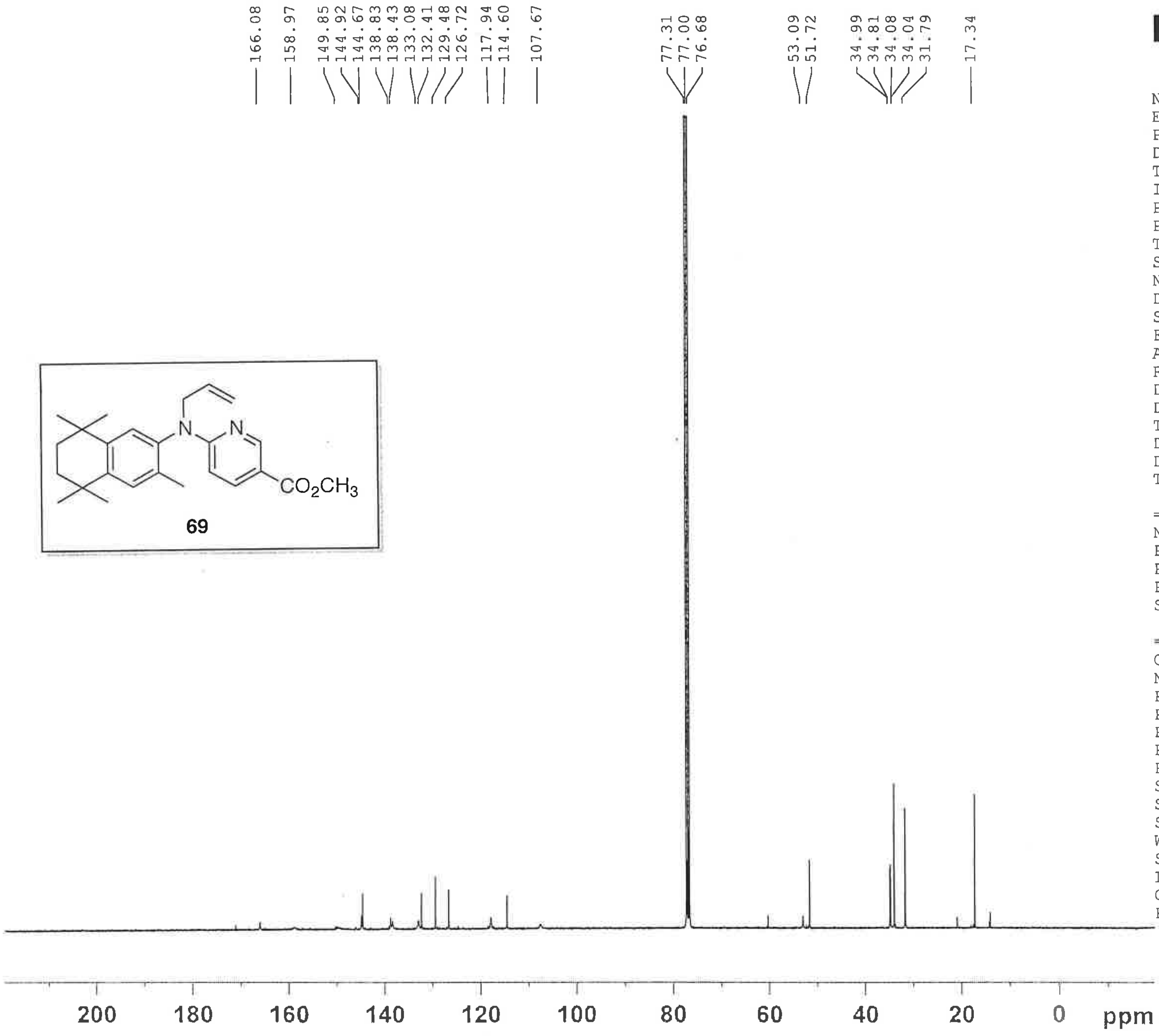

69

BRUKER

NAME	CWVI-083	
EXPNO	2	
PROCNO	1	
Date_	20180725	
Time	21.38	
INSTRUM	spect	
PROBHD	5 mm PABBO BB-	
PULPROG	zgig30	
TD	65536	
SOLVENT	CDCl3	
NS	12624	
DS	4	
SWH	24038.461	Hz
FIDRES	0.366798	Hz
AQ	1.3631988	sec
RG	2050	
DW	20.800	usec
DE	6.50	usec
TE	295.0	K
D1	2.00000000	sec
D11	0.03000000	sec
TD0	1	

======== CHANNEL f1 ========

NUC1	13C	
P1	8.25	usec
PL1	-2.10	dB
PL1W	60.29227829	W
SFO1	100.6228298	MHz

======== CHANNEL f2 ========

CPDPRG2	waltz16	
NUC2	1H	
PCPD2	90.00	usec
PL2	0.50	dB
PL12	16.21	dB
PL2W	12.76071072	W
PL12W	0.34266910	W
SFO2	400.1316005	MHz
SI	32768	
SF	100.6127736	MHz
WDW	EM	
SSB	0	
LB	1.00	Hz
GB	0	
PC	1.40	

CWVI-127

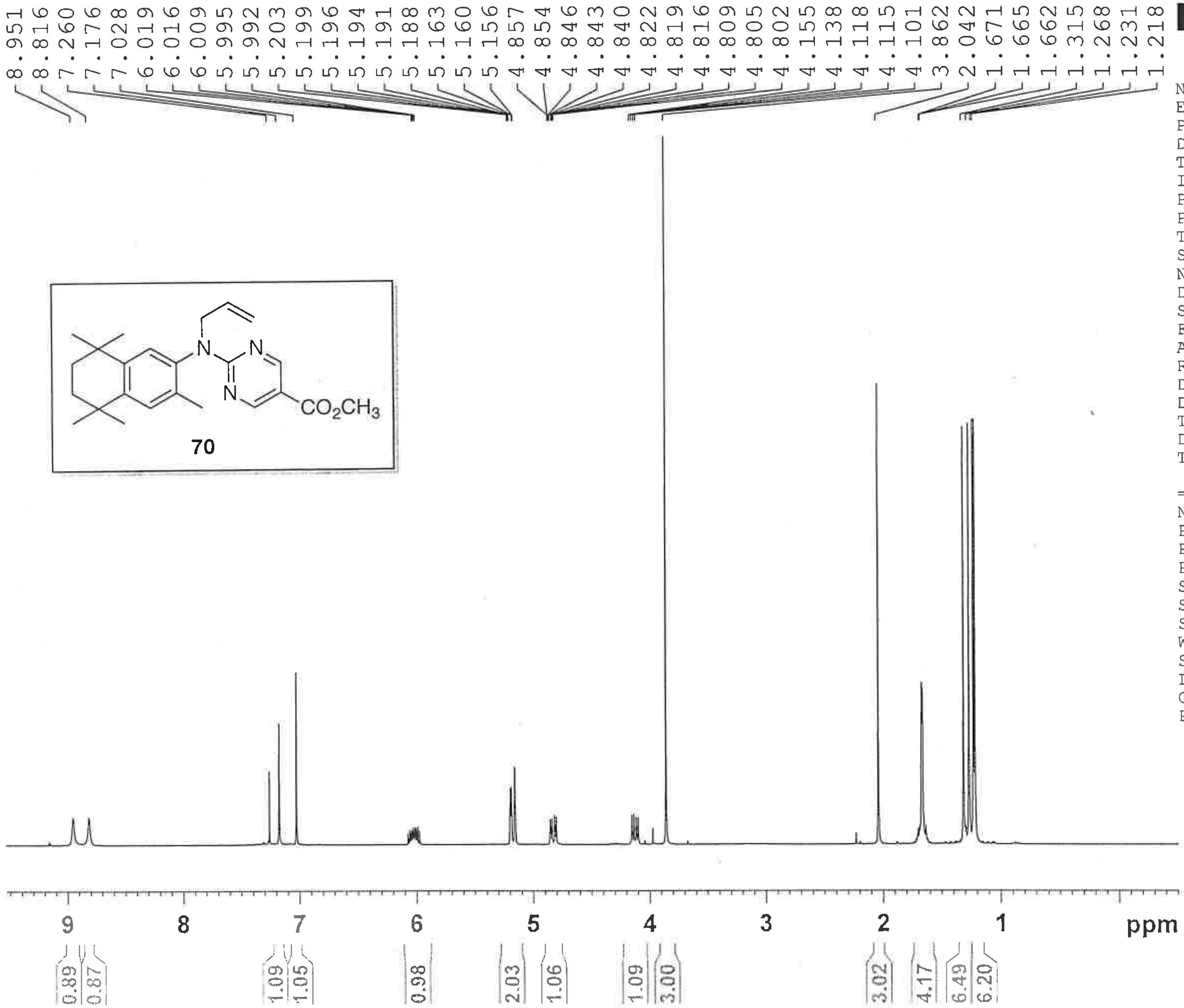

BRUKER

NAME	CWVI-127
EXPNO	1
PROCNO	1
Date_	20180725
Time	12.08
INSTRUM	spect
PROBHD	5 mm PABBO BB-
PULPROG	zg30
TD	65536
SOLVENT	CDCl3
NS	16
DS	2
SWH	8223.685 Hz
FIDRES	0.125483 Hz
AQ	3.9846387 sec
RG	4
DW	60.800 usec
DE	6.50 usec
TE	294.3 K
D1	1.00000000 sec
TD0	1

======== CHANNEL f1 ========

NUC1	1H
P1	14.75 usec
PL1	0.50 dB
PL1W	12.76071072 W
SFO1	400.1324710 MHz
SI	32768
SF	400.1300099 MHz
WDW	EM
SSB	0
LB	0.30 Hz
GB	0
PC	1.00

CWVI-127

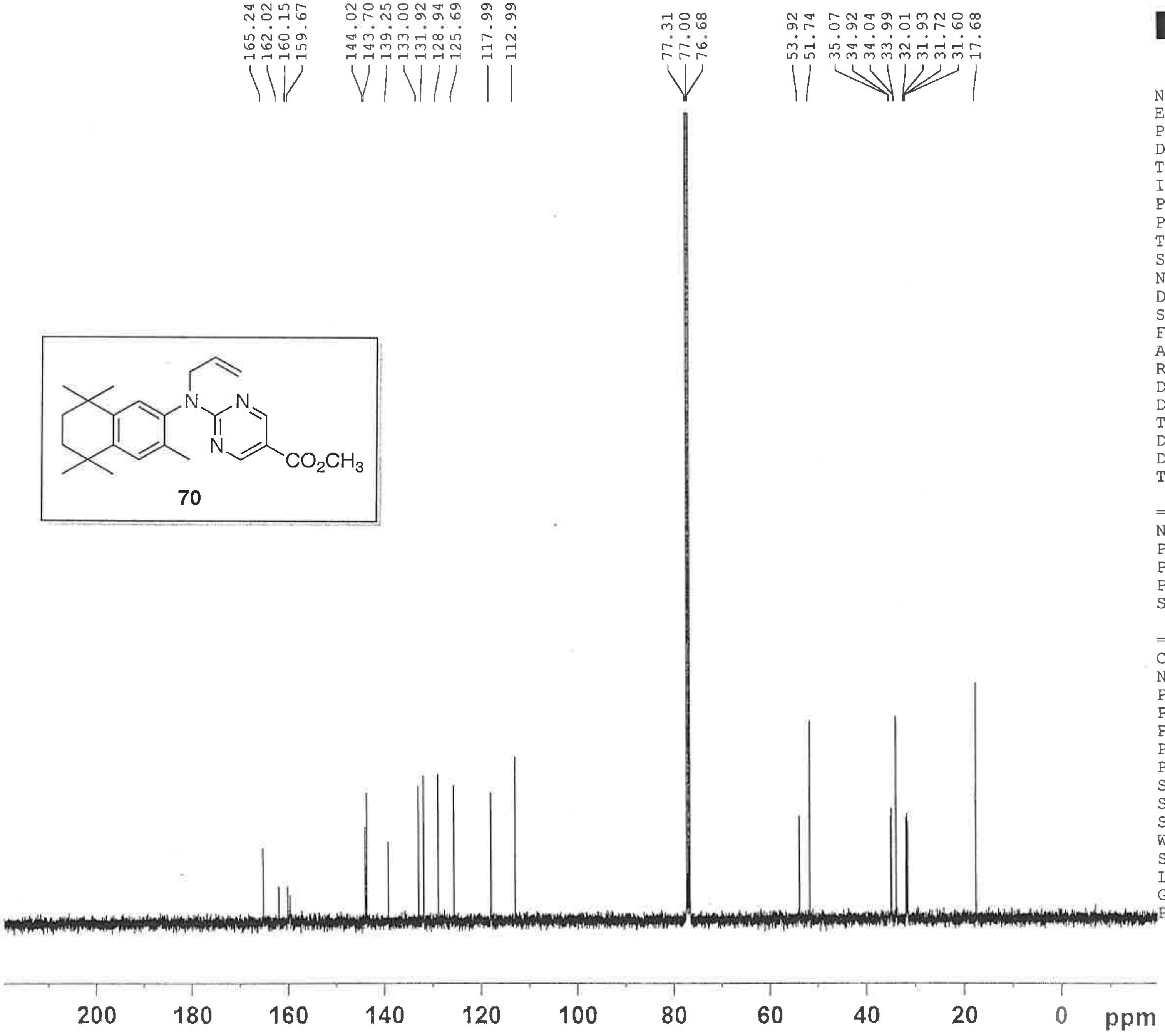

70

NAME	CWVI-127
EXPNO	2
PROCNO	1
Date_	20180725
Time_	12.22
INSTRUM	spect
PROBHD	5 mm PABBO BB-
PULPROG	zgig30
TD	65536
SOLVENT	CDCl3
NS	302
DS	4
SWH	24038.461 Hz
FIDRES	0.366798 Hz
AQ	1.3631988 sec
RG	2050
DW	20.800 usec
DE	6.50 usec
TE	294.5 K
D1	2.00000000 sec
D11	0.03000000 sec
TD0	1
======== CHANNEL f1 ========	
NUC1	13C
P1	8.25 usec
PL1	-2.10 dB
PL1W	60.29227829 W
SFO1	100.6228298 MHz
======== CHANNEL f2 ========	
CPDPRG2	waltz16
NUC2	1H
PCPD2	90.00 usec
PL2	0.50 dB
PL12	16.21 dB
PL2W	12.76071072 W
PL12W	0.34266910 W
SFO2	400.1316005 MHz
SI	32768
SF	100.6127744 MHz
WDW	EM
SSB	0
LB	1.00 Hz
GB	0
PC	1.40

CWVI-187

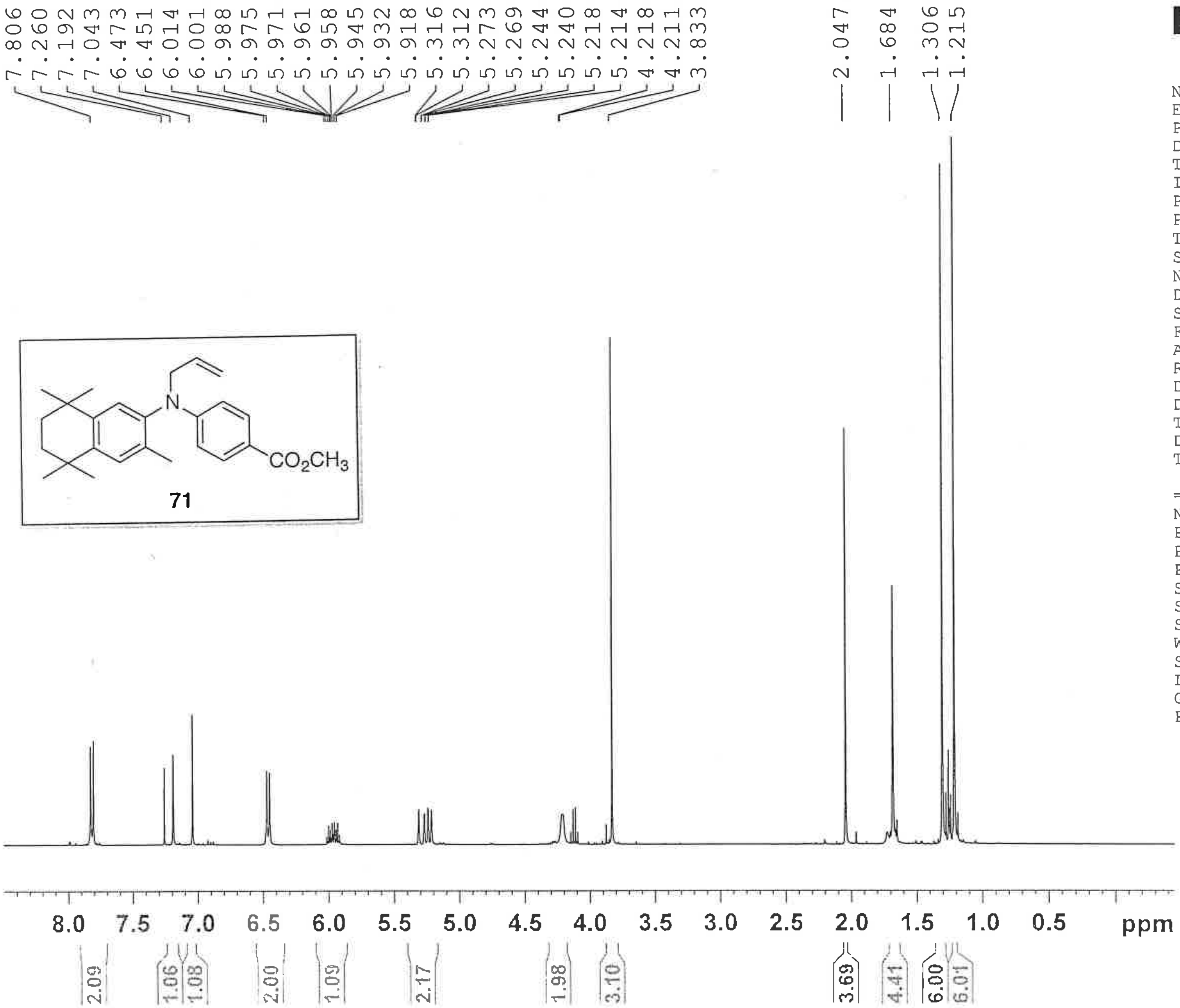

71

BRUKER

NAME	CWVI-187
EXPNO	1
PROCNO	1
Date_	20180830
Time_	13.58
INSTRUM	spect
PROBHD	5 mm PABBO BB-
PULPROG	zg30
TD	65536
SOLVENT	CDCl3
NS	16
DS	2
SWH	8223.685 Hz
FIDRES	0.125483 Hz
AQ	3.9846387 sec
RG	4
DW	60.800 usec
DE	6.50 usec
TE	294.0 K
D1	1.00000000 sec
TD0	1

======== CHANNEL f1 ========

NUC1	1H
P1	14.75 usec
PL1	0.50 dB
PL1W	12.76071072 W
SFO1	400.1324710 MHz
SI	32768
SF	400.1300099 MHz
WDW	EM
SSB	0
LB	0.30 Hz
GB	0
PC	1.00

CWVI-187

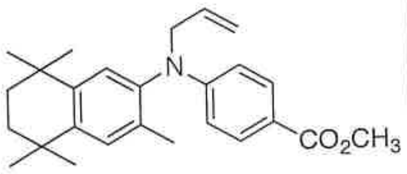

71

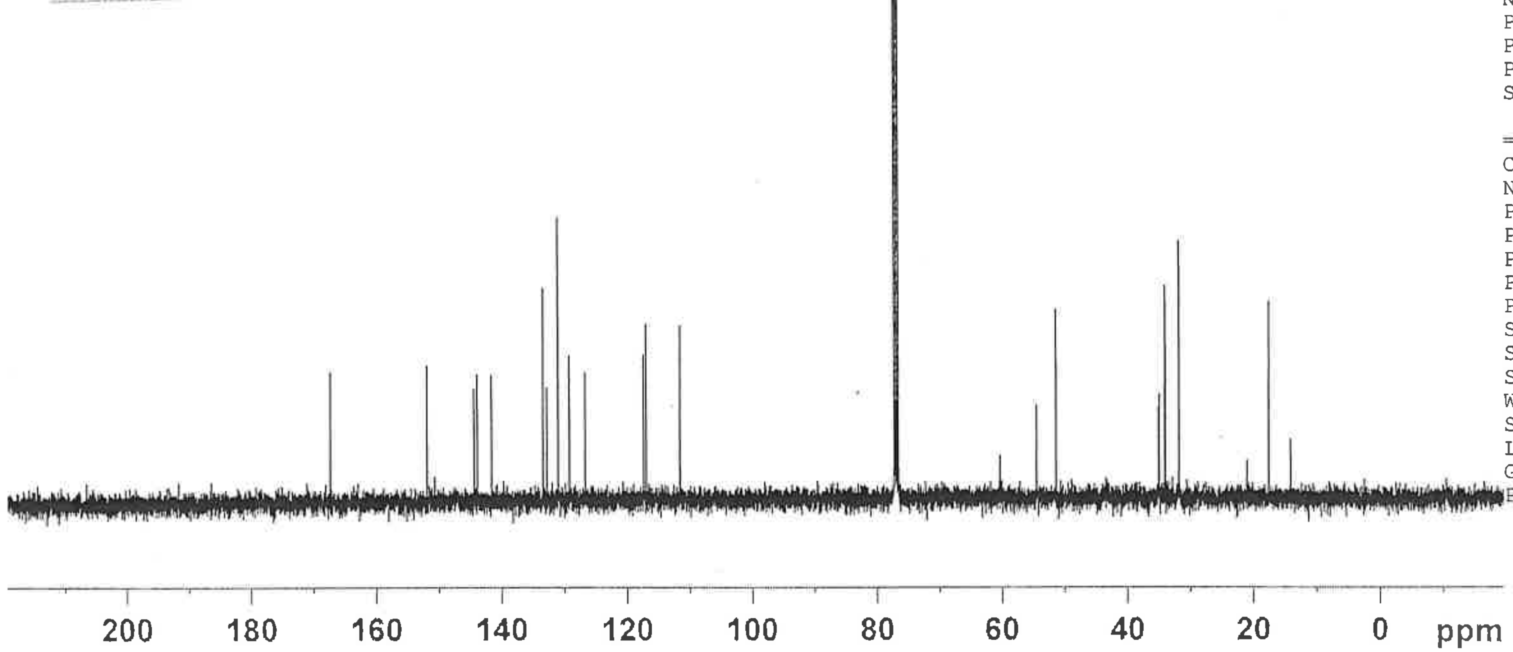

NAME	CWVI-187
EXPNO	2
PROCNO	1
Date_	20180830
Time	14.07
INSTRUM	spect
PROBHD	5 mm PABBO BB-
PULPROG	zgig30
TD	65536
SOLVENT	CDCl3
NS	185
DS	4
SWH	24038.461 Hz
FIDRES	0.366798 Hz
AQ	1.3631988 sec
RG	2050
DW	20.800 usec
DE	6.50 usec
TE	294.3 K
D1	2.00000000 sec
D11	0.03000000 sec
TD0	1
======== CHANNEL f1 ========	
NUC1	13C
P1	8.25 usec
PL1	-2.10 dB
PL1W	60.29227829 W
SFO1	100.6228298 MHz
======== CHANNEL f2 ========	
CPDPRG2	waltz16
NUC2	1H
PCPD2	90.00 usec
PL2	0.50 dB
PL12	16.21 dB
PL2W	12.76071072 W
PL12W	0.34266910 W
SFO2	400.1316005 MHz
SI	32768
SF	100.6127729 MHz
WDW	EM
SSB	0
LB	1.00 Hz
GB	0
PC	1.40

CWVI-105

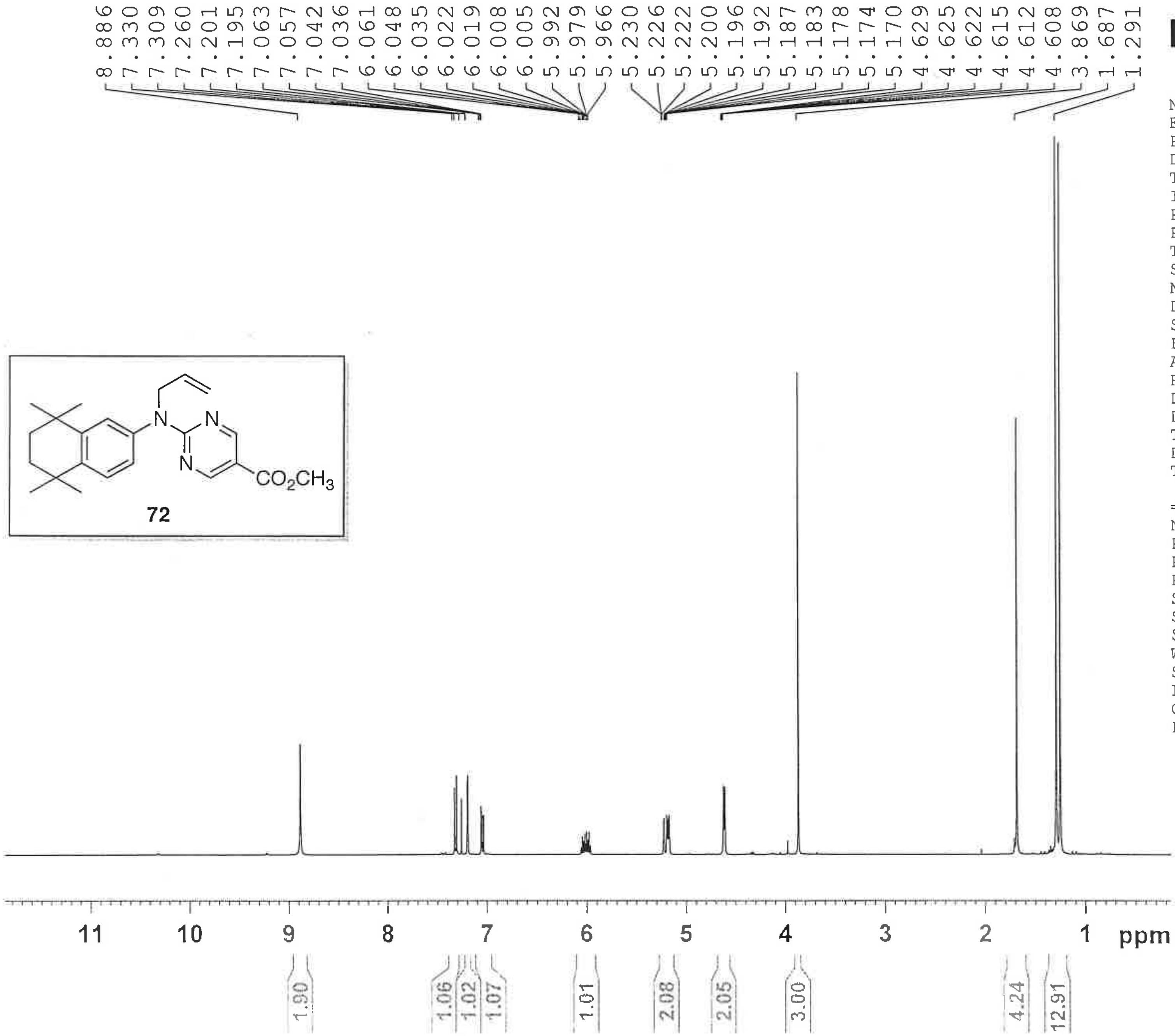

NAME	CWVI-105
EXPNO	1
PROCNO	1
Date_	20180725
Time	12.58
INSTRUM	spect
PROBHD	5 mm PABBO BB-
PULPROG	zg30
TD	65536
SOLVENT	CDCl3
NS	16
DS	2
SWH	8223.685 Hz
FIDRES	0.125483 Hz
AQ	3.9846387 sec
RG	4
DW	60.800 usec
DE	6.50 usec
TE	294.2 K
D1	1.00000000 sec
TD0	1

======== CHANNEL f1 ========

NUC1	1H
P1	14.75 usec
PL1	0.50 dB
PL1W	12.76071072 W
SFO1	400.1324710 MHz
SI	32768
SF	400.1300099 MHz
WDW	EM
SSB	0
LB	0.30 Hz
GB	0
PC	1.00

CWVI-105

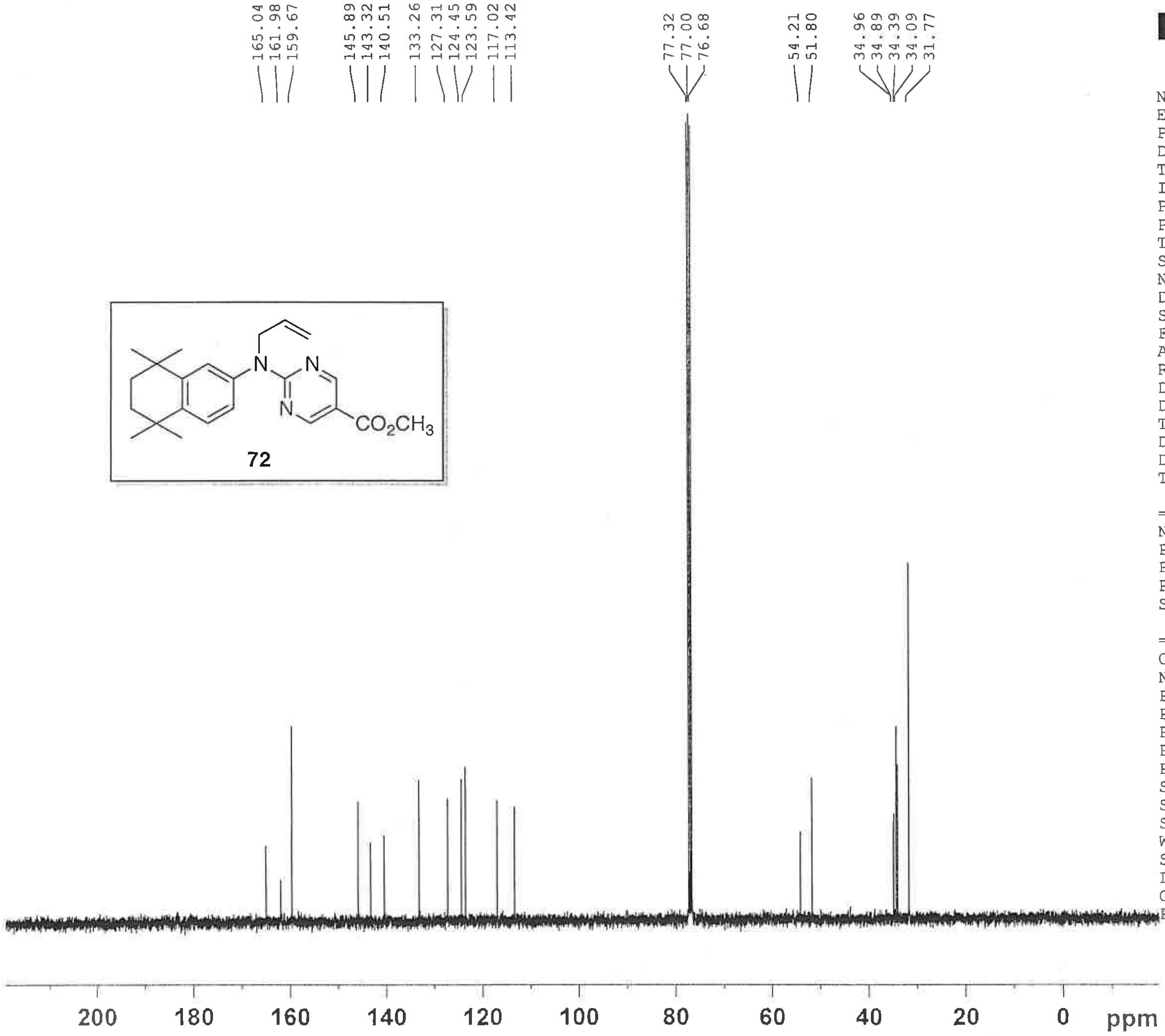

72

NAME	CWVI-105
EXPNO	2
PROCNO	1
Date_	20180725
Time_	13.14
INSTRUM	spect
PROBHD	5 mm PABBO BB-
PULPROG	zgig30
TD	65536
SOLVENT	CDCl3
NS	300
DS	4
SWH	24038.461 Hz
FIDRES	0.366798 Hz
AQ	1.3631988 sec
RG	2050
DW	20.800 usec
DE	6.50 usec
TE	294.6 K
D1	2.00000000 sec
D11	0.03000000 sec
TD0	1
======== CHANNEL f1 ========	
NUC1	13C
P1	8.25 usec
PL1	-2.10 dB
PL1W	60.29227829 W
SFO1	100.6228298 MHz
======== CHANNEL f2 ========	
CPDPRG2	waltz16
NUC2	1H
PCPD2	90.00 usec
PL2	0.50 dB
PL12	16.21 dB
PL2W	12.76071072 W
PL12W	0.34266910 W
SFO2	400.1316005 MHz
SI	32768
SF	100.6127744 MHz
WDW	EM
SSB	0
LB	1.00 Hz
GB	0
PC	1.40

CWVI-109

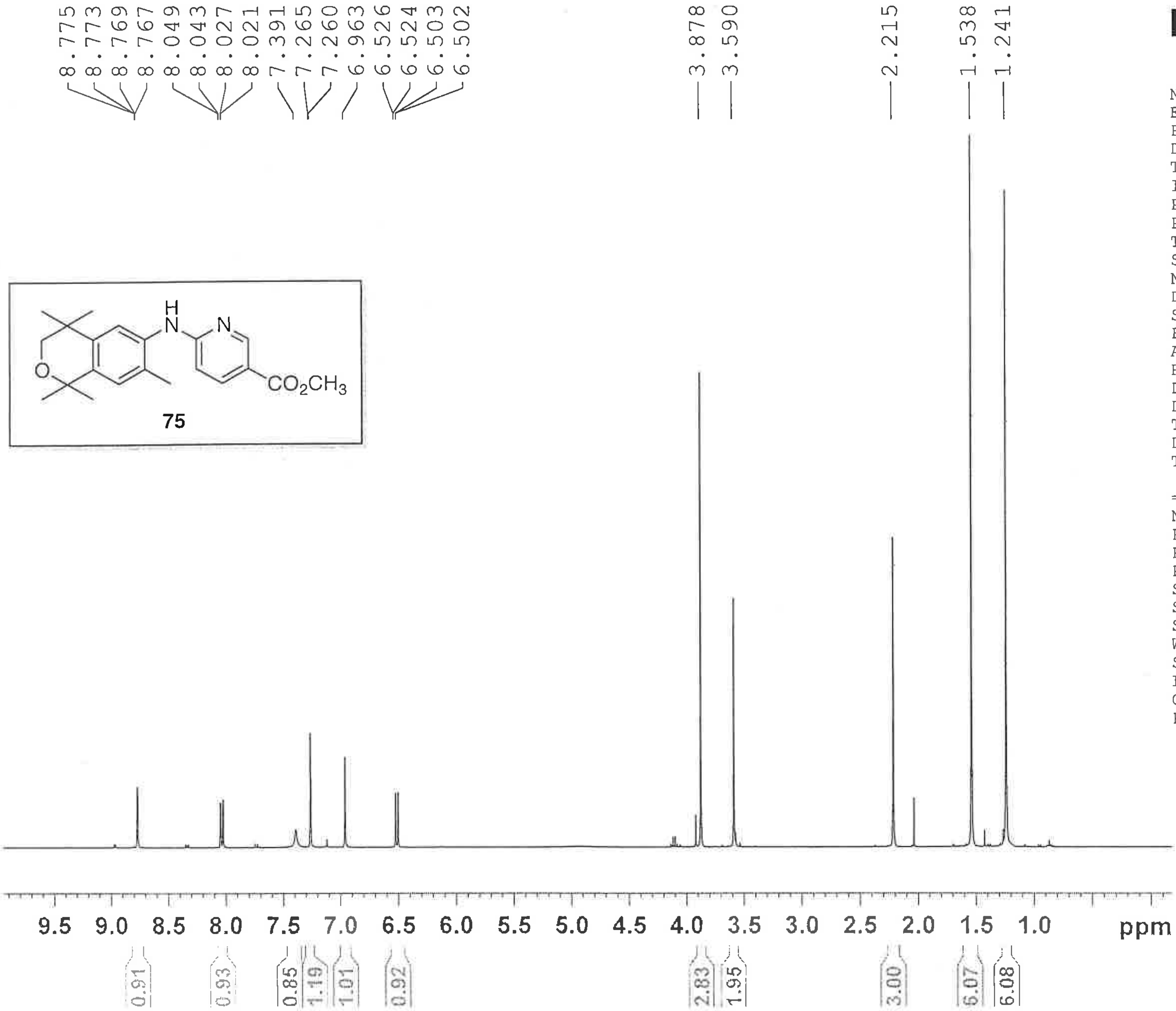

75

NAME	CWVI-109
EXPNO	1
PROCNO	1
Date_	20180329
Time	12.34
INSTRUM	spect
PROBHD	5 mm PABBO BB-
PULPROG	zg30
TD	65536
SOLVENT	CDCl3
NS	16
DS	2
SWH	8223.685 Hz
FIDRES	0.125483 Hz
AQ	3.9846387 sec
RG	4
DW	60.800 usec
DE	6.50 usec
TE	292.9 K
D1	1.00000000 sec
TD0	1

======== CHANNEL f1 ========

NUC1	1H
P1	14.75 usec
PL1	0.50 dB
PL1W	12.76071072 W
SFO1	400.1324710 MHz
SI	32768
SF	400.1300104 MHz
WDW	EM
SSB	0
LB	0.30 Hz
GB	0
PC	1.00

CWVI-109

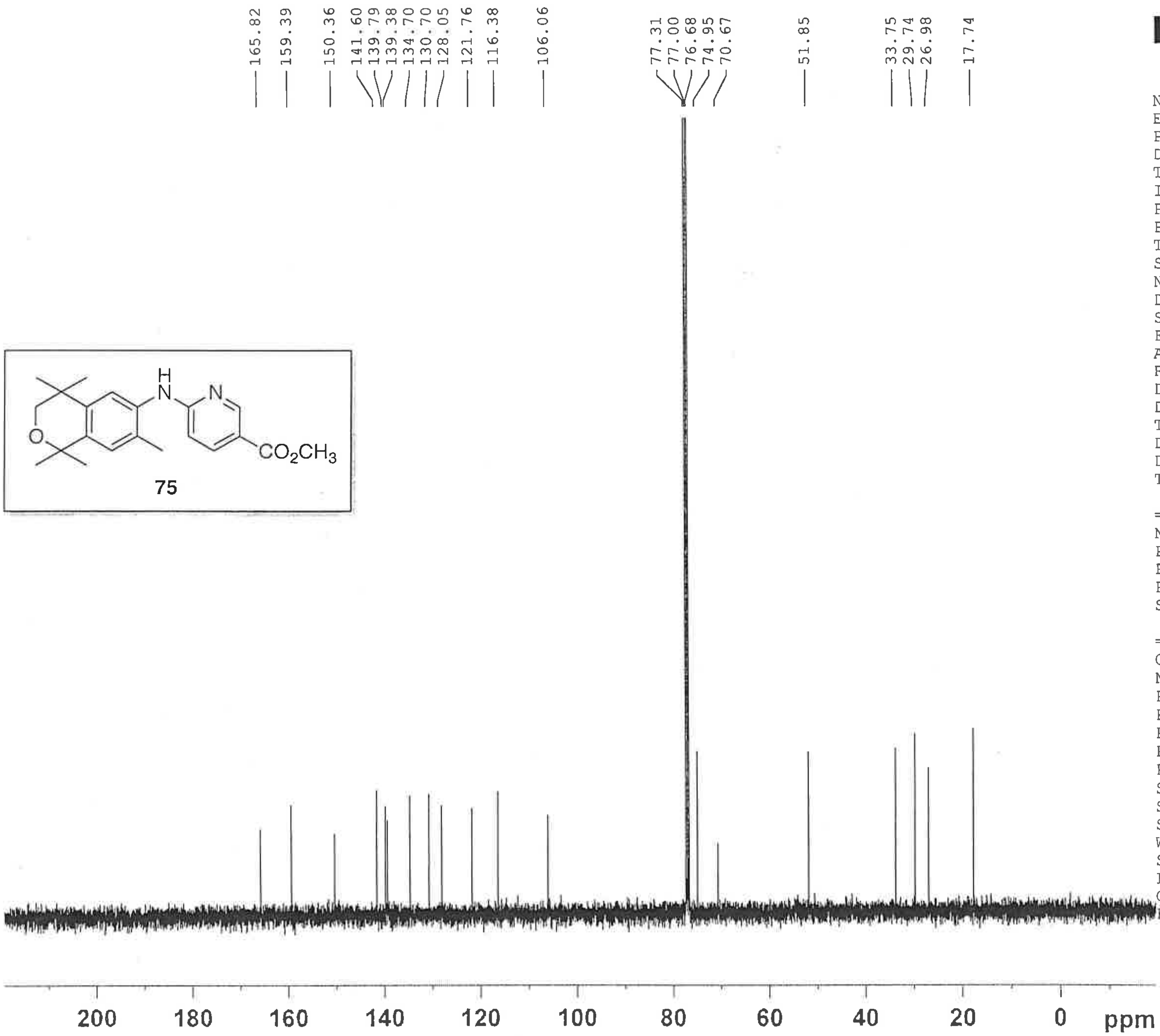

75

NAME	CWVI-109
EXPNO	2
PROCNO	1
Date_	20180329
Time	12.38
INSTRUM	spect
PROBHD	5 mm PABBO BB-
PULPROG	zgig30
TD	65536
SOLVENT	CDCl3
NS	104
DS	4
SWH	24038.461 Hz
FIDRES	0.366798 Hz
AQ	1.3631988 sec
RG	2050
DW	20.800 usec
DE	6.50 usec
TE	293.1 K
D1	2.00000000 sec
D11	0.03000000 sec
TD0	1

======== CHANNEL f1 ========

NUC1	13C
P1	8.50 usec
PL1	-2.10 dB
PL1W	60.29227829 W
SFO1	100.6228298 MHz

======== CHANNEL f2 ========

CPDPRG2	waltz16
NUC2	1H
PCPD2	90.00 usec
PL2	0.50 dB
PL12	16.21 dB
PL2W	12.76071072 W
PL12W	0.34266910 W
SFO2	400.1316005 MHz
SI	32768
SF	100.6127751 MHz
WDW	EM
SSB	0
LB	1.00 Hz
GB	0
PC	1.40

CWVI-113

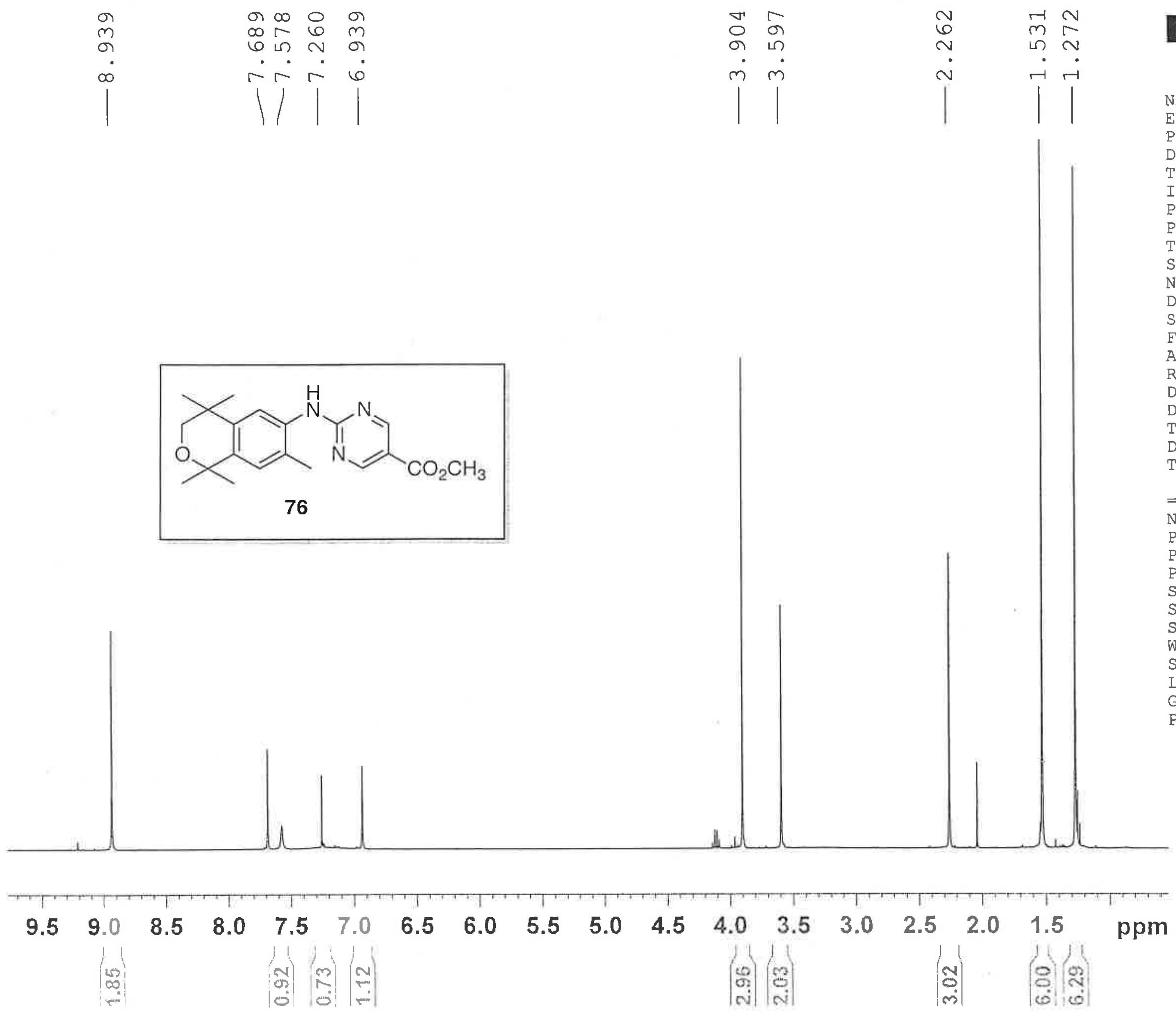

NAME	CWVI-113
EXPNO	1
PROCNO	1
Date_	20180420
Time_	9.26
INSTRUM	spect
PROBHD	5 mm PABBO BB-
PULPROG	zg30
TD	65536
SOLVENT	CDCl3
NS	16
DS	2
SWH	8223.685 Hz
FIDRES	0.125483 Hz
AQ	3.9846387 sec
RG	4
DW	60.800 usec
DE	6.50 usec
TE	292.7 K
D1	1.00000000 sec
TD0	1

======== CHANNEL f1 ========

NUC1	1H
P1	14.75 usec
PL1	0.50 dB
PL1W	12.76071072 W
SFO1	400.1324710 MHz
SI	32768
SF	400.1300096 MHz
WDW	EM
SSB	0
LB	0.30 Hz
GB	0
PC	1.00

CWVI-113

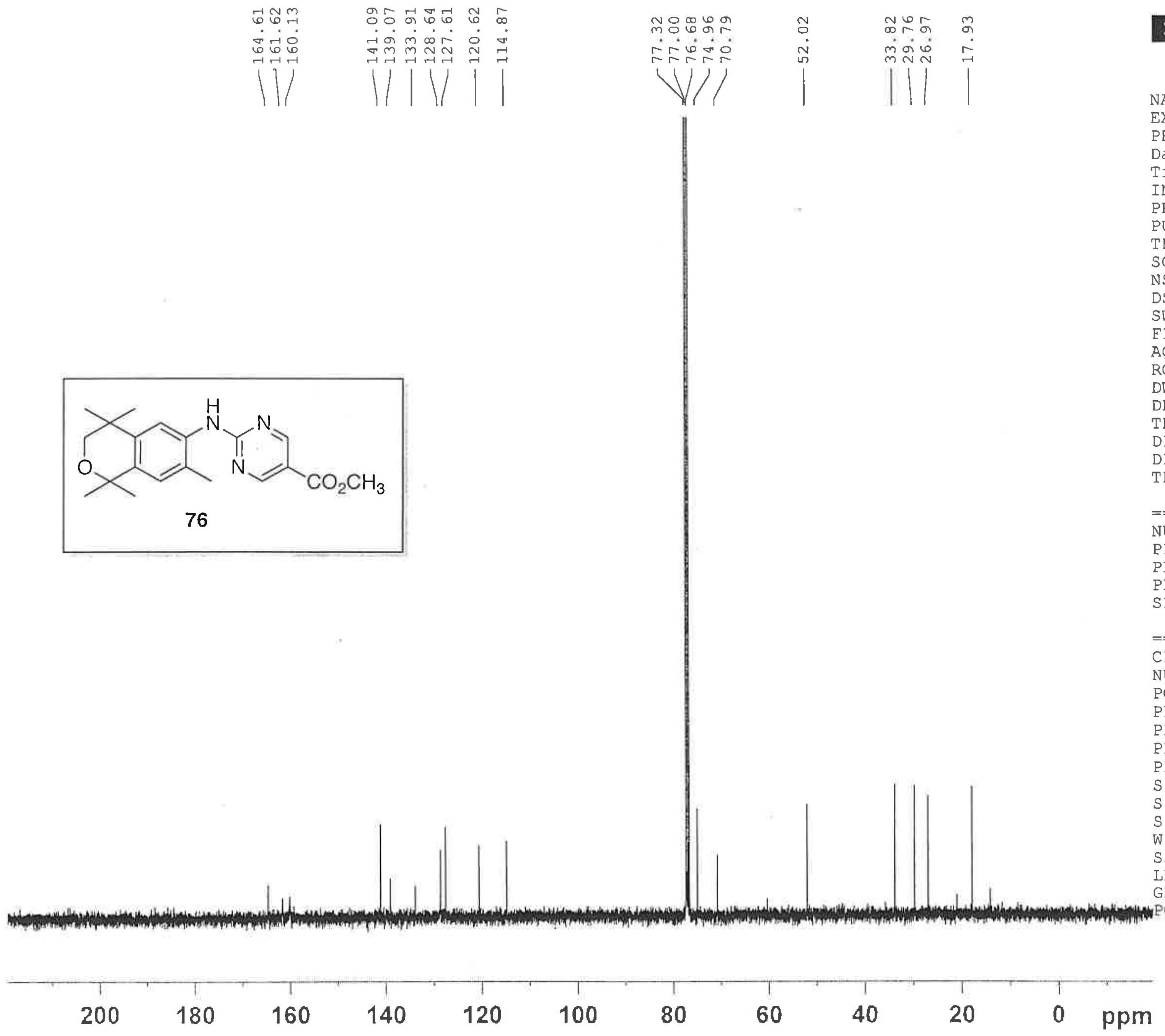

76

NAME	CWVI-113
EXPNO	2
PROCNO	1
Date_	20180420
Time	9.36
INSTRUM	spect
PROBHD	5 mm PABBO BB-
PULPROG	zgig30
TD	65536
SOLVENT	CDCl3
NS	250
DS	4
SWH	24038.461 Hz
FIDRES	0.366798 Hz
AQ	1.3631988 sec
RG	2050
DW	20.800 usec
DE	6.50 usec
TE	293.1 K
D1	2.00000000 sec
D11	0.03000000 sec
TD0	1
======== CHANNEL f1 ========	
NUC1	13C
P1	8.25 usec
PL1	-2.10 dB
PL1W	60.29227829 W
SFO1	100.6228298 MHz
======== CHANNEL f2 ========	
CPDPRG2	waltz16
NUC2	1H
PCPD2	90.00 usec
PL2	0.50 dB
PL12	16.21 dB
PL2W	12.76071072 W
PL12W	0.34266910 W
SFO2	400.1316005 MHz
SI	32768
SF	100.6127744 MHz
WDW	EM
SSB	0
LB	1.00 Hz
GB	0
PC	1.40

CWVI-117

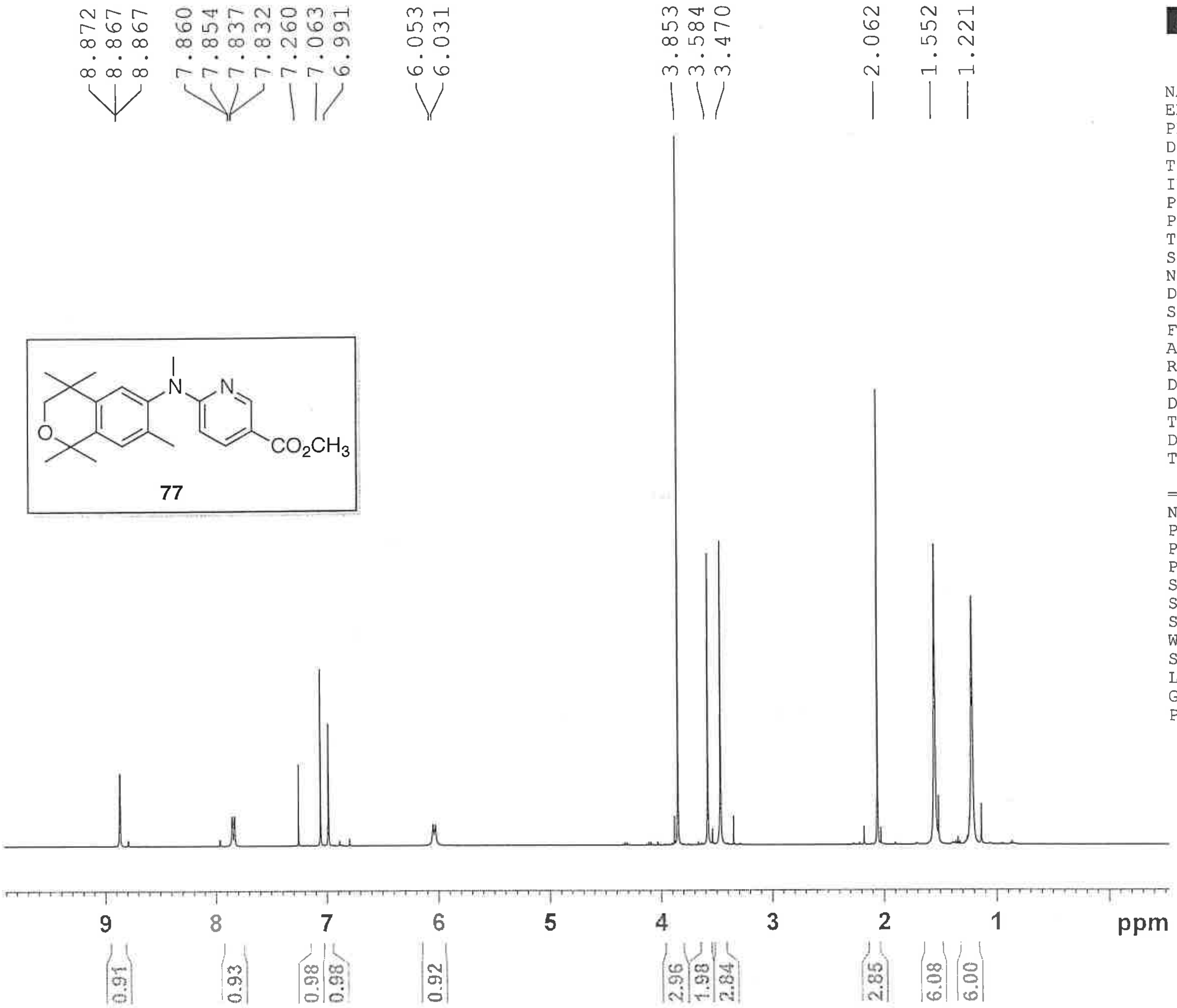

77

NAME	CWVI-117
EXPNO	1
PROCNO	1
Date_	20180725
Time_	11.41
INSTRUM	spect
PROBHD	5 mm PABBO BB-
PULPROG	zg30
TD	65536
SOLVENT	CDCl3
NS	16
DS	2
SWH	8223.685 Hz
FIDRES	0.125483 Hz
AQ	3.9846387 sec
RG	4
DW	60.800 usec
DE	6.50 usec
TE	294.2 K
D1	1.00000000 sec
TD0	1

======== CHANNEL f1 ========

NUC1	1H
P1	14.75 usec
PL1	0.50 dB
PL1W	12.76071072 W
SFO1	400.1324710 MHz
SI	32768
SF	400.1300099 MHz
WDW	EM
SSB	0
LB	0.30 Hz
GB	0
PC	1.00

CWVI-117

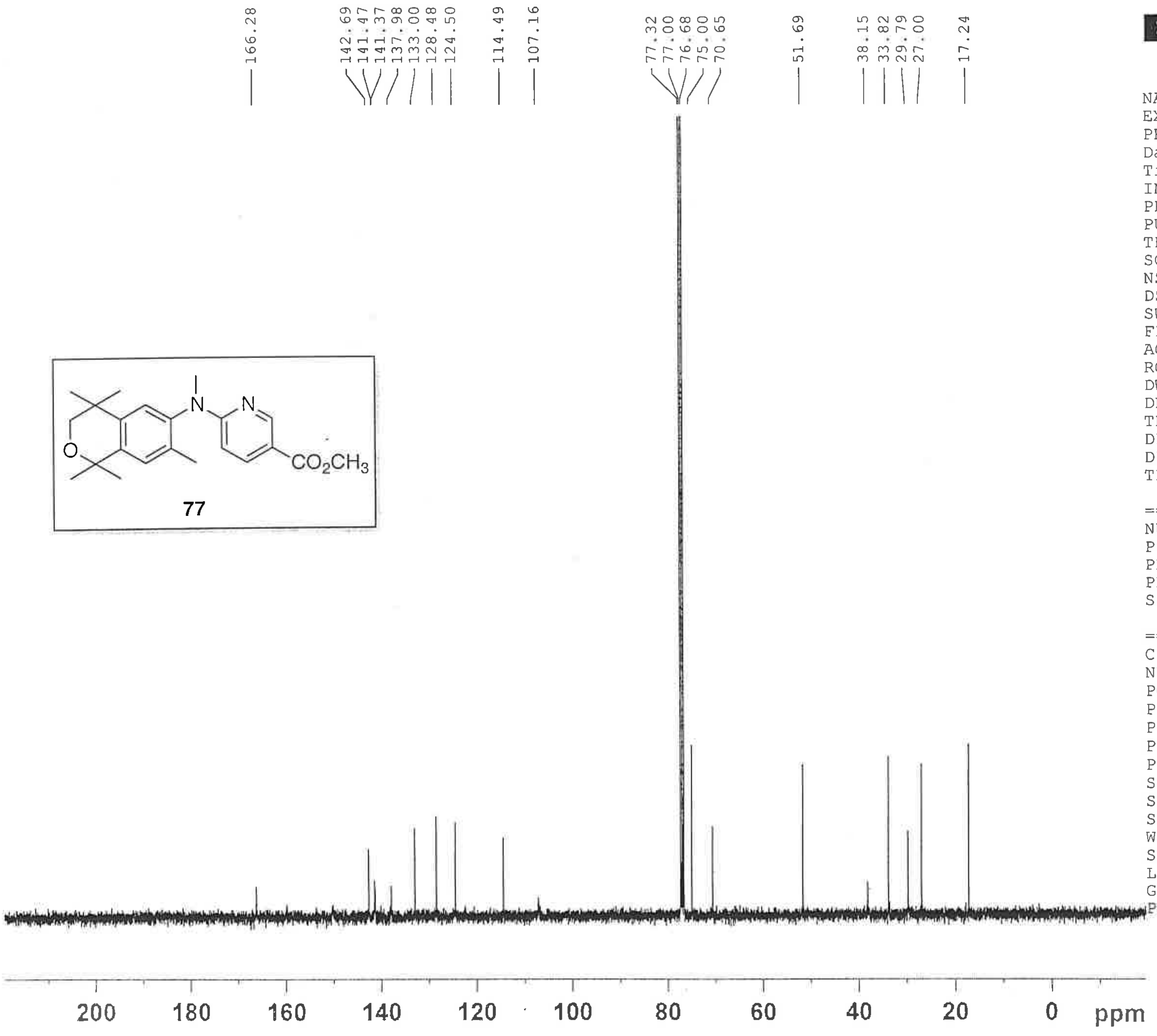

BRUKER

NAME	CWVI-117
EXPNO	2
PROCNO	1
Date_	20180725
Time_	11.46
INSTRUM	spect
PROBHD	5 mm PABBO BB-
PULPROG	zgig30
TD	65536
SOLVENT	CDCl3
NS	335
DS	4
SWH	24038.461 Hz
FIDRES	0.366798 Hz
AQ	1.3631988 sec
RG	2050
DW	20.800 usec
DE	6.50 usec
TE	294.5 K
D1	2.00000000 sec
D11	0.03000000 sec
TD0	1

======== CHANNEL f1 ========

NUC1	13C
P1	8.25 usec
PL1	-2.10 dB
PL1W	60.29227829 W
SFO1	100.6228298 MHz

======== CHANNEL f2 ========

CPDPRG2	waltz16
NUC2	1H
PCPD2	90.00 usec
PL2	0.50 dB
PL12	16.21 dB
PL2W	12.76071072 W
PL12W	0.34266910 W
SFO2	400.1316005 MHz
SI	32768
SF	100.6127736 MHz
WDW	EM
SSB	0
LB	1.00 Hz
GB	0
PC	1.40

CWVI-123

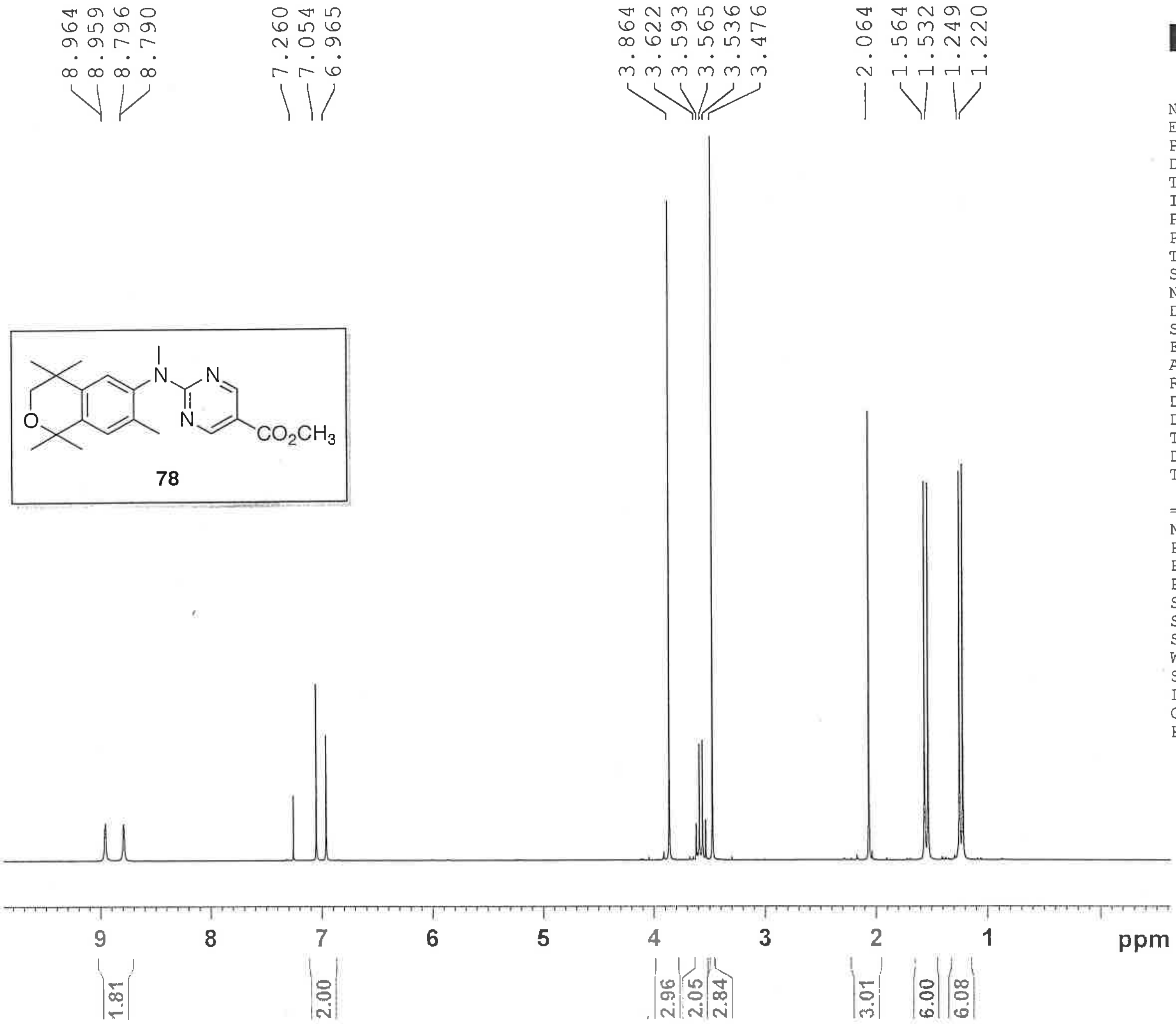

78

NAME	CWVI-123
EXPNO	2
PROCNO	1
Date_	20180725
Time	12.32
INSTRUM	spect
PROBHD	5 mm PABBO BB-
PULPROG	zg30
TD	65536
SOLVENT	CDCl3
NS	16
DS	2
SWH	8223.685 Hz
FIDRES	0.125483 Hz
AQ	3.9846387 sec
RG	4
DW	60.800 usec
DE	6.50 usec
TE	294.2 K
D1	1.00000000 sec
TD0	1

======== CHANNEL f1 ========

NUC1	1H
P1	14.75 usec
PL1	0.50 dB
PL1W	12.76071072 W
SFO1	400.1324710 MHz
SI	32768
SF	400.1300099 MHz
WDW	EM
SSB	0
LB	0.30 Hz
GB	0
PC	1.00

CWVI-123

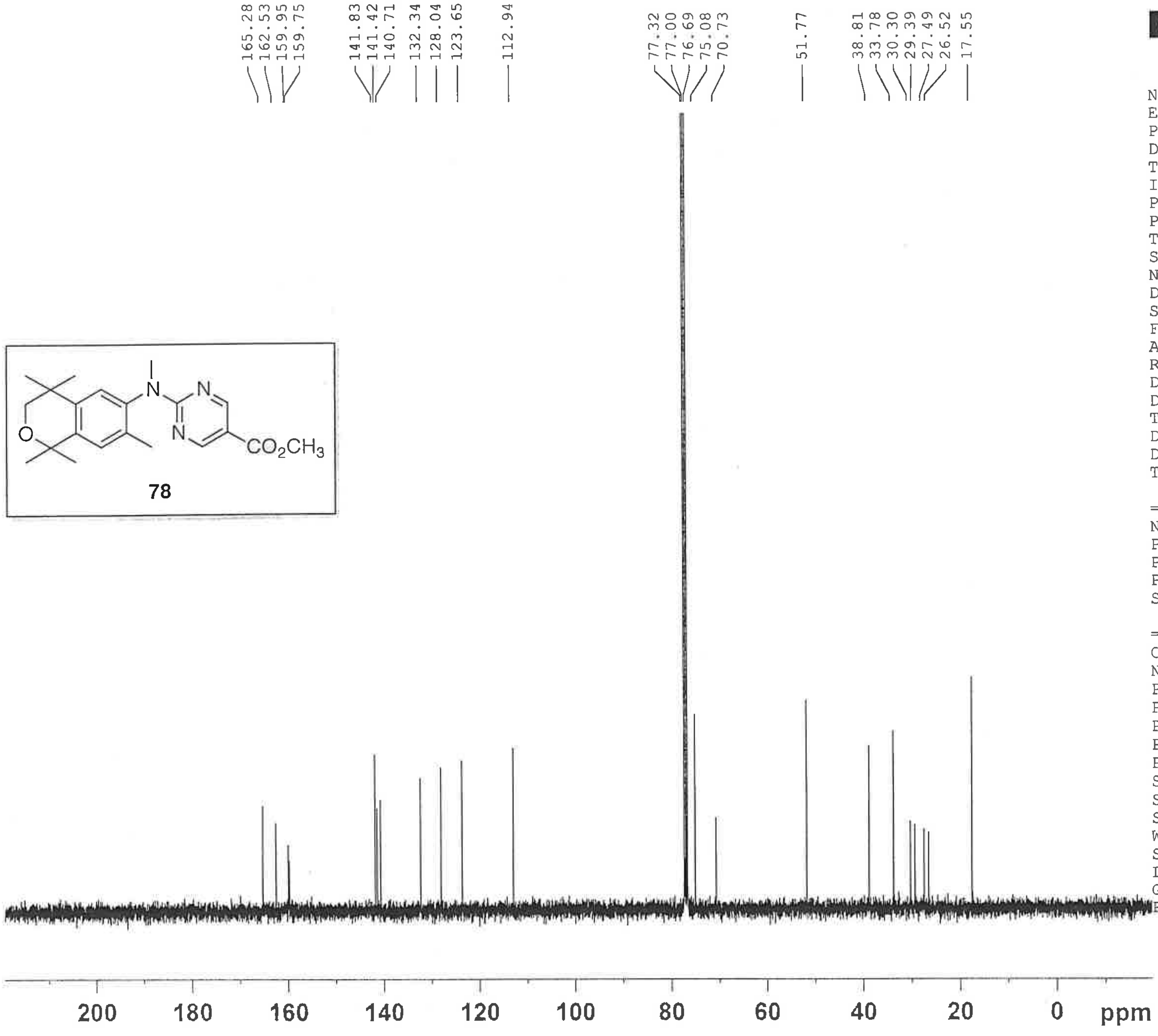

78

NAME	CWVI-123
EXPNO	1
PROCNO	1
Date_	20180725
Time_	12.45
INSTRUM	spect
PROBHD	5 mm PABBO BB-
PULPROG	zgig30
TD	65536
SOLVENT	CDCl3
NS	155
DS	4
SWH	24038.461 Hz
FIDRES	0.366798 Hz
AQ	1.3631988 sec
RG	2050
DW	20.800 usec
DE	6.50 usec
TE	294.5 K
D1	2.00000000 sec
D11	0.03000000 sec
TD0	1
======== CHANNEL f1 ========	
NUC1	13C
P1	8.25 usec
PL1	-2.10 dB
PL1W	60.29227829 W
SFO1	100.6228298 MHz
======== CHANNEL f2 ========	
CPDPRG2	waltz16
NUC2	1H
PCPD2	90.00 usec
PL2	0.50 dB
PL12	16.21 dB
PL2W	12.76071072 W
PL12W	0.34266910 W
SFO2	400.1316005 MHz
SI	32768
SF	100.6127736 MHz
WDW	EM
SSB	0
LB	1.00 Hz
GB	0
PC	1.40

CWVI-115

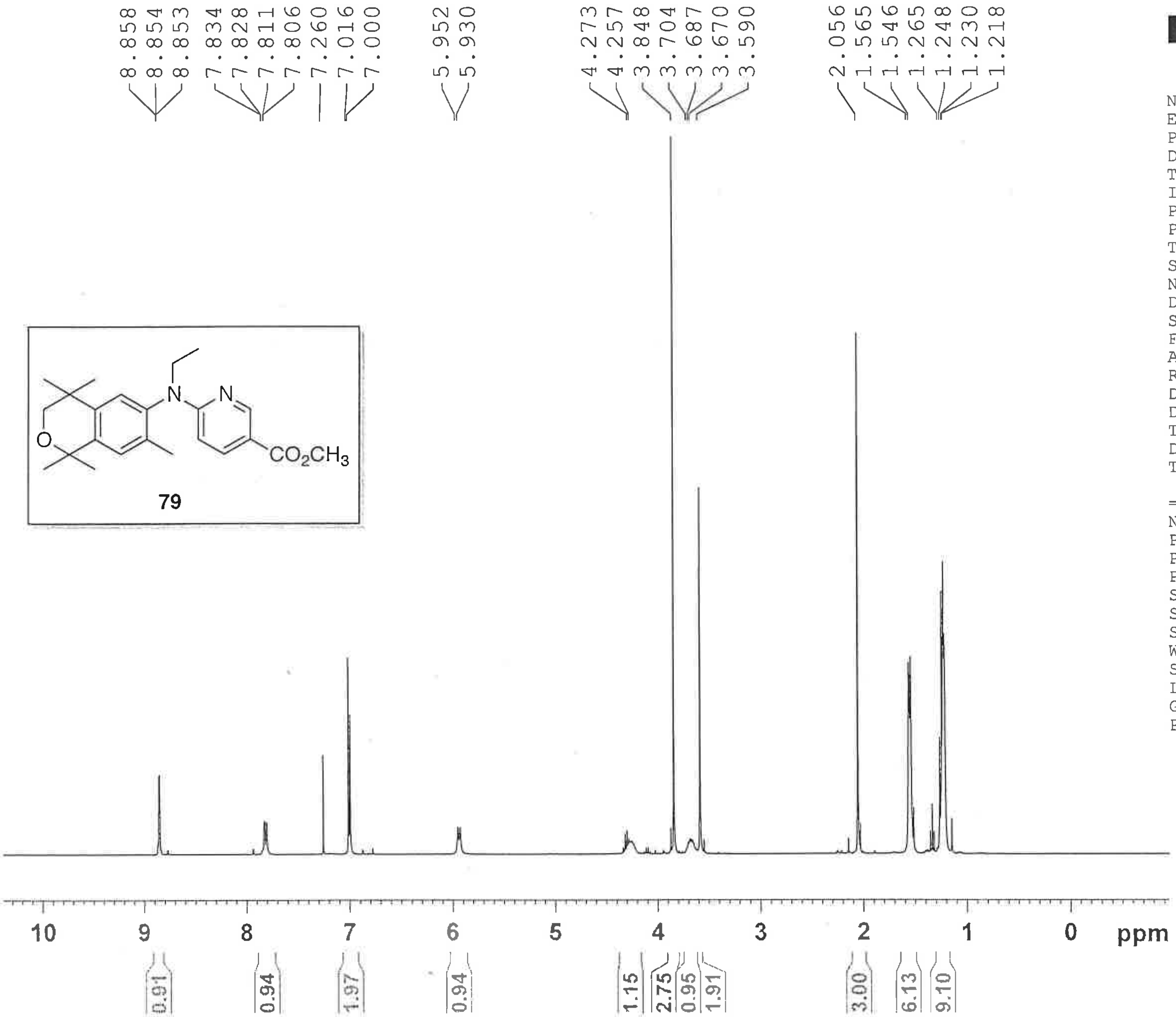

79

NAME	CWVI-115
EXPNO	1
PROCNO	1
Date_	20180725
Time	14.02
INSTRUM	spect
PROBHD	5 mm PABBO BB-
PULPROG	zg30
TD	65536
SOLVENT	CDCl3
NS	16
DS	2
SWH	8223.685 Hz
FIDRES	0.125483 Hz
AQ	3.9846387 sec
RG	4
DW	60.800 usec
DE	6.50 usec
TE	294.5 K
D1	1.00000000 sec
TD0	1
======== CHANNEL f1 ========	
NUC1	1H
P1	14.75 usec
PL1	0.50 dB
PL1W	12.76071072 W
SFO1	400.1324710 MHz
SI	32768
SF	400.1300099 MHz
WDW	EM
SSB	0
LB	0.30 Hz
GB	0
PC	1.00

CWVI-115

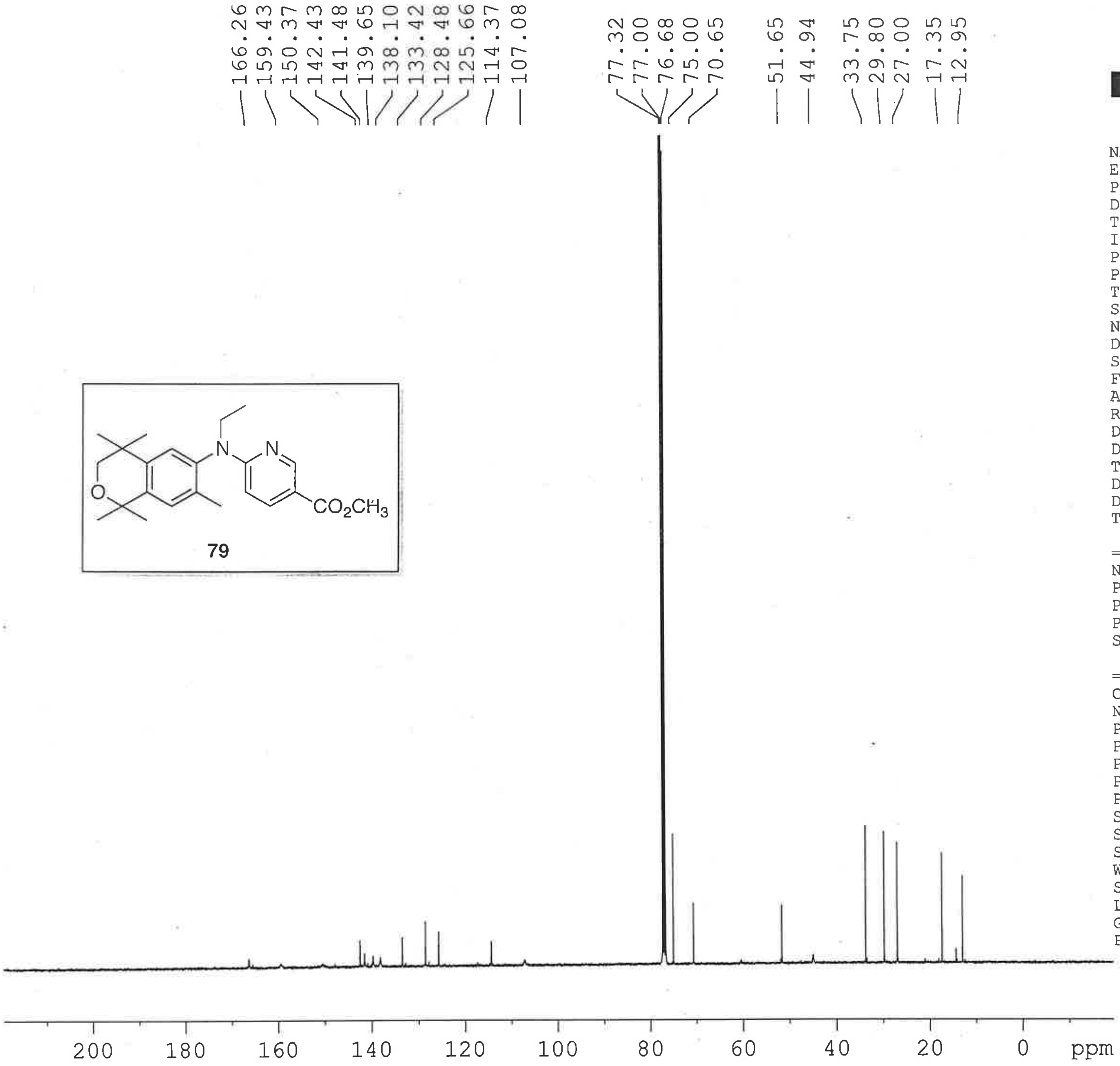

79

BRUKER

NAME	CWVI-115
EXPNO	3
PROCNO	1
Date_	20180725
Time	20.42
INSTRUM	spect
PROBHD	5 mm PABBO BB-
PULPROG	zgig30
TD	65536
SOLVENT	CDCl3
NS	6603
DS	4
SWH	24038.461 Hz
FIDRES	0.366798 Hz
AQ	1.3631988 sec
RG	2050
DW	20.800 usec
DE	6.50 usec
TE	295.4 K
D1	2.00000000 sec
D11	0.03000000 sec
TD0	1

======== CHANNEL f1 ========

NUC1	13C
P1	8.25 usec
PL1	-2.10 dB
PL1W	60.29227829 W
SFO1	100.6228298 MHz

======== CHANNEL f2 ========

CPDPRG2	waltz16
NUC2	1H
PCPD2	90.00 usec
PL2	0.50 dB
PL12	16.21 dB
PL2W	12.76071072 W
PL12W	0.34266910 W
SFO2	400.1316005 MHz
SI	32768
SF	100.6127729 MHz
WDW	EM
SSB	0
LB	1.00 Hz
GB	0
PC	1.40

CWVI-121

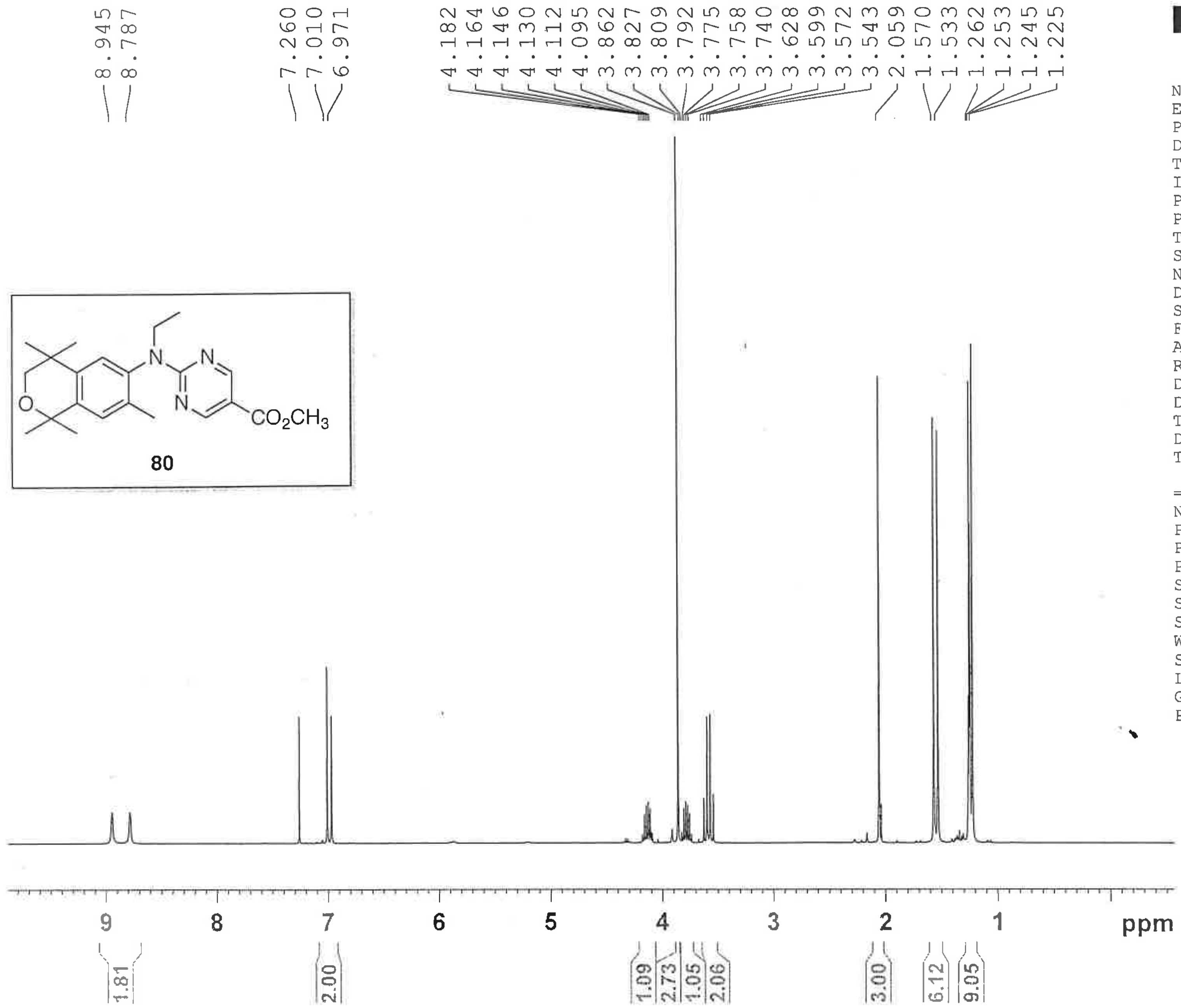

80

NAME	CWVI-121
EXPNO	1
PROCNO	1
Date_	20180725
Time	13.47
INSTRUM	spect
PROBHD	5 mm PABBO BB-
PULPROG	zg30
TD	65536
SOLVENT	CDCl3
NS	16
DS	2
SWH	8223.685 Hz
FIDRES	0.125483 Hz
AQ	3.9846387 sec
RG	4
DW	60.800 usec
DE	6.50 usec
TE	294.3 K
D1	1.00000000 sec
TD0	1

======== CHANNEL f1 ========

NUC1	1H
P1	14.75 usec
PL1	0.50 dB
PL1W	12.76071072 W
SFO1	400.1324710 MHz
SI	32768
SF	400.1300099 MHz
WDW	EM
SSB	0
LB	0.30 Hz
GB	0
PC	1.00

CWVI-121

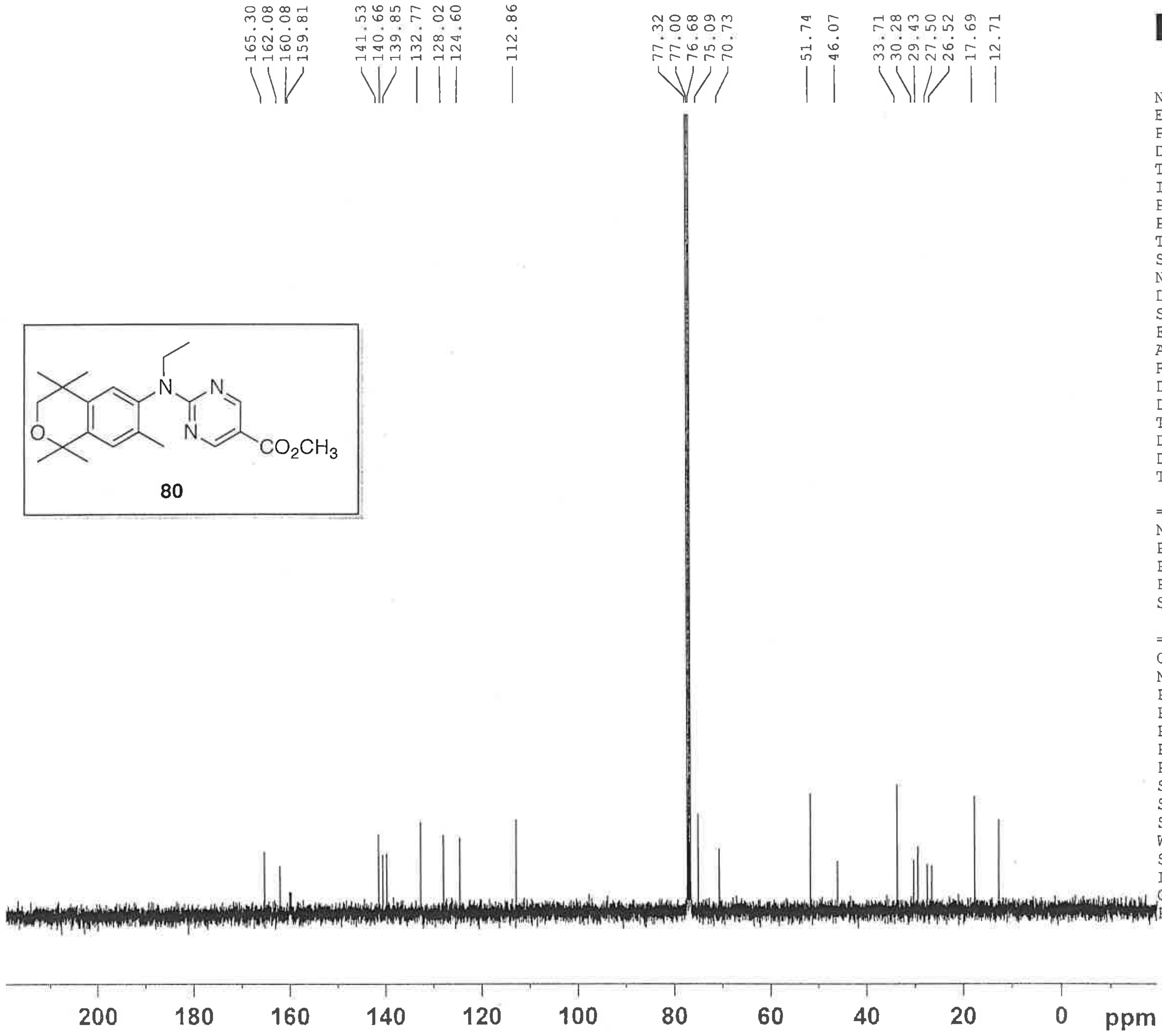

80

BRUKER

NAME	CWVI-121
EXPNO	2
PROCNO	1
Date_	20180725
Time	13.54
INSTRUM	spect
PROBHD	5 mm PABBO BB-
PULPROG	zgig30
TD	65536
SOLVENT	CDCl3
NS	133
DS	4
SWH	24038.461 Hz
FIDRES	0.366798 Hz
AQ	1.3631988 sec
RG	2050
DW	20.800 usec
DE	6.50 usec
TE	294.7 K
D1	2.00000000 sec
D11	0.03000000 sec
TD0	1

======== CHANNEL f1 ========

NUC1	13C
P1	8.25 usec
PL1	-2.10 dB
PL1W	60.29227829 W
SFO1	100.6228298 MHz

======== CHANNEL f2 ========

CPDPRG2	waltz16
NUC2	1H
PCPD2	90.00 usec
PL2	0.50 dB
PL12	16.21 dB
PL2W	12.76071072 W
PL12W	0.34266910 W
SFO2	400.1316005 MHz
SI	32768
SF	100.6127729 MHz
WDW	EM
SSB	0
LB	1.00 Hz
GB	0
PC	1.40

CWVI-119

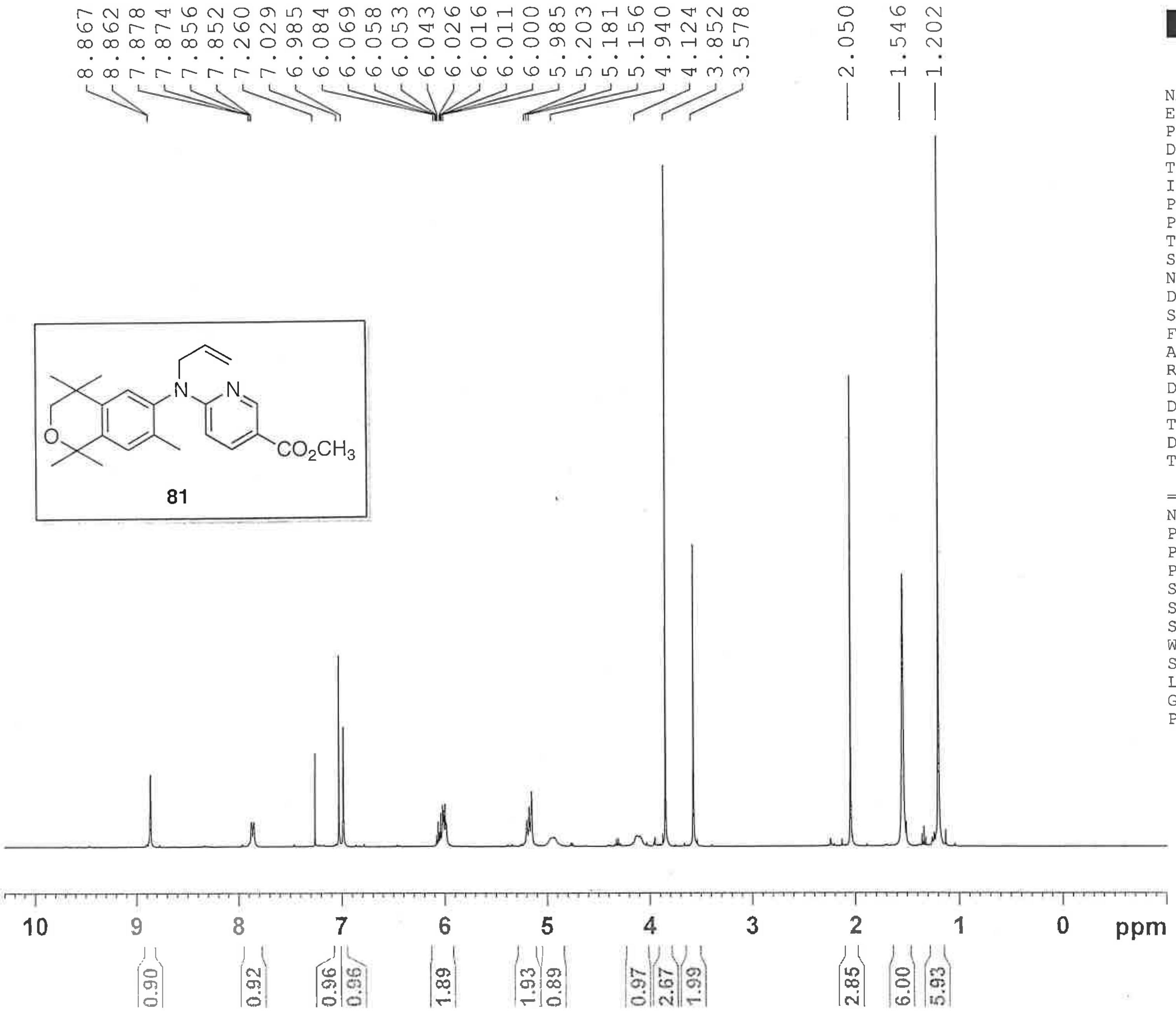

81

NAME	CWVI-119
EXPNO	1
PROCNO	1
Date_	20180725
Time_	21.15
INSTRUM	spect
PROBHD	5 mm PABBO BB-
PULPROG	zg30
TD	65536
SOLVENT	CDCl3
NS	16
DS	2
SWH	8223.685 Hz
FIDRES	0.125483 Hz
AQ	3.9846387 sec
RG	4
DW	60.800 usec
DE	6.50 usec
TE	294.9 K
D1	1.00000000 sec
TD0	1

======== CHANNEL f1 ========

NUC1	1H
P1	14.75 usec
PL1	0.50 dB
PL1W	12.76071072 W
SFO1	400.1324710 MHz
SI	32768
SF	400.1300099 MHz
WDW	EM
SSB	0
LB	0.30 Hz
GB	0
PC	1.00

CWVI-119

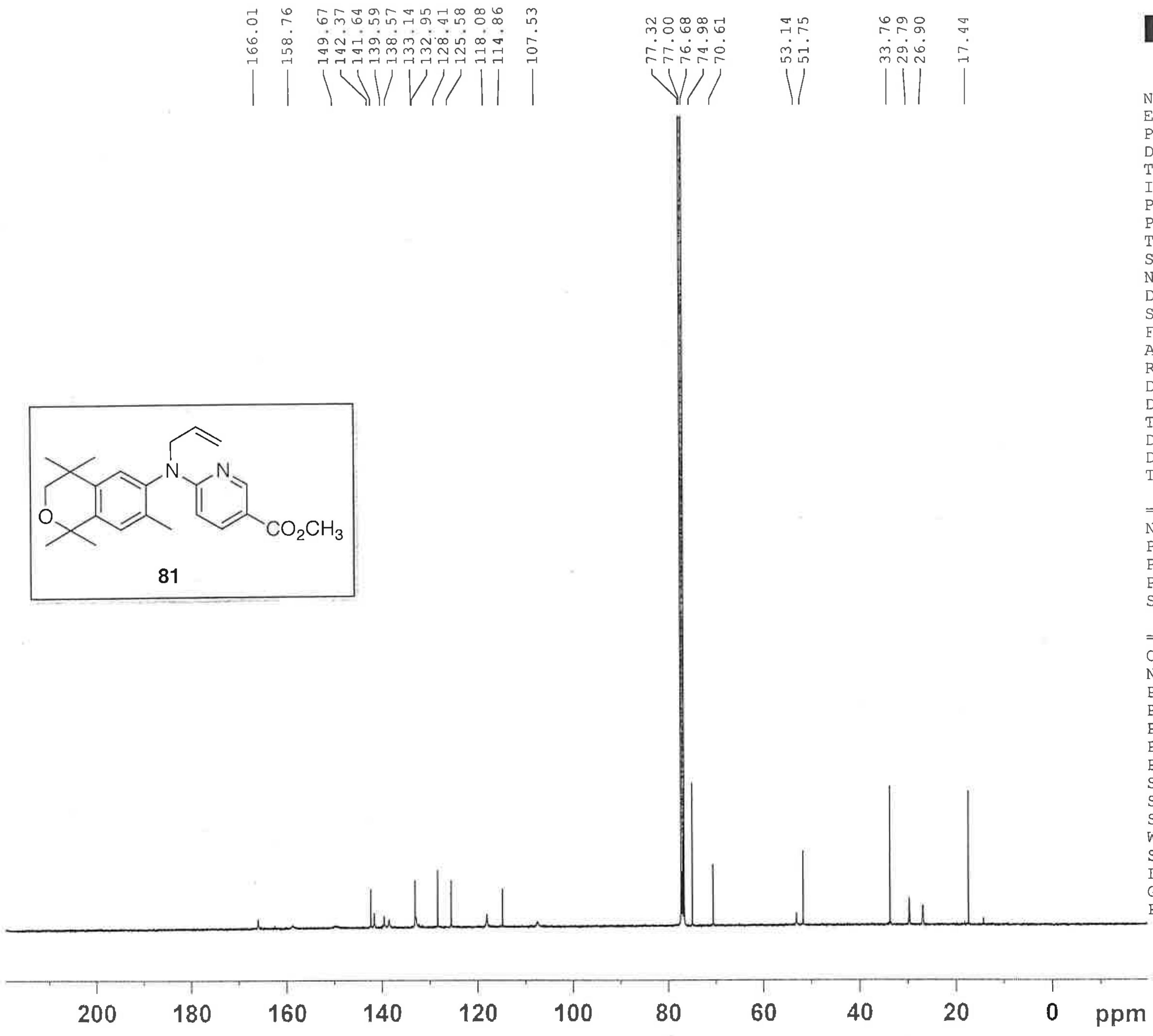

NAME	CWVI-119	
EXPNO	3	
PROCNO	1	
Date_	20180726	
Time	12.30	
INSTRUM	spect	
PROBHD	5 mm PABBO BB-	
PULPROG	zgig30	
TD	65536	
SOLVENT	CDCl3	
NS	8193	
DS	4	
SWH	24038.461	Hz
FIDRES	0.366798	Hz
AQ	1.3631988	sec
RG	2050	
DW	20.800	usec
DE	6.50	usec
TE	295.0	K
D1	2.00000000	sec
D11	0.03000000	sec
TD0	1	

======== CHANNEL f1 ========

NUC1	13C	
P1	8.25	usec
PL1	-2.10	dB
PL1W	60.29227829	W
SFO1	100.6228298	MHz

======== CHANNEL f2 ========

CPDPRG2	waltz16	
NUC2	1H	
PCPD2	90.00	usec
PL2	0.50	dB
PL12	16.21	dB
PL2W	12.76071072	W
PL12W	0.34266910	W
SFO2	400.1316005	MHz
SI	32768	
SF	100.6127736	MHz
WDW	EM	
SSB	0	
LB	1.00	Hz
GB	0	
PC	1.40	

CWVI-147

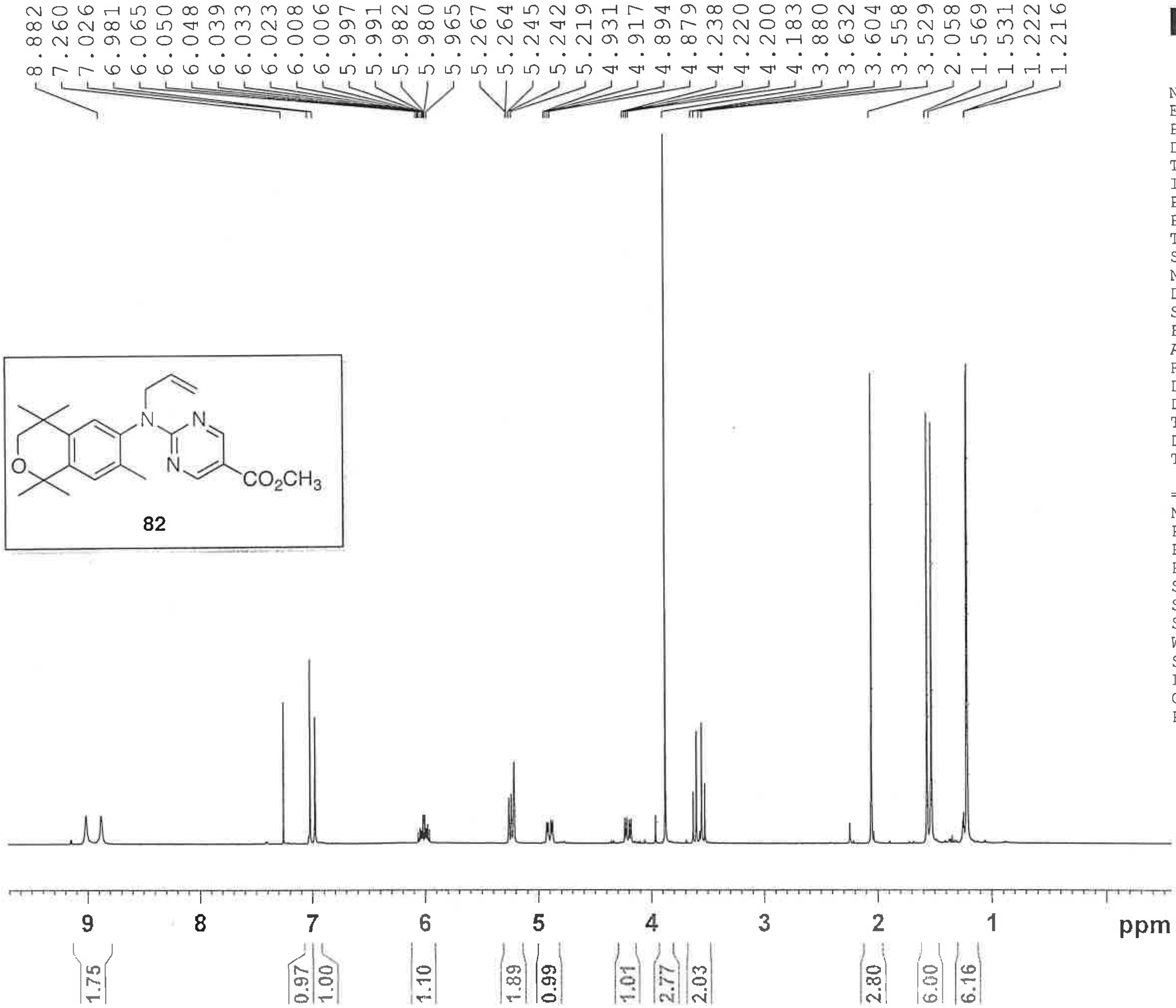

82

BRUKER

NAME	CWVI-147
EXPNO	1
PROCNO	1
Date_	20180726
Time	17.28
INSTRUM	spect
PROBHD	5 mm PABBO BB-
PULPROG	zg30
TD	65536
SOLVENT	CDCl3
NS	16
DS	2
SWH	8223.685 Hz
FIDRES	0.125483 Hz
AQ	3.9846387 sec
RG	4
DW	60.800 usec
DE	6.50 usec
TE	295.3 K
D1	1.00000000 sec
TD0	1

======== CHANNEL f1 ========

NUC1	1H
P1	14.75 usec
PL1	0.50 dB
PL1W	12.76071072 W
SFO1	400.1324710 MHz
SI	32768
SF	400.1300099 MHz
WDW	EM
SSB	0
LB	0.30 Hz
GB	0
PC	1.00

CWVI-147

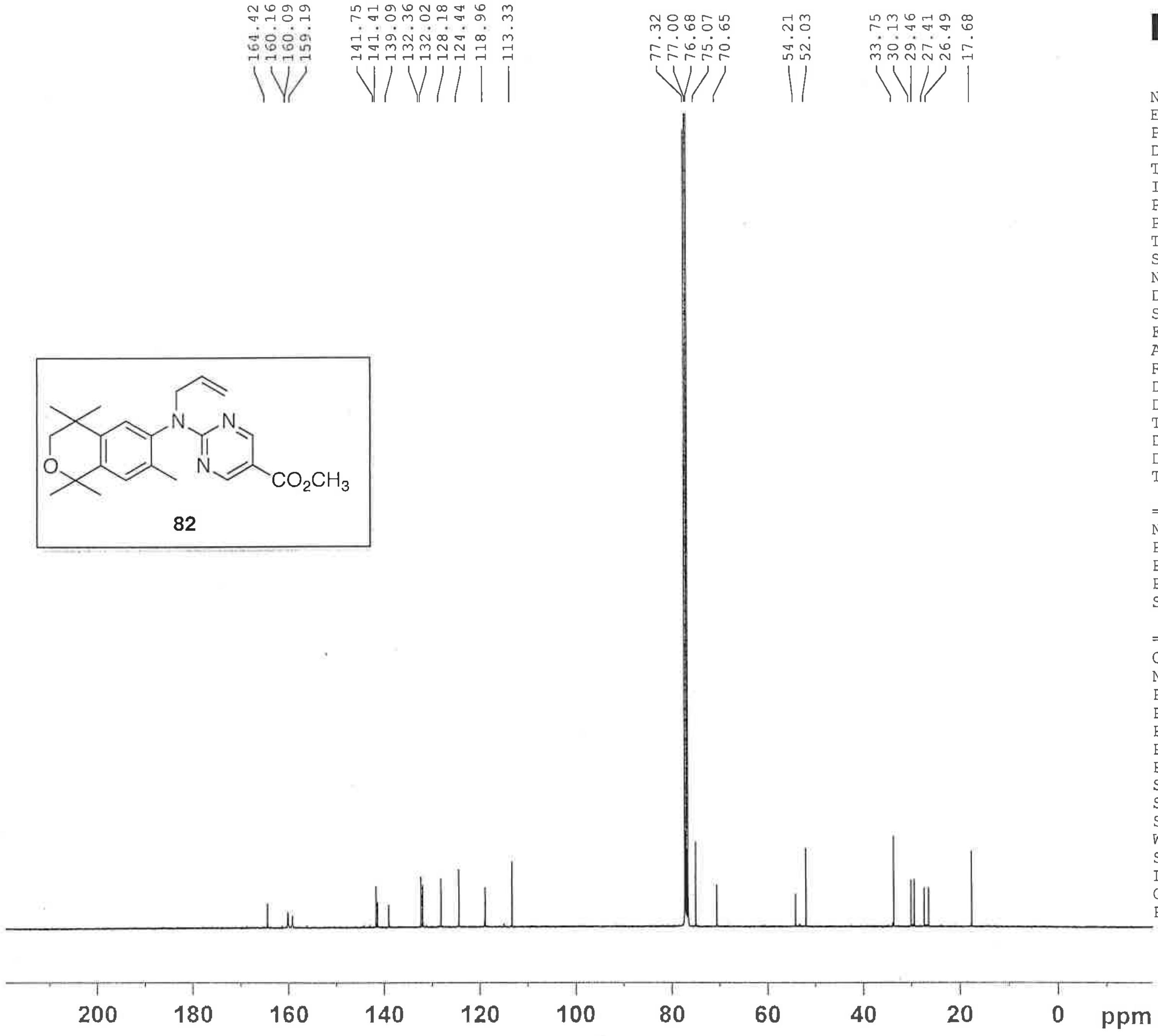

82

NAME	CWVI-147
EXPNO	2
PROCNO	1
Date_	20180726
Time	17.38
INSTRUM	spect
PROBHD	5 mm PABBO BB-
PULPROG	zgig30
TD	65536
SOLVENT	CDCl3
NS	16384
DS	4
SWH	24038.461 Hz
FIDRES	0.366798 Hz
AQ	1.3631988 sec
RG	2050
DW	20.800 usec
DE	6.50 usec
TE	295.5 K
D1	2.00000000 sec
D11	0.03000000 sec
TD0	1

======== CHANNEL f1 ========

NUC1	13C
P1	8.25 usec
PL1	-2.10 dB
PL1W	60.29227829 W
SFO1	100.6228298 MHz

======== CHANNEL f2 ========

CPDPRG2	waltz16
NUC2	1H
PCPD2	90.00 usec
PL2	0.50 dB
PL12	16.21 dB
PL2W	12.76071072 W
PL12W	0.34266910 W
SFO2	400.1316005 MHz
SI	32768
SF	100.6127729 MHz
WDW	EM
SSB	0
LB	1.00 Hz
GB	0
PC	1.40

CWVI-177

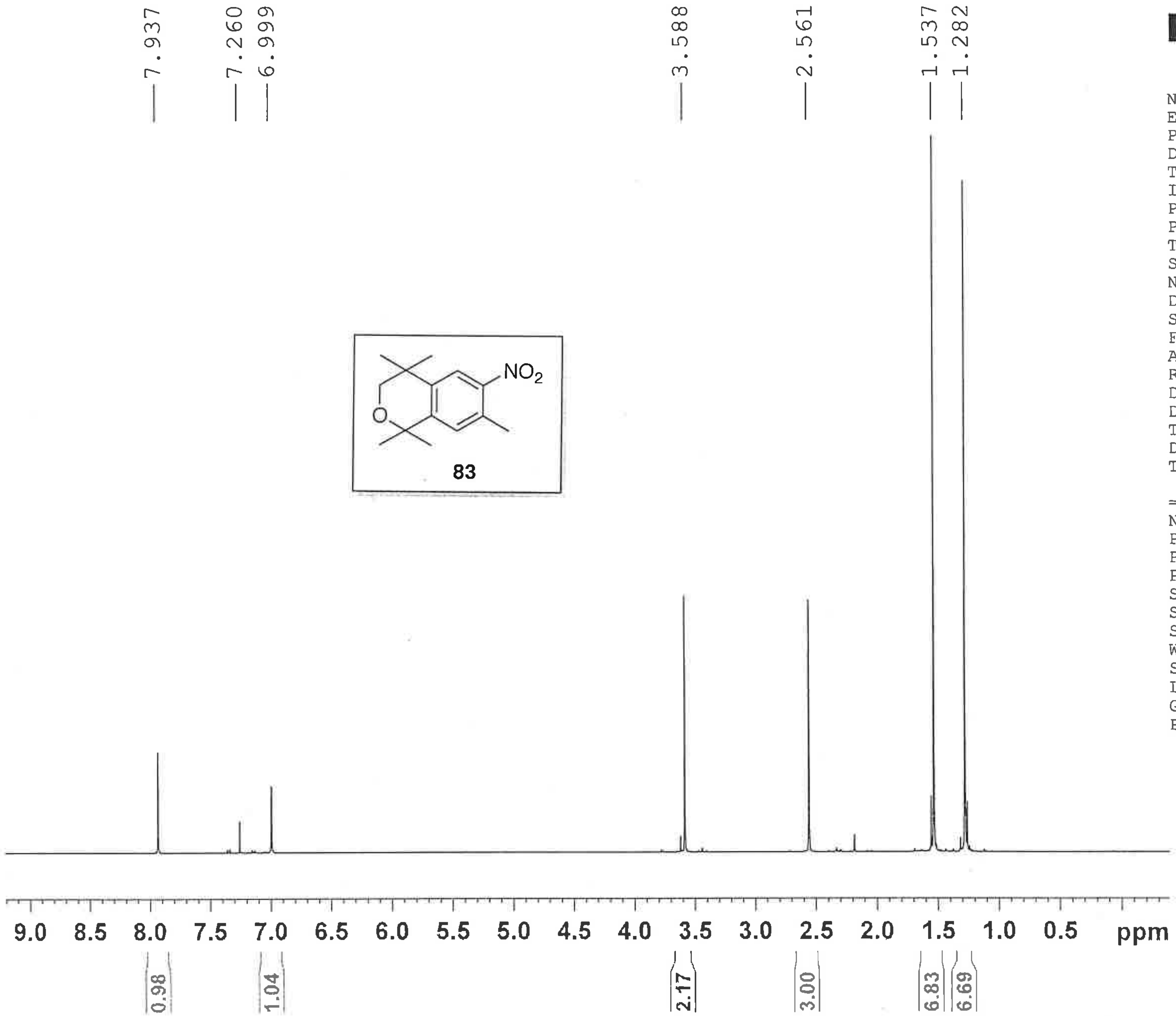

NAME	CWVI-177
EXPNO	1
PROCNO	1
Date_	20180828
Time	13.19
INSTRUM	spect
PROBHD	5 mm PABBO BB-
PULPROG	zg30
TD	65536
SOLVENT	CDCl3
NS	16
DS	2
SWH	8223.685 Hz
FIDRES	0.125483 Hz
AQ	3.9846387 sec
RG	4
DW	60.800 usec
DE	6.50 usec
TE	292.1 K
D1	1.00000000 sec
TD0	1

======== CHANNEL f1 ========

NUC1	1H
P1	14.75 usec
PL1	0.50 dB
PL1W	12.76071072 W
SFO1	400.1324710 MHz
SI	32768
SF	400.1300099 MHz
WDW	EM
SSB	0
LB	0.30 Hz
GB	0
PC	1.00

CWVI-177

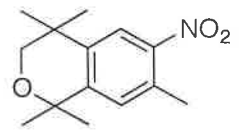

83

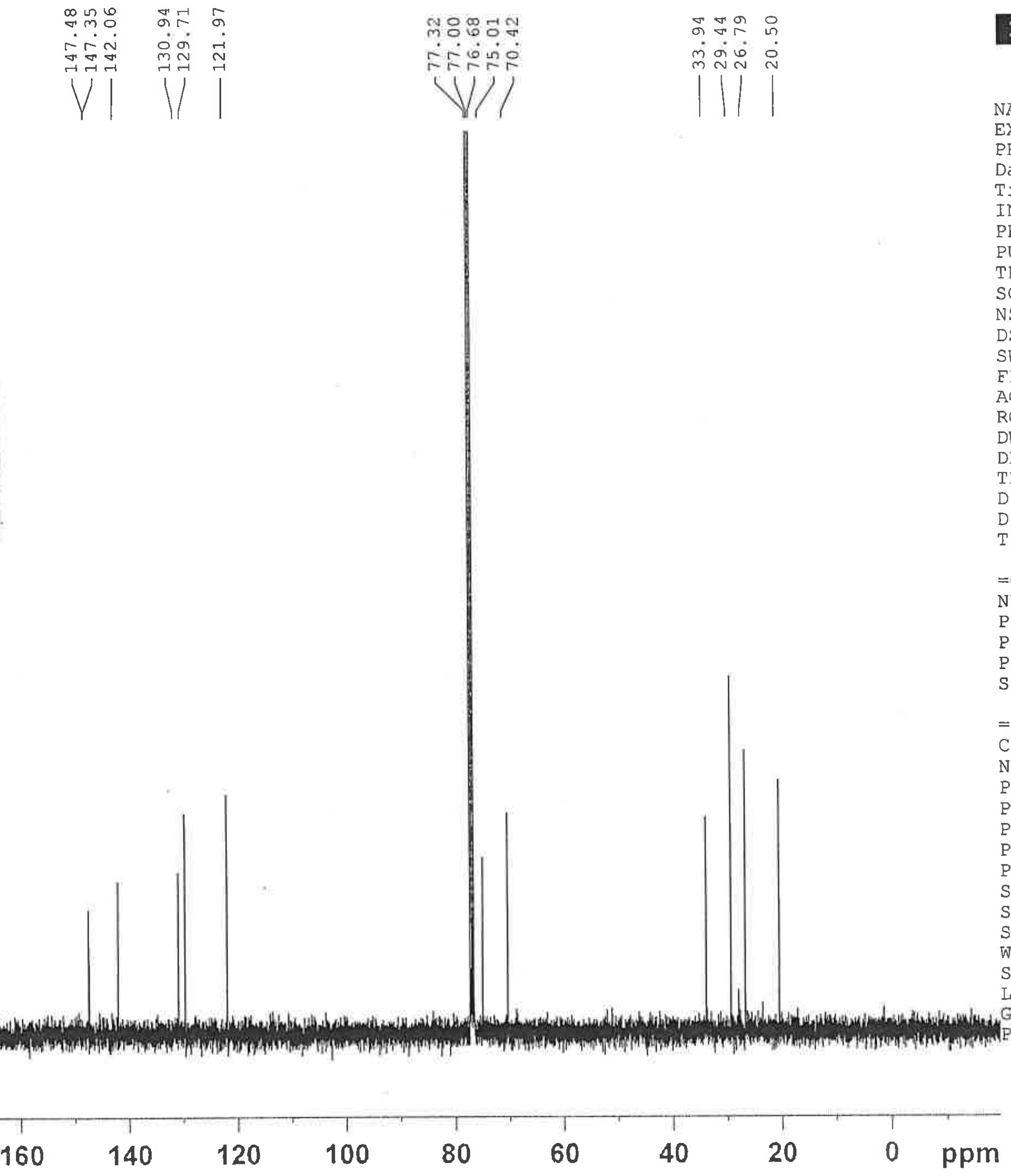

NAME	CWVI-177
EXPNO	2
PROCNO	1
Date_	20180828
Time_	13.23
INSTRUM	spect
PROBHD	5 mm PABBO BB-
PULPROG	zgig30
TD	65536
SOLVENT	CDCl3
NS	66
DS	4
SWH	24038.461 Hz
FIDRES	0.366798 Hz
AQ	1.3631988 sec
RG	2050
DW	20.800 usec
DE	6.50 usec
TE	292.5 K
D1	2.00000000 sec
D11	0.03000000 sec
TD0	1

======== CHANNEL f1 ========

NUC1	13C
P1	8.25 usec
PL1	-2.10 dB
PL1W	60.29227829 W
SFO1	100.6228298 MHz

======== CHANNEL f2 ========

CPDPRG2	waltz16
NUC2	1H
PCPD2	90.00 usec
PL2	0.50 dB
PL12	16.21 dB
PL2W	12.76071072 W
PL12W	0.34266910 W
SFO2	400.1316005 MHz
SI	32768
SF	100.6127736 MHz
WDW	EM
SSB	0
LB	1.00 Hz
GB	0
PC	1.40

CWVI-181

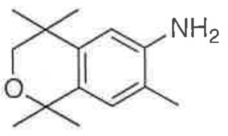

84

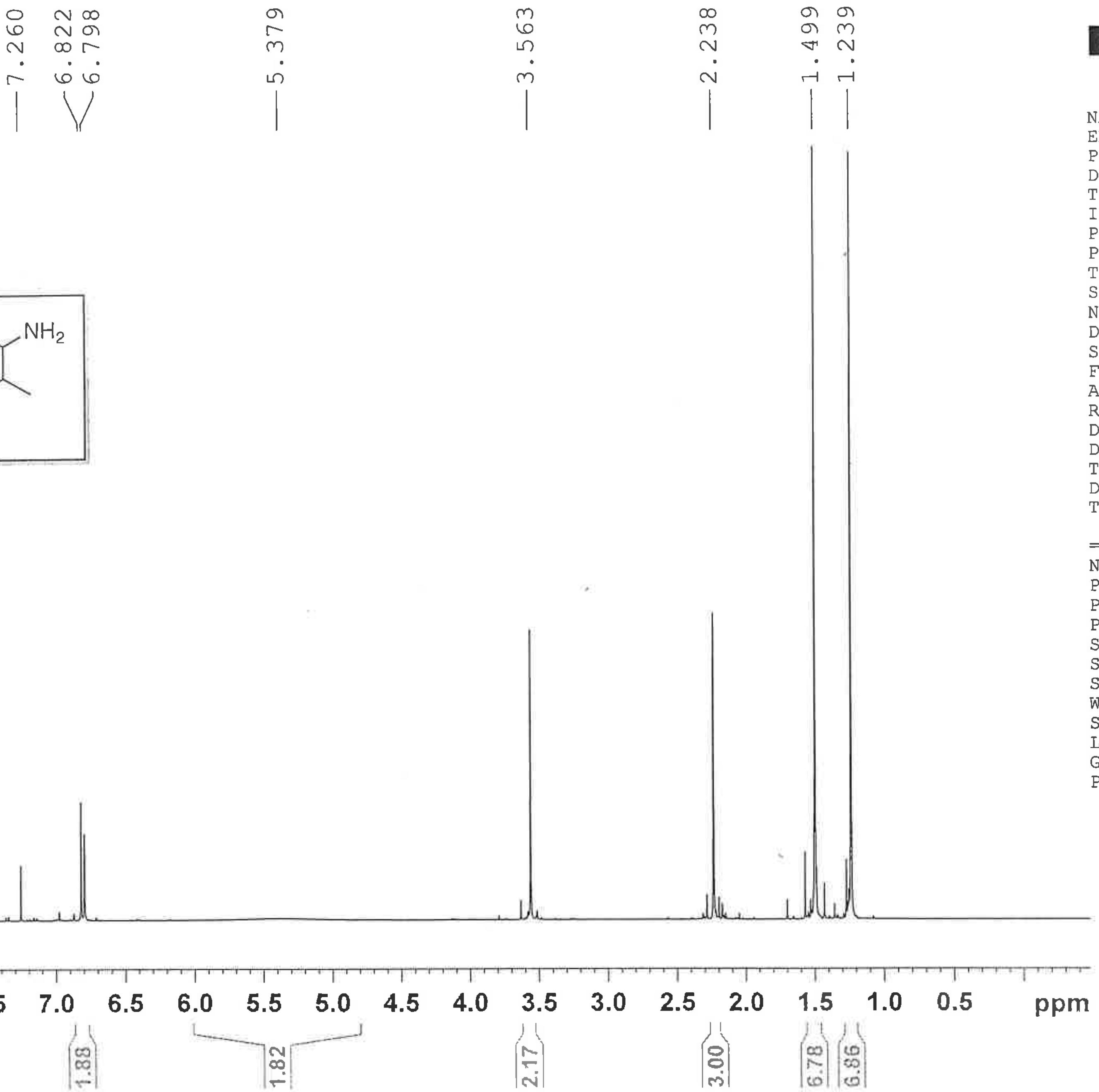

NAME	CWVI-181
EXPNO	1
PROCNO	1
Date_	20180828
Time	13.29
INSTRUM	spect
PROBHD	5 mm PABBO BB-
PULPROG	zg30
TD	65536
SOLVENT	CDCl3
NS	16
DS	2
SWH	8223.685 Hz
FIDRES	0.125483 Hz
AQ	3.9846387 sec
RG	4
DW	60.800 usec
DE	6.50 usec
TE	292.4 K
D1	1.00000000 sec
TD0	1

======== CHANNEL f1 ========

NUC1	1H
P1	14.75 usec
PL1	0.50 dB
PL1W	12.76071072 W
SFO1	400.1324710 MHz
SI	32768
SF	400.1300099 MHz
WDW	EM
SSB	0
LB	0.30 Hz
GB	0
PC	1.00

CWVI-181

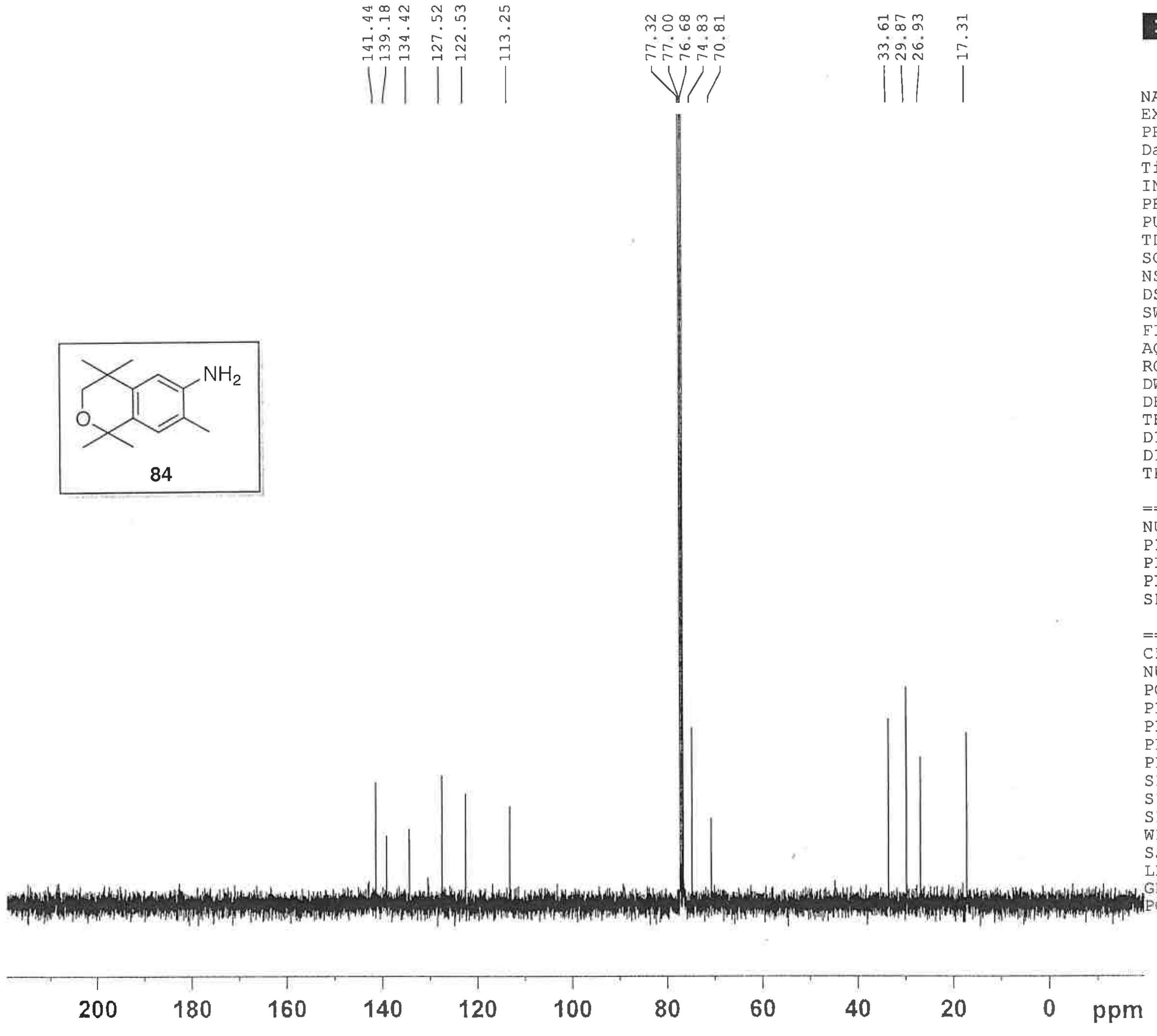

84

NAME	CWVI-181
EXPNO	2
PROCNO	1
Date_	20180828
Time	13.33
INSTRUM	spect
PROBHD	5 mm PABBO BB-
PULPROG	zgig30
TD	65536
SOLVENT	CDCl3
NS	58
DS	4
SWH	24038.461 Hz
FIDRES	0.366798 Hz
AQ	1.3631988 sec
RG	2050
DW	20.800 usec
DE	6.50 usec
TE	292.7 K
D1	2.00000000 sec
D11	0.03000000 sec
TD0	1
======== CHANNEL f1 ========	
NUC1	13C
P1	8.25 usec
PL1	-2.10 dB
PL1W	60.29227829 W
SFO1	100.6228298 MHz
======== CHANNEL f2 ========	
CPDPRG2	waltz16
NUC2	1H
PCPD2	90.00 usec
PL2	0.50 dB
PL12	16.21 dB
PL2W	12.76071072 W
PL12W	0.34266910 W
SFO2	400.1316005 MHz
SI	32768
SF	100.6127751 MHz
WDW	EM
SSB	0
LB	1.00 Hz
GB	0
PC	1.40

CWVI-183 f 12-16

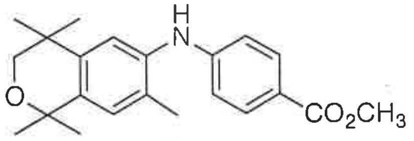

86

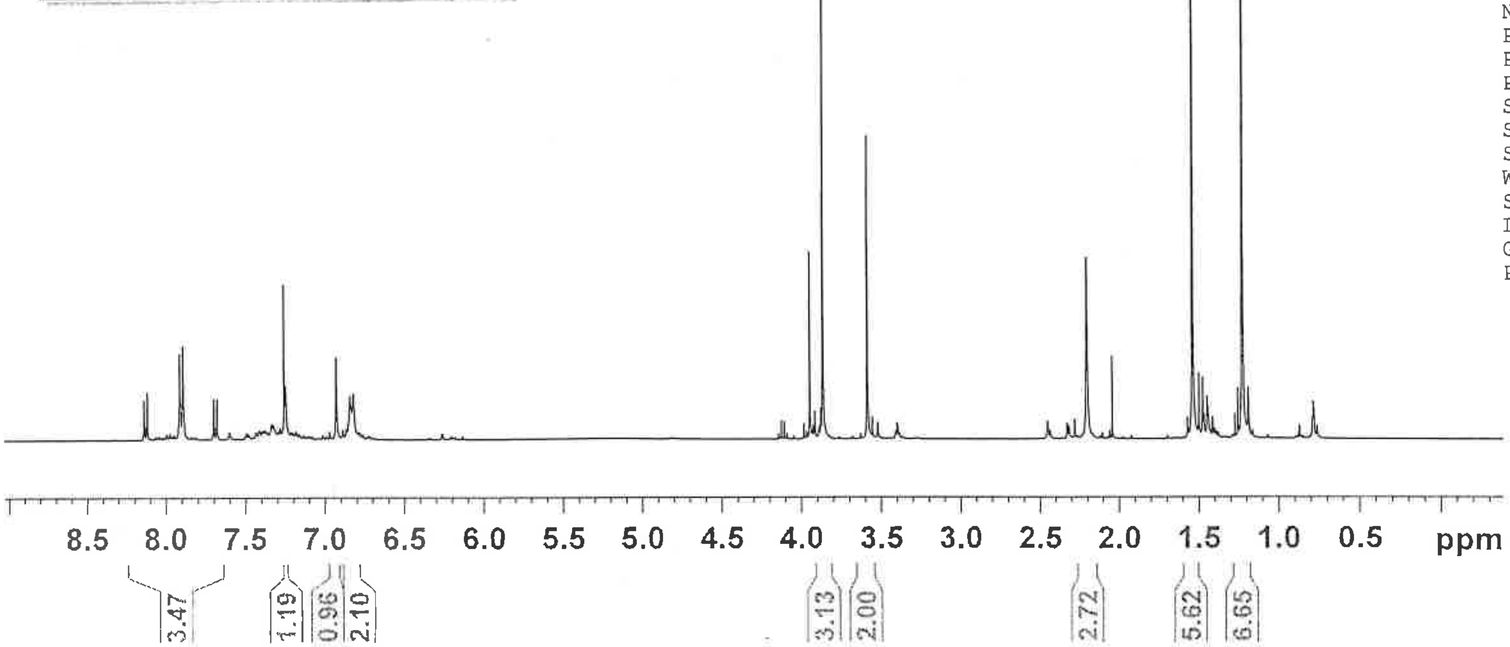

NAME	CWVI-183
EXPNO	3
PROCNO	1
Date_	20180828
Time	13.49
INSTRUM	spect
PROBHD	5 mm PABBO BB-
PULPROG	zg30
TD	65536
SOLVENT	CDCl3
NS	16
DS	2
SWH	8223.685 Hz
FIDRES	0.125483 Hz
AQ	3.9846387 sec
RG	4
DW	60.800 usec
DE	6.50 usec
TE	292.8 K
D1	1.00000000 sec
TD0	1

======== CHANNEL f1 ========

NUC1	1H
P1	14.75 usec
PL1	0.50 dB
PL1W	12.76071072 W
SFO1	400.1324710 MHz
SI	32768
SF	400.1300099 MHz
WDW	EM
SSB	0
LB	0.30 Hz
GB	0
PC	1.00

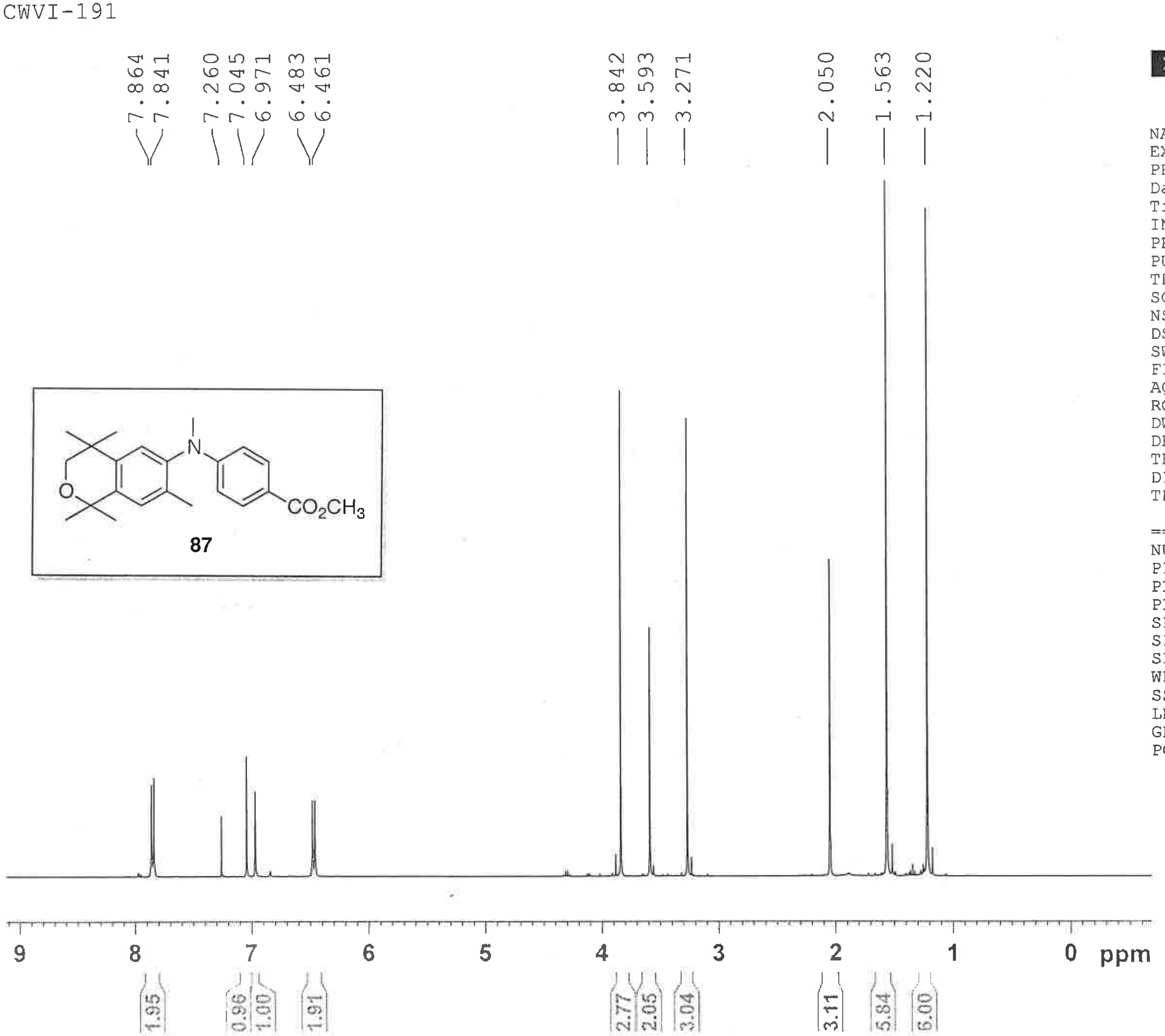

NAME	CWVI-191
EXPNO	1
PROCNO	1
Date_	20180906
Time	12.54
INSTRUM	spect
PROBHD	5 mm PABBO BB-
PULPROG	zg30
TD	65536
SOLVENT	CDCl3
NS	16
DS	2
SWH	8223.685 Hz
FIDRES	0.125483 Hz
AQ	3.9846387 sec
RG	4
DW	60.800 usec
DE	6.50 usec
TE	293.6 K
D1	1.00000000 sec
TD0	1

======== CHANNEL f1 ========

NUC1	1H
P1	14.75 usec
PL1	0.50 dB
PL1W	12.76071072 W
SFO1	400.1324710 MHz
SI	32768
SF	400.1300099 MHz
WDW	EM
SSB	0
LB	0.30 Hz
GB	0
PC	1.00

CWVI-191

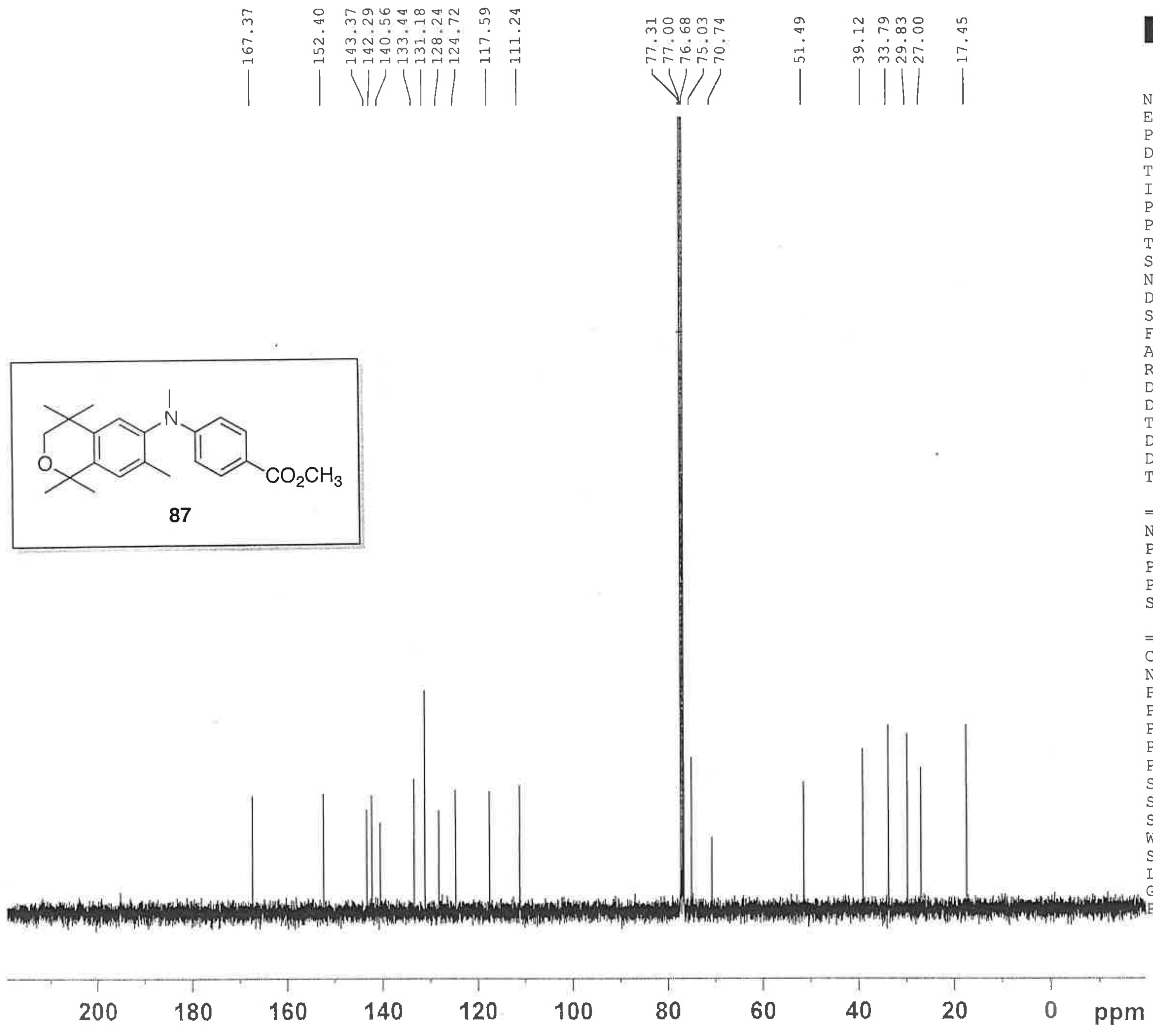

87

NAME	CWVI-191
EXPNO	2
PROCNO	1
Date_	20180906
Time	12.58
INSTRUM	spect
PROBHD	5 mm PABBO BB-
PULPROG	zgig30
TD	65536
SOLVENT	CDCl3
NS	100
DS	4
SWH	24038.461 Hz
FIDRES	0.366798 Hz
AQ	1.3631988 sec
RG	2050
DW	20.800 usec
DE	6.50 usec
TE	293.8 K
D1	2.00000000 sec
D11	0.03000000 sec
TD0	1

======== CHANNEL f1 ========

NUC1	13C
P1	8.25 usec
PL1	-2.10 dB
PL1W	60.29227829 W
SFO1	100.6228298 MHz

======== CHANNEL f2 ========

CPDPRG2	waltz16
NUC2	1H
PCPD2	90.00 usec
PL2	0.50 dB
PL12	16.21 dB
PL2W	12.76071072 W
PL12W	0.34266910 W
SFO2	400.1316005 MHz
SI	32768
SF	100.6127744 MHz
WDW	EM
SSB	0
LB	1.00 Hz
GB	0
PC	1.40

CWVI-189

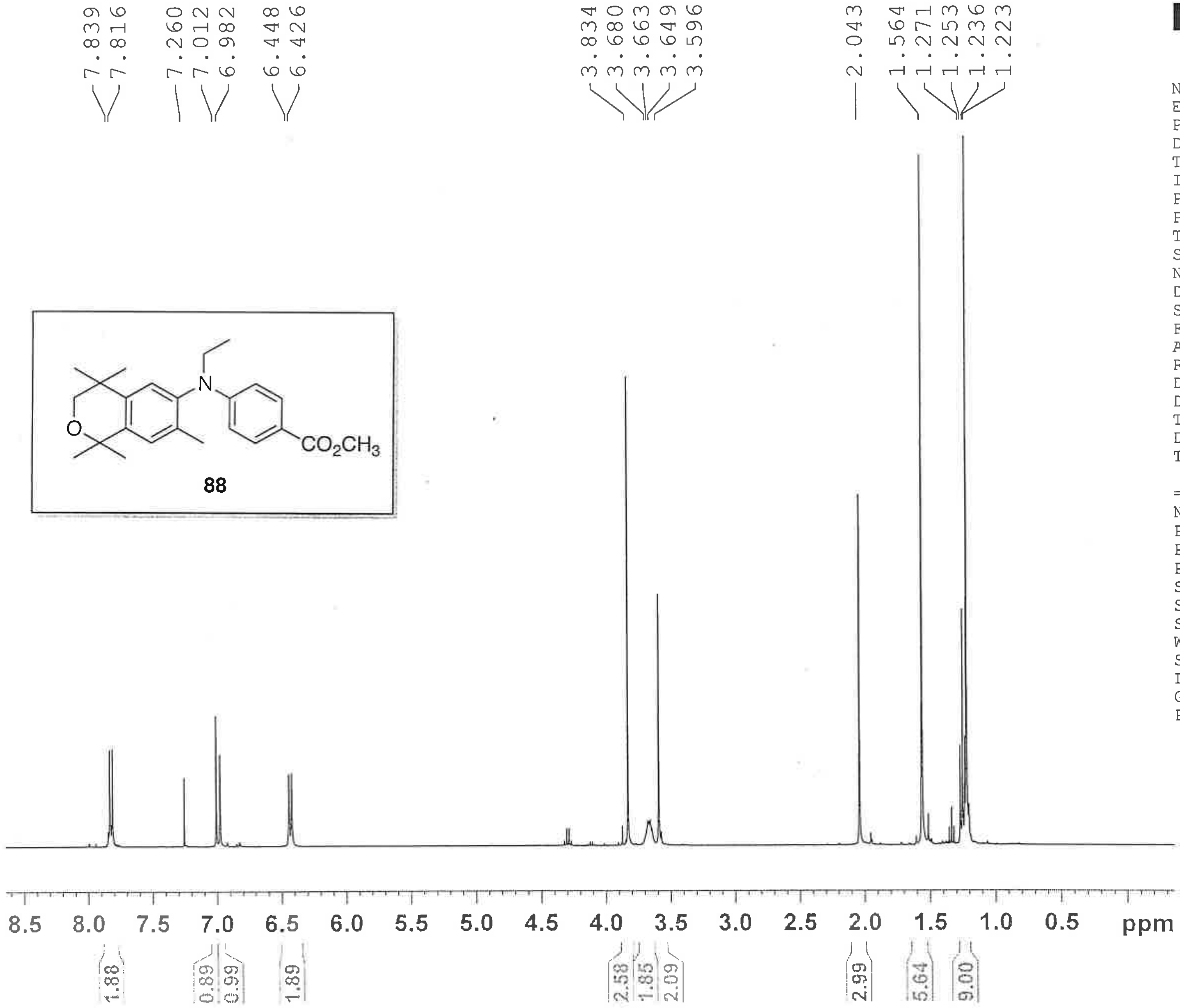

88

NAME	CWVI-189
EXPNO	1
PROCNO	1
Date_	20180906
Time	12.39
INSTRUM	spect
PROBHD	5 mm PABBO BB-
PULPROG	zg30
TD	65536
SOLVENT	CDCl3
NS	16
DS	2
SWH	8223.685 Hz
FIDRES	0.125483 Hz
AQ	3.9846387 sec
RG	4
DW	60.800 usec
DE	6.50 usec
TE	293.5 K
D1	1.00000000 sec
TD0	1

======== CHANNEL f1 ========

NUC1	1H
P1	14.75 usec
PL1	0.50 dB
PL1W	12.76071072 W
SFO1	400.1324710 MHz
SI	32768
SF	400.1300099 MHz
WDW	EM
SSB	0
LB	0.30 Hz
GB	0
PC	1.00

CWVI-189

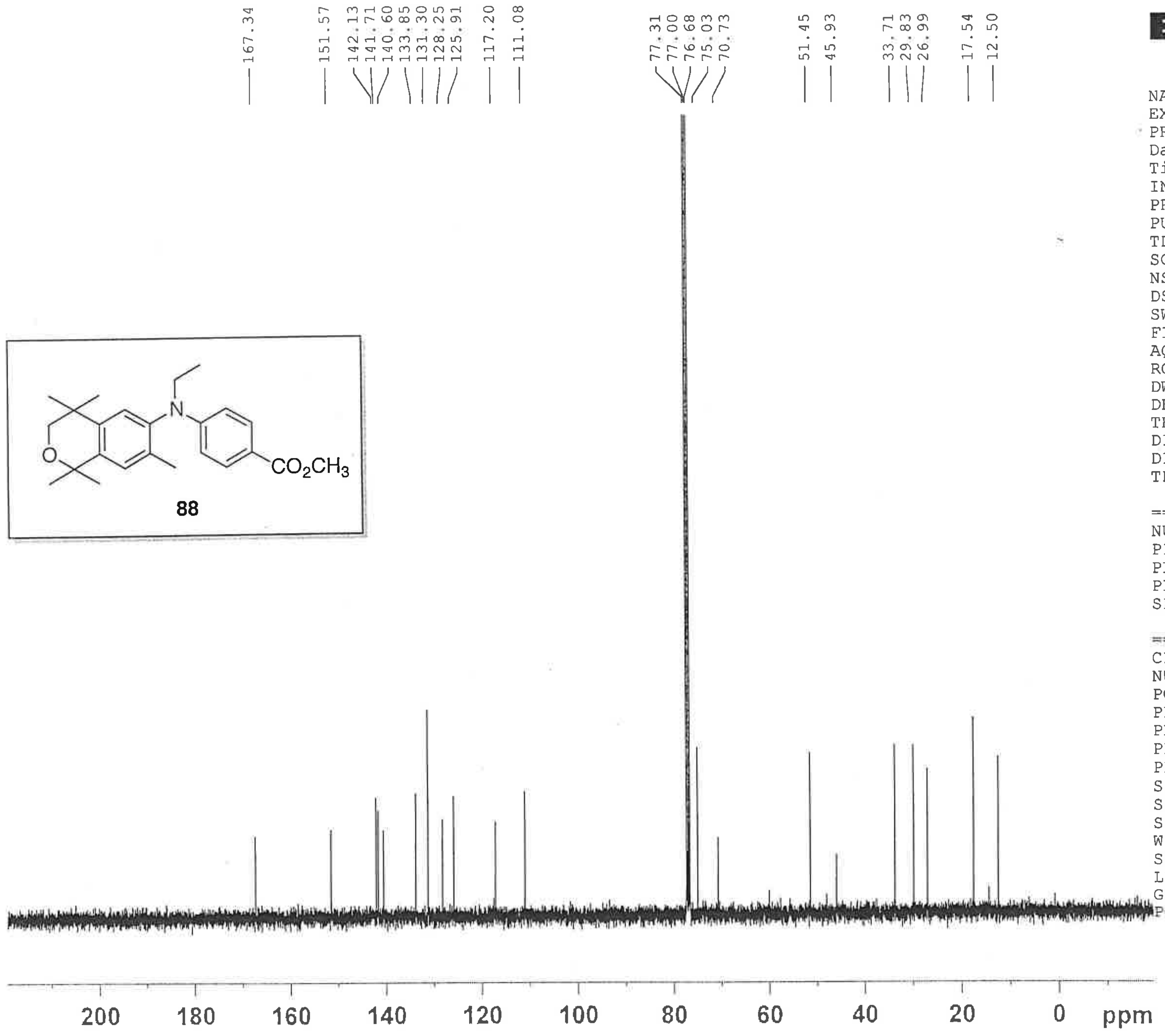

BRUKER

NAME	CWVI-189
EXPNO	2
PROCNO	1
Date_	20180906
Time	12.45
INSTRUM	spect
PROBHD	5 mm PABBO BB-
PULPROG	zgig30
TD	65536
SOLVENT	CDCl3
NS	151
DS	4
SWH	24038.461 Hz
FIDRES	0.366798 Hz
AQ	1.3631988 sec
RG	2050
DW	20.800 usec
DE	6.50 usec
TE	293.8 K
D1	2.00000000 sec
D11	0.03000000 sec
TD0	1

======== CHANNEL f1 ========

NUC1	13C
P1	8.25 usec
PL1	-2.10 dB
PL1W	60.29227829 W
SFO1	100.6228298 MHz

======== CHANNEL f2 ========

CPDPRG2	waltz16
NUC2	1H
PCPD2	90.00 usec
PL2	0.50 dB
PL12	16.21 dB
PL2W	12.76071072 W
PL12W	0.34266910 W
SFO2	400.1316005 MHz
SI	32768
SF	100.6127744 MHz
WDW	EM
SSB	0
LB	1.00 Hz
GB	0
PC	1.40

CWVI-193

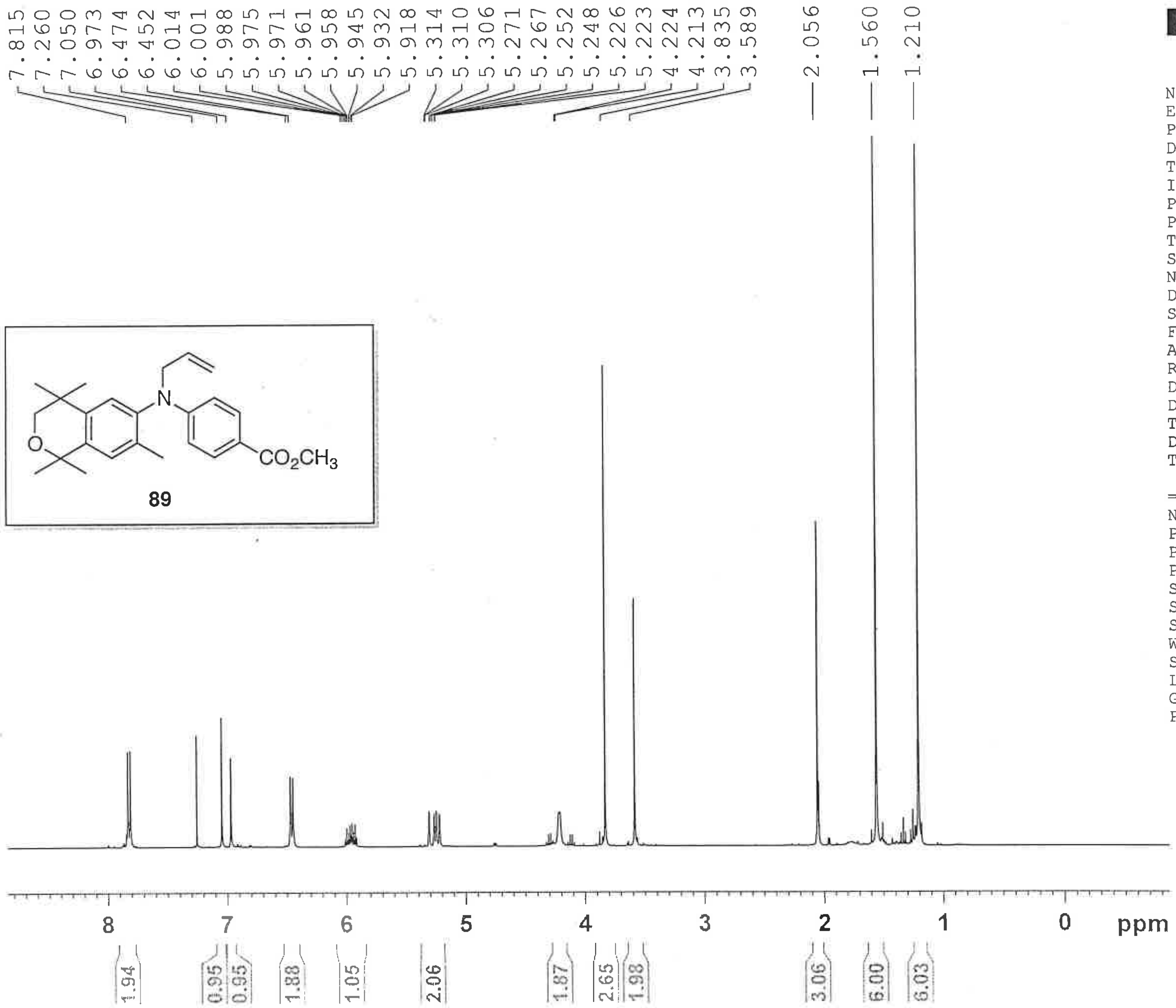

BRUKER

NAME	CWVI-193
EXPNO	1
PROCNO	1
Date_	20180906
Time_	13.06
INSTRUM	spect
PROBHD	5 mm PABBO BB-
PULPROG	zg30
TD	65536
SOLVENT	CDCl3
NS	16
DS	2
SWH	8223.685 Hz
FIDRES	0.125483 Hz
AQ	3.9846387 sec
RG	4
DW	60.800 usec
DE	6.50 usec
TE	293.6 K
D1	1.00000000 sec
TD0	1

======== CHANNEL f1 ========

NUC1	1H
P1	14.75 usec
PL1	0.50 dB
PL1W	12.76071072 W
SFO1	400.1324710 MHz
SI	32768
SF	400.1300099 MHz
WDW	EM
SSB	0
LB	0.30 Hz
GB	0
PC	1.00

CWVI-193

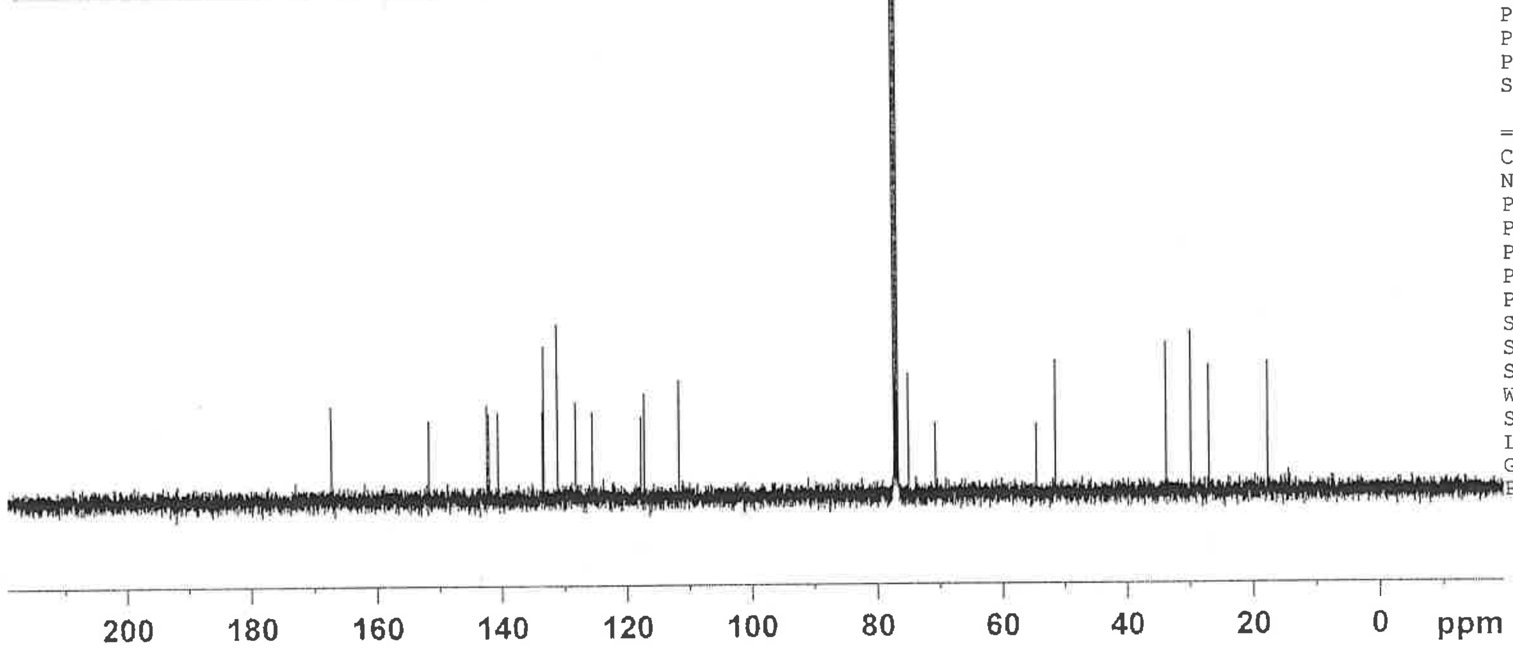

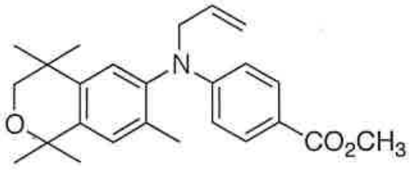

89

BRUKER

NAME	CWVI-193
EXPNO	2
PROCNO	1
Date_	20180906
Time	13.14
INSTRUM	spect
PROBHD	5 mm PABBO BB-
PULPROG	zgig30
TD	65536
SOLVENT	CDCl3
NS	200
DS	4
SWH	24038.461 Hz
FIDRES	0.366798 Hz
AQ	1.3631988 sec
RG	2050
DW	20.800 usec
DE	6.50 usec
TE	293.9 K
D1	2.00000000 sec
D11	0.03000000 sec
TD0	1

======== CHANNEL f1 ========

NUC1	13C
P1	8.25 usec
PL1	-2.10 dB
PL1W	60.29227829 W
SFO1	100.6228298 MHz

======== CHANNEL f2 ========

CPDPRG2	waltz16
NUC2	1H
PCPD2	90.00 usec
PL2	0.50 dB
PL12	16.21 dB
PL2W	12.76071072 W
PL12W	0.34266910 W
SFO2	400.1316005 MHz
SI	32768
SF	100.6127729 MHz
WDW	EM
SSB	0
LB	1.00 Hz
GB	0
PC	1.40
